# Rhodium and Iridium Complexes of Anionic Thione and Selone Ligands Derived from Anionic N‐Heterocyclic Carbenes

**DOI:** 10.1002/chem.202104139

**Published:** 2021-12-22

**Authors:** Luong Phong Ho, Angelika Neitzel, Thomas Bannenberg, Matthias Tamm

**Affiliations:** ^1^ Institut für Anorganische und Analytische Chemie Technische Universität Braunschweig Hagenring 30 38106 Braunschweig Germany

**Keywords:** Iridium, N-heterocyclic carbine, rhodium, selenium, sulfur

## Abstract

The lithium salts of anionic N‐heterocyclic thiones and selones [{(WCA‐IDipp)E}Li(toluene)] (**1**: E=S; **2**: E=Se; WCA=B(C_6_F_5_)_3_, IDipp=1,3‐bis(2,6‐diisopropylphenyl)imidazolin‐2‐ylidene), which contain a weakly coordinating anionic (WCA) borate moiety in the imidazole backbone were reacted with Me_3_SiCl, to furnish the silylated adducts (WCA‐IDipp)ESiMe_3_ (**3**: E=S; **4**: E=Se). The reaction of the latter with [(*η* ^5^‐C_5_Me_5_)MCl_2_]_2_ (M=Rh, Ir) afforded the rhodium(III) and iridium(III) half‐sandwich complexes [{(WCA‐IDipp)E}MCl(*η* ^5^‐C_5_Me_5_)] (**5**–**8**). The direct reaction of the lithium salts **1** and **2** with a half or a full equivalent of [M(COD)Cl]_2_ (M=Rh, Ir) afforded the monometallic complexes [{(WCA‐IDipp)E}M(COD)] (**9**–**12**) or the bimetallic complexes [*μ*_2_‐{(WCA‐IDipp)E}M_2_(COD)_2_(*μ*_2_‐Cl)] (**13**–**16**), respectively. The bonding situation in these complexes has been investigated by means of density functional theory (DFT) calculations, revealing thiolate or selenolate ligand character with negligible metal‐chalcogen π‐interaction.

## Graphical abstract

**Taken under its wing**: Anionic N‐heterocyclic carbenes that bear a weakly coordinating fluoroborate moiety have been used to prepare a new class of anionic imidazolin‐2‐thione and ‐selone ligands; their coordination chemistry towards rhodium and iridium was explored to reveal, *inter alia*, their ability to develop secondary arene‐metal interactions with the “wings” of the carbene ligands.

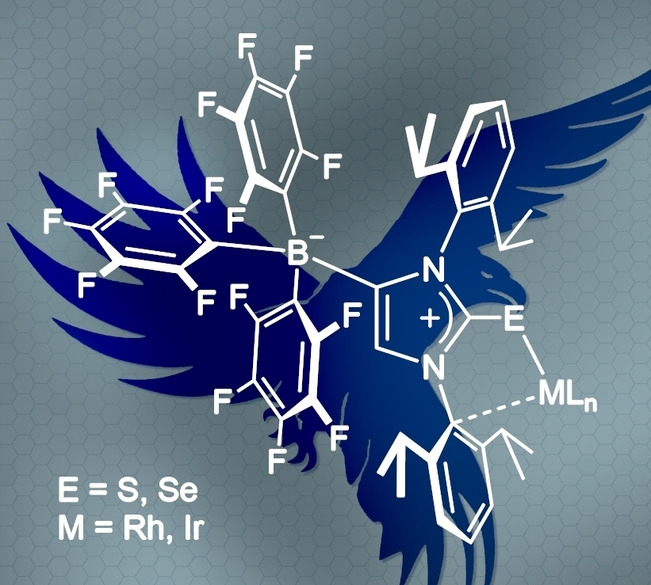

## Introduction

**Figure 1:**
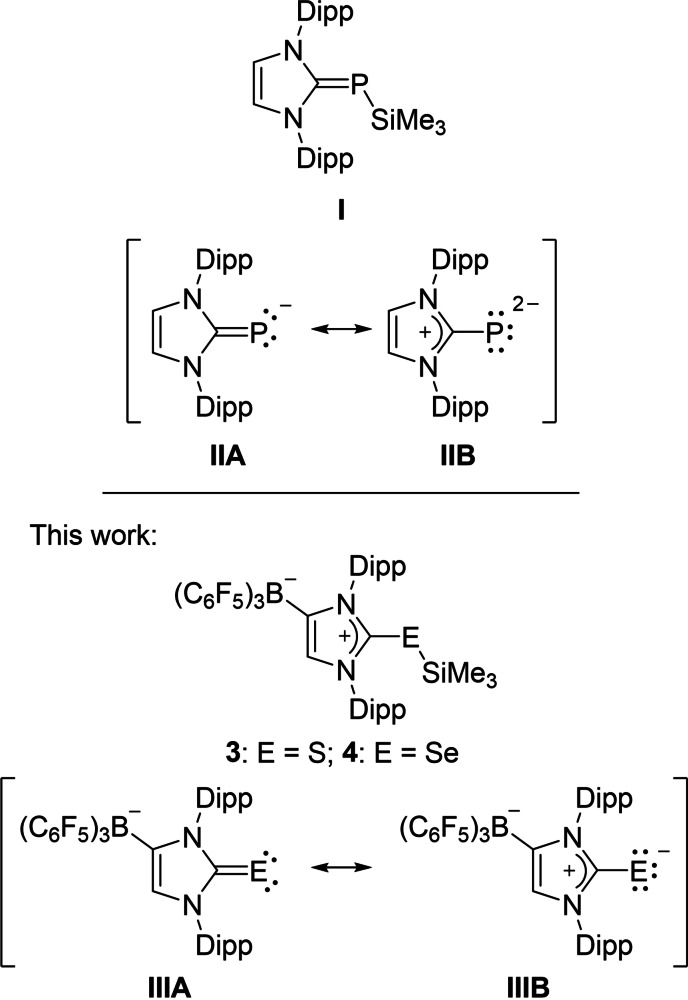
NHC‐phosphinidenide and NHC‐chalcogenide ligands.

Immediately after the isolation and structural characterization of the first stable and “bottleable” N‐heterocyclic carbene (NHC),^[1]^ the synthesis of NHC main group element adducts has become an area of extensive research.^[2]^ NHC‐phosphinidene species (NHC)PR have been among the earliest examples,^[3]^ and their versatile chemistry has again attracted great interest in recent years,^[4]^ not least because of their use as ligands in transition metal chemistry.^[5]^ In this context, the reaction of the trimethylsilyl‐substituted phosphinidene derivative (IDipp)PSiMe_3_ (**I**, IDipp=1,3‐bis(2,6‐diisopropylphenyl)imidazolin‐2‐ylidene, Figure 1) with transition metal halides proved to be particularly useful to enable the transfer of the monoanionic NHC‐phosphinidenide moiety **II** into the coordination sphere of transition metals via M−P bond formation accompanied by trimethylsilyl chloride elimination.^[6]^ Following this approach, half‐sandwich complexes such as [{(IDipp)P}MCl(*η* ^6^‐*p*‐cymene)] (M=Ru, Os) and [{(IDipp)P}MCl(*η* ^5^‐C_5_Me_5_)] (M=Rh, Ir) as well as related cationic species were prepared,[^6^, ^7^] which have structural and spectroscopic properties very similar to prototypic nucleophilic phosphinidene complexes of the type [RP=ML_n_], for example, low‐field ^31^P NMR resonances and short metal‐phosphorus double bonds.^[8]^ These characteristics can be ascribed to a strong polarization of the (IDipp)P^−^ ligand upon metal complexation as described by the mesomeric forms **IIA** and **IIB**, with the latter revealing its ability to act as a 2σ,2π‐electron donor. Compound **I** has also been used to synthesize NHC‐stabilized element‐phosphorus species (EP=PP, AsP, GeP, SnP),^[9]^ while several other phosphinidene and also arsinidene derivatives such as (IMes)PSiMe_3_, (IDipp)AsSiMe_3_ and (IMes)AsSiMe_3_ have recently become available for future application in transition metal and main group element chemistry (IMes=1,3‐bis(2,4,6‐trimethylphenyl)imidazolin‐2‐ylidene).^[10]^

Replacement of the pnictogen element in (NHC)P^−^ and (NHC)As^−^ systems by the neighboring chalcogen atoms affords neutral (valence) isoelectronic imidazolin‐2‐thiones and ‐selones (NHC)E (E=S, Se), which are well‐established and important ligands in transition metal chemistry,^[5]^ including the species (IDipp)S and (IDipp)Se analogous to **II**.^[11]^ (NHC)Se species have also found considerable interest, since their ^77^Se NMR chemical shifts are frequently used as an indicator for the π‐accepting properties of the corresponding NHCs.[^11d^, ^12^] Anionic N‐heterocyclic carbenes represent another important variation of classical NHC ligands,^[13]^ and our group introduced carbenes that contain a weakly coordinating anionic (WCA) fluoroborate moiety, for example, B(C_6_F_5_)_3_, at the C4 position of the imidazole ring.^[14]^ These carbenes were termed WCA‐NHCs and successfully introduced as ancillary ligands in transition metal chemistry and homogenous catalysis.[^14^, ^15^] More recently, these carbenes have also found application in main group element chemistry,[^9c^, ^16^] including the preparation of the complete series of lithium chalcogenides [{(WCA‐NHC)E}Li(solv.)] (E=O, S, Se, Te).^[17]^ Furthermore, the ^77^Se NMR spectra of the selenides [{(WCA‐NHC)Se}Li(toluene)] were studied to determine the π‐accepting properties of the WCA‐NHCs, revealing that the counter cation does not exert any significant influence, if it is fully separated from the selenium atom by solvation, for example, in THF‐*d*_8_.^[18]^

In this context, we envisaged that the lithium salts [{(WCA‐IDipp)E}Li(toluene)] (**1**: E=S; **2**: E=Se) or, similarly to **I**, the trimethylsilyl derivatives (WCA‐IDipp)ESiMe_3_ (**3**: E=S; **4**: E=Se) might serve as suitable reagents for the transfer of the anionic (WCA‐IDipp)E^−^ ligand (**III**, Figure 1). The resulting metal complexes of the type [{(WCA‐IDipp)E}ML_n_] (E=S, Se) will have an identical total charge as the corresponding NHC‐phosphinidenide complexes [{(IDipp)P}ML_n_] and therefore allow the comparison of the phosphorus‐metal and chalcogen‐metal bonds, while preserving the overall structural and electronic properties. Since previous studies with the ligand **II** included rhodium and iridium complexes,[^6^, ^7^] we aimed at the synthesis and investigation of related systems with the ligands **III**, which are reported in this contribution.

## Results and Discussion

### Synthesis and characterization of rhodium(III) and iridium(III) complexes

**Scheme 1:**
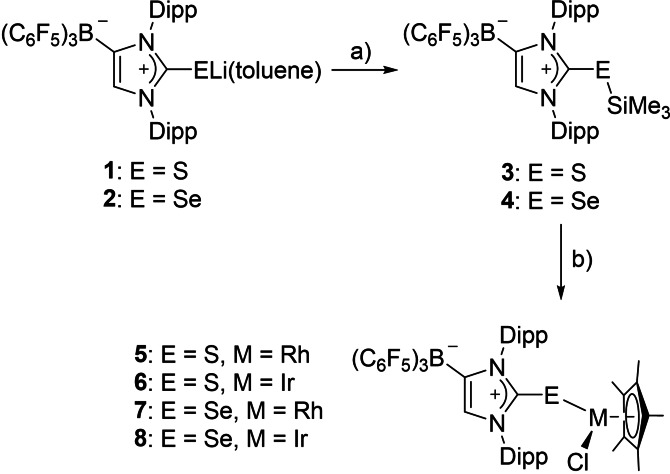
Preparation of trimethyl silyl sulfides and selenides and their subsequent use for the synthesis of Rh and Ir half‐sandwich complexes. a) Me_3_SiCl, toluene; b) 0.5 [(*η* ^5^‐C_5_Me_5_)MCl_2_]_2_, toluene (M=Rh, Ir).

The lithium salts **1** and **2** were prepared as toluene solvates following the published procedures.[^17^, ^18^] Their reactions with trimethylsilyl chloride (Me_3_SiCl) in toluene afforded the silanes (WCA‐IDipp)ESiMe_3_ (**3**: E=S; **4**: E=Se) as off‐white solids in 57 % and 34 % yield, respectively, after separation of LiCl by filtration and recrystallization from toluene/*n*‐hexane solution (Scheme 1). The ^1^H NMR spectra of **3** and **4** (in C_6_D_6_) indicate a loss of symmetry due to the presence of the B(C_6_F_5_)_3_ moiety in the backbone, and a splitting of the signals corresponding to the Dipp groups into two sets is observed. The characteristic signals for the hydrogen atom in the imidazole backbone are observed at 6.67 ppm (**3**) and 6.73 ppm (**4**). In the ^13^C NMR spectra, low‐field resonances at 141.7 and 136.6 ppm can be assigned to the CS and CSe carbon atoms, respectively. The ^11^B resonances for both compounds are found at ca. −15 ppm, and the ^19^F NMR spectra show three signals for the *ortho*‐, *meta*‐ and *para*‐fluorine atoms. For the selenium compound **4**, the ^77^Se NMR chemical shift is 79 ppm, which is at higher field compared to 114 ppm reported for [{(WCA‐IDipp)Se}Li(toluene)] (**2**)^[18]^ and 147 ppm reported for (WCA‐IDipp)SeH (both in THF‐*d*_8_);^[17]^ similar chemical shifts have been reported for trimethylsilyl aryl selenides such as 1,4‐C_6_H_4_(SeSiMe_3_)_2_ (78 ppm in C_6_D_6_).^[19]^ NMR data of all compounds are assembled in Table 1.

**Table 1:** NMR Chemical Shifts [ppm] of the complexes **1**–**16**.

	E	ML_n_	δ ^13^C (*C*E)	δ ^77^Se
**1^[a]^**	S	Li(thf)_4_	167.8	–
**2^[b]^**	Se	Li(tol.)	161.7	114
**3**	S	SiMe_3_	141.7	–
**4**	Se	SiMe_3_	136.6	79
**5**	S	RhCl(*η* ^5^‐C_5_Me_5_)	153.9	–
**6**	S	IrCl(*η* ^5^‐C_5_Me_5_)	155.0	–
**7**	Se	RhCl(*η* ^5^‐C_5_Me_5_)	149.2	117
**8**	Se	IrCl(*η* ^5^‐C_5_Me_5_)	146.7	122
**9**	S	Rh(COD)	163.0	–
**10**	S	Ir(COD)	169.0	–
**11**	Se	Rh(COD)	141.3	38
**12**	Se	Ir(COD)	140.8	53
**13**	S	Rh_2_(COD)_2_Cl	164.3	–
**14**	S	Ir_2_(COD)_2_Cl	169.0	–
**15**	Se	Rh_2_(COD)_2_Cl	141.3	39
**16**	Se	Ir_2_(COD)_2_Cl	140.2	50

[a] ref. [17]. [b] ref. [18].

**Figure 2:**
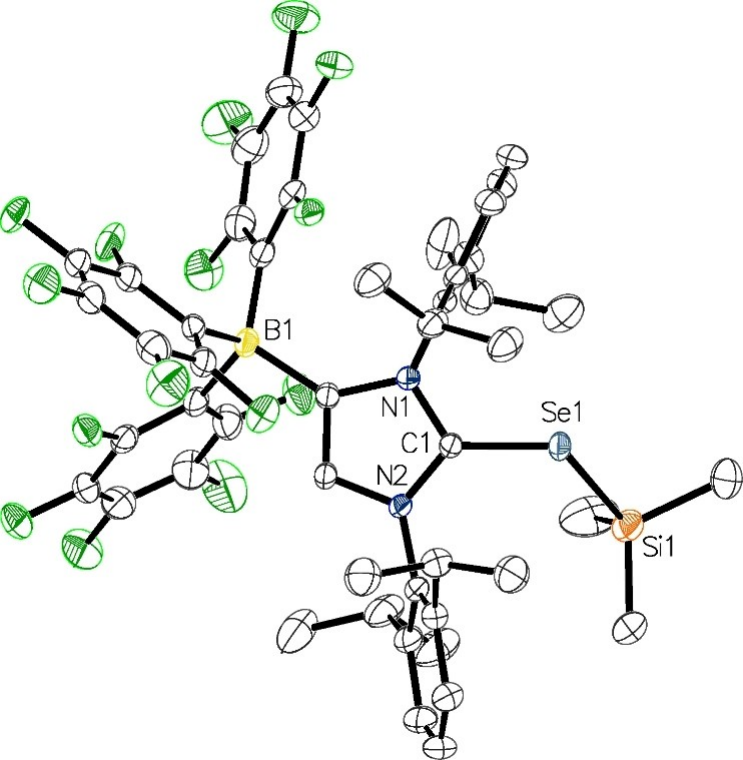
Molecular structure of **4** in **4** ⋅ *n*‐hexane with thermal displacement parameters drawn at 50 % probability; hydrogen atoms and solvent molecules are omitted for clarity; pertinent structural data for the compounds **1**–**16** are assembled in Table 2.

Single crystals were obtained from toluene/*n*‐hexane solutions of **3** and **4** at −30 °C and subjected to X‐ray diffraction analysis. The molecular structure of **4** is presented in Figure 2, whereas the molecular structure of **3** is shown in the Supporting Information (Figure S1). Pertinent structural data are summarized in Table 2. As expected, the C1−S1 bond length of 1.7358(11) Å as well as the C1−Se1 bond length of 1.890(2) Å are elongated, when compared to the distances found in the lithiated species **1** (1.685(2) Å) and **2** (1.845(2) Å).[^17^, ^18^] The N1−C1−N2 angles increase from 105.23(13)° in **1** to 107.22(9)° in **3** and from 106.03(17)° in **2** to 107.55(19)° in **4**, respectively, in agreement with an increase of imidazolium character. Overall, these structural parameters are similar to those established for the protonated species (WCA‐IDipp)EH (E=S, Se).^[17]^ The E−Si bond lengths are 2.2116(5) Å (E=S) and 2.3333(9) Å (E=Se), which is slightly longer compared to trimethylsilyl aryl sulfides^[20]^ and selenides,^[19]^ whereas the C1−E−Si angles of 107.97° (**3**) and 110.20° (**4**) correspond to the C1−P−Si and C1−As−Si angles of 110.71(4)°/106.40(4)° and 108.68(5)°/104.10(5)° established each for two independent molecules of the phosphorus and arsenic analogues (IDipp)E'SiMe_3_ (E’=P, As).[^6^, ^10b^]

**Table 2:** Selected Bond Lengths [Å] and Angles [°] of Compounds **1**–**16**.

Compound	E	ML_n_	E−C	M−E	M−C_*ipso*_	M−E−C1	E−M−Cl	N−C−N
**1** ^[a]^	S	Li(thf)_4_	1.685(2)	‐	–	–	–	105.23(13)
**2** ^[b]^	Se	Li(tol.)	1.845(2	2.543(5)	–	–	–	106.03(17)
**3**	S	SiMe_3_	1.7358(11)	2.2116(5)	‐	107.87(4)	–	107.22(9)
**4**	Se	SiMe_3_	1.890(2)	2.3333(9)	–	110.20(7)	–	107.55(19)
**5**	S	RhCl(*η* ^5^‐C_5_Me_5_)	1.730(3)	2.3001(7)	–	111.44(8)	95.00(3)	107.0(2)
**6**	S	IrCl(*η* ^5^‐C_5_Me_5_)	1.736(3)	2.2827(10)	–	111.00(11)	94.16(3)	107.0(3)
**7**	Se	RhCl(*η* ^5^‐C_5_Me_5_)	1.8815(15)	2.4014(5)	–	109.19(6)	97.990(18)	106.99(12)
**8**	Se	IrCl(*η* ^5^‐C_5_Me_5_)	1.897(2)	2.3970(4)	–	106.93(7)	94.47(2)	107.64(19)
**9**	S	Rh(COD)	1.702(4)	2.3073(9)	2.579(2)	104.11(10)	–	108.0(3)
**10** ^[c]^	S	Ir(COD)	1.703(3) 1.700(3)	2.3165(9) 2.3148(9)	2.381(3) 2.368(3)	102.48(10) 102.46(10)	–	107.8(2) 107.5(2)
**11**	Se	Rh(COD)	1.861(2)	2.4328(4)	2.587(2)	100.45(7)	–	108.08(18)
**12**	Se	Ir(COD)	1.8544(19)	2.4323(4)	2.3990(19)	98.66(6)	–	108.14(16)
**13**	S	Rh_2_(COD)_2_Cl	1.748(7)	2.4288(18) 2.4540(16)	–	115.8(3) 115.2(2)	86.39(6) 85.55(6)	107.4(6)
**14**	S	Ir_2_(COD)_2_Cl	1.760(4)	2.4104(10) 2.4240(11)	–	112.94(12) 112.86(13)	85.50(4) 85.18(3)	107.1(4)
**15**	Se	Rh_2_(COD)_2_Cl	1.907(5)	2.5163(7) 2.5439(7)	–	113.37(15) 113.10(15)	86.55(4) 87.30(4)	108.1(4)
**16**	Se	Ir_2_(COD)_2_Cl	1.913(6)	2.5214(6) 2.5225(6)	–	112.39(17) 112.84(17)	86.40(4) 86.58(5)	107.8(5)

[a] From ref. [17]. [b] From ref. [18]. [c] Two independent molecules in the asymmetric unit.

It is noteworthy that recrystallization of the selenium compound **4** from THF afforded crystals of the trimethylsilyl ether (WCA‐IDipp)Se(CH_2_)_4_OSiMe_3_, which must have formed by activation and insertion of ring‐opened THF into the Se−Si bond; see the Supporting Information for analytical and crystallographic data. This reactivity is reminiscent of frustrated trimethylsilyl‐NHC adducts^[21]^ and suggests an intrinsic lability of the chalcogen‐silicon bonds in **3** and **4**, which could be exploited for the transfer of the (WCA‐IDipp)E upon reaction with transition metal halides. To investigate this reactivity, **3** and **4** were each reacted with half an equivalent of the binuclear rhodium(III) and iridium(III) complexes [(*η* ^5^‐C_5_Me_5_)MCl_2_]_2_ (M=Rh, Ir) in toluene. After stirring for 15 min at room temperature, the solutions were evaporated, and the 16 valence electron complexes [{(WCA‐IDipp)E}MCl(*η* ^5^‐C_5_Me_5_)] (**5**–**8**) were isolated as dark brown (**5**/**7**) or dark orange (**6**/**8**) solids in moderate yields after washing with diethyl ether and extraction with THF. The ^77^Se NMR spectra for the selones **7** and **8** were recorded in THF‐*d*_8_ and reveal a high‐field shift of the selenium signal to 117 ppm (**7**) and 122 ppm (**8**), compared to 79 ppm in the silylated adduct **4**.

**Figure 3:**
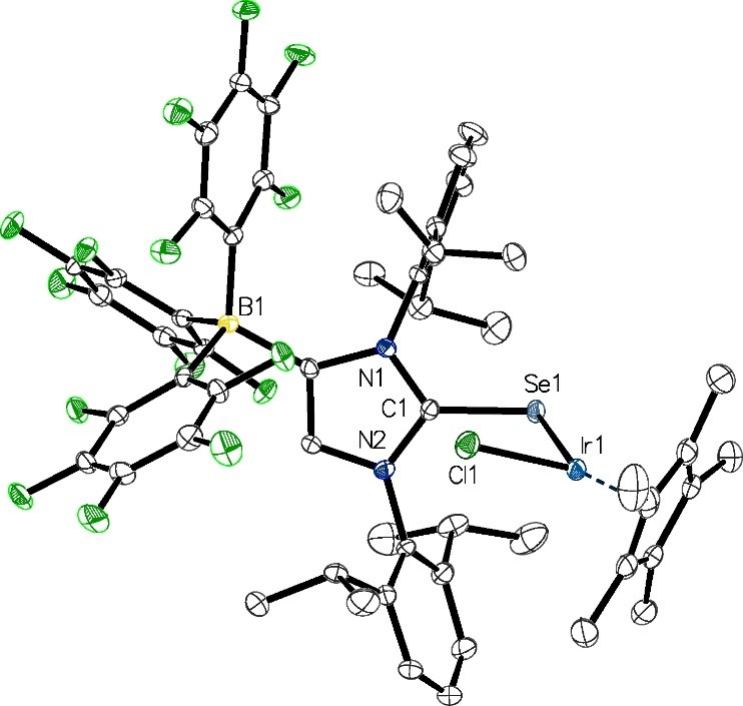
Molecular structure of **8** in **8** ⋅ CH_2_Cl_2_ with thermal displacement parameters drawn at 50 % probability; hydrogen atoms and solvent molecules are omitted for clarity; pertinent structural data for the compounds **1**–**16** are assembled in Table 2.

Single crystals suitable for X‐ray diffraction analysis were obtained by layering C_6_D_6_ solutions of **5** and **7** or CH_2_Cl_2_ solutions of **6** and **8** with *n*‐hexane. The molecular structure of **8** is shown in Figure 3, whereas presentations of **5**–**7** can be found in the Supporting Information (Figures S4–S6). Relevant structural data are compiled in Table 2. The metal atoms in complexes **5**–**8** all display a pseudo trigonal‐planar environment (two‐legged piano stool geometry) with small E−M−Cl angles of 94°–98° (E=S, Se; M=Rh, Ir). In all cases, the imidazole moiety adopts a *Z* orientation facing the chlorido ligand, and together with small M−E−C1 angles in the range 107°–111°, the overall structural features are very similar to those established for the analogous NHC‐phosphinidenide complexes [{(IDipp)P}MCl(*η* ^5^‐C_5_Me_5_)] (M=Rh, Ir).^[7a]^ However, the metal‐sulfur bond lengths of 2.3001(7) Å (**5**) and 2.2827(10) Å (**6**) are longer than the metal‐phosphorus bond lengths in the latter systems, revealing a considerably weaker metal‐ligand interaction (see below). Slightly shorter metal‐sulfur bonds of 2.2694(7) Å (Rh−S) and 2.2617(7) Å (Ir−S) were found in structurally closely related 2,6‐dimesitylbenzenethiolate (TerS) complexes [(TerS)MCl(*η* ^5^‐C_5_Me_5_)] (M=Rh, Ir),^[22]^ which also undergo interesting chloride abstraction and ligand addition reactions.^[23]^ As expected, the metal‐selenium bonds in **7** and **8** are longer than the metal‐sulfur bonds in **5** and **6**; with 2.4014(5) Å (**7**) and 2.3970(4) Å (**8**), however, they are significantly shorter than found in a few coordinatively saturated half‐sandwich rhodium and iridium complexes bearing chelating imidazolin‐2‐selone ligands.^[24]^

### Synthesis and characterization of rhodium(I) and iridium(I) complexes

**Scheme 2:**
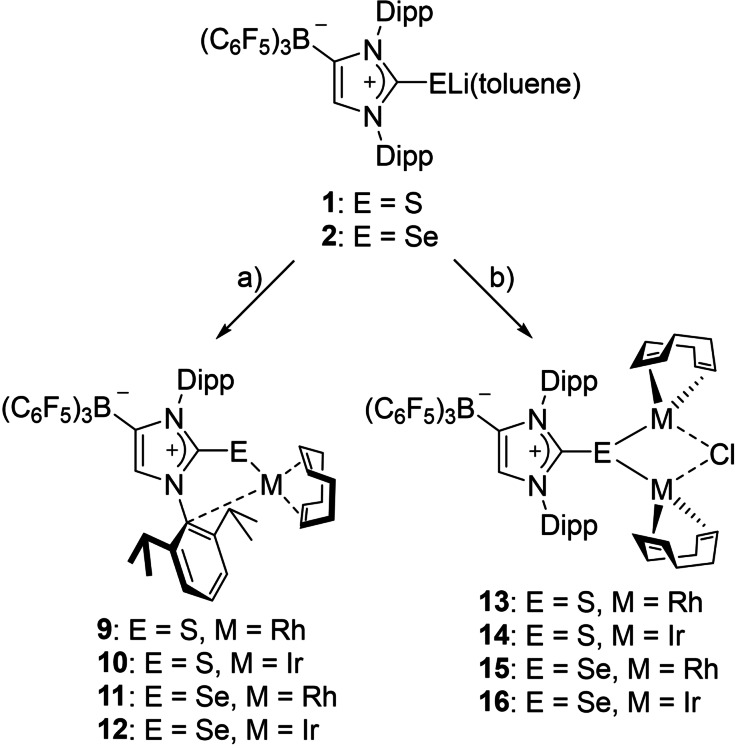
Preparation of rhodium and iridium complexes. a) 0.5 equiv. [M(COD)Cl]_2_, toluene; b) 1 equiv. [M(COD)Cl]_2_, toluene (M=Rh, Ir).

We have previously studied the reactions of the N‐heterocyclic carbene‐trimethylsilyl phosphinidene adduct (IDipp)PSiMe_3_ (**I**) with the binuclear rhodium(I) and iridium(I) 1,5‐cyclooctadiene (COD) complexes [M(COD)Cl]_2_ (M=Rh, Ir), which independently of the stoichiometry afforded a bimetallic rhodium complex [*μ*_2_‐{(IDipp)P}Rh_2_(COD)_2_(*μ*_2_‐Cl)].^[6]^ In contrast, no elimination of Me_3_SiCl was observed with iridium, but instead the monometallic complex [{(IDipp)PSiMe_3_}Ir(COD)Cl] was isolated.^[25]^ Therefore, the reactions of the silylated sulfur congener **3** with [M(COD)Cl]_2_ were initially studied by NMR spectroscopy, which, depending on the stoichiometry, resulted in a clean formation of the corresponding mono‐ and bimetallic complexes **9** and **13**. However, since the preparation of the silylated educts **3** and **4** proceeded via the lithium species **1** and **2** with a significant loss of yield, they were also directly employed for the synthesis of the corresponding rhodium and iridium COD complexes **9**–**12** and **13**–**16** (Scheme 2). Thus, the reactions of **1** or **2** with 0.5 equiv. of [M(COD)Cl]_2_ in toluene furnished the desired compounds [{(WCA‐IDipp)E}M(COD)] (**9**: E=S, M=Rh; **10**: E=S, M=Ir; **11**: E=Se, M=Rh; **12**: E=Se, M=Ir) as orange crystalline solids in satisfactory yields (45 %–74 %) after filtration and recrystallization from toluene/*n*‐hexane solution. The ^1^H and ^13^C NMR spectra of complexes **9**–**12** indicate the formation of *C*_s_‐symmetric complexes, e. g., by observation of four well separated doublets and two septets for the isopropyl methyl and methine hydrogen atoms, respectively. In addition, two different sets of CH and CH_2_ signals are found for the COD ligand. The ^77^Se NMR spectra (in THF‐*d*_8_) of the two selenides **11** and **12** exhibit signals at 38 ppm (**11**) and 53 ppm (**12**), respectively, indicating less deshielded selenium nuclei compared to the corresponding rhodium(III) and iridium(III) complexes **7** (117 ppm) and **8** (122 ppm, see above).

**Figure 4:**
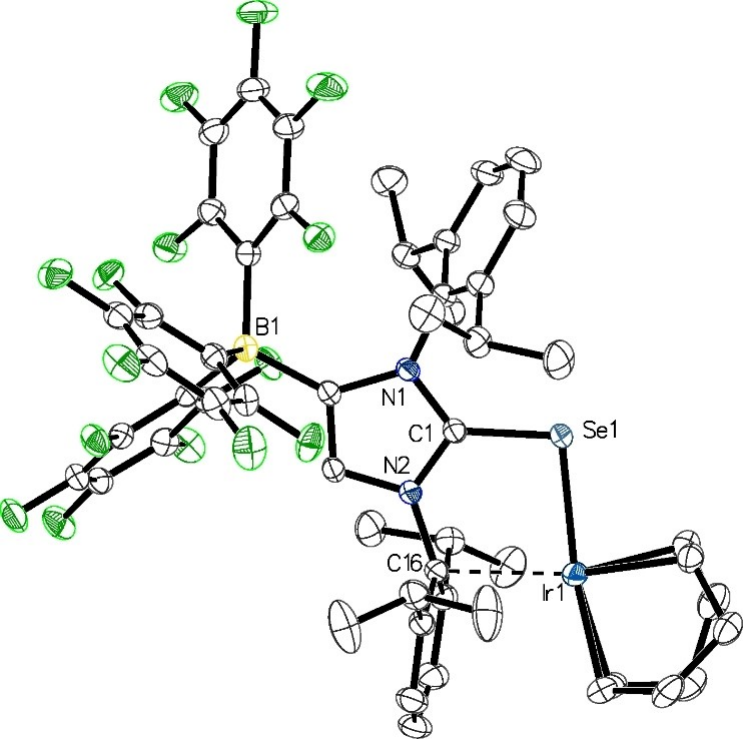
Molecular structure of **12** with thermal displacement parameters drawn at 50 % probability; hydrogen atoms are omitted for clarity; pertinent structural data for the compounds **1**–**16** are assembled in Table 2.

The molecular structures of **9**–**12** were determined by single‐crystal X‐ray diffraction analysis; see Figure 4 for **12** and the Supporting Information for **9**–**11** (Figures S8–S10). Pertinent structural parameters are assembled in Table 2. In all four complexes, the metal atoms reside in square‐planar environments. In addition to binding of the *η* ^4^‐COD ligand and the selenium atom, the coordination sphere is completed by an arene‐metal interaction (π‐face donation)^[26]^ that largely involves the *ipso*‐carbon atom of the Dipp substituent facing away from the borate moiety. Significantly longer, albeit distinctly different metal‐carbon distances are found for the adjacent *ortho*‐carbon atoms, which indicates a situation intermediate between *η* ^1^‐ and *η* ^2^‐coordination modes.^[27]^ Surprisingly, the Rh−C_*ipso*_ distances of 2.579(2) Å (**9**) and 2.587(2) Å (**11**) are significant longer than the Ir−C_*ipso*_ distances of 2.381(3) Å (**10**) and 2.3990(19) Å (**12**); in view of similar Rh and Ir atomic radii,^[28]^ this finding indicates a significantly stronger arene‐iridium interaction. It is also surprising that π‐face donation in complexes **9**–**12** does not involve the borate‐flanking Dipp substituent, which was consistently found in WCA‐NHC complexes, including related rhodium(I) and iridium(I) complexes of the type [(WCA‐NHC)M(COD)] (M=Rh, Ir).[^15a^, ^15b^, ^15g^, ^15h^] Theoretical calculations performed for the iridium complexes **10** and **12** (see details below, Table 3) indicate similar energies of the isomeric complexes in which the metal atom is bound to either one of the Dipp substituents, with a slight preference for the experimentally observed *anti*‐isomer versus the *syn*‐isomer (**10**: Δ*H*_298K_=−1.5 kcal mol^−1^; **12**: Δ*H*_298K_=−2.5 kcal mol^−1^). Moreover, the intramolecular π‐face donation in **9**–**12** enforces a coplanar orientation of the imidazole ring towards the M−E−C1 plane with significantly smaller M−E−C1 angles of 99°–104° compared to the perpendicular arrangement found in the rhodium(III) and iridium(III) half‐sandwich complexes **5**–**8**. Perpendicular orientations are also found in related Rh(I) and Ir(I) imidazolin‐2‐thione complexes such as neutral [{(^Me^IMe)S}Rh(COD)Cl],^[29]^ and cationic [{(^Me^I*i*Pr)S}_2_M(COD)]^+^ (M=Rh, Ir;^[30]^ for NHC acronyms see ref.^[5]^). These complexes exhibit longer metal‐sulfur bonds compared to 2.3073(2) Å in **9** and 2.3165(9)/2.3148(9) Å in **10** (for two independent molecules). As observed for the Rh/Ir pair **9**/**10**, the metal‐selenium bonds in **11**/**12** (2.4328(4)/2.4323(4) Å) are almost identical; to the best of our knowledge, however, no related imidazolin‐2‐selone complexes have been reported for comparison.

**Table 3:** Relative enthalpies (ΔH_298K_), Natural Bond Orbital (NBO) charges (q), Wiberg Bond Indices (WBI) and selected bond lengths of **III**, **6**, **8**, **10** and **12**.

Compound	*ΔH*_298K_ [kcal/mol]	*q*(E)	*q*(Ir)	WBI(E−C_NHC_)	WBI(Ir−E)	*d*(E−C_NHC_)	*d*(Ir−E) [Å]	*d*(Ir−C_*ipso*_) [Å]
**III** (E=S)	–	−0.31	–	1.44	–	1.688	–	–
**III** (E=Se)	–	−0.29	–	1.30	–	1.869	–	–
**6** (E=S)	–	0.09	−0.06	1.13	0.88	1.740	2.311	–
**8** (E=Se)	–	0.20	−0.10	1.04	0.90	1.915	2.453	–
**10** (E=S)	−1.5^[a]^	0.08	−0.08	1.19	0.75	1.726	2.383	2.368
**12** (E=Se)	−2.5^[a]^	0.19	−0.13	1.10	0.79	1.892	2.531	2.358

[a] *ΔH*_298K_=*H*_298K_(*anti*)−*H*_298K_(*syn*)

The stoichiometry of [M(COD)Cl]_2_ in the reaction with the lithium salts **1** or **2** can be increased to 1 equiv. to furnish the bimetallic complexes [*μ*_2_‐{(WCA‐IDipp)E}M_2_(COD)_2_(*μ*_2_‐Cl)] (**13**: E=S, M=Rh; **14**: E=S, M=Ir; **15** E=Se, M=Rh; **16**: E=Se, M=Ir); Scheme 2). The reactions were performed in toluene, and after separation of LiCl and recrystallization, the rhodium complexes **13** and **15** were obtained as dark orange crystals in 83 % and 33 % yield and the iridium complexes **14** and **16** as dark purple crystals in 64 % and 37 % yield. The recrystallization of **13** and **15** was performed from a saturated toluene solution layered with *n*‐hexane, whereas for the recrystallization of **14** and **16**, a solution in THF was layered with *n*‐hexane. The ^1^H and ^13^C NMR spectra of **13**–**16** exhibit the same number of signals for the WCA‐NHC unit as the monometallic complexes **9**–**12**, revealing time‐averaged *C*_s_ symmetry in solution through fast rotation around the E−C bonds (E=S, Se). Because of the bimetallic nature, however, the number of COD signals doubles compared to **9**–**12**. The ^77^Se NMR chemical shifts for the rhodium and iridium selenides **15** and **16** are recorded at 39 ppm and 50 ppm, respectively, which is almost identical to the values established for the monometallic complexes **11** and **12**.

**Figure 5:**
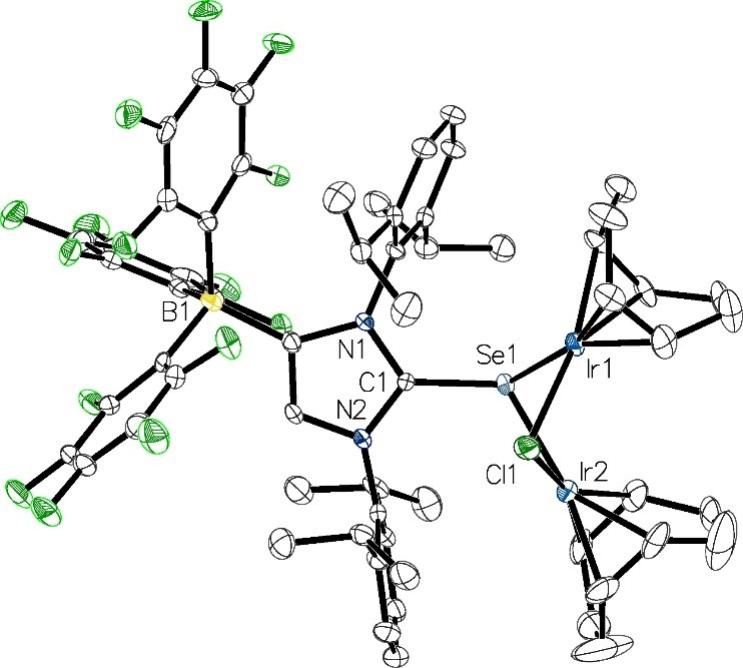
Molecular structure of **16** in **16** ⋅ 0.5 THF⋅0.5 HMDSO with thermal displacement parameters drawn at 50 % probability; hydrogen atoms and solvent molecules are omitted for clarity; pertinent structural data for the compounds **1**–**16** are assembled in Table 2. Selected bond angles [°]: Ir1−Cl1−Ir2 75.49(5), Ir1−Se1−Ir2 71.07(2).

The molecular structures of all four bimetallic complexes could be established by single‐crystal X‐ray diffraction analysis; see Figure 5 for the representation of **16** and Figures S12–S14 in the Supporting Information for the molecular structures of **13**–**15**. Overall, the structures resemble that of the bridged NHC‐phosphinidenide complex [*μ*_2_‐{(IDipp)P}Rh_2_(COD)_2_(*μ*_2_‐Cl)],^[6]^ with the bridging chalcogen atoms residing in trigonal‐pyramidal environments. *Z* configurations can be assigned to the carbene moieties, which are facing the chlorine atoms, while the imidazole planes adopt approximately horizontal orientations with respect to the metal‐metal axes; this renders the complexes *C*_1_ symmetric in the solid state. The four‐membered M1−E−Cl−M2 rings are puckered and display butterfly‐like conformations. As expected, the metal‐chalcogen bonds in the bimetallic complexes **13**–**15** are significantly elongated compared to the monometallic congeners **9**–**12**, with a different degree of asymmetry in the M1−E and M2−E bond lengths. Likewise, a pronounced increase of the E−C bond lengths can be observed (Table 2).

### Computational studies

In order to assess the bonding situation in the thione and selone complexes described above, the structures of the iridium derivatives **6**, **8**, **10**, and **12** were optimized using the density functional theory (DFT) method B97‐D, followed by natural bond orbital (NBO) analysis. The computational details are summarized in the Supporting Information, and contour plots of selected NBOs are presented in Tables S16–S19. The calculated structural parameters are in good agreement with the solid‐state structures, with the optimized gas‐phase geometries consistently showing slightly longer bond lengths (Tables 2 and 3). The Wiberg bond index (WBI) associated with the iridium‐chalcogen bonds in **6** (0.88) and **8** (0.90) clearly reveal the presence of single bonds in contrast to the situation established for the related NHC‐phosphinidenide complex [{(IDipp)P}IrCl(*η* ^5^‐C_5_Me_5_)] (1.32)^[7a]^ and related cationic systems (1.35–1.40)^[7b]^ with a significant degree of phosphorus‐metal π‐interaction. The negligible degree of chalcogen‐metal π‐bonding results in significantly smaller charge transfer towards the metal atom as indicated by comparatively small NBO charges *q*(Ir). Accordingly, two lone pairs can be assigned each to the sulfur and selenium atoms, which show the expected pure p or high s orbital character, respectively. This bonding situation can be best described by polarization of the (WCA‐IDipp)E^−^ ligands **III** (E=S, Se) upon metal coordination as illustrated by the mesomeric form **IIIB** in Figure 1, in line with WBI(E−C_NHC_) values close to 1.

For complexes **10** and **12**, the geometries of both isomers with *syn‐* or *anti*‐orientations of the borate moiety with respect to the iridium atom were optimized, and as mentioned above, the experimentally observed *anti*‐isomer is energetically slightly favored (Table 3). Somewhat weaker iridium‐chalcogen bonds can be assigned to these iridium(I) complexes based on the WBI values of 0.75 (**10**) and 0.79 (**12**) compared to the iridium(III) species **6** and **8**. Again, two lone pairs are located at the chalcogen atoms with high s and pure p orbital character; the latter is perpendicular to the imidazole ring, which is coplanar with the Ir−E−C1 plane to enable π‐face donation through the *ipso‐*carbon atom. This bonding scheme agrees with the NBO analyses of the anionic thione and selone ligands **III** (Figure 1), which reveal three lone pairs located at the chalcogen atoms, one with high s orbital character, while the other two pure p orbitals have similar energies and are available for coordination to the metal atom in either **6**/**8** or **10**/**12** (see the Supporting Information).

## Conclusion

Half‐sandwich complexes of the type [{(WCA‐IDipp)E}MCl(*η* ^5^‐C_5_Me_5_)] (**5**–**8**) have been synthesized, which are structurally closely related to NHC‐phosphinidenide complexes [{(IDipp)P}MCl(*η* ^5^‐C_5_Me_5_)] (M=Rh, Ir). Furthermore, we have demonstrated the ability of these chalcogen ligands to form rhodium(I) and iridium(I) cyclooctadiene complexes [{(WCA‐IDipp)E}M(COD)] (**9**–**12**) or complexes [*μ*_2_‐{(WCA‐IDipp)E}M_2_(COD)_2_(*μ*_2_‐Cl)] (**13**–**16**), depending on the stoichiometry of the starting materials. Interestingly, the monometallic COD species **9**–**12** display a π‐face donation of one Dipp substituent towards the Rh or Ir atoms in order to saturate their coordination sphere. The bonding schemes of the ligands **III** were further assessed by computational studies, revealing in all cases distinct metal‐chalcogen single bonds and two lone pairs located at the chalcogen atoms. This contrasts with the related NHC‐phosphinidenide complexes, where substantial metal‐phosphorus π bond character was found.

With the lithium complexes **1**/**2** and the silyl derivatives **3**/**4**, suitable reagents are available for the transfer of the anionic sulfur and selenium ligands **III**, which we regard as important new additions to the large family of chalcogenolate ligands.^[31]^ The coordination chemistry of these systems with main group elements and transition metals has been extensively studied,^[32]^ with considerable implications for the preparation of complexes that mimic the active sites in metalloproteins.^[33]^ We envisage that the anionic charge will significantly expand the scope of N‐heterocyclic thione and selone ligands in coordination chemistry and homogeneous catalysis,^[5]^ which follows the same concept developed for neutral NHCs with the introduction of their anionic WCA‐NHC congeners.[^14^, ^15a^]

## Experimental Section

**Materials and Method**s: Unless otherwise indicated, all starting materials were obtained from commercial sources (Sigma‐Aldrich, Alfa‐Aesar, Roth, TCI, VWR or Fisher Chemical) and were used without further purification. Elemental analyses were carried out on a Vario Micro Cube System. All operations with air‐ and moisture‐sensitive compounds were performed in a glove box under a dry argon atmosphere (MBraun 200B) or on a high vacuum line using Schlenk techniques. The ^1^H, ^11^B, ^13^C, ^19^F and ^77^ Se NMR spectra were recorded on Bruker AVII300 (300 MHz), Bruker AVIIHD400 (400 MHz), Bruker AVIIHD500 (500 MHz) and Bruker AVII600 (600 MHz). All spectra were recorded at 298 K. The chemical shifts are expressed in parts per million (ppm) with the residual solvent signal as internal standard for ^1^H and ^13^C NMR spectra. All other spectra were calibrated using external references (^11^B: BF_3_ ⋅ OEt_2_; ^19^F: CFCl_3_; ^77^Se: Me_2_Se). Coupling constants (*J*) are reported in Hertz (Hz) and splitting patterns are indicated as s (singlet), d (doublet), t (triplet), sept (septet), m (multiplet) and br (broad). ^11^B, ^13^C, ^19^F and ^77^Se NMR spectra were measured broadband proton decoupled. Signal assignments were performed based on 2D NMR analysis. Presentations of all NMR spectra can be found in the Supporting Information. *n‐*hexane, tetrahydrofuran (THF), diethyl ether (Et_2_O), and toluene were purified by distillation over sodium/benzophenone. Chlorobenzene was purified by distillation over CaH_2_. Deuterated solvents were purified by stirring the degassed solvents with Na/K alloy overnight. Subsequently, the solvents were filtered and then distilled under reduced pressure. All solvents were stored over molecular sieves (4 Å) in argon atmosphere prior to use. All yields were calculated based on the WCA‐IDipp containing substrate. [Rh(COD)Cl]_2_,^[34]^ [Ir(COD)Cl]_2_,^[35]^ [(*η* ^5^‐C_5_Me_5_)RhCl_2_]_2_,^[36]^ [(*η* ^5^‐C_5_Me_5_)IrCl_2_]_2_,^[37]^ [{(WCA‐IDipp)S}Li(toluene)]^[17]^ and [{(WCA‐IDipp)Se}Li(toluene)]^[18]^ were prepared according to literature procedures.

For full crystallographic details, see the Supporting Information. Deposition Number(s) 2122410‐2122424 contain(s) the supplementary crystallographic data for this paper. These data are provided free of charge by the joint Cambridge Crystallographic Data Centre and Fachinformationszentrum Karlsruhe Access Structures service www.ccdc.cam.ac.uk/structures.

**Synthesis of (WCA‐IDipp)SSiMe_3_ (3)**: [(WCA‐IDipp)S]Li ⋅ (toluene) (250 mg, 0.243 mmol, 1 equiv.) was suspended in 5 mL toluene and Me_3_SiCl (40 mg, 0.364 mmol, 1.5 equiv.) diluted in 2 mL toluene was added dropwise, resulting in a pale‐yellow and slightly cloudy solution. The mixture was stirred for 3 h at room temperature and subsequently filtered, giving a clear pale‐yellow solution. The solvent was evaporated, and the yellow residue was washed with 3×2 mL *n*‐hexane. The yellow washings were discarded and residual solvent in the solid was removed in *vacuo*, yielding the product as an off‐white solid (138 mg, 0.137 mmol, 57 %). ^**1**^ **H NMR** (600 MHz, C_6_D_6_) *δ*=7.01 (t, ^3^ *J*(^1^H,^1^H)=7.8 Hz, 2H, *p*‐Dipp), 6.85 (d, ^3^ *J*(^1^H,^1^H)=7.8 Hz, 4H, *m*‐Dipp), 6.67 (s, 1H, HC=CB), 2.73–2.69 (m, 2H, C *H* (CH_3_)_2_), 2.64–2.59 (m, 2H, C *H* (CH_3_)_2_), 1.13–1.07 (m, ^3^ *J*(^1^H,^1^H)=6.7 Hz, 18H, CH(C *H*_3_)_2_), 0.87–0.83 (m, ^3^ *J*(^1^H,^1^H)=4.8 Hz, 6H, CH(C *H*_3_)_2_), −0.46 (s, 9H, Si(CH_3_)_3_). ^**11**^ **B NMR** (96 MHz, C_6_D_6_) *δ*=−15.18 (s). ^**13**^ **C NMR** (151 MHz, C_6_D_6_) *δ*=150.50–150.10 (m, Ar^F^), 148.96–148.35 (m, Ar^F^), 146.92 (s, *o*‐Dipp), 146.01 (s, *o*‐Dipp), 141.66 (s, N−C−N), 140.75–140.34 (m, Ar^F^), 139.00–138.72 (m, Ar^F^), 138.44–138.05 (m, Ar^F^), 136.85–136.42 (m, Ar^F^), 133.01 (s, H *C* =CB), 132.31 (s, *ipso*‐Dipp), 132.09 (s, *ipso*‐Dipp), 131.89 (s, *p*‐Dipp), 131.48 (s, *p*‐Dipp), 125.71 (s, *m*‐Dipp), 124.53 (s, *m*‐Dipp), 31.95 (s, *C* H(CH_3_)_2_), 28.92 (s, CH(*C* H_3_)_2_), 28.68 (s, *C* H(CH_3_)_2_), 27.16 (s, CH(*C* H_3_)_2_), 25.94 (s, CH(*C* H_3_)_2_), 22.75 (s, CH(*C* H_3_)_2_), 0.80 (s, Si(CH_3_)_3_).A signal corresponding to HC= *C* B was not observed. ^**19**^ **F NMR** (283 MHz, C_6_D_6_) *δ*=−127.82–−132.17 (m), −159.54 (t, ^3^ *J*(^19^F,^19^F)=20.4 Hz), −163.89–−167.17 (m). **EA** – Anal. Calc. for C_48_H_44_BF_15_N_2_OSSi ⋅ C_4_H_8_O: C, 58.00; H, 4.87; N, 2.60. Found: C, 58.18; H, 4.54; N, 2.65.

**Synthesis of (WCA‐IDipp)SeSiMe_3_ (4)**: [(WCA‐IDipp)Se]Li ⋅ (toluene) (500 mg, 0.464 mmol, 1 equiv.) was suspended in 10 mL toluene and Me_3_SiCl (50 mg, 0.464 mmol, 1 equiv.) diluted in 2 mL toluene was added dropwise, resulting in a dark orange cloudy solution. The mixture was stirred for 3 d at room temperature and subsequently filtered, giving a clear dark orange solution. The solvent was evaporated, and the brown residue was recrystallized from toluene layered with *n*‐hexane. The resulting light brown crystals were collected by decantation of the supernatant solution. Residual solvent was removed in *vacuo*, yielding the product as a light brown solid (166 mg, 0.158 mmol, 34 %). ^**1**^ **H NMR** (500 MHz, C_6_D_6_) *δ*=7.19 (t, ^3^ *J*(^1^H,^1^H)=7.8 Hz, 1H, *p*‐Dipp), 7.03 (t, ^3^ *J*(^1^H,^1^H)=7.8 Hz, 1H, *p*‐Dipp), 6.87 (d, ^3^ *J*(^1^H,^1^H)=7.8 Hz, 4H, *m*‐Dipp), 6.73 (s, 1H, HC=CB), 2.79–2.72 (m, 2H, C *H* (CH_3_)_2_), 2.67–2.54 (m, 2H, C *H* (CH_3_)_2_), 1.17–1.04 (m, 18H, CH(C *H*_3_)_2_), 0.91–0.87 (m, ^3^ *J*(^1^H,^1^H)=6.8 Hz, 6H, CH(C *H*_3_)_2_), −0.40 (s, 9H, Si(CH_3_)_3_). ^**11**^ **B NMR** (161 MHz, C_6_D_6_) *δ*=−15.09 (s). ^**13**^ **C NMR** (126 MHz, C_6_D_6_) *δ*=155.89 (d, ^1^ *J*(^11^B,^13^C)=53 Hz, HC= *C* B), 150.74–149.97 (m, Ar^F^), 148.90–148.17 (m, Ar^F^), 146.85 (s, *o*‐Dipp), 145.96 (s, *o*‐Dipp, *o*‐Dipp), 140.89–140.33 (m, Ar^F^), 139.07–138.58 (m, Ar^F^), 138.58–138.23 (m, Ar^F^), 136.63 (s, N−C−N), 136.55–136.06 (m, Ar^F^), 133.95 (s, H *C* =CB), 133.41 (s, *ipso*‐Dipp), 132.81 (s, *ipso*‐Dipp), 131.87 (s, *p*‐Dipp), 131.48 (s, *p*‐Dipp), 125.61 (s, *m*‐Dipp), 124.66 (s, *m*‐Dipp), 31.95 (s, *C* H(CH_3_)_2_), 28.98 (s, CH(*C* H_3_)_2_), 28.63 (s, *C* H(CH_3_)_2_), 26.91 (s, CH(*C* H_3_)_2_), 25.92 (s, CH(*C* H_3_)_2_), 22.68 (s, CH(*C* H_3_)_2_), 1.19 (s, Si(CH_3_)_3_). ^**19**^ **F NMR** (377 MHz, C_6_D_6_) *δ*=−129.58–−134.88 (m), −159.54 (br s), −163.84–−167.42 (m). ^**77**^ **Se NMR** (95 MHz, C_6_D_6_) *δ*=79.32 (s). **EA** – Anal. Calc. for C_48_H_44_BF_15_N_2_SeSi ⋅ 0.5(C_6_H_14_): C, 55.95; H, 4.70; N, 2.56. Found: C, 55.66; H, 3.89; N, 2.57.

**Synthesis of [{(WCA‐IDipp)S}RhCl(*η* ^5^‐C_5_Me_5_)] (5)**: (WCA‐IDipp)SSiMe_3_ (20 mg, 0.020 mmol, 1 equiv.) and [(*η* ^5^‐C_5_Me_5_)RhCl_2_]_2_ (6.15 mg, 0.010 mmol, 0.5 equiv.) were mixed and dissolved in 2 mL toluene. The mixture turns dark brown within 2 min and was stirred for a total of 15 min at room temperature. The solvent was removed in *vacuo* and the resulting dark brown solid was washed with 2×1 mL Et_2_O and subsequently extracted with 0.2 mL THF, giving a dark brown clear solution. The solvent was removed in *vacuo* and the residual THF was removed by co‐evaporation with *n*‐hexane, yielding the product as an orange‐brown solid (14 mg, 0.0116 mmol, 58 %). ^**1**^ **H NMR** (500 MHz, THF‐*d*_8_) *δ*=7.30 (t, ^3^ *J*(^1^H,^1^H)=7.7 Hz, 1H, *p*‐Dipp), 7.22–7.16 (m, 3H, *p*‐Dipp & *m*‐Dipp), 6.91 (d, ^3^ *J*(^1^H,^1^H)=7.5 Hz, 2H, *m*‐Dipp), 6.61 (s, 1H, HC=CB), 2.79–2.68 (m, 4H, 2×C *H* (CH_3_)_2_), 1.31 (d, ^3^ *J*(^1^H,^1^H)=6.4 Hz, 6H), 1.25 (s, 15H, Cp*), 0.96 (d, ^3^ *J*(^1^H,^1^H)=6.0 Hz, 6H, CH(C *H*_3_)_2_), 0.82 (d, ^3^ *J*(^1^H,^1^H)=5.9 Hz, 6H, CH(C *H*_3_)_2_), 0.72 (d, ^3^ *J*(^1^H,^1^H)=6.4 Hz, 6H, CH(C *H*_3_)_2_). ^**11**^ **B NMR** (161 MHz, THF‐*d*_8_) *δ*=−15.19 (s). ^**13**^ **C NMR** (126 MHz, THF‐*d*_8_) *δ*=153.93 (s, N−C−N), 150.92–150.32 (m, Ar^F^), 148.87 (s, *o*‐Dipp), 148.85–148.48 (m, Ar^F^), 147.32 (s, *o*‐Dipp), 140.92–140.44 (m, Ar^F^), 138.93–138.10 (m, 2×Ar^F^), 136.75–136.12 (m, Ar^F^), 134.28 (s, *ipso*‐Dipp), 134.03 (s, *ipso*‐Dipp), 132.69 (s, H *C* =CB), 130.99 (s, *p*‐Dipp), 130.64 (s, *p*‐Dipp), 125.26 (s, *m*‐Dipp), 124.34 (s, *m*‐Dipp), 98.54 (d, ^1^ *J*(^13^C,^103^Rh)=8.1 Hz, C=C(Cp*)), 29.69 (s, *C* H(CH_3_)_2_), 28.76 (s, *C* H(CH_3_)_2_), 28.69 (s, CH(*C* H_3_)_2_), 26.61 (s, CH(*C* H_3_)_2_), 23.40 (s, CH(*C* H_3_)_2_), 22.90 (s, CH(*C* H_3_)_2_), 9.26 (s, Me−Cp*). A signal corresponding to HC= *C* B was not observed. ^**19**^ **F NMR** (471 MHz, THF‐*d*_8_) *δ*=−162.42 (s), −167.16 (s). One CF signal was too broad and could not be resolved. **EA** – Anal. Calc. for C_55_H_50_BClF_15_N_2_RhS: C, 54.81; H, 4.18; N, 2.32. Found: C, 54.88; H, 4.36; N, 2.22.

**Synthesis of [{(WCA‐IDipp)S}IrCl(*η* ^5^‐C_5_Me_5_)] (6)**: (WCA‐IDipp)SSiMe_3_ (20 mg, 0.020 mmol, 1 equiv.) and [(*η* ^5^‐C_5_Me_5_)IrCl_2_]_2_ (7.93 mg, 0.010 mmol, 0.5 equiv.) were mixed and dissolved in 2 mL toluene. The mixture turns dark brown within 2 min and was stirred for a total of 15 min at room temperature. The solvent was removed in *vacuo* and the resulting dark orange‐brown solid was washed with 2×1 mL Et_2_O and subsequently extracted with 0.2 mL THF, giving a dark brown clear solution. The solvent was removed in *vacuo* and the residual THF was removed co‐evaporation with *n*‐hexane, yielding the product as a dark orange solid (17 mg, 0.0131 mmol, 66 %). ^**1**^ **H NMR** (500 MHz, THF‐*d*_8_) *δ*=7.33–7.29 (m, 1H, *p*‐Dipp), 7.22–7.18 (m, 3H, *p*‐Dipp & *m*‐Dipp), 6.93 (d, ^3^ *J*(^1^H,^1^H)=7.8 Hz, 2H, *m*‐Dipp), 6.69 (s, 1H, HC=CB), 2.78–2.68 (m, 4H, 2×C *H* (CH_3_)_2_), 1.29 (d, ^3^ *J*(^1^H,^1^H)=6.6 Hz, 6H, CH(C *H*_3_)_2_), 1.25 (s, 15H, Cp*), 0.95 (d, ^3^ *J*(^1^H,^1^H)=6.5 Hz, 6H, CH(C *H*_3_)_2_), 0.83 (d, ^3^ *J*(^1^H,^1^H)=6.3 Hz, 6H, CH(C *H*_3_)_2_), 0.73 (d, ^3^ *J*(^1^H,^1^H)=6.7 Hz, 6H, CH(C *H*_3_)_2_). ^**11**^ **B NMR** (161 MHz, THF‐*d*_8_) *δ*=−15.11 (s). ^**13**^ **C NMR** (126 MHz, THF‐*d*_8_) *δ*=155.04 (s, N−C−N), 150.88–150.33 (m, Ar^F^), 148.81 (s, *o*‐Dipp), 148.82–148.44 (m, Ar^F^), 147.09 (s, *o*‐Dipp), 141.19–140.43 (m, Ar^F^), 138.80–138.29 (m, 2×Ar^F^), 136.87–136.15 (m, Ar^F^), 134.07 (s, *ipso*‐Dipp), 133.82 (s, *ipso*‐Dipp), 132.98 (s, H *C* =CB), 131.06 (s, *p*‐Dipp), 130.82 (s, *p*‐Dipp), 125.30 (s, *m*‐Dipp), 124.49 (s, *m*‐Dipp), 91.78 (s, C=C(Cp*)), 29.82 (s, *C* H(CH_3_)_2_), 28.74 (s, *C* H(CH_3_)_2_), 28.61 (s, CH(*C* H_3_)_2_), 26.58 (s, CH(*C* H_3_)_2_), 23.27 (s, CH(*C* H_3_)_2_), 22.93 (s, CH(*C* H_3_)_2_), 9.45 (s, Me−Cp*). A signal corresponding to HC= *C* B was not observed. ^**19**^ **F NMR** (471 MHz, THF‐*d*_8_) *δ*=−163.22 (s), −168.05 (s). One CF signal was too broad and could not be resolved. **EA** – Anal. Calc. for C_55_H_50_BClF_15_N_2_IrS ⋅ 0.5(C_7_H_8_): C, 52.41; H, 4.06; N, 2.09. Found: C, 52.65; H, 4.44; N, 2.02.

**Synthesis of [{(WCA‐IDipp)Se}RhCl(*η* ^5^‐C_5_Me_5_)] (7)**: (WCA‐IDipp)SeSiMe_3_ (20 mg, 0.0199 mmol, 1 equiv.) and [(*η* ^5^‐C_5_Me_5_)RhCl_2_]_2_ (5.9 mg, 0.0095 mmol, 0.5 equiv.) were mixed and dissolved in 2 mL toluene. The mixture turns dark brown within 2 min and was stirred for a total of 15 min at room temperature. The solvent was removed in *vacuo* and the resulting dark brown solid was washed with 2×1 mL Et_2_O and subsequently extracted with 0.2 mL THF, giving a dark brown clear solution. The solvent was removed in *vacuo* and the residual THF was removed co‐evaporation with *n*‐hexane, yielding the product as a dark brown solid (9 mg, 0.0072 mmol, 36 %). ^**1**^ **H NMR** (500 MHz, THF‐*d*_8_) *δ*=7.15–7.11 (m, 1H, *p*‐Dipp), 7.06 (t, ^3^ *J*(^1^H,^1^H)=7.7 Hz, 1H, *p*‐Dipp), 7.01 (d, ^3^ *J*(^1^H,^1^H)=7.7 Hz, 2H, *m*‐Dipp), 6.81 (d, ^3^ *J*(^1^H,^1^H)=7.7 Hz, 2H, *m*‐Dipp), 6.05 (s, 1H, HC=CB), 2.92–2.83 (m, 2H, C *H* (CH_3_)_2_), 2.74–2.67 (m, 2H, C *H* (CH_3_)_2_), 1.53 (s, 15H, Cp*), 1.22 (d, ^3^ *J*(^1^H,^1^H)=7.9 Hz, 6H, CH(C *H*_3_)_2_), 1.14 (d, ^3^ *J*(^1^H,^1^H)=6.8 Hz, 6H, CH(C *H*_3_)_2_), 0.94 (d, ^3^ *J*(^1^H,^1^H)=6.9 Hz, 6H, CH(C *H*_3_)_2_), 0.74 (d, ^3^ *J*(^1^H,^1^H)=6.6 Hz, 6H, CH(C *H*_3_)_2_). ^**11**^ **B NMR** (161 MHz, THF‐*d*_8_) *δ*=−15.10 (s).^**13**^ **C NMR** (126 MHz, THF‐*d*_8_) *δ*=153.06–152.58 (m, Ar^F^), 151.17–150.78 (m, Ar^F^), 150.74 (s, *o*‐Dipp), 149.41 (s, *o*‐Dipp), 149.18 (s, N−C−N), 142.54–142.13 (m, Ar^F^), 140.37 (s, *ipso*‐Dipp), 140.63–140.02 (m, 2×Ar^F^), 139.27 (s, *ipso*‐Dipp), 138.54–137.92 (m, Ar^F^), 131.26 (s, *p*‐Dipp), 130.94 (s, *p*‐Dipp), 130.32 (s, H *C* =CB), 125.97 (s, *m*‐Dipp), 125.45 (s, *m*‐Dipp), 99.08 (d, ^1^ *J*(^13^C,^103^Rh)=9.6 Hz, C=C(Cp*)), 31.20 (s, *C* H(CH_3_)_2_), 31.00 (s, *C* H(CH_3_)_2_), 28.89 (s, CH(*C* H_3_)_2_), 26.58 (s, CH(*C* H_3_)_2_), 25.98 (s, CH(*C* H_3_)_2_), 25.43 (s, CH(*C* H_3_)_2_), 11.31 (s, Me−Cp*). A signal corresponding to HC= *C* B was not observed. ^**19**^ **F NMR** (471 MHz, THF‐*d*_8_) *δ*=−164.78 (s), −168.71 (s). One CF signal was too broad and could not be resolved. ^**77**^ **Se NMR** (95 MHz, THF‐*d*_8_) *δ*=117.5 (s). The ^1^ *J*(^77^Se,^103^Rh) coupling could not be resolved. **EA** – Anal. Calc. for C_55_H_50_BClF_15_N_2_RhSe: C, 52.76; H, 4.03; N, 2.24. Found: C, 52.41; H, 3.99; N, 2.19.

**Synthesis of [{(WCA‐IDipp)Se}IrCl(*η* ^5^‐C_5_Me_5_)] (8)**: (WCA‐IDipp)SeSiMe_3_ (20 mg, 0.0199 mmol, 1 equiv.) and [(*η* ^5^‐C_5_Me_5_)IrCl_2_]_2_ (7.55 mg, 0.0095 mmol, 0.5 equiv.) were mixed and dissolved in 2 mL toluene. The mixture turns dark brown within 2 min and was stirred for a total of 15 min at room temperature. The solvent was removed in *vacuo* and the resulting dark orange‐brown solid was washed with 2×1 mL Et_2_O and subsequently extracted with 0.2 mL THF, giving a dark brown clear solution. The solvent was removed in *vacuo* and the residual THF was removed co‐evaporation with *n*‐hexane, yielding the product as a dark orange‐brown solid (11 mg, 0.0082 mmol, 43 %). ^**1**^ **H NMR** (500 MHz, THF‐*d*_8_) *δ*=7.36–7.31 (m, 1H, *p*‐Dipp), 7.27–7.20 (m, 3H, *p*‐Dipp & *m*‐Dipp), 6.98 (d, ^3^ *J*(^1^H,^1^H)=7.1 Hz, 2H, *m*‐Dipp), 6.79 (s, 1H, HC=CB), 2.81–2.72 (m, 4H, 2×C *H* (CH_3_)_2_), 1.32 (d, ^3^ *J*(^1^H,^1^H)=6.6 Hz, 6H, CH(C *H*_3_)_2_), 1.29 (s, 15H, Cp*), 0.98 (d, ^3^ *J*(^1^H,^1^H)=6.9 Hz, 6H, CH(C *H*_3_)_2_), 0.86–0.76 (m, 12H, 2×CH(C *H*_3_)_2_). ^**11**^ **B NMR** (161 MHz, THF‐*d*_8_) *δ*=−15.04 (s). ^**13**^ **C NMR** (126 MHz, THF‐*d*_8_) *δ*=151.11–150.34 (m, Ar^F^), 149.00–148.48 (m, Ar^F^), 147.41 (s, *o*‐Dipp), 146.98 (s, *o*‐Dipp), 146.74 (s, N−C−N), 140.98–140.42 (m, Ar^F^), 138.91–138.20 (m, 2×Ar^F^), 136.76–136.17 (m, Ar^F^), 134.97 (s, *ipso*‐Dipp), 134.60 (s, *ipso*‐Dipp), 134.02 (s, H *C* =CB), 131.04 (s, *p*‐Dipp), 130.85 (s, *p*‐Dipp), 125.18 (s, *m*‐Dipp), 124.61 (s, *m*‐Dipp), 91.19 (s, C=C(Cp*)), 29.88 (s, *C* H(CH_3_)_2_), 28.64 (s, *C* H(CH_3_)_2_), 26.64 (s, CH(*C* H_3_)_2_), 26.37 (s, CH(*C* H_3_)_2_), 23.97 (s, CH(*C* H_3_)_2_), 23.22 (s, CH(*C* H_3_)_2_), 9.64 (s, Me−Cp*). A signal corresponding to HC= *C* B was not observed. ^**19**^ **F NMR** (471 MHz, THF‐*d*_8_) *δ*=−162.16 (s), −167.04 (s). One CF signal was too broad and could not be resolved. ^**77**^ **Se NMR** (95 MHz, THF‐*d*_8_) *δ*=122.4 (s). **EA** – Anal. Calc. for C_55_H_50_BClF_15_N_2_IrSe: C, 49.25; H, 3.76; N, 2.09. Found: C, 49.62; H, 4.03; N, 1.88.

**Synthesis of [{(WCA‐IDipp)S}Rh(COD)] (9)**: [(WCA‐IDipp)S]Li ⋅ (toluene) (100 mg, 0.097 mmol, 1 equiv.) was suspended in 3 mL toluene and [Rh(COD)Cl]_2_ (24 mg, 0.049 mmol, 0.5 equiv.) was added as one solid portion. The orange mixture was stirred for 5 h at room temperature and subsequently filtered, giving an orange solution. This solution was layered with *n*‐hexane at room temperature, forming orange crystals within 7 d, which were collected by decantation of the supernatant solution. Residual solvent was removed in *vacuo*, yielding the product as an orange crystalline solid (79 mg, 0.069 mmol, 71 %). ^**1**^ **H NMR** (400 MHz, C_6_D_6_) *δ*=7.21 (t, ^3^ *J*(^1^H,^1^H)=7.8 Hz, 1H, *p*‐Dipp), 6.99 (d, ^3^ *J*(^1^H,^1^H)=7.8 Hz, 2H, *m*‐Dipp), 6.72–6.63 (m, 3H, *p*‐Dipp & *m*‐Dipp), 5.96 (s, 1H, HC=CB),3.88–3.72 (m, 2H, HC=CH(COD)), 3.16–3.08 (m, 4H, HC=CH(COD) & C *H* (CH_3_)_2_), 2.80–2.71 (m, 2H, C *H* (CH_3_)_2_), 1.65–1.52 (m, 10H, H_2_C‐CH_2_(COD) & CH(C *H*_3_)_2_), 1.41 (d, ^3^ *J*(^1^H,^1^H)=6.8 Hz, 6H, CH(C *H*_3_)_2_), 1.13 (d, ^3^ *J*(^1^H,^1^H)=6.6 Hz, 6H, CH(C *H*_3_)_2_), 1.04 (d, ^3^ *J*(^1^H,^1^H)=6.8 Hz, 6H, CH(C *H*_3_)_2_) 0.96–0.89 (m, 4H, HC=CH(COD)). ^**11**^ **B NMR** (128 MHz, C_6_D_6_) *δ*=−15.30 (s). ^**13**^ **C NMR** (151 MHz, C_6_D_6_) *δ*=162.98 (s, N−C−N), 150.24–149.45 (m, Ar^F^), 148.69–147.93 (m, Ar^F^), 147.09 (s, *o*‐Dipp), 146.01 (s, *o*‐Dipp), 140.29–139.67 (m, Ar^F^), 138.50–138.12 (m, Ar^F^), 138.12–137.51 (m, Ar^F^), 136.42–135.94 (m, Ar^F^), 132.84 (s, *ipso*‐Dipp), 131.31 (s, *p*‐Dipp), 130.63 (s, *p*‐Dipp), 129.87 (s, *m*‐Dipp), 126.00 (s, *ipso*‐Dipp), 124.15 (s, *m*‐Dipp), 80.80 (s, C=C(COD)), 76.09 (s, C=C(COD)), 29.47 (s, *C* H(CH_3_)_2_), 28.88 (s, C−C(COD)), 28.35 (s, *C* H(CH_3_)_2_), 27.15 (s, C−C(COD)), 25.71 (s, CH(*C* H_3_)_2_), 24.95 (s, CH(*C* H_3_)_2_), 23.50 (s, CH(*C* H_3_)_2_), 22.50 (s, CH(*C* H_3_)_2_). A signal corresponding to HC= *C* B was not observed. A signal corresponding to HC= *C* B is overlayed with the residual solvent signal of the C_6_D_6_. ^**19**^ **F NMR** (377 MHz, C_6_D_6_) *δ*=−127.20–−131.31 (m), −160.18 (t, ^3^ *J*(^19^F,^19^F)=21.0 Hz), −165.67 (br s). **EA** – Anal. Calc. for C_53_H_47_BF_15_N_2_RhS ⋅ 0.5(C_2_H_2_Cl_2_): C, 54.22; H, 4.08; N, 2.36. Found: C, 54.51; H, 3.92; N, 2.10.

**Synthesis of [{(WCA‐IDipp)S}Ir(COD)] (10)**: [(WCA‐IDipp)S]Li ⋅ (toluene) (100 mg, 0.097 mmol, 1 equiv.) was suspended in 3 mL toluene and [Ir(COD)Cl]_2_ (33 mg, 0.049 mmol, 0.5 equiv.) was added as one solid portion. The orange mixture was stirred for 6 h at room temperature and subsequently filtered, giving an orange solution. This solution was layered with *n*‐hexane and stored at −30 °C, forming orange crystals within 3 d, which were collected by decantation of the supernatant solution. Residual solvent was removed in *vacuo*, yielding the product as an orange crystalline solid (88 mg, 0.071 mmol, 74 %). ^**1**^ **H NMR** (400 MHz, THF‐*d*_8_) *δ*=7.57–7.51 (m, 3H, *p*‐Dipp & *m*‐Dipp), 7.30 (t, ^3^ *J*(^1^H,^1^H)=7.8 Hz, 1H, *p*‐Dipp), 7.03 (d, ^3^ *J*(^1^H,^1^H)=7.8 Hz, 2H, *m*‐Dipp), 6.10 (s, 1H, HC=CB), 3.95 (s, 2H, HC=CH(COD)), 2.83 (sept, ^3^ *J*(^1^H,^1^H)=6.8 Hz, 2H, C *H* (CH_3_)_2_), 2.42 (sept, ^3^ *J*(^1^H,^1^H)=6.8 Hz, 2H, C *H* (CH_3_)_2_), 2.11–1.93 (m, 6H, HC=CH(COD) & H_2_C‐CH_2_(COD)), 1.59 (d, ^3^ *J*(^1^H,^1^H)=6.9 Hz, 6H, CH(C *H*_3_)_2_), 1.31 (d, ^3^ *J*(^1^H,^1^H)=9.8 Hz, 4H, H_2_C‐CH_2_(COD)), 1.15 (d, ^3^ *J*(^1^H,^1^H)=6.8 Hz, 6H, CH(C *H*_3_)_2_), 1.00 (d, ^3^ *J*(^1^H,^1^H)=6.8 Hz, 6H, CH(C *H*_3_)_2_), 0.84 (d, ^3^ *J*(^1^H,^1^H)=6.6 Hz, 6H, CH(C *H*_3_)_2_). ^**11**^ **B NMR** (161 MHz, THF‐*d*_8_) *δ*=−15.20 (s). ^**13**^ **C NMR** (126 MHz, THF‐*d*_8_) *δ*=168.96 (s, N−C−N), 150.98–150.38 (m, Ar^F^), 148.92–148.48 (m, Ar^F^), 148.15 (s, *o*‐Dipp), 147.00 (s, *o*‐Dipp), 141.01–140.30 (m, Ar^F^), 139.06–138.09 (m, 2×Ar^F^), 136.77–136.20 (m, Ar^F^), 134.89 (s, *p*‐Dipp), 133.64 (s, *ipso*‐Dipp), 131.35 (s, *p*‐Dipp), 130.36 (s, *m*‐Dipp), 128.33 (s, H *C* =CB), 124.75 (s, *m*‐Dipp), 122.41 (s, *ipso*‐Dipp), 80.37 (s, C=C(COD)), 62.65 (s, C=C(COD)), 32.86 (s, C−C(COD)), 31.46 (s, *C* H(CH_3_)_2_), 29.05 (s, *C* H(CH_3_)_2_), 28.29 (s, C−C(COD)), 26.04 (s, CH(*C* H_3_)_2)_ 23.89 (s, CH(*C* H_3_)_2_), 22.97 (s, CH(*C* H_3_)_2_). A signal corresponding to CH(*C* H_3_)_2_ is overlayed with the residual solvent signal of the THF‐*d*_8_. A signal corresponding to HC= *C* B was not observed. ^**19**^ **F NMR** (377 MHz, THF‐*d*_8_) *δ*=−126.36–−131.30 (m), −162.06 (t, ^3^ *J*(^19^F,^19^F)=20.1 Hz), −166.70 (br s). **EA** – Anal. Calc. for C_53_H_47_BF_15_IrN_2_S ⋅ 2(C_7_H8): C, 56.82; H, 4.48; N, 1.98. Found: C, 56.31; H, 4.66; N, 1.84.

**Synthesis of [{(WCA‐IDipp)Se}Rh(COD)] (11)**: [(WCA‐IDipp)Se]Li ⋅ (toluene) (150 mg, 0.139 mmol, 1 equiv.) was suspended in 3 mL toluene and [Rh(COD)Cl]_2_ (34 mg, 0.070 mmol, 0.5 equiv.) was added as one solid portion. The orange mixture was stirred for 8 h at room temperature and subsequently filtered, giving an orange solution. This solution was layered with *n*‐hexane and stored at −30 °C, forming orange crystals within 7 d, which were collected by decantation of the supernatant solution. Residual solvent was removed in *vacuo*, yielding the product as an orange crystalline solid (75 mg, 0.063 mmol, 45 %). ^**1**^ **H NMR** (500 MHz, THF‐*d*_8_) *δ*=7.66 (t, ^3^ *J*(^1^H,^1^H)=7.8 Hz, 1H, *p*‐Dipp), 7.54–7.46 (m, 3H, *p*‐Dipp & *m*‐Dipp), 7.24 (d, ^3^ *J*(^1^H,^1^H)=7.8 Hz, 2H, *m*‐Dipp), 6.72 (s, 1H, HC=CB), 3.82–3.74 (m, 2H, HC=CH(COD)), 2.95–2.76 (m, 4H, HC=CH(COD) & C *H* (CH_3_)_2_), 2.44–2.35 (m, 2H, C *H* (CH_3_)_2_), 1.88–1.74 (m, 4H, H_2_C‐CH_2_(COD)), 1.55–1.46 (m, 4H, H_2_C‐CH_2_(COD)), 1.44 (d, ^3^ *J*(^1^H,^1^H)=6.5 Hz, 6H, CH(C *H*_3_)_2_), 1.30 (d, ^3^ *J*(^1^H,^1^H)=6.4 Hz, 6H, CH(C *H*_3_)_2_), 0.90 (d, ^3^ *J*(^1^H,^1^H)=6.5 Hz, 6H, CH(C *H*_3_)_2_), 0.83 (d, ^3^ *J*(^1^H,^1^H)=5.8 Hz, 6H, CH(C *H*_3_)_2_). ^**11**^ **B NMR** (161 MHz, THF‐*d*_8_) *δ*=−14.77 (s). ^**13**^ **C NMR** (126 MHz, THF‐*d*_8_) *δ*=153.39 (q, ^1^ *J*(^11^B,^13^C)=59 Hz, HC= *C* B), 151.13–150.51 (m, Ar^F^), 149.27–148.54 (m, Ar^F^), 147.55 (s, *o*‐Dipp), 147.30 (s, *o*‐Dipp), 141.26 (s, N−C−N), 140.99–140.56 (m, Ar^F^), 139.02–138.23 (m, 2×Ar^F^), 136.84–136.54 (m, Ar^F^), 136.46 (s, *ipso*‐Dipp), 135.46 (s, *ipso*‐Dipp), 134.83 (s, H *C* =CB), 132.22 (s, *p*‐Dipp), 131.50 (s, *p*‐Dipp), 126.16 (s, 2×*m*‐Dipp), 78.87 (s, C=C(COD)), 75.09 (s, C=C(COD)), 31.30 (s, *C* H(CH_3_)_2_), 30.91 (s, C−C(COD)), 29.66 (s, *C* H(CH_3_)_2_), 29.34 (s, C−C(COD)), 27.75 (s, CH(*C* H_3_)_2_), 26.36 (s, CH(*C* H_3_)_2_), 23.22 (s, 2×CH(*C* H_3_)_2_). ^**19**^ **F NMR** (377 MHz, THF‐*d*_8_) *δ*=−133.03–−134.70 (m), −161.70 (br s), −166.31 (br s). ^**77**^ **Se NMR** (95 MHz, THF‐*d*_8_) *δ*=37.57 (s). The ^1^ *J*(^77^Se,^103^Rh) coupling could not be resolved. **EA** – Anal. Calc. for C_53_H_47_BF_15_N_2_RhSe ⋅ (C_2_H_2_Cl_2_): C, 50.89; H, 3.88; N, 2.20. Found: C, 50.62; H, 3.54; N, 1.83.

**Synthesis of [{(WCA‐IDipp)Se}Ir(COD)] (12)**: [(WCA‐IDipp)Se]Li ⋅ (toluene) (150 mg, 0.139 mmol, 1 equiv.) was suspended in 3 mL toluene and [Ir(COD)Cl]_2_ (46 mg, 0.070 mmol, 0.5 equiv.) was added as one solid portion. The red mixture was stirred for overnight at room temperature and subsequently filtered, giving a red orange solution. This solution was layered with *n*‐hexane and stored at −30 °C, forming red orange crystals within 7 d, which were collected by decantation of the supernatant solution. Residual solvent was removed in *vacuo*, yielding the product as a red orange crystalline solid (94 mg, 0.073 mmol, 53 %). ^**1**^ **H NMR** (500 MHz, THF‐*d*_8_) *δ*=7.53–7.44 (m, 3H, *p*‐Dipp & *m*‐Dipp), 7.30 (t, ^3^ *J*(^1^H,^1^H)=7.8 Hz, 1H, *p*‐Dipp), 7.04 (d, ^3^ *J*(^1^H,^1^H)=7.8 Hz, 2H, *m*‐Dipp), 6.15 (s, 1H, HC=CB), 3.84–3.75 (m, 2H, HC=CH(COD)), 2.79 (sept, ^3^ *J*(^1^H,^1^H)=6.6 Hz, 2H, C *H* (CH_3_)_2_), 2.60–2.52 (m, 2H, HC=CH(COD)), 2.35 (sept, ^3^ *J*(^1^H,^1^H)=6.7 Hz, 2H, C *H* (CH_3_)_2_), 2.02–1.91 (m, 4H, H_2_C‐CH_2_(COD)), 1.55 (d, ^3^ *J*(^1^H,^1^H)=6.9 Hz, 6H, CH(C *H*_3_)_2_), 1.24–1.18 (m, 4H, H_2_C‐CH_2_(COD)), 1.15 (d, ^3^ *J*(^1^H,^1^H)=6.7 Hz, 6H, CH(C *H*_3_)_2_), 0.97 (d, ^3^ *J*(^1^H,^1^H)=6.8 Hz, 6H, CH(C *H*_3_)_2_), 0.80 (d, ^3^ *J*(^1^H,^1^H)=6.5 Hz, 6H, CH(C *H*_3_)_2_). ^**11**^ **B NMR** (161 MHz, THF‐*d*_8_) *δ*=−15.16 (s). ^**13**^ **C NMR** (126 MHz, THF‐*d*_8_) *δ*=150.96–150.26 (m, Ar^F^), 148.96–148.61 (m, Ar^F^), 148.02 (s, *o*‐Dipp), 146.57 (s, *o*‐Dipp), 141.13–140.26 (m, Ar^F^), 140.81 (s, N−C−N), 139.24–137.77 (m, 2×Ar^F^), 136.92–136.05 (m, Ar^F^), 134.59 (s, *ipso*‐Dipp), 134.48 (s, *p*‐Dipp), 131.40 (s, *p*‐Dipp), 130.48 (s, H *C* =CB), 130.04 (s, *m*‐Dipp), 124.97 (s, *m*‐Dipp), 123.09 (s, *ipso*‐Dipp), 82.42 (s, C=C(COD)), 62.76 (s, C=C(COD)), 32.54 (s, C−C(COD)), 31.49 (s, *C* H(CH_3_)_2_), 30.27 (s, C−C(COD)), 29.06 (s, *C* H(CH_3_)_2_), 26.44 (s, CH(*C* H_3_)_2_), 23.89 (s, CH(*C* H_3_)_2_), 23.17 (s, CH(*C* H_3_)_2_). A signal corresponding to CH(*C* H_3_)_2_ is overlayed with the residual solvent signal of the THF‐*d*_8_. A signal corresponding to HC= *C* B was not observed. ^**19**^ **F NMR** (471 MHz, THF‐*d*_8_) *δ*=−128.90–−133.80 (m), −162.01 (t, ^3^ *J*(^19^F,^19^F)=20.1 Hz), −166.68 (s). ^**77**^ **Se NMR** (95 MHz, THF‐*d*_8_) *δ*=53.42 (s). **EA** – Anal. Calc. for C_53_H_47_BF_15_N_2_IrSe ⋅ (C_6_H_14_): C, 51.91; H, 4.50; N, 2.05. Found: C, 51.63; H, 4.41; N, 2.09.

**Synthesis of [{(WCA‐IDipp)S}Rh_2_(COD)_2_Cl] (13)**: [(WCA‐IDipp)S]Li ⋅ (toluene) (100 mg, 0.097 mmol, 1 equiv.) was suspended in 3 mL toluene and [Rh(COD)Cl]_2_ (48 mg, 0.097 mmol, 1 equiv.) was added as one solid portion. The orange mixture was stirred for 30 h at room temperature and subsequently filtered, giving a orange solution. This solution was layered with *n*‐hexane at room temperature, forming orange crystals within 7 d, which were collected by decantation of the supernatant solution. Residual solvent was removed in *vacuo*, yielding the product as an orange crystalline solid (112 mg, 0.081 mmol, 83 %). ^**1**^ **H NMR** (500 MHz, THF‐*d*_8_) *δ*=7.32 (t, ^3^ *J*(^1^H,^1^H)=7.8 Hz, 1H, *p*‐Dipp), 7.12 (t, ^3^ *J*(^1^H,^1^H)=7.8 Hz, 1H, *p*‐Dipp), 6.90 (d, ^3^ *J*(^1^H,^1^H)=7.7 Hz, 2H, *m*‐Dipp), 6.66 (d, ^3^ *J*(^1^H,^1^H)=7.8 Hz, 2H, *m*‐Dipp), 6.19 (s, 1H, HC=CB), 4.24–4.12 (m, 2H, HC=CH(COD)), 3.88–3.79 (m, 2H, HC=CH(COD)), 3.65–3.58 (m, 2H, HC=CH(COD)), 2.52–2.44 (m, 2H, C *H* (CH_3_)_2_), 2.36–2.31 (m, 2H, C *H* (CH_3_)_2_), 2.28–2.20 (m, 2H, HC=CH(COD)), 2.09–2.02 (m, 4H, 2×H_2_C‐CH_2_(COD)), 1.86–1.79 (m, 4H, H_2_C‐CH_2_(COD)), 1.49 (d, ^3^ *J*(^1^H,^1^H)=6.8 Hz, 6H, CH(C *H*_3_)_2_), 1.22–1.16 (m, 4H, 2×H_2_C‐CH_2_(COD)), 0.83 (d, ^3^ *J*(^1^H,^1^H)=6.8 Hz, 6H, CH(C *H*_3_)_2_), 0.70 (d, ^3^ *J*(^1^H,^1^H)=6.8 Hz, 6H, CH(C *H*_3_)_2_), 0.55–0.46 (m, *J*=11.8 Hz, 10H, 2×H_2_C‐CH_2_(COD) & CH(C *H*_3_)_2_). ^**11**^ **B NMR** (161 MHz, THF‐*d*_8_) *δ*=−14.62 (s). ^**13**^ **C NMR** (126 MHz, THF‐*d*_8_) *δ*=164.29 (s, N−C−N), 154.23 (q, ^1^ *J*(^11^B,^13^C)=62.4 Hz, HC= *C* B), 150.91–150.48 (m, Ar^F^), 148.94–148.62 (m, Ar^F^), 148.17 (s, *o*‐Dipp), 147.82–147.43 (m, Ar^F^), 146.98 (s, *o*‐Dipp), 140.98–140.45 (m, Ar^F^), 139.07–138.20 (m, Ar^F^), 136.83–136.13 (m, Ar^F^), 135.82 (s, *p*‐Dipp), 134.81 (s, *p*‐Dipp), 133.75 (s, *ipso*‐Dipp), 129.72 (s, H *C* =CB), 128.31 (s, *ipso*‐Dipp), 126.30 (s, *m*‐Dipp), 124.69 (s, *m*‐Dipp), 87.29–86.76 (m, C=C(COD)), 86.62 (d, ^1^ *J*(^103^Rh,^13^C)=11.5 Hz, C=C(COD)), 78.96 (d, ^1^ *J*(^103^Rh,^13^C)=13.9 Hz, 2×C=C(COD)), 31.64 (s, 2×C−C(COD)), 30.69 (s, *C* H(CH_3_)_2_), 29.86 (s, C−C(COD)), 29.32 (s, C−C(COD)), 29.05 (s, *C* H(CH_3_)_2_), 26.35 (s, C−C(COD)), 26.15 (s, C−C(COD)), 25.80 (s, CH(*C* H_3_)_2_), 24.02 (s, CH(*C* H_3_)_2_), 23.01 (s, CH(*C* H_3_)_2_). Signals corresponding to 2×C−C(COD) and CH(*C* H_3_)_2_ are overlayed with the residual solvent signal of the THF‐*d*_8_. ^**19**^ **F NMR** (283 MHz, THF‐*d*_8_) *δ*=−126.87–−131.41 (m), −158.90 (t, ^3^ *J*(^19^F,^19^F)=20.7 Hz), −164.14–−165.97 (m). **EA** – Anal. Calc. for C_61_H_59_BClF_15_N_2_Rh_2_S: C, 53.42; H, 4.62; N, 1.92. Found: C, 53.37; H, 4.37; N, 1.80.

**Synthesis of [{(WCA‐IDipp)S}Ir_2_(COD)_2_Cl] (14)**: [(WCA‐IDipp)S]Li ⋅ (toluene) (100 mg, 0.097 mmol, 1 equiv.) was suspended in 3 mL toluene and [Ir(COD)Cl]_2_ (66 mg, 0.097 mmol, 1 equiv.) was added as one solid portion. The red mixture was stirred overnight at room temperature and subsequently filtered, giving a red purple solution. This solution was layered with *n*‐hexane at room temperature, forming purple crystals within 7 d, which were collected by decantation of the supernatant solution. Residual solvent was removed in *vacuo*, yielding the product as a purple crystalline solid (98 mg, 0.063 mmol, 64 %). ^**1**^ **H NMR** (500 MHz, THF‐*d*_8_) *δ*=7.52 (d, ^3^ *J*(^1^H,^1^H)=7.8 Hz, 2H, *m*‐Dipp), 7.49 (t, ^3^ *J*(^1^H,^1^H)=7.7 Hz, 1H, *p*‐Dipp), 7.28 (t, ^3^ *J*(^1^H,^1^H)=7.8 Hz, 1H, *p*‐Dipp), 7.02 (d, ^3^ *J*(^1^H,^1^H)=7.8 Hz, 2H, *m*‐Dipp), 6.09 (s, 1H, HC=CB), 4.11–4.06 (m, 2H, HC=CH(COD)), 3.97–3.89 (m, 2H, HC=CH(COD)), 2.85–2.77 (m, 2H, C *H* (CH_3_)_2_), 2.44–2.35 (m, 2H, C *H* (CH_3_)_2_), 2.19–1.92 (m, 10H, 2×HC=CH(COD) & 3×H_2_C‐CH_2_(COD)), 1.58 (d, ^3^ *J*(^1^H,^1^H)=6.8 Hz, 6H, CH(C *H*_3_)_2_), 1.40–1.24 (m, 10H, 5×H_2_C‐CH_2_(COD)), 1.13 (d, ^3^ *J*(^1^H,^1^H)=6.8 Hz, 6H, CH(C *H*_3_)_2_), 0.98 (d, ^3^ *J*(^1^H,^1^H)=6.8 Hz, 6H, CH(C *H*_3_)_2_), 0.82 (d, ^3^ *J*(^1^H,^1^H)=6.6 Hz, 6H, CH(C *H*_3_)_2_). ^**11**^ **B NMR** (161 MHz, THF‐*d*_8_) *δ*=−15.20 (s). ^**13**^ **C NMR** (126 MHz, THF‐*d*_8_) *δ*=168.96 (s, N−C−N), 150.90–150.46 (m, Ar^F^), 149.07–148.56 (m, Ar^F^), 148.14 (s, *o*‐Dipp), 146.99 (s, *o*‐Dipp), 140.94–140.43 (m, Ar^F^), 138.97–138.23 (m, 2×Ar^F^), 136.77–136.20 (m, Ar^F^), 134.89 (s, *p*‐Dipp), 133.63 (s, *ipso*‐Dipp), 131.35 (s, *p*‐Dipp), 130.38 (s, *m*‐Dipp), 128.31 (s, H *C* =CB), 124.75 (s, *m*‐Dipp), 122.40 (s, *ipso*‐Dipp), 80.38 (s, C=C(COD)), 62.55 (s, C=C(COD)), 32.85 (s, 2×C−C(COD)), 32.53 (s, 2×C−C(COD)), 31.45 (s, *C* H(CH_3_)_2_), 29.05 (s, *C* H(CH_3_)_2_), 28.27 (s, 2×C−C(COD)), 26.04 (s, CH(*C* H_3_)_2_), 23.88 (s, CH(*C* H_3_)_2_), 22.97 (s, CH(*C* H_3_)_2_). A signal corresponding to HC= *C* B was not observed and the signals corresponding to 2×C−C(COD) and CH(*C* H_3_)_2_ are overlayed with the residual solvent signal of the THF‐*d*_8_. ^**19**^ **F NMR** (471 MHz, THF‐*d*_8_) *δ*=−131.74–133.91 (m), −163.06 (s), −167.73 (s). **EA** – Anal. Calc. for C_61_H_59_BClF_15_N_2_Ir_2_S ⋅ (C_7_H_8_): C, 49.20; H, 4.07; N, 1.69. Found: C, 49.22; H, 4.05; N, 1.91.

**Synthesis of [{(WCA‐IDipp)Se}Rh_2_(COD)_2_Cl] (15)**: [(WCA‐IDipp)Se]Li ⋅ (toluene) (100 mg, 0.093 mmol, 1 equiv.) was suspended in 3 mL toluene and [Rh(COD)Cl]_2_ (46 mg, 0.093 mmol, 1 equiv.) was added as one solid portion. The red orange mixture was stirred for 3 d at room temperature and subsequently filtered, giving a red orange solution. This solution was layered with *n*‐hexane and stored at −30 °C, forming red orange crystals within 7 d, which were collected by decantation of the supernatant solution. Residual solvent was removed in *vacuo*, yielding the product as a red orange crystalline solid (44 mg, 0.031 mmol, 33 %). ^**1**^ **H NMR** (500 MHz, THF‐*d*_8_) *δ*=7.64 (t, ^3^ *J*(^1^H,^1^H)=7.8 Hz, 1H, *p*‐Dipp), 7.49 (d, ^3^ *J*(^1^H,^1^H)=7.7 Hz, 2H, *m*‐Dipp), 7.46 (t, ^3^ *J*(^1^H,^1^H)=7.8 Hz, 1H, *p*‐Dipp), 7.22 (d, ^3^ *J*(^1^H,^1^H)=7.8 Hz, 2H, *m*‐Dipp), 6.70 (s, 1H, HC=CB), 4.52–4.45 (m, 2H, HC=CH(COD)), 4.26–4.19 (m, 2H, HC=CH(COD)), 3.79–3.71 (m, 2H, HC=CH(COD)), 2.91–2.76 (m, 4H, C *H* (CH_3_)_2_ & HC=CH(COD)), 2.56–2.44 (m, 2H, H_2_C‐CH_2_(COD)), 2.41–2.26 (m, 4H, C *H* (CH_3_)_2_ & H_2_C‐CH_2_(COD)), 2.18–2.08 (m, 2H, H_2_C‐CH_2_(COD)), 1.86–1.73 (m, 6H, 3×H_2_C‐CH_2_(COD)), 1.53–1.46 (m, 2H, H_2_C‐CH_2_(COD)), 1.45–1.36 (m, 8H, H_2_C‐CH_2_(COD) & CH(C *H*_3_)_2_), 1.28 (d, ^3^ *J*(^1^H,^1^H)=6.9 Hz, 6H, CH(C *H*_3_)_2_), 0.88 (d, ^3^ *J*(^1^H,^1^H)=6.5 Hz, 6H, CH(C *H*_3_)_2_), 0.81 (d, ^3^ *J*(^1^H,^1^H)=5.7 Hz, 6H, CH(C *H*_3_)_2_). ^**11**^ **B NMR** (161 MHz, THF‐*d*_8_) *δ*=−14.77 (s). ^**13**^ **C NMR** (126 MHz, THF‐*d*_8_) *δ*=153.38 (d, ^1^ *J*(^11^B,^13^C)=59.5 Hz, HC= *C* B), 151.08–150.41 (m, Ar^F^), 149.22–148.60 (m, Ar^F^), 147.55 (s, *o*‐Dipp), 147.29 (s, *o*‐Dipp), 141.27 (s, N−C−N), 140.97–140.57 (m, Ar^F^), 139.06–138.16 (m, 2×Ar^F^), 136.89–136.57 (m, Ar^F^), 136.46 (s, *ipso*‐Dipp), 135.46 (s, *ipso*‐Dipp), 134.79 (s, H *C* =CB), 132.22 (s, *p*‐Dipp), 131.49 (s, *p*‐Dipp), 126.16 (s, 2×*m*‐Dipp), 87.79 (d, ^1^ *J*(^103^Rh,^13^C)=10.6 Hz, C=C(COD)), 87.18 (d, ^1^ *J*(^103^Rh,^13^C)=12.8 Hz, C=C(COD)), 78.95 (d, ^1^ *J*(^103^Rh,^13^C)=13.9 Hz, C=C(COD)), 75.07 (s, C=C(COD)), 34.76 (s, 2×C−C(COD)), 31.64 (s, 2×C−C(COD)), 30.91 (s, 2×C−C(COD)), 29.83 (s, *C* H(CH_3_)_2_), 29.67 (s, 2×C−C(COD)), 29.34 (s, *C* H(CH_3_)_2_), 27.74 (s, CH(*C* H_3_)_2_), 26.36 (s, CH(*C* H_3_)_2_), 23.51 (s, CH(*C* H_3_)_2_), 23.22 (s, CH(*C* H_3_)_2_). ^**19**^ **F NMR** (471 MHz, THF‐*d*_8_) *δ*=−134.33–−135.11 (m), −162.64 (s), −166.96–−167.95 (m). ^**77**^ **Se NMR** (95 MHz, THF‐*d*_8_) *δ*=37.66 (s). The ^1^ *J*(^77^Se,^103^Rh) coupling could not be resolved. **EA** – Anal. Calc. for C_61_H_59_BClF_15_N_2_Rh_2_Se: C, 51.02; H, 4.14; N, 1.95. Found: C, 50.65; H, 4.16; N, 1.58.

**Synthesis of [{(WCA‐IDipp)Se}Ir_2_(COD)_2_Cl] (16)**: [(WCA‐IDipp)Se]Li ⋅ (toluene) (100 mg, 0.093 mmol, 1 equiv.) was suspended in 3 mL toluene and [Ir(COD)Cl]_2_ (62 mg, 0.093 mmol, 1 equiv.) was added as one solid portion. The red purple mixture was stirred overnight at room temperature and subsequently filtered, giving a purple solution. This solution was layered with *n*‐hexane and stored at −30 °C, forming purple crystals within 5 d, which were collected by decantation of the supernatant solution. Residual solvent was removed in *vacuo*, yielding the product as a purple crystalline solid (55 mg, 0.034 mmol, 37 %). ^**1**^ **H NMR** (500 MHz, THF‐*d*_8_) *δ*=7.60 (t, ^3^ *J*(^1^H,^1^H)=7.8 Hz, 1H, *p*‐Dipp), 7.47 (t, ^3^ *J*(^1^H,^1^H)=7.8 Hz, 1H, *p*‐Dipp), 7.43 (d, ^3^ *J*(^1^H,^1^H)=7.8 Hz, 2H, *m*‐Dipp), 7.21 (d, ^3^ *J*(^1^H,^1^H)=7.7 Hz, 2H, *m*‐Dipp), 6.79 (s, 1H, HC=CB), 4.26–4.16 (m, 2H, HC=CH(COD)), 4.14–4.07 (m, 2H, HC=CH(COD)), 3.51–3.46 (m, 2H, HC=CH(COD)), 2.98–2.82 (m, 4H, HC=CH(COD) & C *H* (CH_3_)_2_), 2.42–2.32 (m, 2H, C *H* (CH_3_)_2_), 2.08–1.96 (m, 4H, 2×H_2_C‐CH_2_(COD)), 1.86–1.75 (m, 2H, H_2_C‐CH_2_(COD)), 1.71–1.64 (m, 2H, H_2_C‐CH_2_(COD)), 1.60–1.52 (m, 2H, H_2_C‐CH_2_(COD)), 1.42–1.32 (m, 8H, CH(C *H*_3_)_2_ & H2 C‐CH_2_(COD)), 1.28 (d, ^3^ *J*(^1^H,^1^H)=6.7 Hz, 6H, CH(C *H*_3_)_2_), 1.23–1.12 (m, 2H, H_2_C‐CH_2_(COD)), 1.09–1.01 (m, 2H, H_2_C‐CH_2_(COD)), 0.88 (d, ^3^ *J*(^1^H,^1^H)=6.3 Hz, 6H, CH(C *H*_3_)_2_), 0.83–0.75 (m, 6H, CH(C *H*_3_)_2_). ^**11**^ **B NMR** (161 MHz, THF‐*d*_8_) *δ*=−14.70 (s). ^**13**^ **C NMR** (126 MHz, THF‐*d*_8_) *δ*=154.20 (q, ^1^ *J*(^11^B,^13^C)=58.0 Hz, HC= *C* B), 151.02–150.20 (m, Ar^F^), 149.19–148.40 (m, Ar^F^), 147.33 (s, *o*‐Dipp), 147.16 (s, *o*‐Dipp), 140.93–140.55 (m, Ar^F^), 140.18 (s, N−C−N), 139.07–138.21 (m, 2×Ar^F^), 137.07–136.31 (m, Ar^F^), 136.22 (s, *ipso*‐Dipp), 135.17 (s, *ipso*‐Dipp & H *C* =CB), 132.40 (s, *p*‐Dipp), 131.70 (s, *p*‐Dipp), 126.48 (s, *m*‐Dipp), 126.16 (s, *m*‐Dipp), 74.01 (s, C=C(COD)), 71.10 (s, C=C(COD)), 62.06 (s, C=C(COD)), 60.25 (s, C=C(COD)), 35.09 (s, 2×C−C(COD)), 32.30 (s, 2×C−C(COD)), 31.83 (s, 2×C−C(COD)), 30.54 (s, 2×C−C(COD)), 29.98 (s, *C* H(CH_3_)_2_), 29.40 (s, *C* H(CH_3_)_2_), 27.93 (s, CH(*C* H_3_)_2_), 26.46 (s, CH(*C* H_3_)_2_), 23.63 (s, CH(*C* H_3_)_2_), 23.01 (s, CH(*C* H_3_)_2_). ^**19**^ **F NMR** (471 MHz, THF‐*d*_8_) *δ*=−133.26–−134.42 (m), −161.51 (br s), −165.81–−166.89 (m). ^**77**^ **Se NMR** (95 MHz, THF‐*d*_8_) *δ*=50.48 (s). **EA** – Anal. Calc. for C_61_H_59_BClF_15_Ir_2_N_2_Se: C, 45.37; H, 3.68; N, 1.73. Found: C, 45.43; H, 3.98; N, 1.43.

## Conflict of interest

The authors declare no conflict of interest.

1

## Supporting information

As a service to our authors and readers, this journal provides supporting information supplied by the authors. Such materials are peer reviewed and may be re‐organized for online delivery, but are not copy‐edited or typeset. Technical support issues arising from supporting information (other than missing files) should be addressed to the authors.

Supporting Information

## Data Availability

The data that support the findings of this study are available in the supplementary material of this article.
