# Supplementary material for: Rhodium and Iridium Complexes of Anionic Thione and Selone Ligands Derived from Anionic N‐Heterocyclic Carbenes
Source: Chemistry. 2021 Dec 22;28(4):e202104139. doi: 10.1002/chem.202104139 (PMC9305287; doi:10.1002/chem.202104139)
Supplement: Supplementary file 1 — Supporting Information [file CHEM-28-0-s001.pdf]

Chemistry–A European Journal

Supporting Information

Rhodium and Iridium Complexes of Anionic Thione and Selone Ligands Derived from Anionic N-Heterocyclic Carbenes

Luong Phong Ho, Angelika Neitzel, Thomas Bannenberg, and Matthias Tamm*

Content

S1 Synthesis of (WCA-IDipp)Se$(CH_2)_4$OSiMe_3 (**4A**) 1
S2 Crystallographic details 2
S2.1 (WCA-IDipp)SSiMe_3(**3**) 3
S2.2 (WCA-IDipp)SeSiMe_3·*n*-hexane (**4**·*n*-hexane) 5
S2.3 (WCA-IDipp)Se$(CH_2)_4$OSiMe_3·toluene (**4A**·toluene) 7
S2.4 [{(WCA-IDipp)S}RhCl(η^5-C_5Me_5)] (**5**·2(benzene)) 9
S2.5 [{(WCA-IDipp)S}IrCl(η^5-C_5Me_5)] (**6**·1.5(CH_2Cl_2)) 11
S2.6 [{(WCA-IDipp)Se}RhCl(η^5-C_5Me_5)] (**7**·(benzene)0.5(n-hexane)) 13
S2.6 [{(WCA-IDipp)Se}IrCl(η^5-C_5Me_5)] (**8**·CH_2Cl_2) 15
S2.4 [{(WCA-IDipp)S}Rh(COD)] (**9**) 17
S2.5 [{(WCA-IDipp)S}Ir(COD)]·1.5toluene·0.5*n*-hexane (**10**·1.5(toluene)0.5(*n*-hexane)) 19
S2.6 [{(WCA-IDipp)Se}Rh(COD)] (**11**) 21
S2.7 [{(WCA-IDipp)Se}Ir(COD)] (**12**) 23
S2.8 [{(WCA-IDipp)S}$Rh_2(COD)_2$Cl] (**13**) 25
S2.9 [{(WCA-IDipp)S}$Ir_2(COD)_2$Cl]·3.5THF (**14**·3.5(THF)) 27
S2.10 [{(WCA-IDipp)Se}$Rh_2(COD)_2$Cl] (**15**) 29
S2.11 [{(WCA-IDipp)Se}$Ir_2(COD)_2$Cl]·0.5THF·HMDSO (**16**·0.5(THF)0.5(HMDSO)) 31
S3 ^{1}H, ^{11}B, ^{13}C, ^{19}F, and ^{77}Se NMR Spectra 33
S3.1 (WCA-IDipp)SSiMe_3 (**3**) 33
S3.2 (WCA-IDipp)SeSiMe_3 (**4**) 37
S3.3 (WCA-IDipp)Se$(CH_2)_4$OSiMe_3 (**4A**) 42
S3.4 [{(WCA-IDipp)S}RhCl(η^5-C_5Me_5)] (**5**) 46
S3.5 [{(WCA-IDipp)S}IrCl(η^5-C_5Me_5)] (**6**) 50
S3.6 [{(WCA-IDipp)Se}RhCl(η^5-C_5Me_5)] (**7**) 54
S3.7 [{(WCA-IDipp)Se}IrCl(η^5-C_5Me_5)] (**8**) 59
S3.8 [{(WCA-IDipp)S}Rh(COD)] (**9**) 64
S3.9 [{(WCA-IDipp)S}Ir(COD)] (**10**) 68
S3.9 [{(WCA-IDipp)Se}Rh(COD)] (**11**) 72
S3.10 [{(WCA-IDipp)Se}Ir(COD)] (**12**) 77
S3.11 [{(WCA-IDipp)S}$Rh_2(COD)_2$Cl] (**13**) 82
S3.13 [{(WCA-IDipp)S}$Ir_2(COD)_2$Cl] (**14**) 86
S3.14 [{(WCA-IDipp)Se}$Rh_2(COD)_2$Cl] (**15**) 90
S3.15 [{(WCA-IDipp)Se}$Ir_2(COD)_2$Cl] (**16**) 95
S4 Computational Details 100
S4 References 107

S1 Synthesis of (WCA-IDipp)Se(CH_2)$_4$OSiMe$_3$ (4A)

(WCA-IDipp)SeSiMe$_3$ (**4**) is dissolved in THF and allowed to remain in THF solution for 1 h. The THF is afterwards removed in *vacuo* and the product can be obtained as a colorless solid in quantitative yield.

^{1}H NMR (600 MHz, THF-d_8, 298 K) δ = 7.23 (t, $^3J(^1H,^1H)$ = 7.8 Hz, 1H, *p*-Dipp), 7.11 (t, $^3J(^1H,^1H)$ = 7.8 Hz, 1H, *p*-Dipp), 6.94 (d, $^3J(^1H,^1H)$ = 7.8 Hz, 2H, *m*-Dipp), 6.91 (d, $^3J(^1H,^1H)$ = 7.8 Hz, 2H, *m*-Dipp), 6.77 (s, 1H, HC=CB), 3.03 (t, $^3J(^1H,^1H)$ = 5.9 Hz, 2H, CH_2), 2.92 (sept, $^3J(^1H,^1H)$ = 6.8 Hz, 2H, C$\underline{H}$(CH_3)$_2$), 2.56 (sept, $^3J(^1H,^1H)$ = 6.8 Hz, 2H, C$\underline{H}$(CH_3)$_2$), 2.14 (t, $^3J(^1H,^1H)$ = 7.5 Hz, 2H, CH_2), 1.17 – 1.12 (m, 12H, CH(C$\underline{H}_3$)$_2$), 1.06 (d, $^3J(^1H,^1H)$ = 5.9 Hz, 6H, CH(C$\underline{H}_3$)$_2$), 1.02 (d, $^3J(^1H,^1H)$ = 6.8 Hz, 6H, CH(C$\underline{H}_3$)$_2$), 0.91 – 0.87 (m, 2H, CH_2), 0.80 – 0.75 (m, 2H, CH_2), 0.05 (s, 9H, Si(CH_3)$_3$).) ppm.

^{11}B NMR (96 MHz, THF-d_8, 298 K) δ = –15.25 (s) ppm.

^{13}C NMR (151 MHz, C_6D_6, 298 K) δ = 150.60 – 150.27 (m, Ar^F), 148.85 – 148.72 (m, Ar^F), 146.84 (s, *o*-Dipp), 145.80 (s, *o*-Dipp), 140.70 – 140.43 (m, Ar^F), 139.16 – 138.72 (m, Ar^F), 138.42 – 138.18 (m, Ar^F), 138.03 (s, N–C–N), 136.83 – 136.46 (m, Ar^F), 133.50 (s, *ipso*-Dipp), 133.25 (s, H$\underline{C}$=CB), 131.88 (s, *p*-Dipp), 131.67 (s, *p*-Dipp), 131.52 (s, *ipso*-Dipp), 125.01 (s, *m*-Dipp), 124.52 (s, *m*-Dipp), 60.98 (s, CH_2), 31.89 (s, CH_2), 29.63 (s, CH_2), 29.09 (s, $\underline{C}$H(CH_3)$_2$), 28.45 (s, $\underline{C}$H(CH_3)$_2$), 25.52 (s, CH($\underline{C}H_3$)$_2$), 25.48 (s, CH($\underline{C}H_3$)$_2$), 24.88 (s, CH_2), 22.84 (s, CH($\underline{C}H_3$)$_2$), 22.44 (s, CH($\underline{C}H_3$)$_2$), –0.66 (s, Si(CH_3)$_3$) ppm.

^{19}F NMR (283 MHz, THF-d_8, 298 K) δ = –127.37 – –134.24 (m), -161.31 (t, $^3J(^{19}F,^{19}F)$ = 20.3 Hz), -166.30 (br s) ppm.

EA – Anal. Calc. for $C_{51}H_{50}BF_{15}N_2OSeSi$·0.5(toluene): C, 56.63; H, 4.71; N, 2.42. Found: C, 56.67; H, 4.62; N, 2.30.

S2 Crystallographic details

Suitable single crystals were mounted on a hair or on a MiTiGen mount in perfluorinated inert oil. The intensity measurements were performed at 100 K on a Rigaku XtaLAB Synergy S Single Source diffractometer using mirror-focused Cu*K*α radiation or a Rigaku XtaLAB Synergy S Single Source diffractometer using monochromated Mo*K*α radiation. The diffractometer software CrysAlisPRO was employed.[1] Absorption corrections were based on multiscans or face-indexation using a gaussian grid. The structures were refined anisotropically on F^2 using SHELXL-2018/3.[2] Hydrogen atoms were included using a riding model or rigid methyl groups. Further details are given in Table S1-11.

Solvent content: Compound **4** contains one co-crystallized n-hexane molecule, which is disordered over two positions. Compound **4A** contains one co-crystallized toluene molecule, which is ordered. Compound **5** contains two co-crystallized benzene molecules per asymmetric unit; one is ordered and two are located on a special position, thus each is half occupied. Compound **6** contains one and a half co-crystallized DCM molecules per asymmetric unit; they are disordered over several positions. Compound **7** contains one benzene and one half of a n-hexane molecule per asymmetric unit; the benzene molecules is disordered over two positions; the n-hexane molecule is located on a special position and is only half occupied. Compound **8** contains one co-crystallized DCM molecule per asymmetric unit; it is ordered. Compound **10** contains three co-crystallized toluene molecules and one half of a co-crystallized n-hexane molecule per asymmetric unit; two toluene molecules are ordered but only half occupied and two are each disordered over two positions. The n-hexane molecule is ordered but only half occupied. Compound **14** contains 3.5 co-crystallized THF molecules per asymmetric unit. One THF molecule is located on a special position and is only half occupied per asymmetric unit. One THF molecule is disordered over two positions. The remaining two THF molecules are ordered. Compound **16** contains one half of a co-crystallized THF and one half of a co-crystallized HMDSO molecule. The THF molecule is ordered but only half occupied and the HMDSO molecule is located on a special position and is only half occupied.

Exceptions and special details: The disordered solvent molecules in **6** and **10** were refined using appropriate restraints to improve stability of refinement, but the dimensions are not entirely satisfactory and should be interpreted with caution. The asymmetric unit of **10** contains two independent [(WCA-IDipp)SIr(COD)] molecules. A higher symmetric space group was checked but the refinement was not satisfactory.

Complete data have been deposited with the Cambridge Crystallographic Data Centre under the CCDC numbers 2122410–2122424 for compounds **3**–**16**. These data can be obtained free of charge from http://www.ccdc.cam.ac.uk/.

S2.1 (WCA-IDipp)SSiMe$_3$(**3**)

Table S1. Crystallographic data for compound **3**.

Compound	**3**	
Identification code	2122411	
Empirical formula	$C_{48}H_{44}BF_{15}N_2SSi$	
Formula weight	1004.81	
Temperature	100(2) K	
Wavelength	0.71073 Å	
Instrument (scan mode)	XtaLAB Synergy, Single source at offset/far, HyPix ([illegible] scan)	
Crystal system	Monoclinic	
Space group	$P2_1/n$	
Unit cell dimensions	a = 11.9552(4) Å	α = 90°
	b = 19.7042(6) Å	β = 107.242(4)°
	c = 20.1350(6) Å	γ = 90°
Volume	4530.0(3) Å^3	
Z	4	
Density (calculated)	1.473 Mg/m^3	
Absorption coefficient	0.198 mm^{-1}	
F(000)	2064	
Crystal habitus	block (irregular)	
Crystal size	0.164 x 0.103 x 0.065 mm^3	
Theta range for data collection	2.548 to 34.214°	
Index ranges	-18<=h<=18, -31<=k<=31, -30<=l<=30	
Reflections collected	291686	
Independent reflections	17562 [R(int) = 0.0748]	
Completeness to theta = 25.242°	99.9 %	
Absorption correction	Gaussian	
Max. and min. transmission	1.000 and 0.512	
Refinement method	Full-matrix least-squares on F^2	
Data / restraints / parameters	17562 / 0 / 624	
Goodness-of-fit on F^2	1.027	
Final R indices [I>2sigma(I)]	R1 = 0.0428, wR2 = 0.0953	
R indices (all data)	R1 = 0.0683, wR2 = 0.1030	
Largest diff. peak and hole	0.459 and -0.327 e.Å^{-3}	
Crystallisation Details:	A saturated solution of WCA-IDipp-SSiMe$_3$ in toluene/hexane (2:1 mix) was stored at -40C for several days.	
Solution	SHELXT-2014/5 (G. M. Sheldrick, Acta Cryst., 2015, A71, 3-8)	
Refinement	SHELXL-2018/3 (G. M. Sheldrick, Acta Cryst., 2008, A64, 112-122)	
Interface	OLEX2 v1.2 (O. V. Dolomanov, J. Appl. Cryst., 2009, 42, 339-341)	
Measurement and Refinement Details:	-	

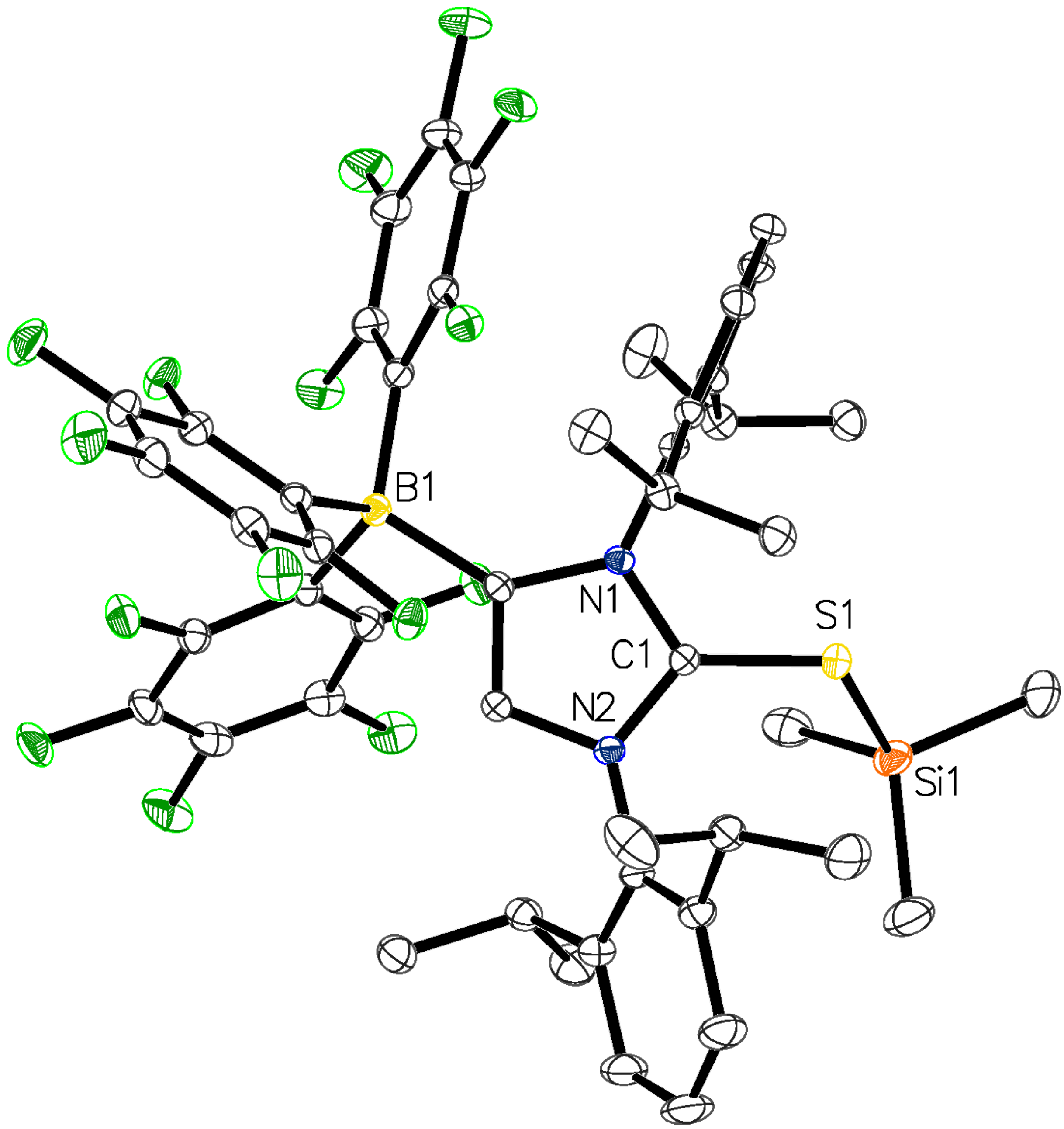

Figure S1. Molecular structure of **3** with thermal displacement parameters drawn at 50% probability; hydrogen atoms are omitted for clarity.

S2.2 (WCA-IDipp)$SeSiMe_3$·*n*-hexane (**4**·*n*-hexane)

Table S2. Crystallographic data for compound **4**·*n*-hexane.

Compound	**4**·*n*-hexane	
Identification code	2122424	
Empirical formula	$C_{54}H_{58}BF_{15}N_2SeSi$	
Formula weight	1137.88	
Temperature	100(2) K	
Wavelength	0.71073 Å	
Instrument (scan mode)	XtaLAB Synergy, Single source at offset/far, HyPix ([illegible] scan)	
Crystal system	Monoclinic	
Space group	$P2_1/c$	
Unit cell dimensions	a = 13.6820(2) Å	α = 90°
	b = 19.1111(3) Å	β = 96.165(2)°
	c = 20.2614(4) Å	γ = 90°
Volume	5267.27(15) $Å^3$	
Z	4	
Density (calculated)	1.435 Mg/m^3	
Absorption coefficient	0.827 mm^{-1}	
F(000)	2336	
Crystal habitus	irregular (colourless)	
Crystal size	0.174 x 0.095 x 0.048 mm^3	
Theta range for data collection	2.359 to 28.282°	
Index ranges	-18<=h<=18, -25<=k<=25, -27<=l<=27	
Reflections collected	261960	
Independent reflections	13066 [R(int) = 0.0962]	
Completeness to theta = 25.242°	99.9 %	
Absorption correction	Gaussian	
Max. and min. transmission	1.000 and 0.679	
Refinement method	Full-matrix least-squares on F^2	
Data / restraints / parameters	13066 / 11 / 758	
Goodness-of-fit on F^2	1.028	
Final R indices [I>2sigma(I)]	R1 = 0.0513, wR2 = 0.1150	
R indices (all data)	R1 = 0.0711, wR2 = 0.1234	
Largest diff. peak and hole	1.298 and -0.513 e.$Å^{-3}$	
Crystallisation Details:	Solid WCA-IDipp-$SeSiMe_3$ with residual amount of toluene was treated with nhexane, upon which the mixture dissolved. After 5 min at ambient temperatures, smal crystals fromed, which were grown by storing at -30°C for 3d.	
Solution	SHELXT-2014/5 (G. M. Sheldrick, Acta Cryst., 2015, A71, 3-8)	
Refinement	SHELXL-2018/3 (G. M. Sheldrick, Acta Cryst., 2008, A64, 112-122)	
Interface	OLEX2 v1.2 (O. V. Dolomanov, J. Appl. Cryst., 2009, 42, 339-341)	
Measurement and Refinement Details:	One nhexane molecule is disordered over two positions and was refined as such. One isopropyl group in one Dipp substituent is disordered over two positions and was refined as such.	

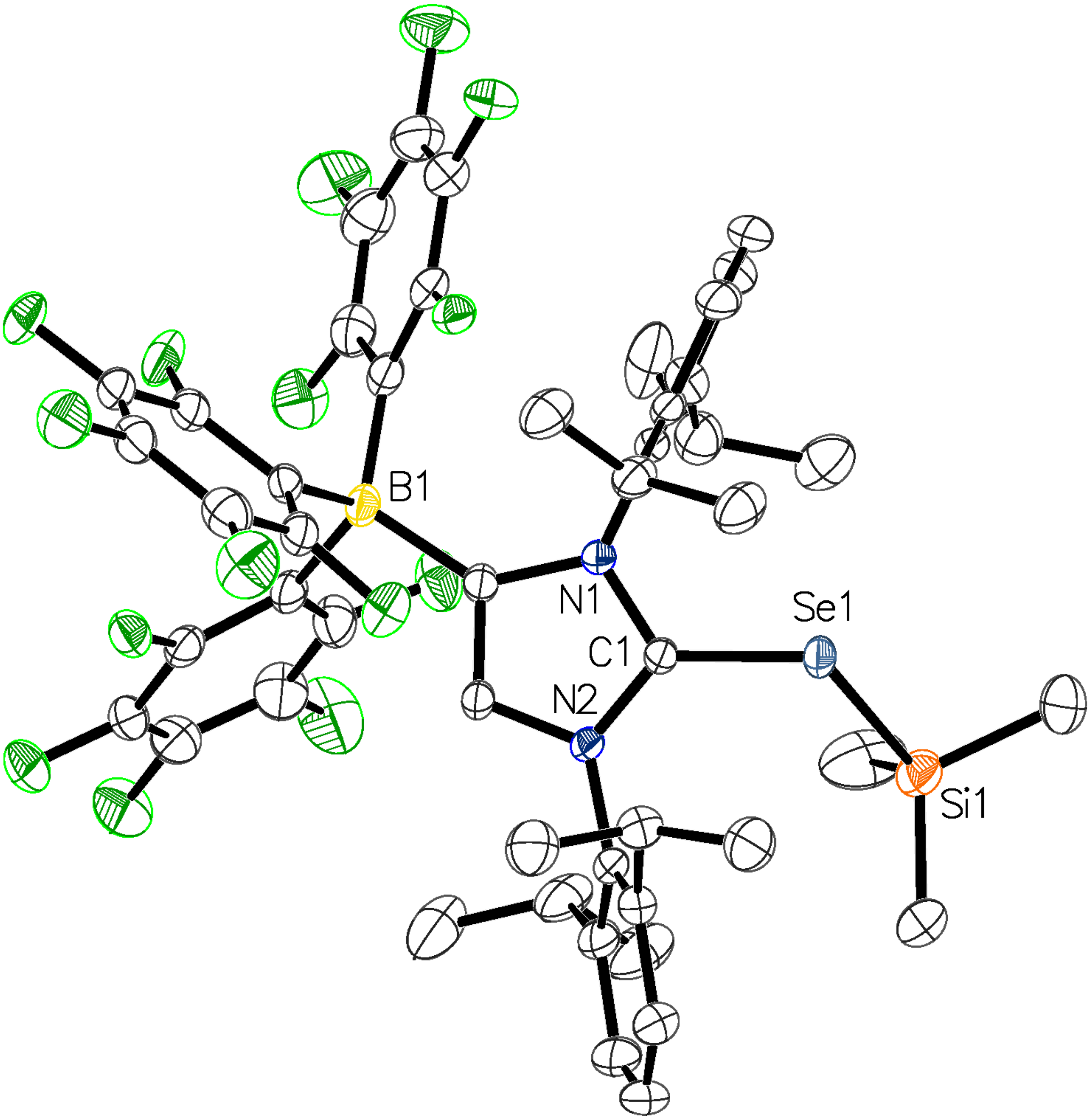

Figure S2. Molecular structure of **4** in **4**·*n*-hexane with thermal displacement parameters drawn at 50% probability; hydrogen atoms and non-coordinating solvent molecules are omitted for clarity.

S2.3 (WCA-IDipp)Se$(CH_2)_4$OSiMe_3·toluene (**4A**·toluene)

Table S3. Crystallographic data for compound **4A**·toluene.

Compound	**4A**·toluene	
Identification code	2122410	
Empirical formula	$C_{55.5}H_{56}BF_{15}N_2OSeSi$	
Formula weight	1169.88	
Temperature	100(2) K	
Wavelength	0.71073 Å	
Instrument (scan mode)	XtaLAB Synergy, Single source at offset/far, HyPix ([illegible] scan)	
Crystal system	Monoclinic	
Space group	$P2_1/c$	
Unit cell dimensions	a = 10.7325(2) Å	α = 90°
	b = 20.1732(4) Å	β = 101.809(2)°
	c = 25.4527(7) Å	γ = 90°
Volume	5394.1(2) $Å^3$	
Z	4	
Density (calculated)	1.441 Mg/m^3	
Absorption coefficient	0.812 mm^{-1}	
F(000)	2396	
Crystal habitus	block (colourless)	
Crystal size	0.438 x 0.370 x 0.149 mm^3	
Theta range for data collection	2.469 to 32.572°	
Index ranges	-14<=h<=15, -28<=k<=26, -34<=l<=37	
Reflections collected	84840	
Independent reflections	16608 [R(int) = 0.0521]	
Completeness to theta = 25.242°	99.9 %	
Absorption correction	Gaussian	
Max. and min. transmission	1.000 and 0.403	
Refinement method	Full-matrix least-squares on F^2	
Data / restraints / parameters	16608 / 134 / 733	
Goodness-of-fit on F^2	1.013	
Final R indices [I>2sigma(I)]	R1 = 0.0602, wR2 = 0.1313	
R indices (all data)	R1 = 0.1035, wR2 = 0.1481	
Largest diff. peak and hole	1.568 and -0.977 e.$Å^{-3}$	
Crystallisation Details:	A saturated solution of WCA-IDipp-Se$(CH_2)_4$OSiMe_3 in toluene/hexane (2:1 mix) was stored at -40C for several days.	
Solution	SHELXT-2014/5 (G. M. Sheldrick, Acta Cryst., 2015, A71, 3-8)	
Refinement	SHELXL-2018/3 (G. M. Sheldrick, Acta Cryst., 2008, A64, 112-122)	
Interface	OLEX2 v1.2 (O. V. Dolomanov, J. Appl. Cryst., 2009, 42, 339-341)	
Measurement and Refinement Details:	One toluene molecule is located on a special position, hence the non integer nomber of carbon atoms. ISOR restraints were applied to the carbon atoms from the activated thf bound to the Se atom.	

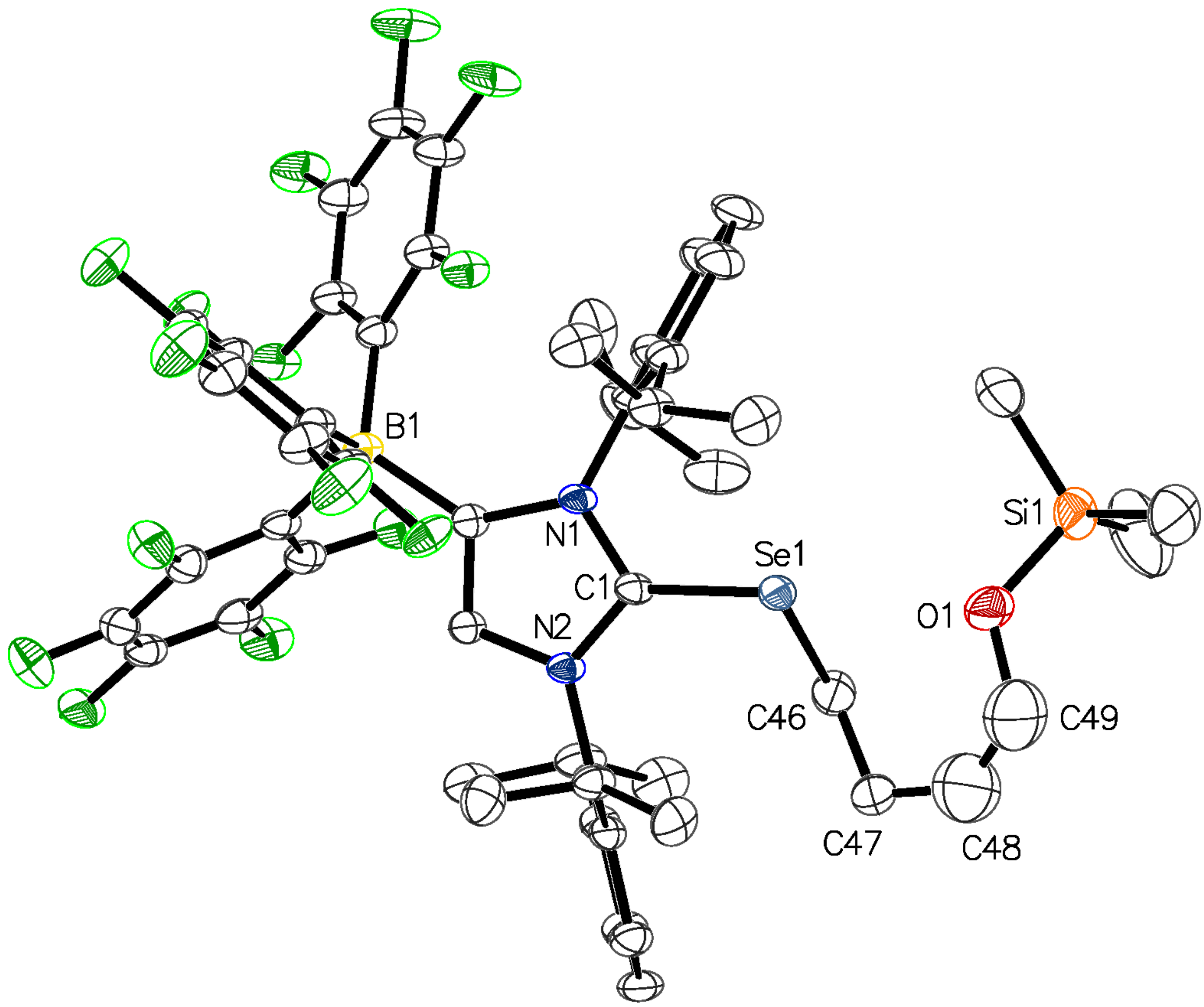

Figure S3. Molecular structure of **4A** in **4A**·toluene with thermal displacement parameters drawn at 50% probability; hydrogen atoms and non-coordinating solvent molecules are omitted for clarity.

S2.4 [{(WCA-IDipp)S}RhCl(η^5-C_5Me_5)] (**5**·2(benzene))

Table S4. Crystallographic data for compound **5**·2(benzene).

Compound	**5**·2(benzene)	
Identification code	2122417	
Empirical formula	$C_{67}H_{62}BClF_{15}N_2RhS$	
Formula weight	1361.41	
Temperature	100(2) K	
Wavelength	0.71073 Å	
Instrument (scan mode)	XtaLAB Synergy, Single source at offset/far, HyPix (⍰ scan)	
Crystal system	Triclinic	
Space group	*P*-1	
Unit cell dimensions	a = 14.1658(4) Å	α = 102.975(4)°
	b = 16.1810(6) Å	β = 114.333(4)°
	c = 16.2049(8) Å	γ = 97.844(4)°
Volume	3186.5(2) Å^3	
Z	2	
Density (calculated)	1.419 Mg/m^3	
Absorption coefficient	0.429 mm^{-1}	
F(000)	1392	
Crystal habitus	plate (brown)	
Crystal size	0.174 x 0.087 x 0.034 mm^3	
Theta range for data collection	2.530 to 32.672°	
Index ranges	-21<=h<=20, -23<=k<=23, -21<=l<=22	
Reflections collected	86259	
Independent reflections	19561 [R(int) = 0.0410]	
Completeness to theta = 25.242°	99.8 %	
Absorption correction	Gaussian	
Max. and min. transmission	1.000 and 0.592	
Refinement method	Full-matrix least-squares on F^2	
Data / restraints / parameters	19561 / 272 / 836	
Goodness-of-fit on F^2	1.072	
Final R indices [I>2sigma(I)]	R1 = 0.0485, wR2 = 0.1315	
R indices (all data)	R1 = 0.0640, wR2 = 0.1380	
Largest diff. peak and hole	1.323 and -0.637 e.Å^{-3}	
Crystallisation Details:	A saturated solution of WCA-IDipp-SRh(COD)Cl in benzene was layered with nhexane at ambient temperatures under inert conditions.	
Solution	SHELXT-2014/5 (G. M. Sheldrick, Acta Cryst., 2015, A71, 3-8)	
Refinement	SHELXL-2018/3 (G. M. Sheldrick, Acta Cryst., 2008, A64, 112-122)	
Interface	OLEX2 v1.2 (O. V. Dolomanov, J. Appl. Cryst., 2009, 42, 339-341)	
Measurement and Refinement Details:	Two benzene molecules are disordered over a special position and were refined as such. Several restraints were applied. Several reflections with error/esds > 10 were omitted.	

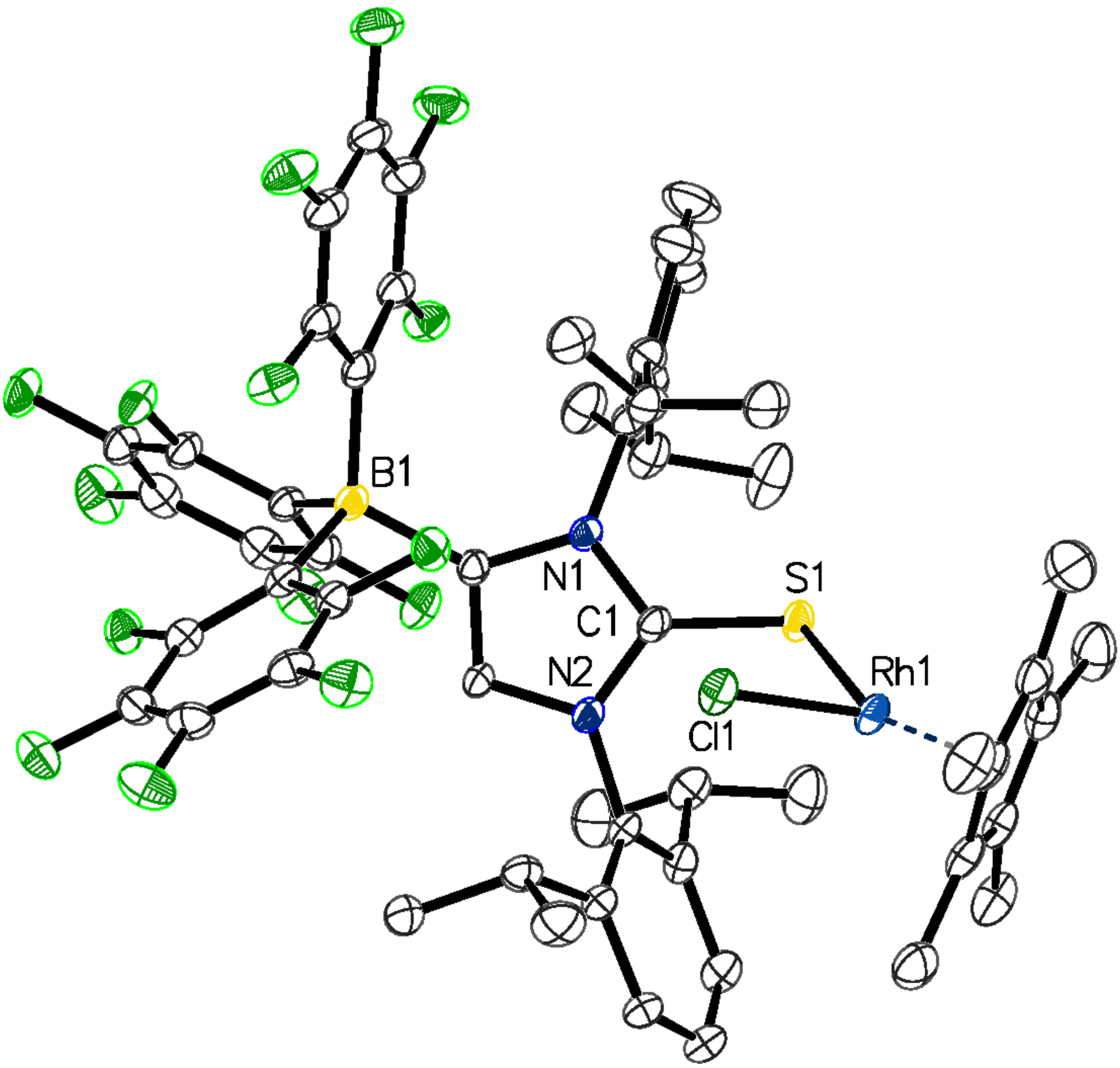

Figure S4. Molecular structure of **5** in **5**·2(benzene) with thermal displacement parameters drawn at 50% probability; hydrogen atoms and non-coordinating solvent molecules are omitted for clarity.

S2.5 [{(WCA-IDipp)S}IrCl(η^5-C_5Me_5)] (**6**·1.5(CH_2Cl_2))

Table S5. Crystallographic data for compound **6**·1.5(CH_2Cl_2).

Compound	**6**·1.5(CH_2Cl_2)	
Identification code	2122414	
Empirical formula	$C_{56.50}H_{53}BCl_4F_{15}IrN_2S$	
Formula weight	1421.88	
Temperature	100(2) K	
Wavelength	0.71073 Å	
Instrument (scan mode)	XtaLAB Synergy, Single source at offset/far, HyPix (w scan)	
Crystal system	Triclinic	
Space group	*P*-1	
Unit cell dimensions	a = 13.7550(2) Å	α = 68.712(2)°
	b = 14.1878(2) Å	β = 83.819(2)°
	c = 16.8904(2) Å	γ = 83.726(2)°
Volume	3044.67(8) $Å^3$	
Z	2	
Density (calculated)	1.551 Mg/m^3	
Absorption coefficient	2.486 mm^{-1}	
F(000)	1414	
Crystal habitus	plate (orange)	
Crystal size	0.223 x 0.153 x 0.043 mm^3	
Theta range for data collection	1.493 to 28.282°	
Index ranges	-18<=h<=18, -18<=k<=18, -22<=l<=22	
Reflections collected	354987	
Independent reflections	15108 [R(int) = 0.0586]	
Completeness to theta = 25.242°	99.8 %	
Absorption correction	Gaussian	
Max. and min. transmission	1.000 and 0.387	
Refinement method	Full-matrix least-squares on F^2	
Data / restraints / parameters	15108 / 260 / 866	
Goodness-of-fit on F^2	1.083	
Final R indices [I>2sigma(I)]	R1 = 0.0353, wR2 = 0.0961	
R indices (all data)	R1 = 0.0378, wR2 = 0.0975	
Largest diff. peak and hole	2.430 and -1.464 e.$Å^{-3}$	
Crystallisation Details:	A saturated solution of WCA-IDipp-SIrCp*Cl in DCM was layered with *n*-hexane at room temperature under inert conditions.	
Solution	SHELXT-2014/5 (G. M. Sheldrick, Acta Cryst., 2015, A71, 3-8)	
Refinement	SHELXL-2018/3 (G. M. Sheldrick, Acta Cryst., 2008, A64, 112-122)	
Interface	OLEX2 v1.2 (O. V. Dolomanov, J. Appl. Cryst., 2009, 42, 339-341)	
Measurement and Refinement Details:	One and one half of a DCM molecules are disordered over several positions and were refined as such. Several Restraints were applied. The half a molecule DCM results in the non-integer number of carbon atoms in the sum formula.<br>Several reflections with an error/esd > 8 were omitted.	

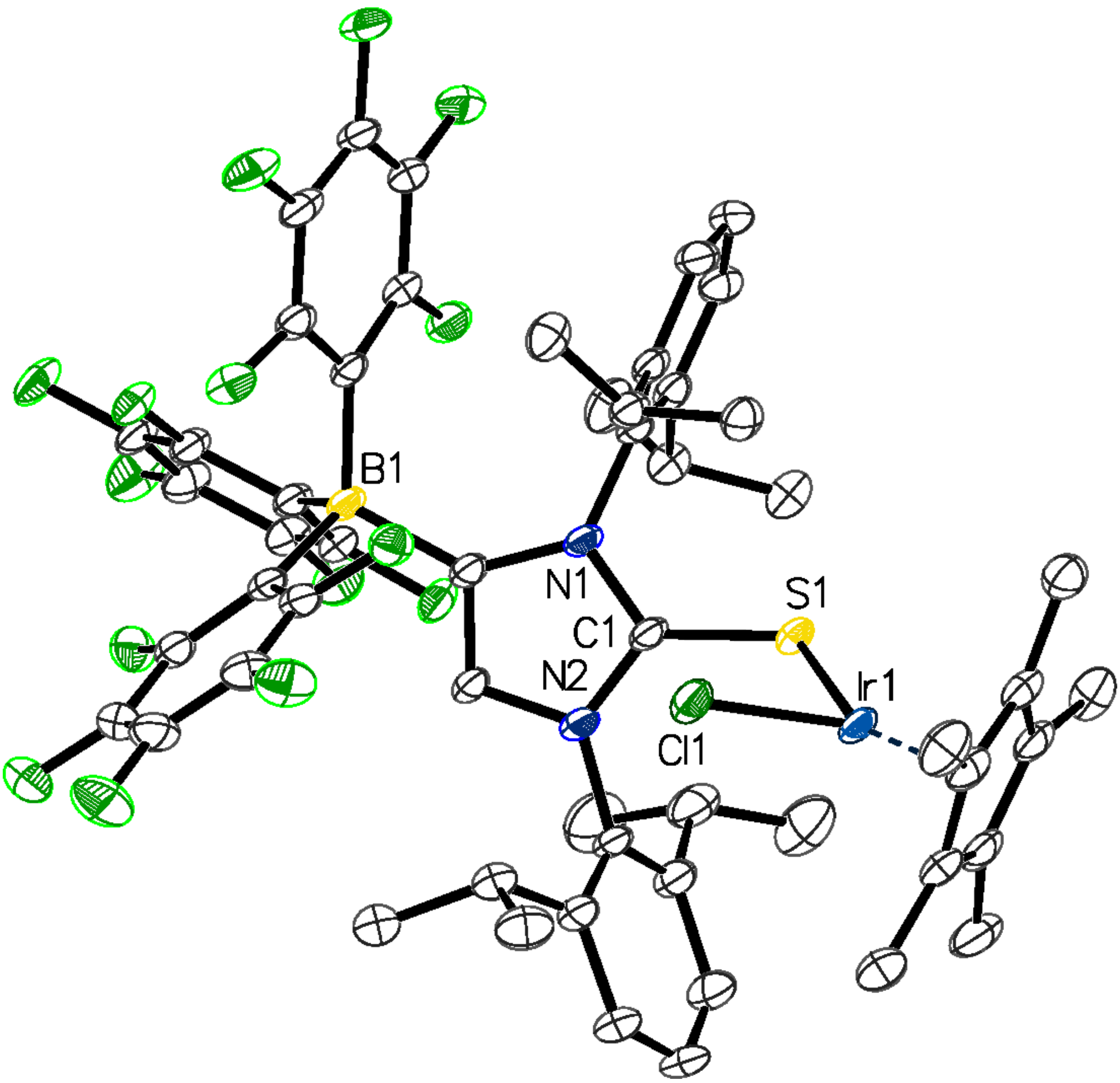

Figure S5. Molecular structure of **6** in **6**·1.5(CH_2Cl_2) with thermal displacement parameters drawn at 50% probability; hydrogen atoms and non-coordinating solvent molecules are omitted for clarity.

S2.6 [{(WCA-IDipp)Se}RhCl(η^5-C_5Me_5)] (**7**·(benzene)0.5(n-hexane))

Table S6. Crystallographic data for compound **7**·(benzene)0.5(n-hexane).

Compound	**7**·(benzene)0.5(n-hexane)	
Identification code	2122418	
Empirical formula	$C_{64}H_{63}BClF_{15}N_2RhSe$	
Formula weight	1373.29	
Temperature	100(2) K	
Wavelength	0.71073 Å	
Instrument (scan mode)	XtaLAB Synergy, Single source at offset/far, HyPix ([illegible] scan)	
Crystal system	Triclinic	
Space group	*P*-1	
Unit cell dimensions	a = 12.0093(2) Å	α = 70.351(2)°
	b = 15.4940(4) Å	β = 77.977(2)°
	c = 17.8801(4) Å	γ = 75.103(2)°
Volume	3000.68(12) $Å^3$	
Z	2	
Density (calculated)	1.520 Mg/m^3	
Absorption coefficient	1.025 mm^{-1}	
F(000)	1394	
Crystal habitus	plate (orange)	
Crystal size	0.130 x 0.081 x 0.035 mm^3	
Theta range for data collection	2.687 to 32.818°	
Index ranges	-17<=h<=18, -22<=k<=23, -26<=l<=26	
Reflections collected	157923	
Independent reflections	19625 [R(int) = 0.0577]	
Completeness to theta = 25.242°	99.9 %	
Absorption correction	Gaussian	
Max. and min. transmission	1.000 and 0.816	
Refinement method	Full-matrix least-squares on F^2	
Data / restraints / parameters	19625 / 0 / 831	
Goodness-of-fit on F^2	1.028	
Final R indices [I>2sigma(I)]	R1 = 0.0358, wR2 = 0.0733	
R indices (all data)	R1 = 0.0574, wR2 = 0.0785	
Largest diff. peak and hole	1.295 and -0.629 e.$Å^{-3}$	
Crystallisation Details:	A saturated solution of WCA-IDipp-SeRhCp*Cl in benzene was layered with nhexane at ambient temperatures under inert conditions.	
Solution	SHELXT-2014/5 (G. M. Sheldrick, Acta Cryst., 2015, A71, 3-8)	
Refinement	SHELXL-2018/3 (G. M. Sheldrick, Acta Cryst., 2008, A64, 112-122)	
Interface	OLEX2 v1.2 (O. V. Dolomanov, J. Appl. Cryst., 2009, 42, 339-341)	
Measurement and Refinement Details:	One molecule of benzene is disordered and was refined over two positions. One nhexane molecule is located on a special position.	

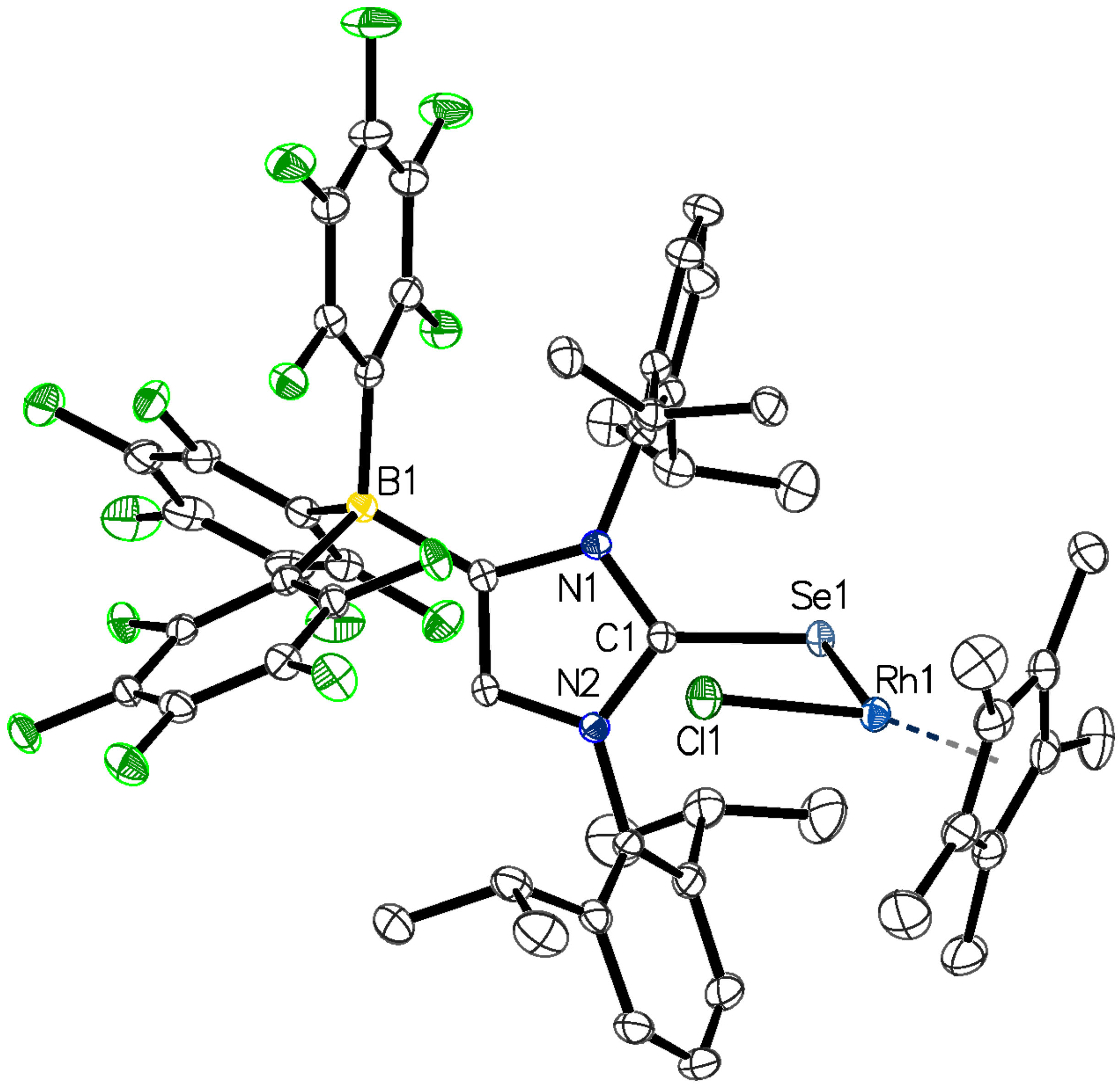

Figure S6. Molecular structure of **7** in **7**·(benzene)0.5(n-hexane) with thermal displacement parameters drawn at 50% probability; hydrogen atoms and non-coordinating solvent molecules are omitted for clarity.

S2.6 [{(WCA-IDipp)Se}IrCl(η^5-C_5Me_5)] (**8**·CH_2Cl_2)

Table S7. Crystallographic data for compound **8**·CH_2Cl_2.

Compound	**8**·CH_2Cl_2	
Identification code	2122415	
Empirical formula	$C_{56}H_{52}BCl_3F_{15}IrN_2Se$	
Formula weight	1426.31	
Temperature	100(2) K	
Wavelength	0.71073 Å	
Instrument (scan mode)	XtaLAB Synergy, Single source at offset/far, HyPix (w scan)	
Crystal system	Triclinic	
Space group	*P*-1	
Unit cell dimensions	a = 11.7529(2) Å	α = 81.168(2)°
	b = 14.2417(4) Å	β = 86.014(2)°
	c = 16.6275(4) Å	γ = 86.473(2)°
Volume	2739.83(11) $Å^3$	
Z	2	
Density (calculated)	1.729 Mg/m^3	
Absorption coefficient	3.339 mm^{-1}	
F(000)	1408	
Crystal habitus	plate (orange)	
Crystal size	0.198 x 0.069 x 0.019 mm^3	
Theta range for data collection	1.449 to 28.282°	
Index ranges	-15<=h<=15, -17<=k<=18, -22<=l<=22	
Reflections collected	148329	
Independent reflections	13585 [R(int) = 0.0634]	
Completeness to theta = 25.242°	100.0 %	
Absorption correction	Gaussian	
Max. and min. transmission	1.000 and 0.480	
Refinement method	Full-matrix least-squares on F^2	
Data / restraints / parameters	13585 / 0 / 725	
Goodness-of-fit on F^2	1.042	
Final R indices [I>2sigma(I)]	R1 = 0.0283, wR2 = 0.0635	
R indices (all data)	R1 = 0.0345, wR2 = 0.0653	
Largest diff. peak and hole	1.740 and -0.925 e.$Å^{-3}$	
Crystallisation Details:	A saturated solution of WCA-IDipp-Se-IrCp*Cl in DCM was layered with *n*-hexane at room temperature under inert conditions.	
Solution	SHELXT-2014/5 (G. M. Sheldrick, Acta Cryst., 2015, A71, 3-8)	
Refinement	SHELXL-2018/3 (G. M. Sheldrick, Acta Cryst., 2008, A64, 112-122)	
Interface	OLEX2 v1.2 (O. V. Dolomanov, J. Appl. Cryst., 2009, 42, 339-341)	
Measurement and Refinement Details:	-	

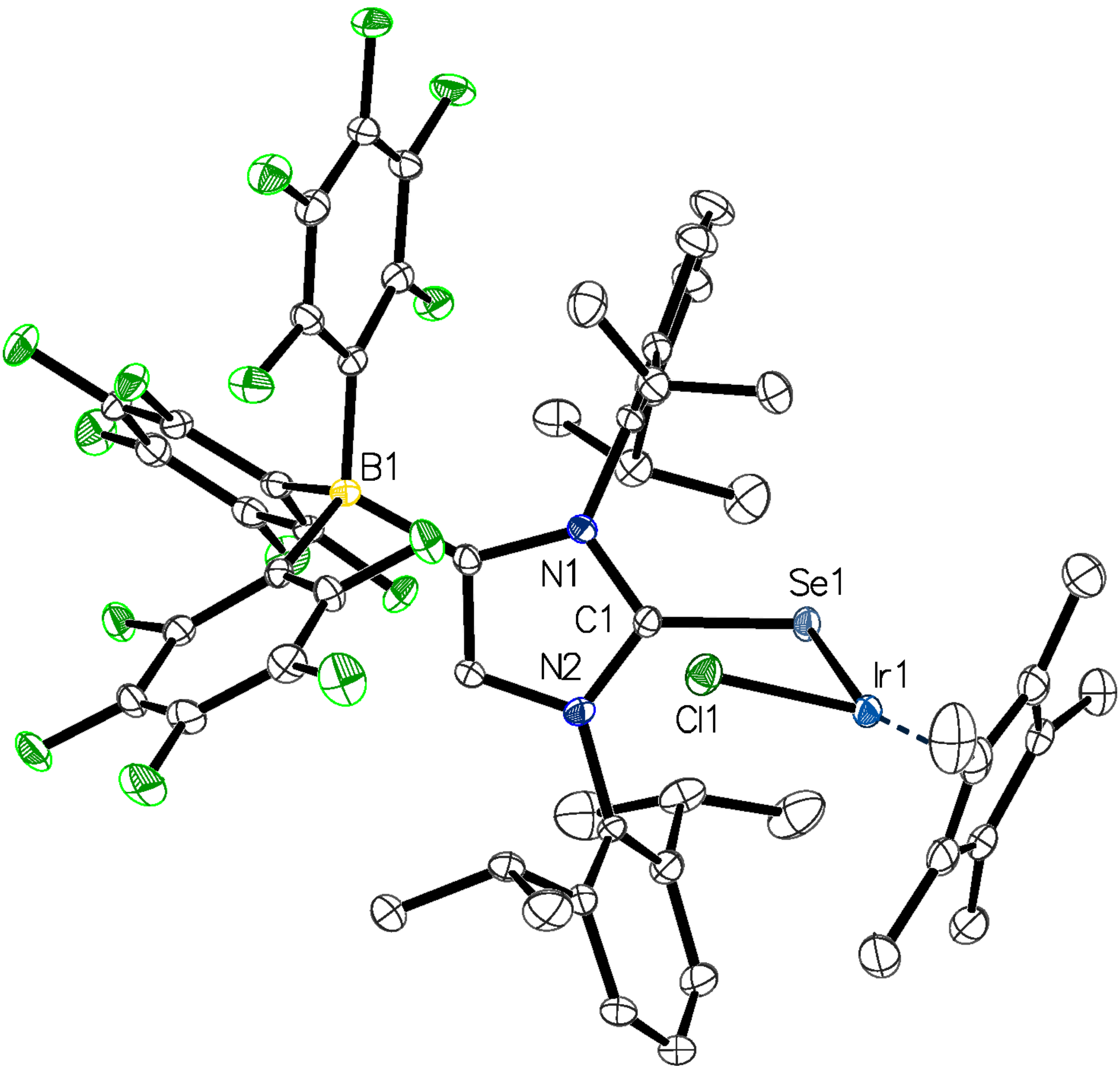

Figure S7. Molecular structure of **8** in **8**·CH_2Cl_2 with thermal displacement parameters drawn at 50% probability; hydrogen atoms and non-coordinating solvent molecules are omitted for clarity.

S2.4 [{(WCA-IDipp)S}Rh(COD)] (**9**)

Table S8. Crystallographic data for compound **9**.

Compound	**9**	
Identification code	2122421	
Empirical formula	$C_{53}H_{47}BF_{15}N_2RhS$	
Formula weight	1142.70	
Temperature	100(2) K	
Wavelength	0.71073 Å	
Instrument (scan mode)	XtaLAB Synergy, Single source at offset/far, HyPix (? scan)	
Crystal system	Monoclinic	
Space group	$P2_1$	
Unit cell dimensions	a = 10.7102(2) Å	α = 90°
	b = 19.9478(6) Å	β = 96.430(2)°
	c = 11.2918(4) Å	γ = 90°
Volume	2397.26(12) Å^3	
Z	2	
Density (calculated)	1.583 Mg/m^3	
Absorption coefficient	0.499 mm^{-1}	
F(000)	1160	
Crystal habitus	plate (orange)	
Crystal size	0.261 x 0.115 x 0.043 mm^3	
Theta range for data collection	2.687 to 28.280°	
Index ranges	-14<=h<=14, -26<=k<=26, -15<=l<=15	
Reflections collected	111863	
Independent reflections	11875 [R(int) = 0.0666]	
Completeness to theta = 25.242°	99.9 %	
Absorption correction	Gaussian	
Max. and min. transmission	1.000 and 0.661	
Refinement method	Full-matrix least-squares on F^2	
Data / restraints / parameters	11875 / 1 / 666	
Goodness-of-fit on F^2	1.050	
Final R indices [I>2sigma(I)]	R1 = 0.0263, wR2 = 0.0637	
R indices (all data)	R1 = 0.0283, wR2 = 0.0644	
Absolute structure parameter	-0.022(7)	
Largest diff. peak and hole	0.774 and -0.364 e.Å^{-3}	
Crystallisation Details:	A saturated solution of WCA-IDipp-SRh(COD) in benzene was layered with nhexane at ambient temperatures under inert conditions.	
Solution	SHELXT-2014/5 (G. M. Sheldrick, Acta Cryst., 2015, A71, 3-8)	
Refinement	SHELXL-2018/3 (G. M. Sheldrick, Acta Cryst., 2008, A64, 112-122)	
Interface	OLEX2 v1.2 (O. V. Dolomanov, J. Appl. Cryst., 2009, 42, 339-341)	
Measurement and Refinement Details:	-	

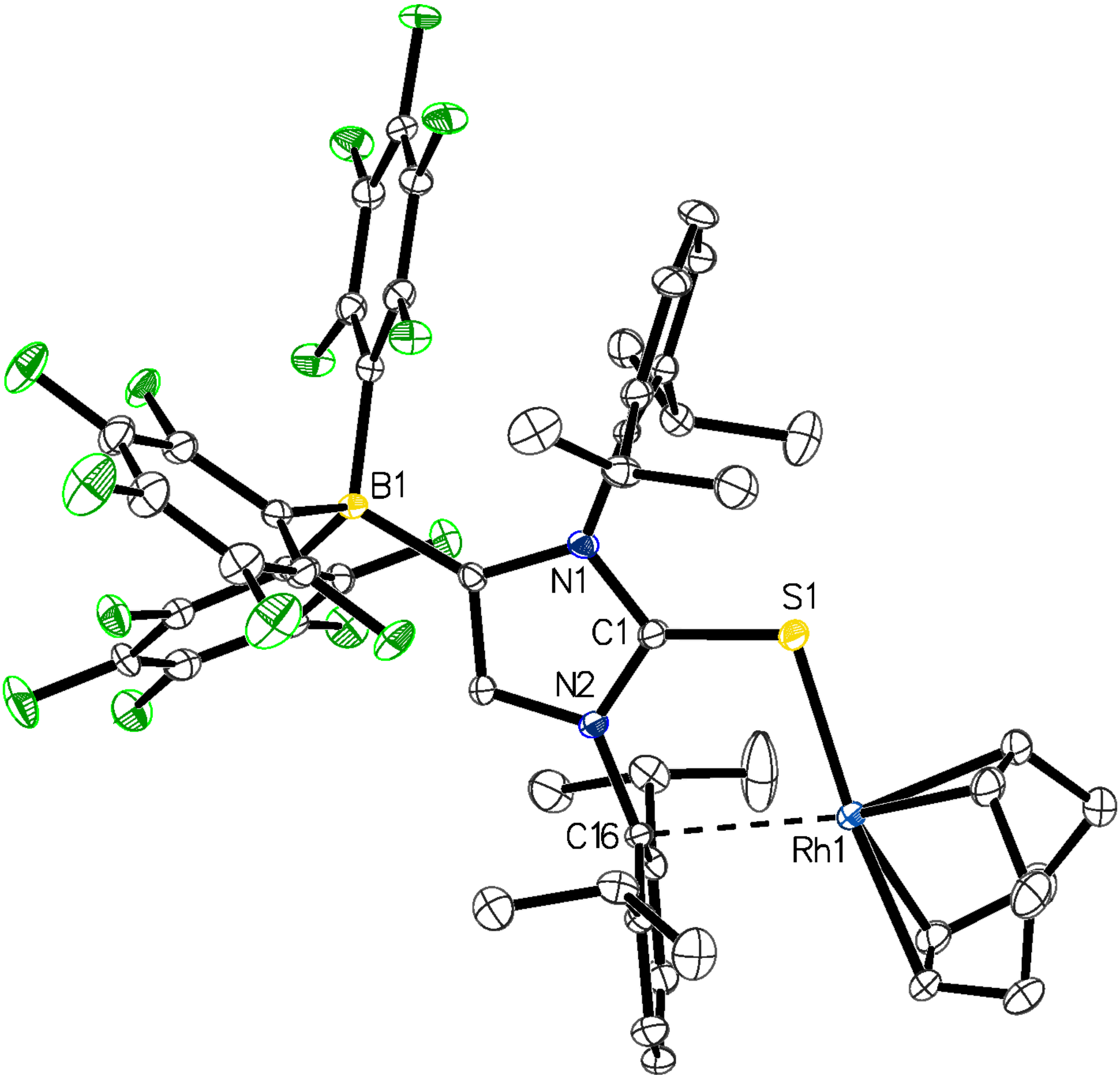

Figure S8. Molecular structure of **9** with thermal displacement parameters drawn at 50% probability; hydrogen atoms are omitted for clarity.

S2.5 [{(WCA-IDipp)S}Ir(COD)]·1.5toluene·0.5*n*-hexane (**10**·1.5(toluene)0.5(*n*-hexane))

Table S9. Crystallographic data for compound **10**·1.5toluene·0.5*n*-hexane.

Compound	**10**·1.5(toluene)0.5(*n*-hexane)	
Identification code	2122413	
Empirical formula	$C_{130}H_{125}B_2F_{30}Ir_2N_4S_2$	
Formula weight	2783.47	
Temperature	100(2) K	
Wavelength	0.71073 Å	
Instrument (scan mode)	XtaLAB Synergy, Single source at offset/far, HyPix ([illegible] scan)	
Crystal system	Triclinic	
Space group	*P*-1	
Unit cell dimensions	a = 15.6690(2) Å	α = 69.093(2)°
	b = 20.3927(2) Å	β = 82.371(2)°
	c = 20.6018(4) Å	γ = 89.874(2)°
Volume	6087.85(17) Å^3	
Z	2	
Density (calculated)	1.518 Mg/m^3	
Absorption coefficient	2.315 mm^{-1}	
F(000)	2798	
Crystal habitus	irregular (orange)	
Crystal size	0.403 x 0.337 x 0.165 mm^3	
Theta range for data collection	2.434 to 30.508°	
Index ranges	-22<=h<=22, -29<=k<=29, -29<=l<=29	
Reflections collected	459097	
Independent reflections	37130 [R(int) = 0.0439]	
Completeness to theta = 25.242°	99.9 %	
Absorption correction	Gaussian	
Max. and min. transmission	1.000 and 0.291	
Refinement method	Full-matrix least-squares on F^2	
Data / restraints / parameters	37130 / 373 / 1621	
Goodness-of-fit on F^2	1.113	
Final R indices [I>2sigma(I)]	R1 = 0.0384, wR2 = 0.0879	
R indices (all data)	R1 = 0.0452, wR2 = 0.0903	
Largest diff. peak and hole	3.522 and -2.178 e.Å^{-3}	
Crystallisation Details:	A saturated solution of WCA-IDipp-S-Ir(COD) in toluene was layered with nhexane at ambient temperatures under inert conditions.	
Solution	SHELXT-2014/5 (G. M. Sheldrick, Acta Cryst., 2015, A71, 3-8)	
Refinement	SHELXL-2018/3 (G. M. Sheldrick, Acta Cryst., 2008, A64, 112-122)	
Interface	OLEX2 v1.2 (O. V. Dolomanov, J. Appl. Cryst., 2009, 42, 339-341)	
Measurement and Refinement Details:	Two molecules of toluene are each disordered over two positions and were refined as such. To one of these disordered toluene molecule EADP was applied. To both disordered toluene molecules afix 66 and 65 were applied to the six membered ring. To all these carbon atoms ISOR restraints were applied. DFIX and SADI restraints were applied to one methyl group. Two toluene molecules are each disordered and located over a special postion and were refined as such. ISOR restraints were applied to all these carbon atoms and to the six membered rings afix 66 and 65 were applied. DFIX and SADI restraints were applied to one methyl group. One nhexane molecule is disordered and located over a special position and was refined as such. ISOR and SADI restraints were applied. Some reflections were omitted because of a error/esd above 10.	

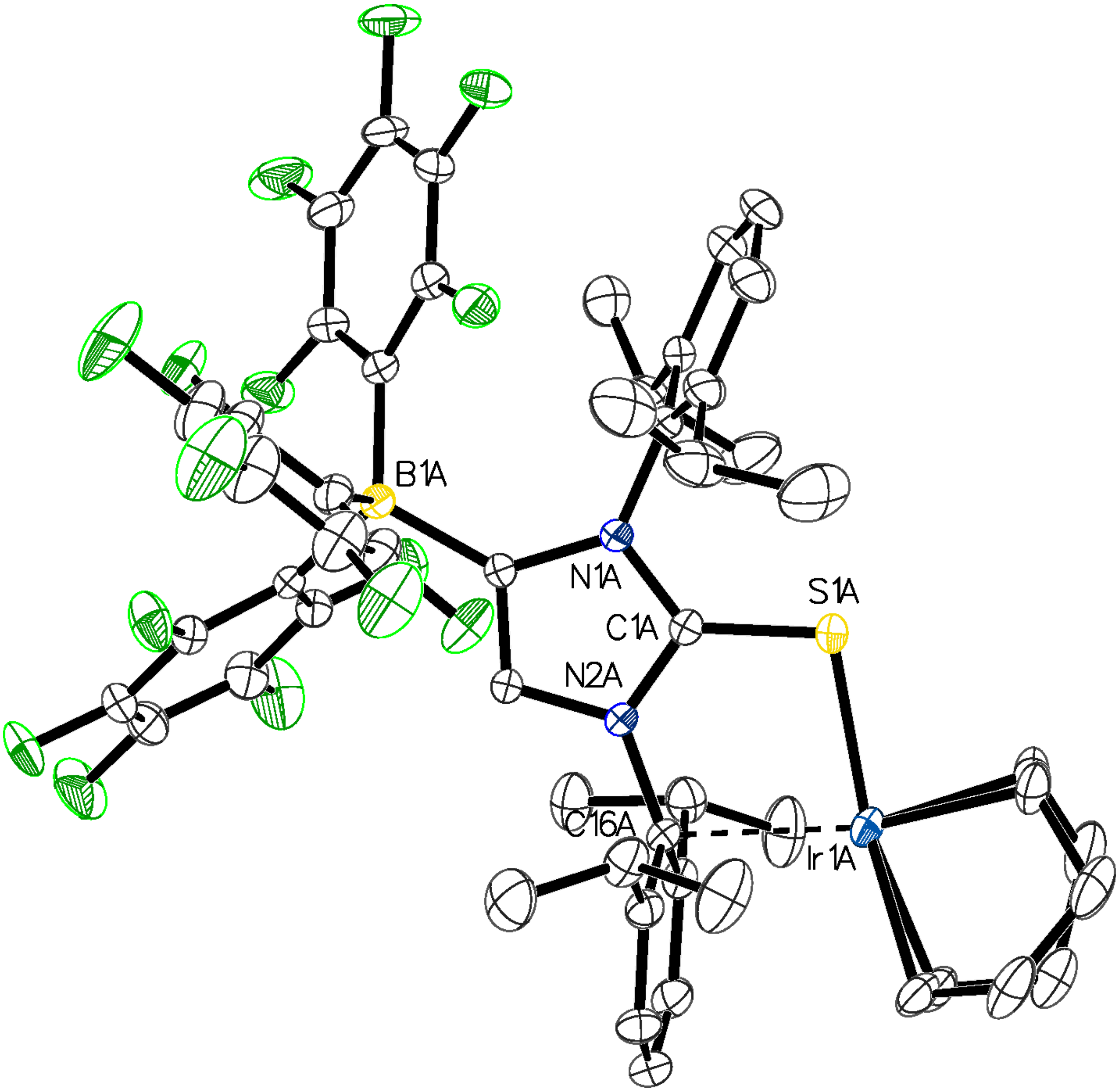

Figure S9. Molecular structure of **10** in **10**·1.5(toluene)0.5(*n*-hexane) with thermal displacement parameters drawn at 50% probability; hydrogen atoms, non-coordinating solvent molecules and the second molecule in the asymmetric unit are omitted for clarity.

S2.6 [{(WCA-IDipp)Se}Rh(COD)] (**11**)

Table S10. Crystallographic data for compound **11**.

Compound	**11**	
Identification code	2122420	
Empirical formula	$C_{53}H_{47}BF_{15}N_2RhSe$	
Formula weight	1189.60	
Temperature	100(2) K	
Wavelength	1.54184 Å	
Instrument (scan mode)	XtaLAB Synergy, Single source at home/near, HyPix ([illegible] scan)	
Crystal system	Monoclinic	
Space group	$P2_1/c$	
Unit cell dimensions	a = 12.5034(2) Å	α = 90°
	b = 18.3334(2) Å	β = 102.567(2)°
	c = 21.6822(4) Å	γ = 90°
Volume	4851.13(13) $Å^3$	
Z	4	
Density (calculated)	1.629 Mg/m^3	
Absorption coefficient	4.572 mm^{-1}	
F(000)	2392	
Crystal habitus	plate (yellow)	
Crystal size	0.149 x 0.075 x 0.020 mm^3	
Theta range for data collection	3.189 to 78.164°	
Index ranges	-15<=h<=15, -20<=k<=23, -27<=l<=26	
Reflections collected	99587	
Independent reflections	10235 [R(int) = 0.0658]	
Completeness to theta = 67.684°	100.0 %	
Absorption correction	Gaussian	
Max. and min. transmission	1.000 and 0.550	
Refinement method	Full-matrix least-squares on F^2	
Data / restraints / parameters	10235 / 0 / 666	
Goodness-of-fit on F^2	1.051	
Final R indices [I>2sigma(I)]	R1 = 0.0394, wR2 = 0.1204	
R indices (all data)	R1 = 0.0428, wR2 = 0.1242	
Largest diff. peak and hole	0.918 and -1.252 e.$Å^{-3}$	
Crystallisation Details:	A saturated solution of WCA-IDipp-SeRh(COD) in toluene was layered with nhexane at ambient temperatures under inert conditions.	
Solution	SHELXT-2014/5 (G. M. Sheldrick, Acta Cryst., 2015, A71, 3-8)	
Refinement	SHELXL-2018/3 (G. M. Sheldrick, Acta Cryst., 2008, A64, 112-122)	
Interface	OLEX2 v1.2 (O. V. Dolomanov, J. Appl. Cryst., 2009, 42, 339-341)	
Measurement and Refinement Details:	-	

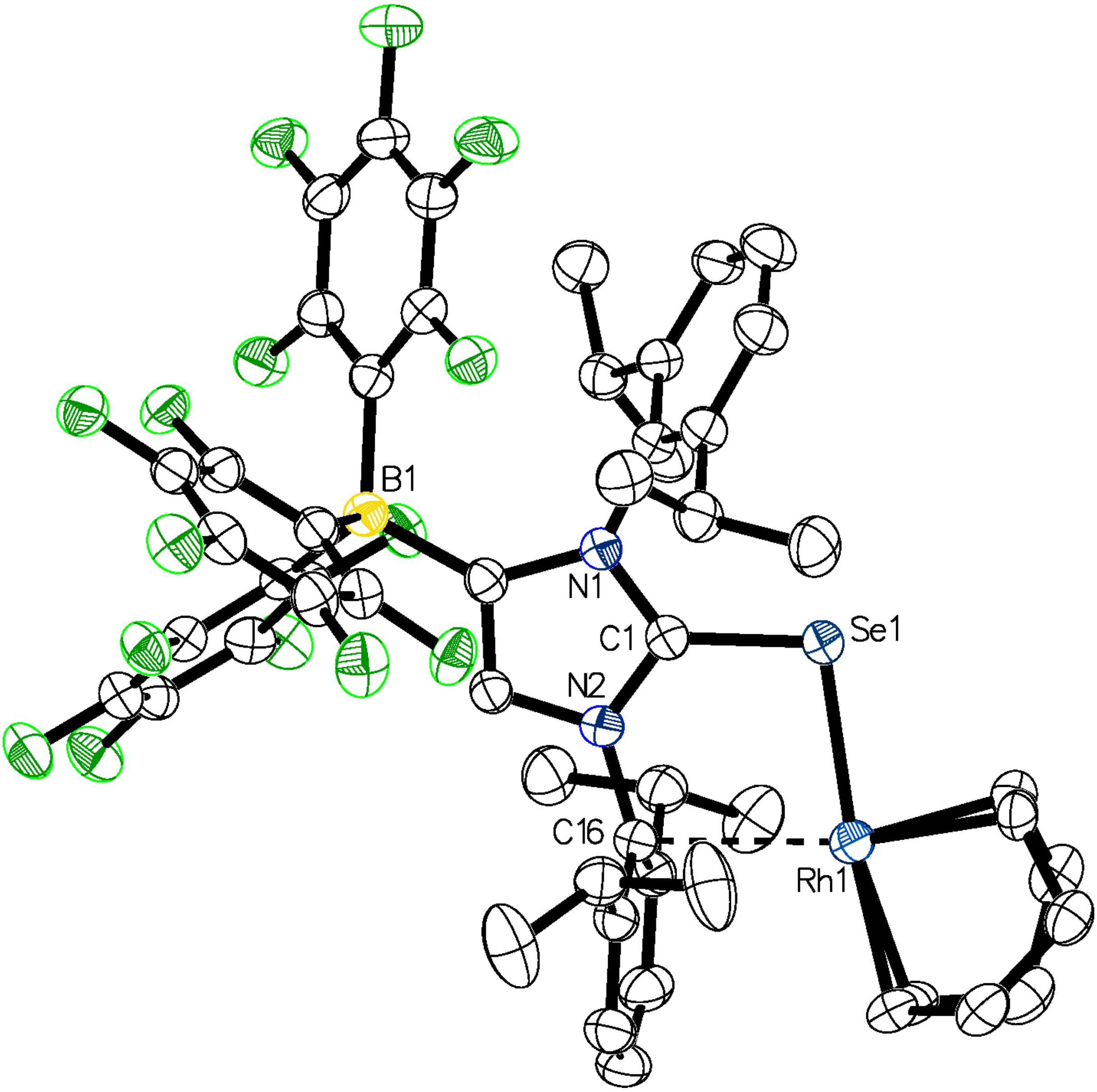

Figure S10. Molecular structure of **11** with thermal displacement parameters drawn at 50% probability; hydrogen atoms are omitted for clarity.

S2.7 [{(WCA-IDipp)Se}Ir(COD)] (**12**)

Table S11. Crystallographic data for compound **12**.

Compound	**12**	
Identification code	2122423	
Empirical formula	$C_{53}H_{47}BF_{15}IrN_2Se$	
Formula weight	1278.89	
Temperature	100(2) K	
Wavelength	0.71073 Å	
Instrument (scan mode)	XtaLAB Synergy, Single source at offset/far, HyPix (w scan)	
Crystal system	Monoclinic	
Space group	$P2_1/c$	
Unit cell dimensions	a = 12.6665(2) Å	α = 90°
	b = 18.1947(2) Å	β = 103.013(2)°
	c = 21.5666(2) Å	γ = 90°
Volume	4842.66(11) $Å^3$	
Z	4	
Density (calculated)	1.754 Mg/m^3	
Absorption coefficient	3.607 mm^{-1}	
F(000)	2520	
Crystal habitus	prism (orange)	
Crystal size	0.211 x 0.165 x 0.147 mm^3	
Theta range for data collection	1.480 to 30.508°	
Index ranges	-18<=h<=18, -25<=k<=25, -30<=l<=30	
Reflections collected	635841	
Independent reflections	14761 [R(int) = 0.0483]	
Completeness to theta = 25.242°	100.0 %	
Absorption correction	Gaussian	
Max. and min. transmission	1.000 and 0.297	
Refinement method	Full-matrix least-squares on F^2	
Data / restraints / parameters	14761 / 0 / 666	
Goodness-of-fit on F^2	1.088	
Final R indices [I>2sigma(I)]	R1 = 0.0269, wR2 = 0.0661	
R indices (all data)	R1 = 0.0305, wR2 = 0.0676	
Largest diff. peak and hole	2.337 and -1.262 e.$Å^{-3}$	

Crystallisation Details:	A saturated solution of WCA-IDippSeIr(COD) in toluene was layered with *n*-hexane at room temperature under inert conditions
Solution	SHELXT-2014/5 (G. M. Sheldrick, Acta Cryst., 2015, A71, 3-8)
Refinement	SHELXL-2018/3 (G. M. Sheldrick, Acta Cryst., 2008, A64, 112-122)
Interface	OLEX2 v1.2 (O. V. Dolomanov, J. Appl. Cryst., 2009, 42, 339-341)
Measurement and Refinement Details:	-

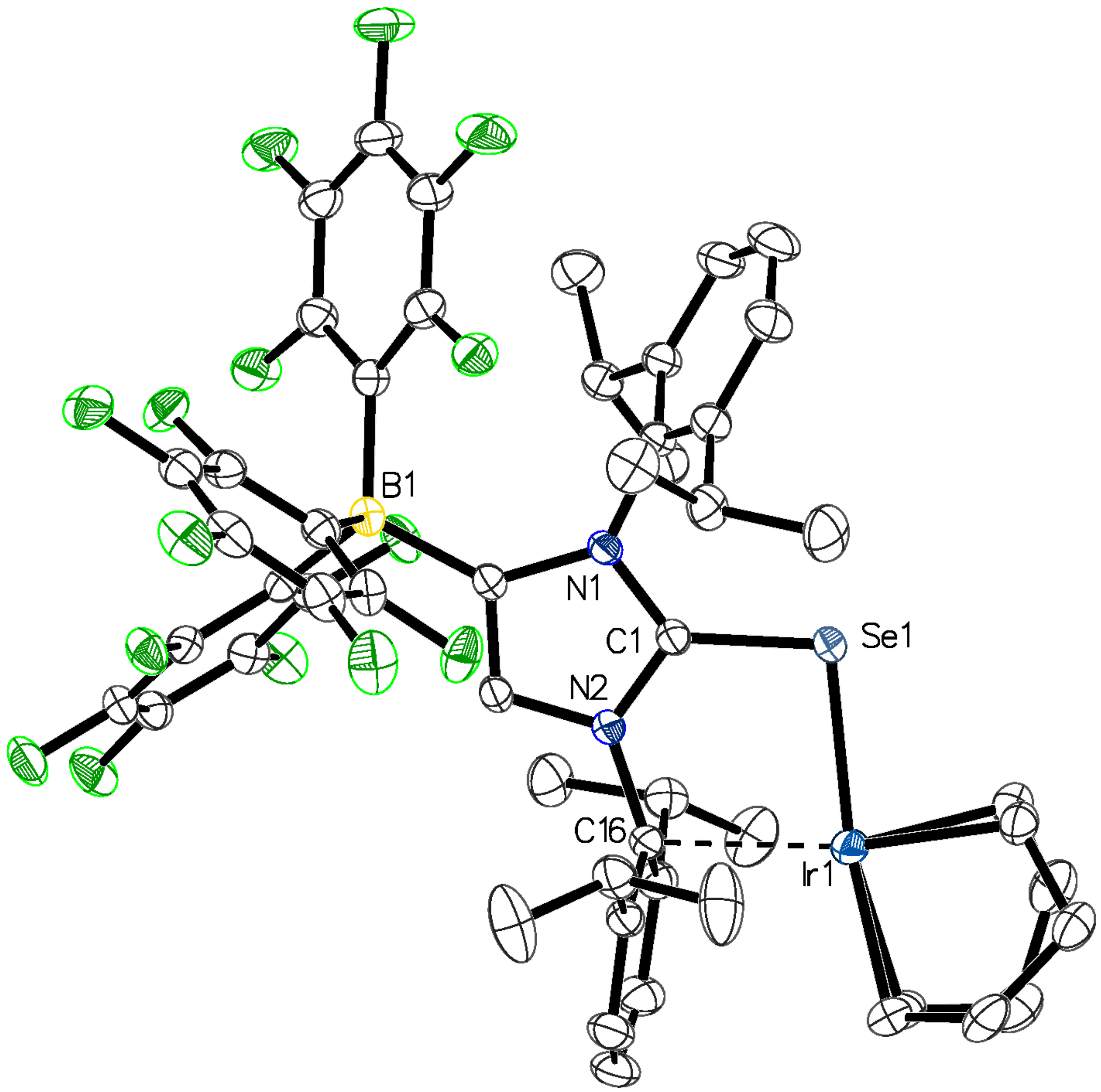

Figure S11. Molecular structure of **12** with thermal displacement parameters drawn at 50% probability; hydrogen atoms are omitted for clarity.

S2.8 [{(WCA-IDipp)S}$Rh_2(COD)_2Cl$] (**13**)

Table S12. Crystallographic data for compound **13**.

Compound	**13**	
Identification code	2122419	
Empirical formula	$C_{61}H_{59}BClF_{15}N_2Rh_2S$	
Formula weight	1389.24	
Temperature	100(2) K	
Wavelength	1.54184 Å	
Instrument (scan mode)	XtaLAB Synergy, Single source at home/near, HyPix ([illegible] scan)	
Crystal system	Monoclinic	
Space group	*I*2/*a*	
Unit cell dimensions	a = 25.5328(10) Å	α = 90°
	b = 19.4623(8) Å	β = 100.499(4)°
	c = 23.5244(12) Å	γ = 90°
Volume	11494.2(9) $Å^3$	
Z	8	
Density (calculated)	1.606 Mg/m^3	
Absorption coefficient	6.208 mm^{-1}	
F(000)	5616	
Crystal habitus	irregular (orange)	
Crystal size	0.092 x 0.053 x 0.026 mm^3	
Theta range for data collection	2.873 to 79.517°	
Index ranges	-32<=h<=32, -21<=k<=24, -29<=l<=29	
Reflections collected	117429	
Independent reflections	12176 [R(int) = 0.0960]	
Completeness to theta = 67.684°	100.0 %	
Absorption correction	Gaussian	
Max. and min. transmission	1.000 and 0.903	
Refinement method	Full-matrix least-squares on F^2	
Data / restraints / parameters	12176 / 96 / 756	
Goodness-of-fit on F^2	1.059	
Final R indices [I>2sigma(I)]	R1 = 0.0869, wR2 = 0.1672	
R indices (all data)	R1 = 0.0965, wR2 = 0.1716	
Largest diff. peak and hole	2.901 and -1.983 e.$Å^{-3}$	
Crystallisation Details:	A saturated solution of WCA-IDipp-S-Rh2COD2Cl in toluene was layered with nhexane at ambient temperatures under inert conditions.	
Solution	SHELXT-2014/5 (G. M. Sheldrick, Acta Cryst., 2015, A71, 3-8)	
Refinement	SHELXL-2018/3 (G. M. Sheldrick, Acta Cryst., 2008, A64, 112-122)	
Interface	OLEX2 v1.2 (O. V. Dolomanov, J. Appl. Cryst., 2009, 42, 339-341)	
Measurement and Refinement Details:	-	

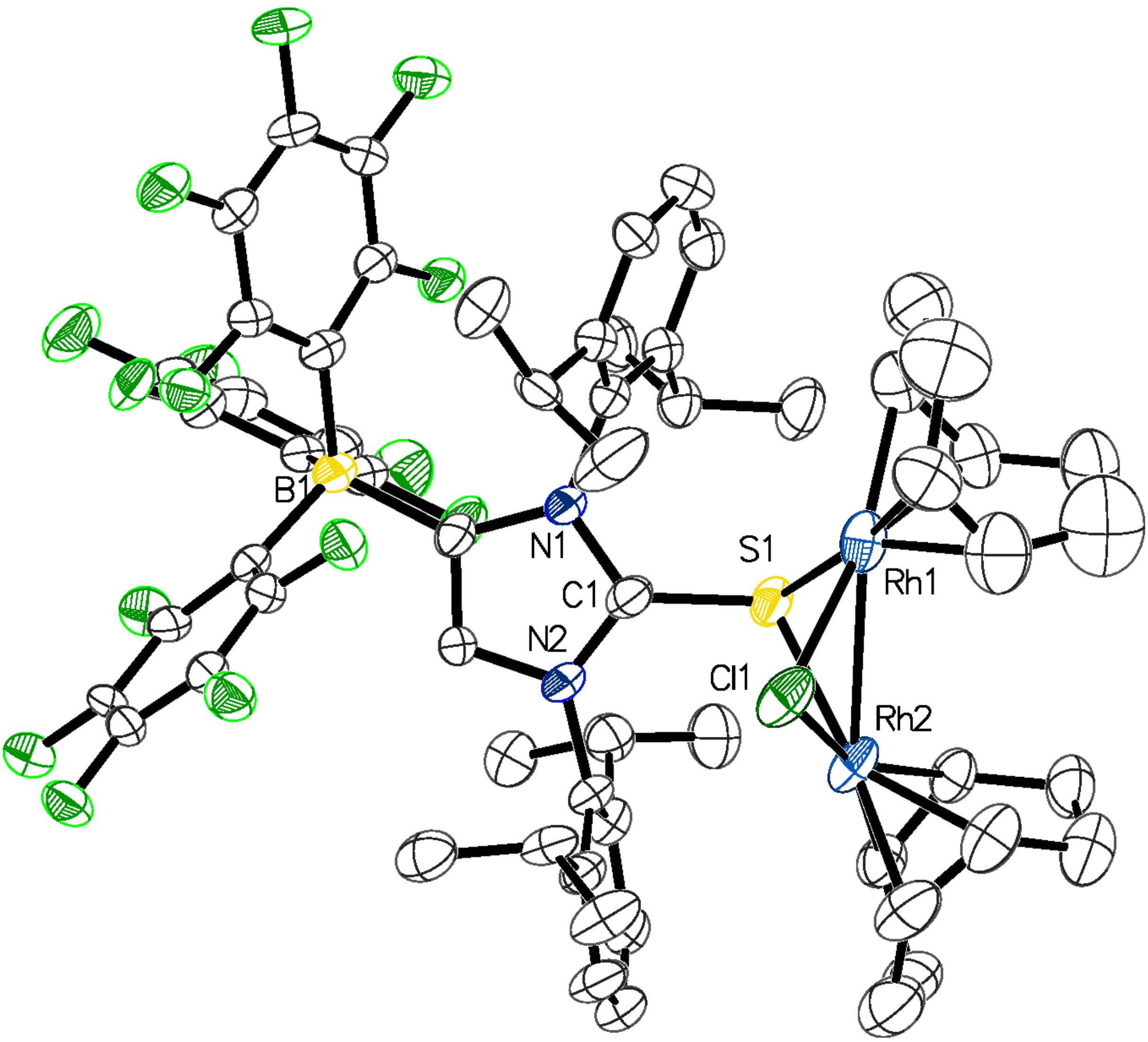

Figure S12. Molecular structure of **13** with thermal displacement parameters drawn at 50% probability; hydrogen atoms are omitted for clarity.

S2.9 [{(WCA-IDipp)S}$Ir_2(COD)_2Cl$]·3.5THF (**14**·3.5(THF))

Table S13. Crystallographic data for compound **14**·3.5(THF).

Compound	**14**·3.5(THF)	
Identification code	2122422	
Empirical formula	$C_{75}H_{87}BClF_{15}Ir_2N_2O_{3.50}S$	
Formula weight	1820.18	
Temperature	100(2) K	
Wavelength	0.71073 Å	
Instrument (scan mode)	XtaLAB Synergy, Single source at offset/far, HyPix ([illegible] scan)	
Crystal system	Monoclinic	
Space group	C2/c	
Unit cell dimensions	a = 44.6027(5) Å	α = 90°
	b = 14.85905(14) Å	β = 90.1615(9)°
	c = 21.54132(18) Å	γ = 90°
Volume	14276.5(2) $Å^3$	
Z	8	
Density (calculated)	1.694 Mg/m^3	
Absorption coefficient	3.881 mm^{-1}	
F(000)	7248	
Crystal habitus	plate (purple)	
Crystal size	0.148 x 0.110 x 0.025 mm^3	
Theta range for data collection	1.444 to 32.796°	
Index ranges	-63<=h<=62, -21<=k<=22, -32<=l<=32	
Reflections collected	491880	
Independent reflections	24288 [R(int) = 0.0901]	
Completeness to theta = 25.242°	100.0 %	
Absorption correction	Gaussian	
Max. and min. transmission	1.000 and 0.542	
Refinement method	Full-matrix least-squares on F^2	
Data / restraints / parameters	24288 / 90 / 982	
Goodness-of-fit on F^2	1.106	
Final R indices [I>2sigma(I)]	R1 = 0.0518, wR2 = 0.0850	
R indices (all data)	R1 = 0.0771, wR2 = 0.0906	
Largest diff. peak and hole	1.553 and -1.412 e.$Å^{-3}$	
Crystallisation Details:	A saturated solution of WCA-IDipp-$SIr_2Cl(COD)_2$ in THF was layered with *n*hexane and then stored at -40°C for several days.	
Solution	SHELXT-2014/5 (G. M. Sheldrick, Acta Cryst., 2015, A71, 3-8)	
Refinement	SHELXL-2018/3 (G. M. Sheldrick, Acta Cryst., 2008, A64, 112-122)	
Interface	OLEX2 v1.2 (O. V. Dolomanov, J. Appl. Cryst., 2009, 42, 339-341)	
Measurement and Refinement Details:	One THF molecule is disordered over two positions and was refined as such. One THF molecule is located on a special position and was refined as such, hence the non-integer number of oxygen atoms in the sum formula.	

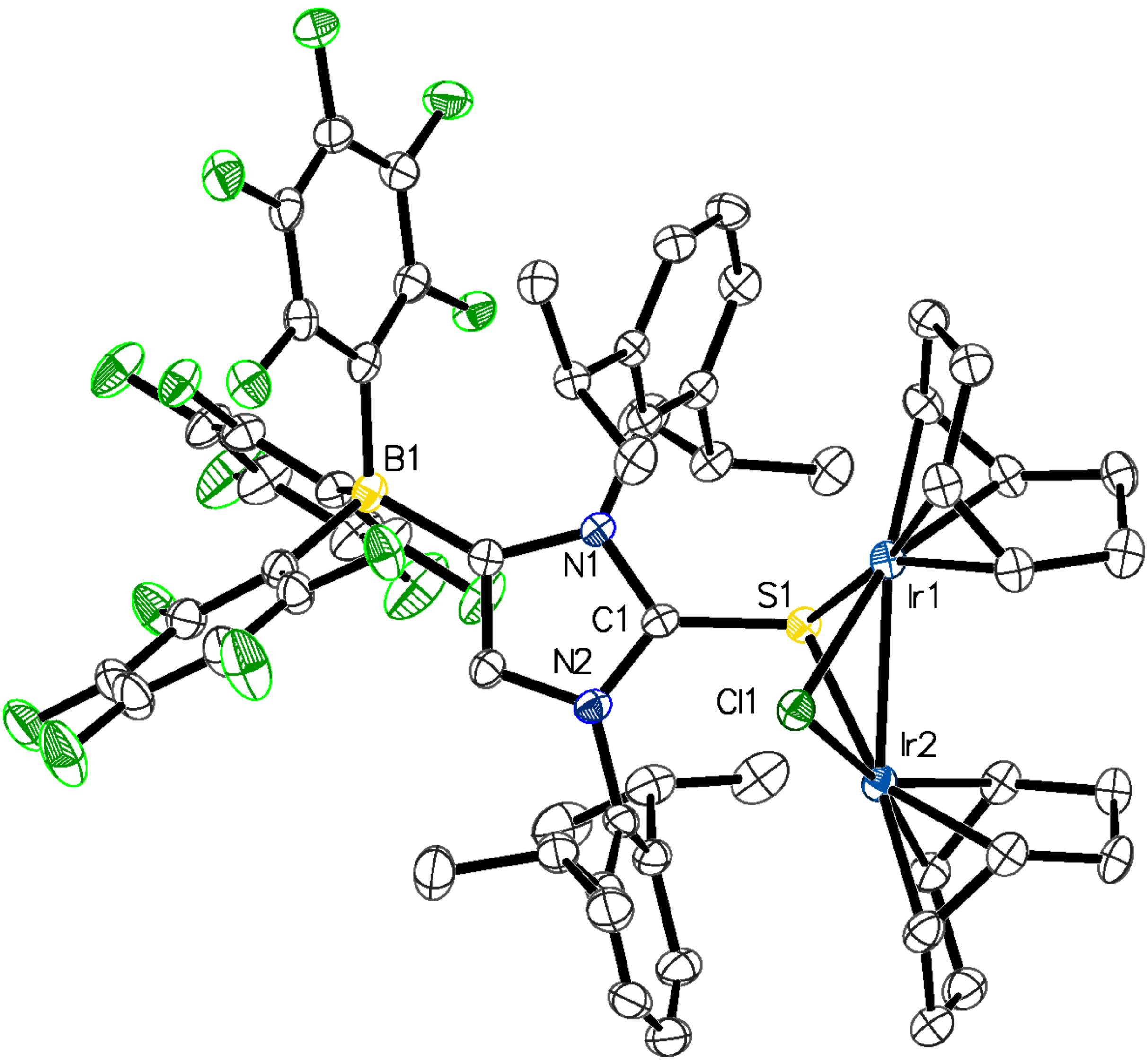

Figure S13. Molecular structure of **14** in **14**·3.5(THF) with thermal displacement parameters drawn at 50% probability; hydrogen atoms are omitted for clarity.

S2.10 [{(WCA-IDipp)Se}$Rh_2(COD)_2Cl$] (**15**)

Table S14. Crystallographic data for compound **15**.

Compound	**15**	
Identification code	2122416	
Empirical formula	$C_{61}H_{59}BClF_{15}N_2Rh_2Se$	
Formula weight	1436.14	
Temperature	100(2) K	
Wavelength	1.54184 Å	
Instrument (scan mode)	XtaLAB Synergy, Single source at home/near, HyPix (□ scan)	
Crystal system	Monoclinic	
Space group	*I*2/*a*	
Unit cell dimensions	a = 25.6070(6) Å	α = 90°
	b = 19.4522(6) Å	β = 100.005(4)°
	c = 23.5282(8) Å	γ = 90°
Volume	11541.5(6) $Å^3$	
Z	8	
Density (calculated)	1.653 Mg/m^3	
Absorption coefficient	6.566 mm^{-1}	
F(000)	5760	
Crystal habitus	plate (orange)	
Crystal size	0.065 x 0.037 x 0.022 mm^3	
Theta range for data collection	2.869 to 79.404°	
Index ranges	-32<=h<=26, -24<=k<=24, -28<=l<=29	
Reflections collected	127390	
Independent reflections	12260 [R(int) = 0.0887]	
Completeness to theta = 67.684°	100.0 %	
Absorption correction	Gaussian	
Max. and min. transmission	1.000 and 0.958	
Refinement method	Full-matrix least-squares on F^2	
Data / restraints / parameters	12260 / 0 / 756	
Goodness-of-fit on F^2	1.058	
Final R indices [I>2sigma(I)]	R1 = 0.0599, wR2 = 0.1485	
R indices (all data)	R1 = 0.0714, wR2 = 0.1557	
Largest diff. peak and hole	1.744 and -1.504 e.$Å^{-3}$	
Crystallisation Details:	A saturated solution of WCA-IDipp-SeRh_2Cl in toluene was layered with nhexane at ambient temperatures under inert conditions.	
Solution	SHELXT-2014/5 (G. M. Sheldrick, Acta Cryst., 2015, A71, 3-8)	
Refinement	SHELXL-2018/3 (G. M. Sheldrick, Acta Cryst., 2008, A64, 112-122)	
Interface	OLEX2 v1.2 (O. V. Dolomanov, J. Appl. Cryst., 2009, 42, 339-341)	
Measurement and Refinement Details:	-	

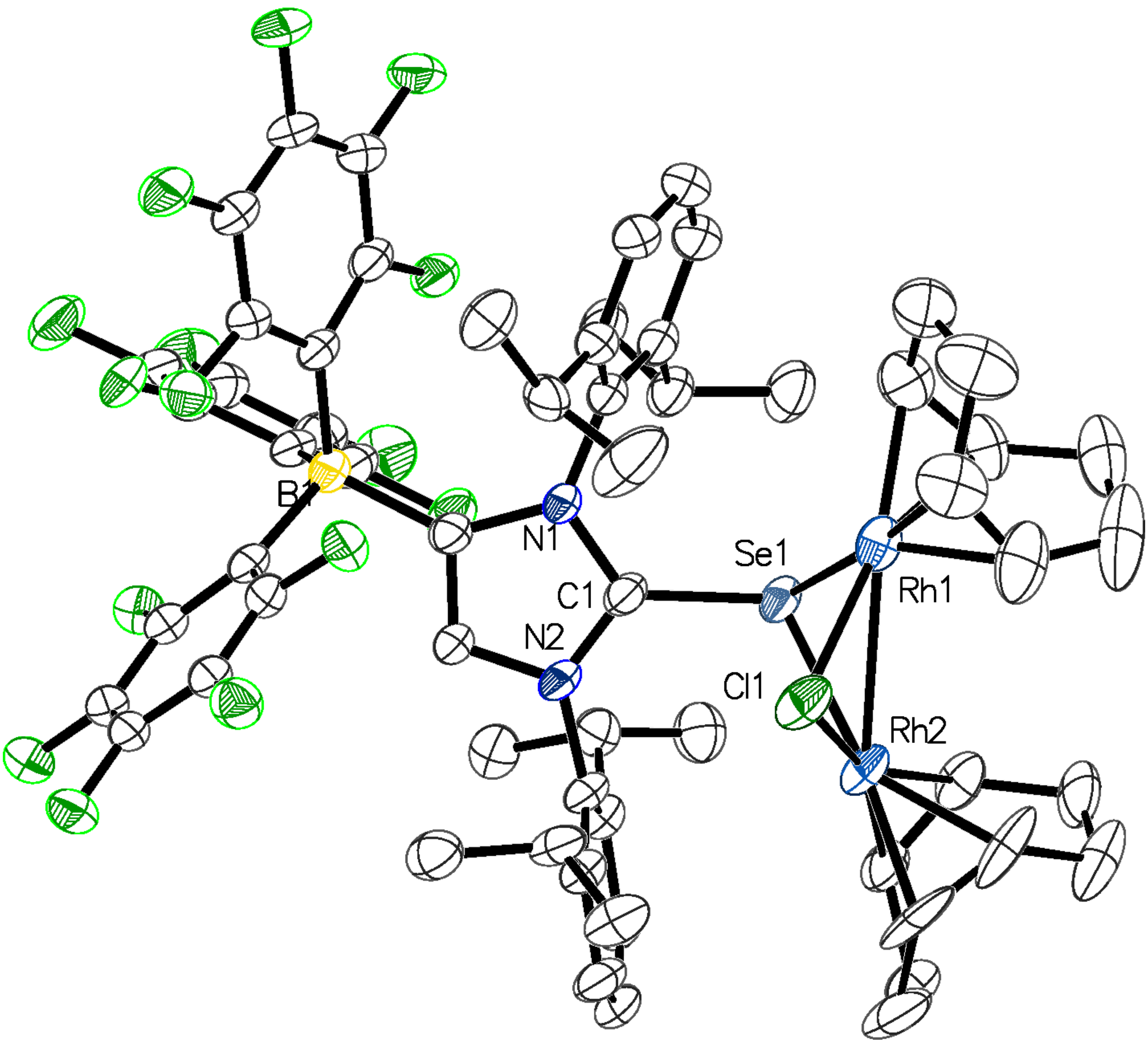

Figure S14. Molecular structure of **15** with thermal displacement parameters drawn at 50% probability; hydrogen atoms are omitted for clarity.

S2.11 [{(WCA-IDipp)Se}$Ir_2(COD)_2Cl$]·0.5THF·HMDSO (**16**·0.5(THF)0.5(HMDSO))

Table S15. Crystallographic data for compound **16**·0.5(THF)0.5(HMDSO).

Compound	**16**·0.5(THF)0.5(HMDSO)	
Identification code	2122412	
Empirical formula	$C_{66}H_{72}BClF_{15}Ir_2N_2OSeSi$	
Formula weight	1731.96	
Temperature	100(2) K	
Wavelength	0.71073 Å	
Instrument (scan mode)	XtaLAB Synergy, Single source at offset/far, HyPix (w scan)	
Crystal system	Orthorhombic	
Space group	*Pbcn*	
Unit cell dimensions	a = 19.0078(3) Å	α = 90°
	b = 25.2525(4) Å	β = 90°
	c = 28.5398(6) Å	γ = 90°
Volume	13698.9(4) $Å^3$	
Z	8	
Density (calculated)	1.680 Mg/m^3	
Absorption coefficient	4.552 mm^{-1}	
F(000)	6792	
Crystal habitus	irregular (purple)	
Crystal size	0.126 x 0.087 x 0.029 mm^3	
Theta range for data collection	2.526 to 28.281°	
Index ranges	-25<=h<=25, -33<=k<=33, -37<=l<=37	
Reflections collected	340464	
Independent reflections	16965 [R(int) = 0.1575]	
Completeness to theta = 25.242°	99.9 %	
Absorption correction	Gaussian	
Max. and min. transmission	1.000 and 0.582	
Refinement method	Full-matrix least-squares on F^2	
Data / restraints / parameters	16965 / 127 / 876	
Goodness-of-fit on F^2	1.169	
Final R indices [I>2sigma(I)]	R1 = 0.0603, wR2 = 0.1083	
R indices (all data)	R1 = 0.0843, wR2 = 0.1147	
Largest diff. peak and hole	2.529 and -1.766 e.$Å^{-3}$	

Crystallisation Details:	A saturated solution of (WCA-IDipp)SeIr2(COD)2Cl in thf was layered with HMDSO at room temperature under inert conditions.
Solution	SHELXT-2014/5 (G. M. Sheldrick, Acta Cryst., 2015, A71, 3-8)
Refinement	SHELXL-2018/3 (G. M. Sheldrick, Acta Cryst., 2008, A64, 112-122)
Interface	OLEX2 v1.2 (O. V. Dolomanov, J. Appl. Cryst., 2009, 42, 339-341)
Measurement and Refinement Details:	One HMDSO molecule is located on a special position and was refined as such. The methyl groups in the HMDSO molecule are disordered and were refined over two positions. Several SADI and ISOR restraints were applied. The THF molecule is only half occupied and was refined as such.

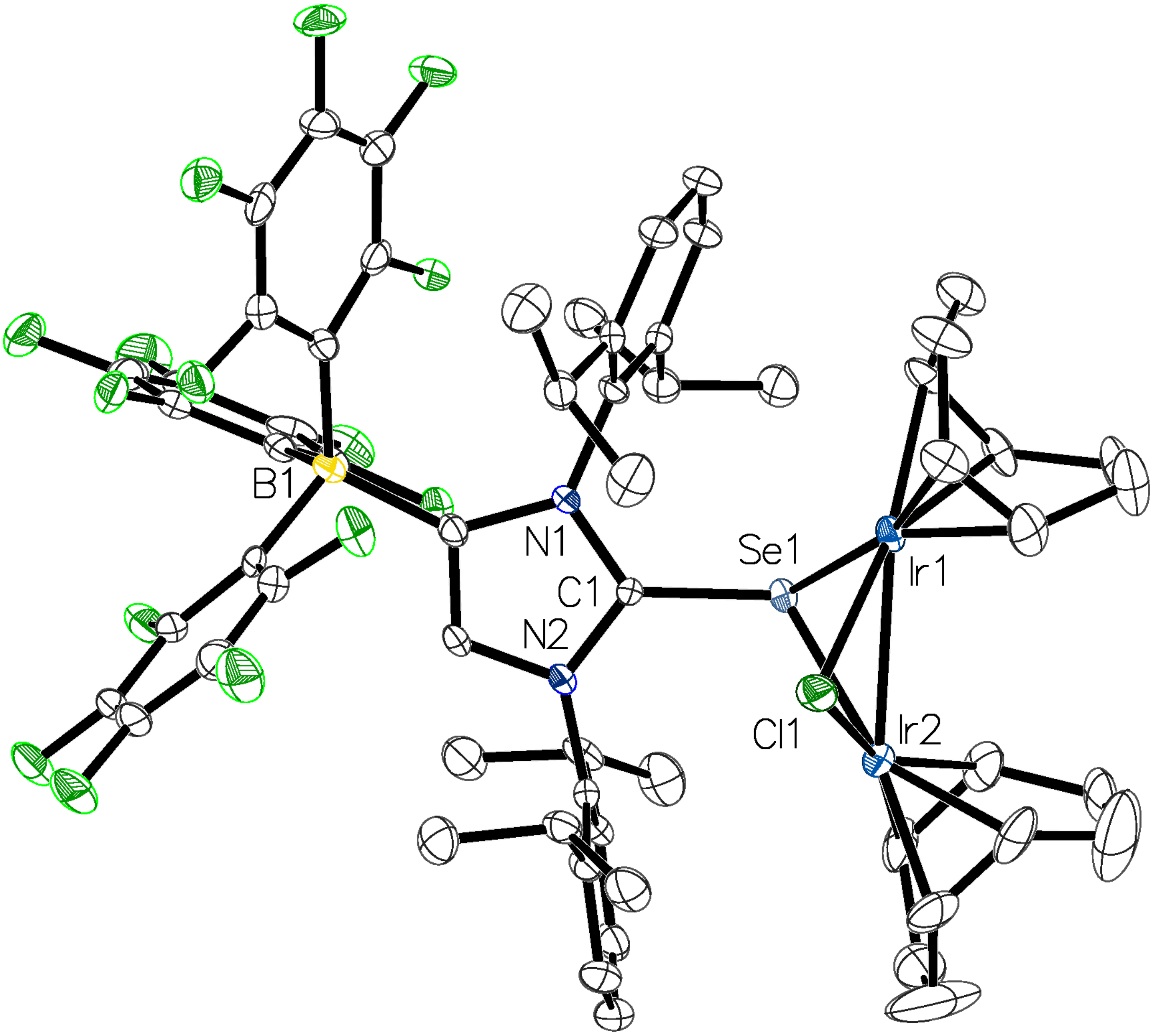

Figure S15. Molecular structure of **16** in **16**·0.5(THF)0.5(HMDSO)with thermal displacement parameters drawn at 50% probability; hydrogen atoms are omitted for clarity.

S3 ^{1}H, ^{11}B, ^{13}C, ^{19}F, and ^{77}Se NMR Spectra

S3.1 (WCA-IDipp)SSiMe$_3$ (**3**)

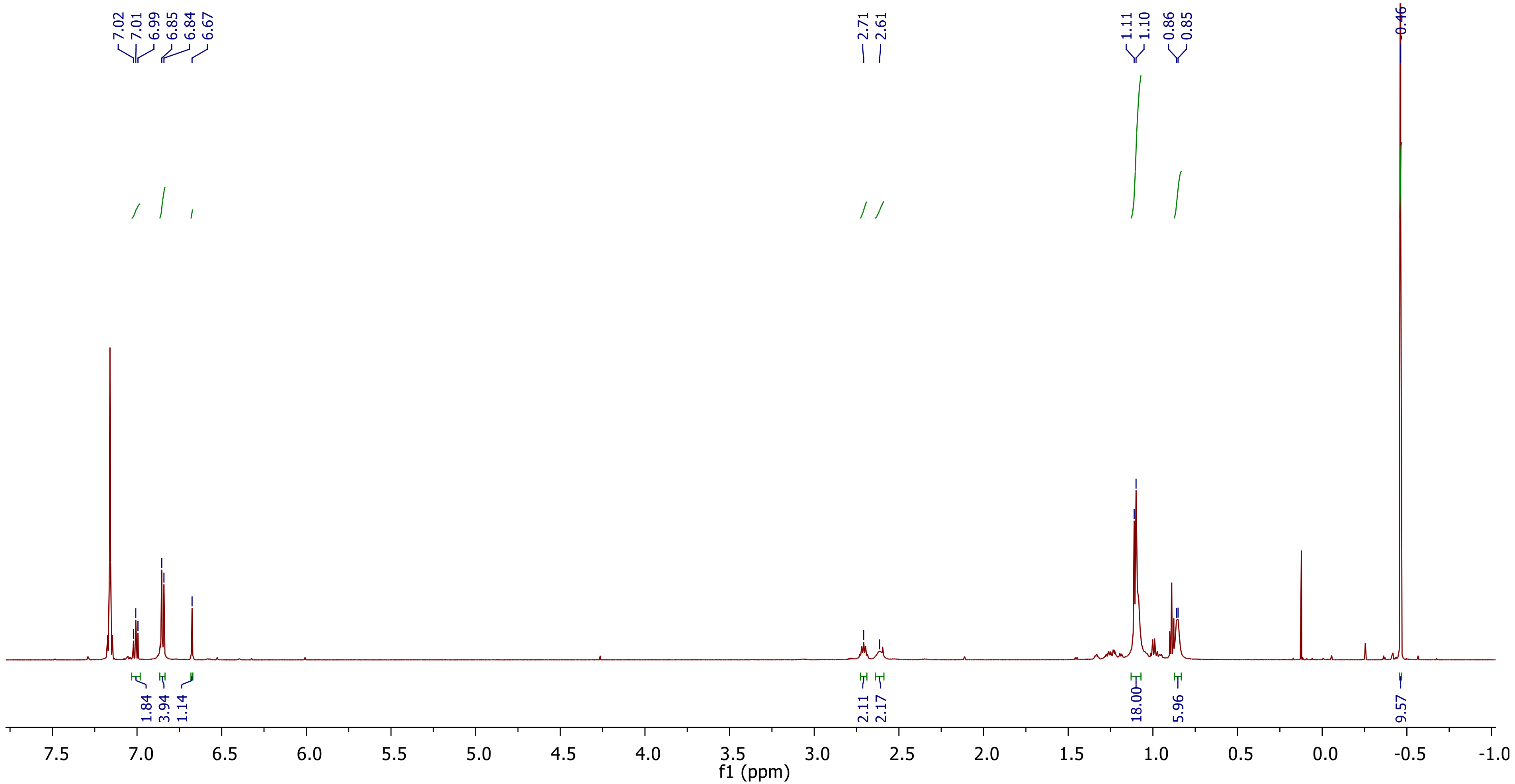

Figure S16. ^{1}H NMR spectrum (600 MHz, C_6D_6, 298K) of **3**.

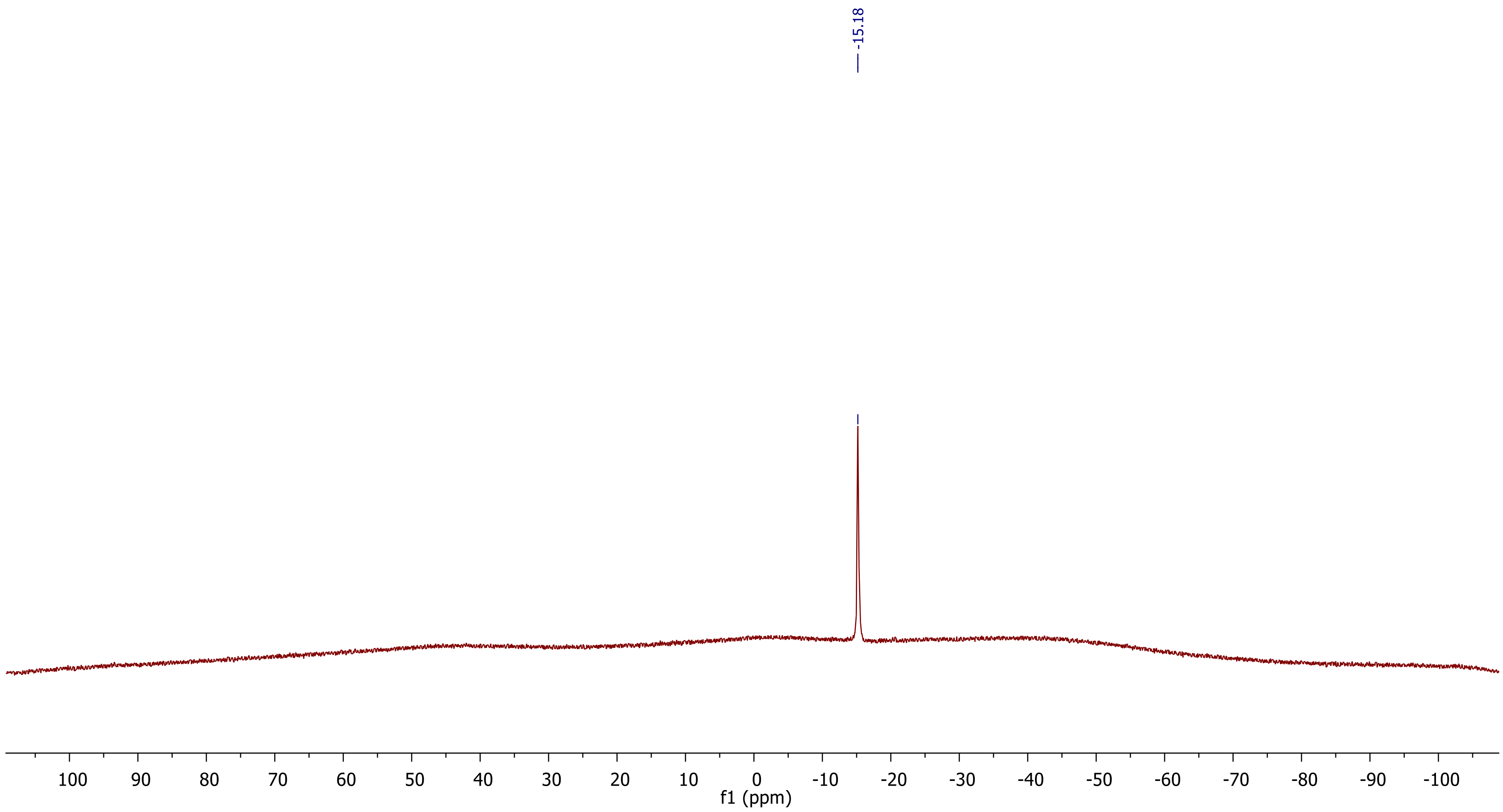

Figure S17. ^{11}B NMR spectrum (96 MHz, C_6D_6, 298K) of **3**.

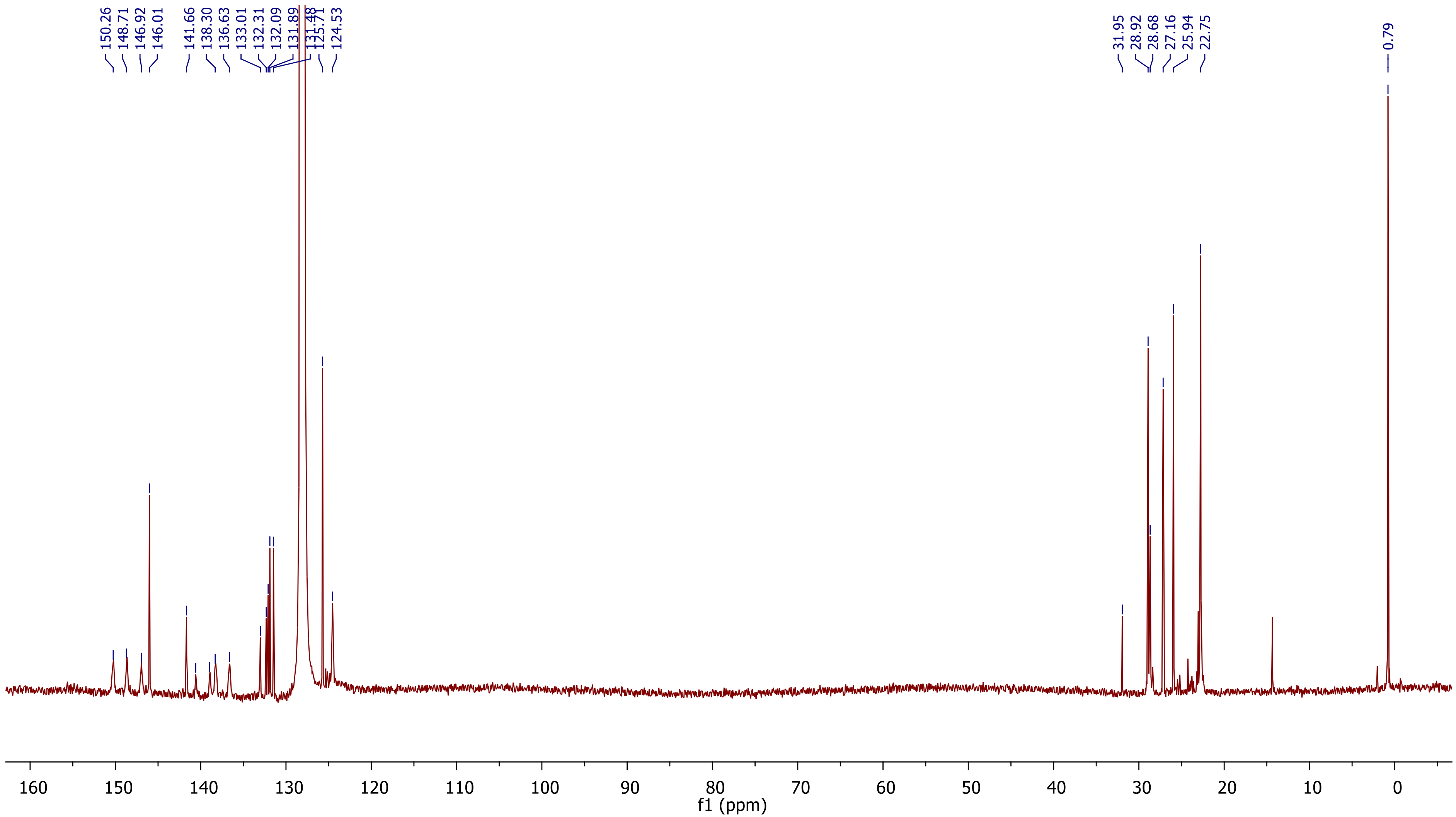

Figure S18. ^{13}C NMR spectrum (151 MHz, C_6D_6, 298K) of **3**.

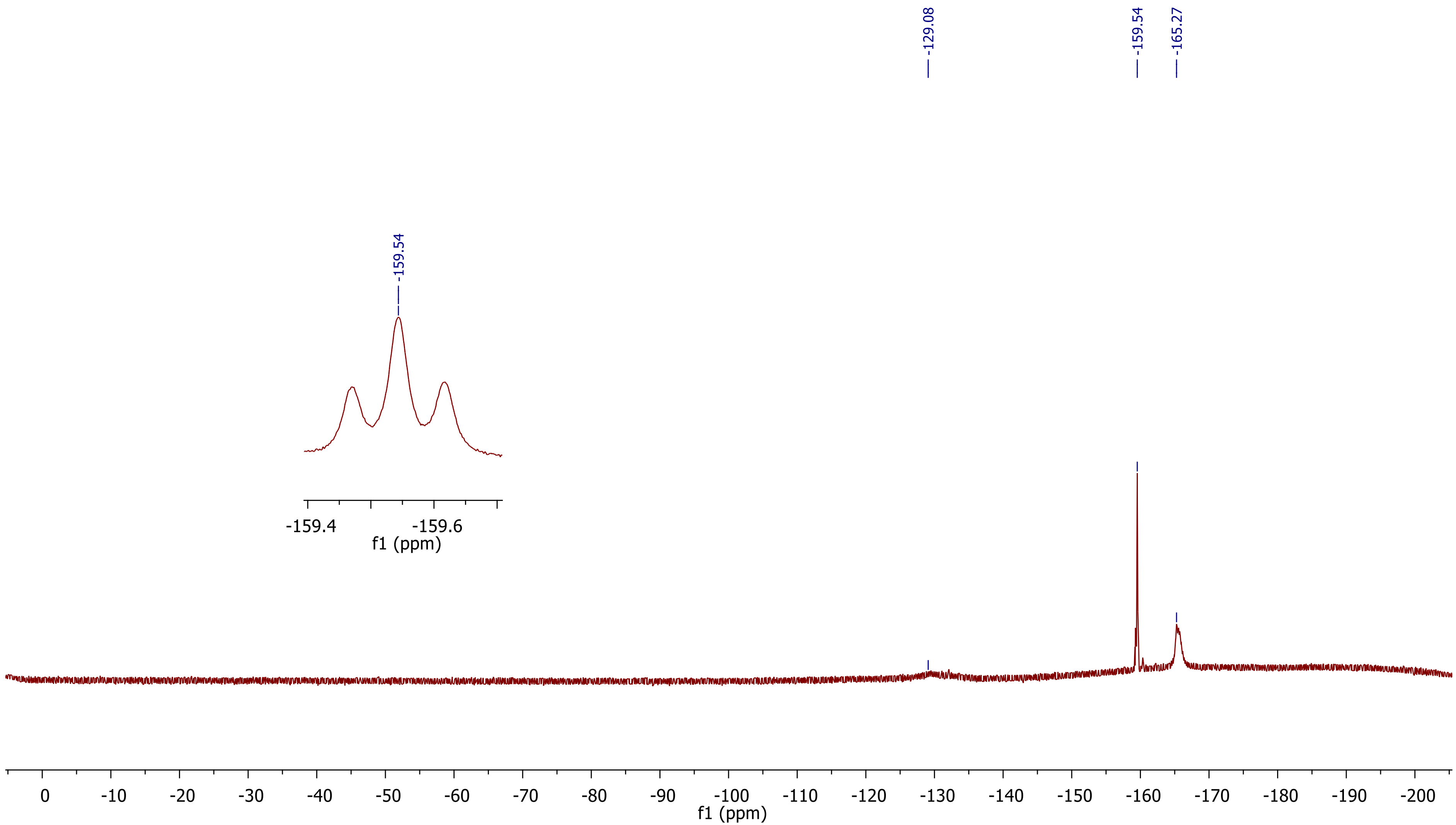

Figure S19. ^{19}F NMR spectrum (283 MHz, C_6D_6, 298K) of **3**.

S3.2 (WCA-IDipp)SeSiMe$_3$ (**4**)

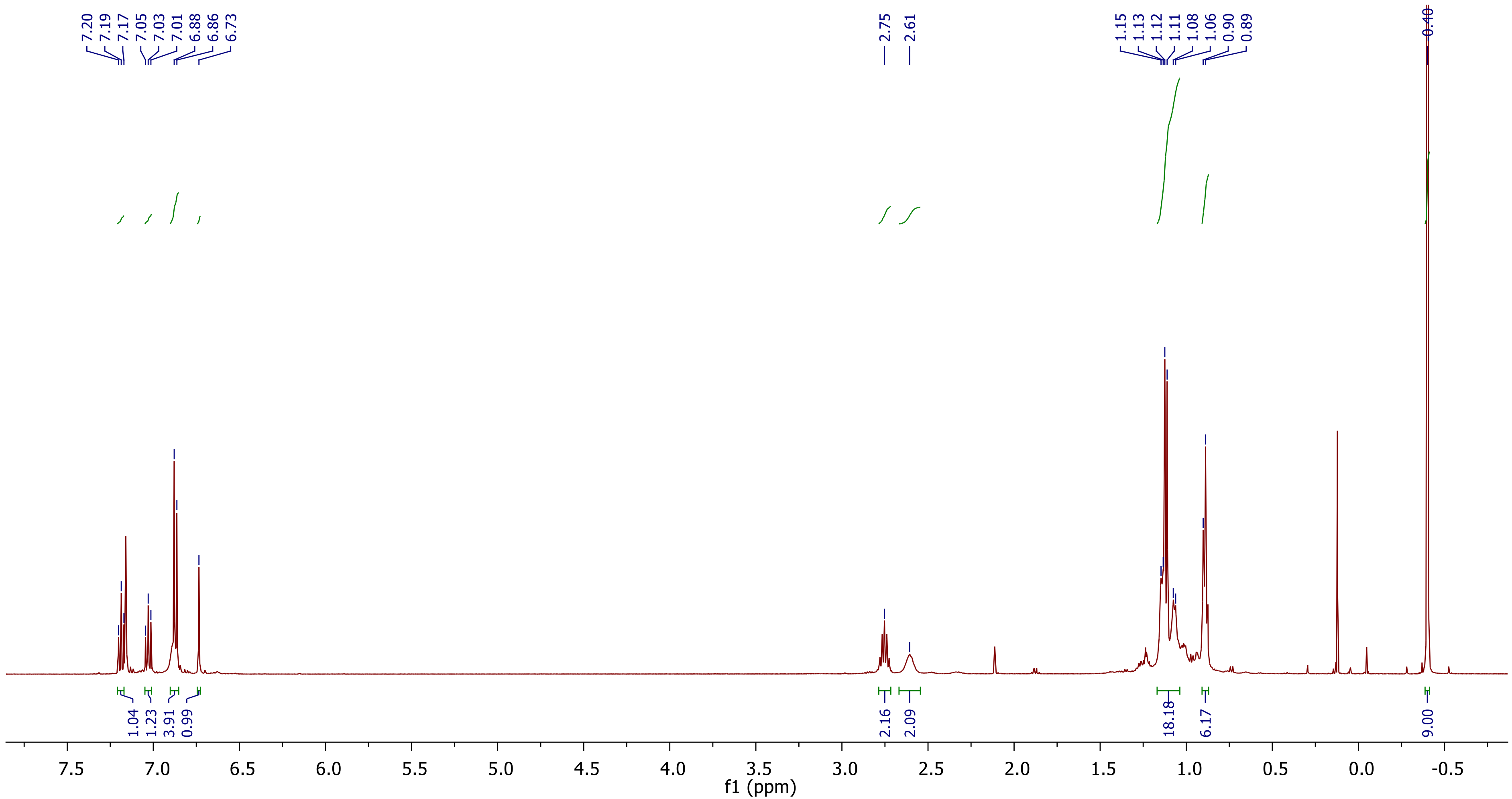

Figure S20. ^{1}H NMR spectrum (500 MHz, C_6D_6, 298K) of **4**.

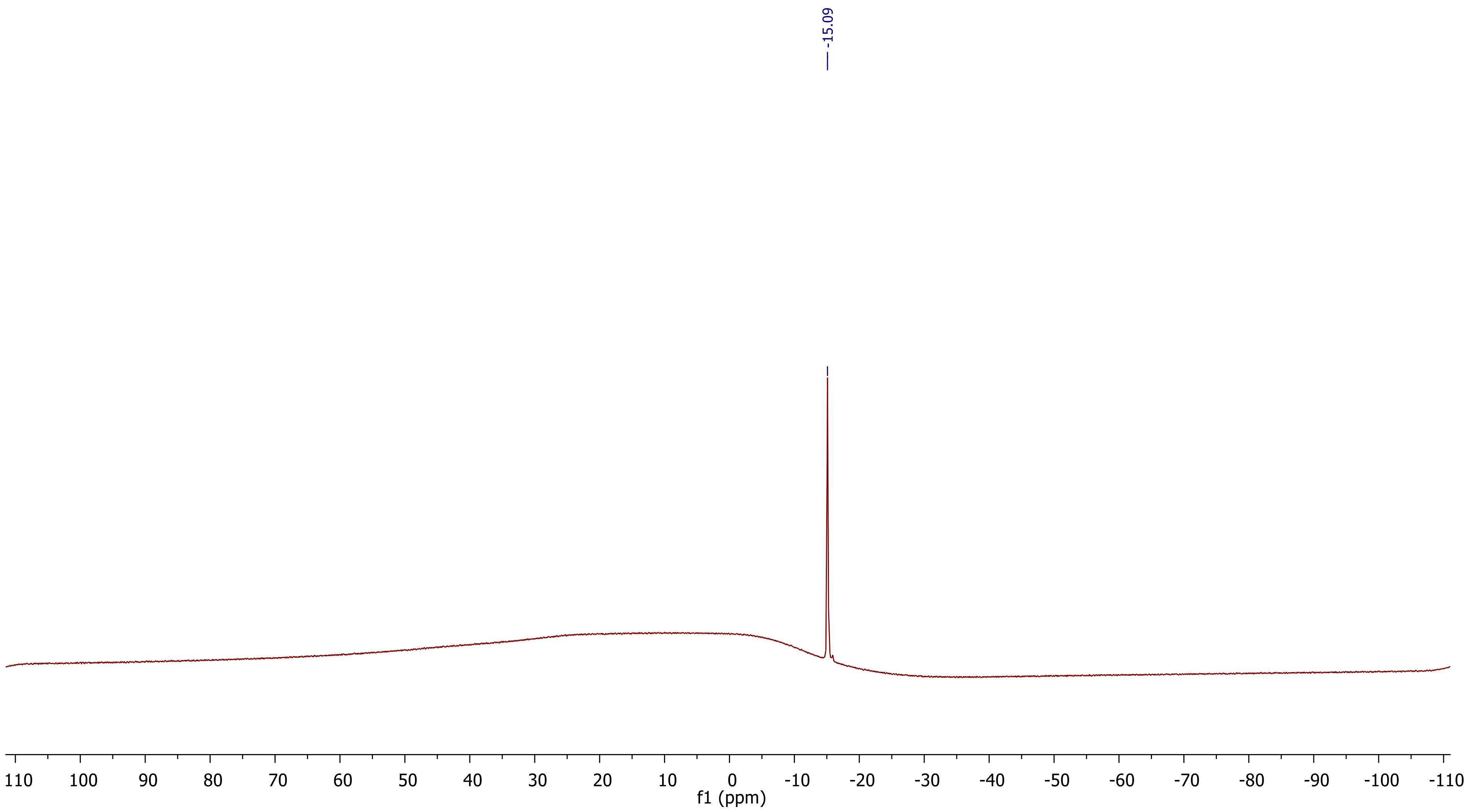

Figure S21. ^{11}B NMR spectrum (161 MHz, C_6D_6, 298K) of **4**.

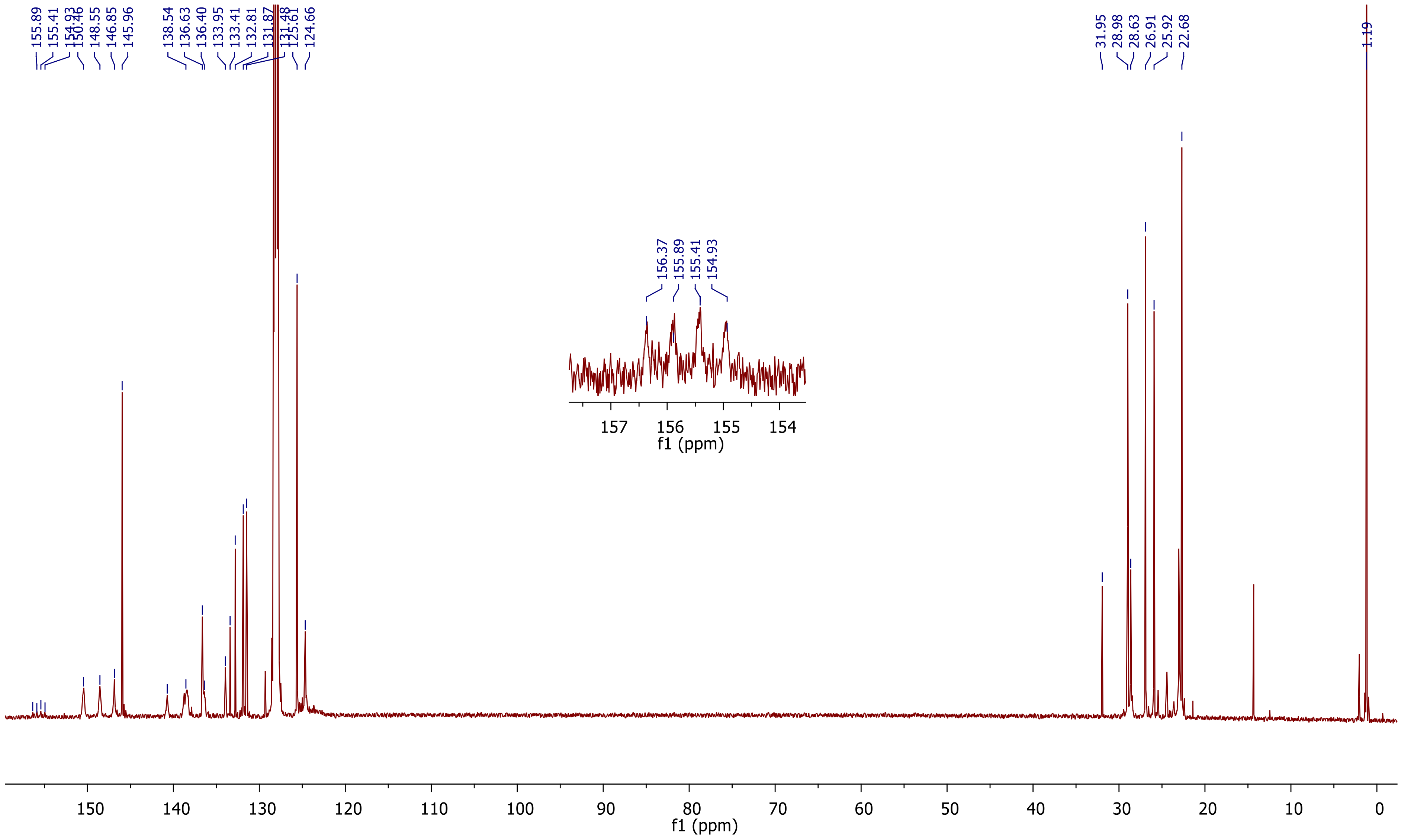

Figure S22. ^{13}C NMR spectrum (126 MHz, C_6D_6, 298K) of **4**.

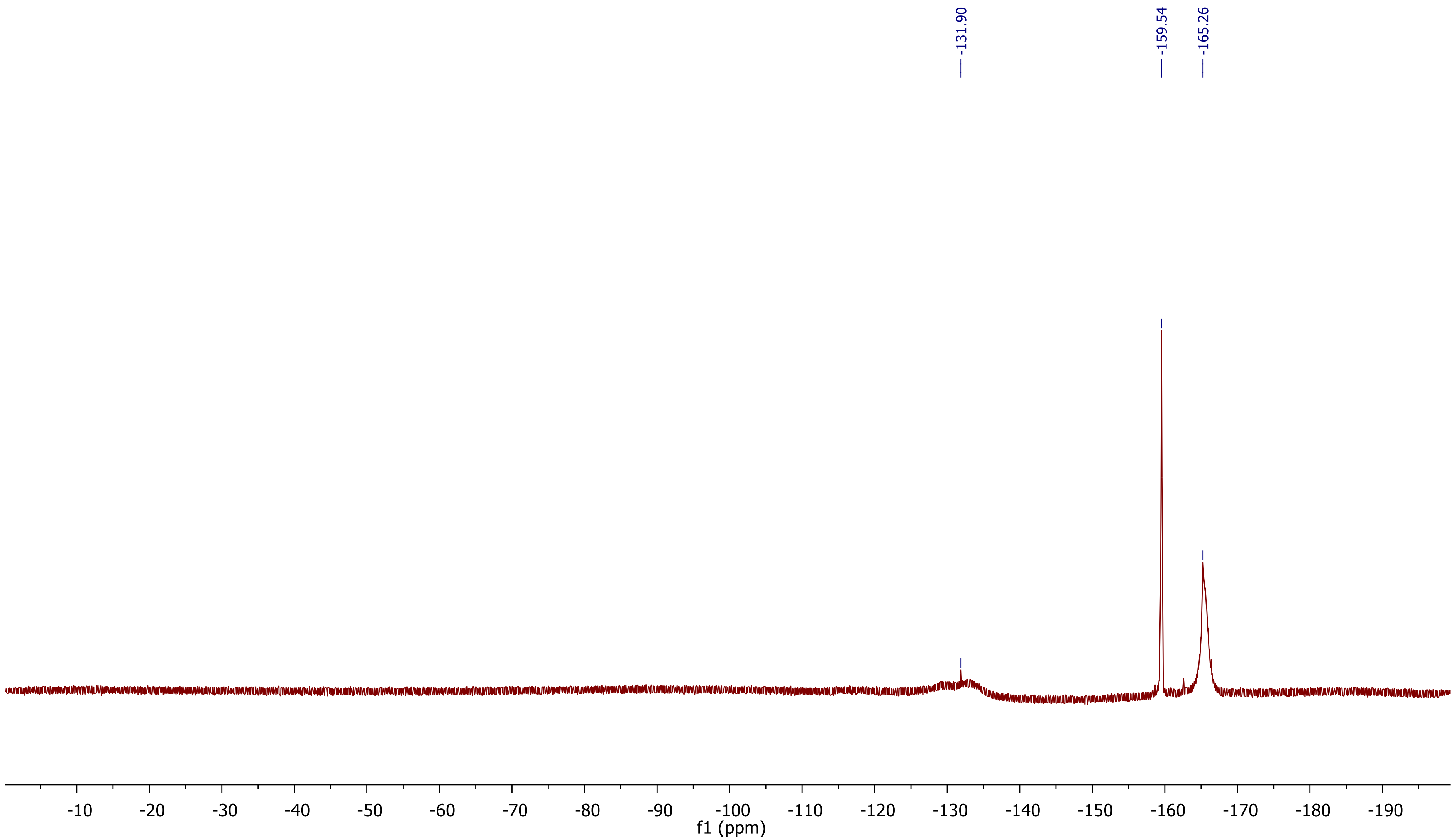

Figure S23. ^{19}F NMR spectrum (377 MHz, C_6D_6, 298K) of **4**.

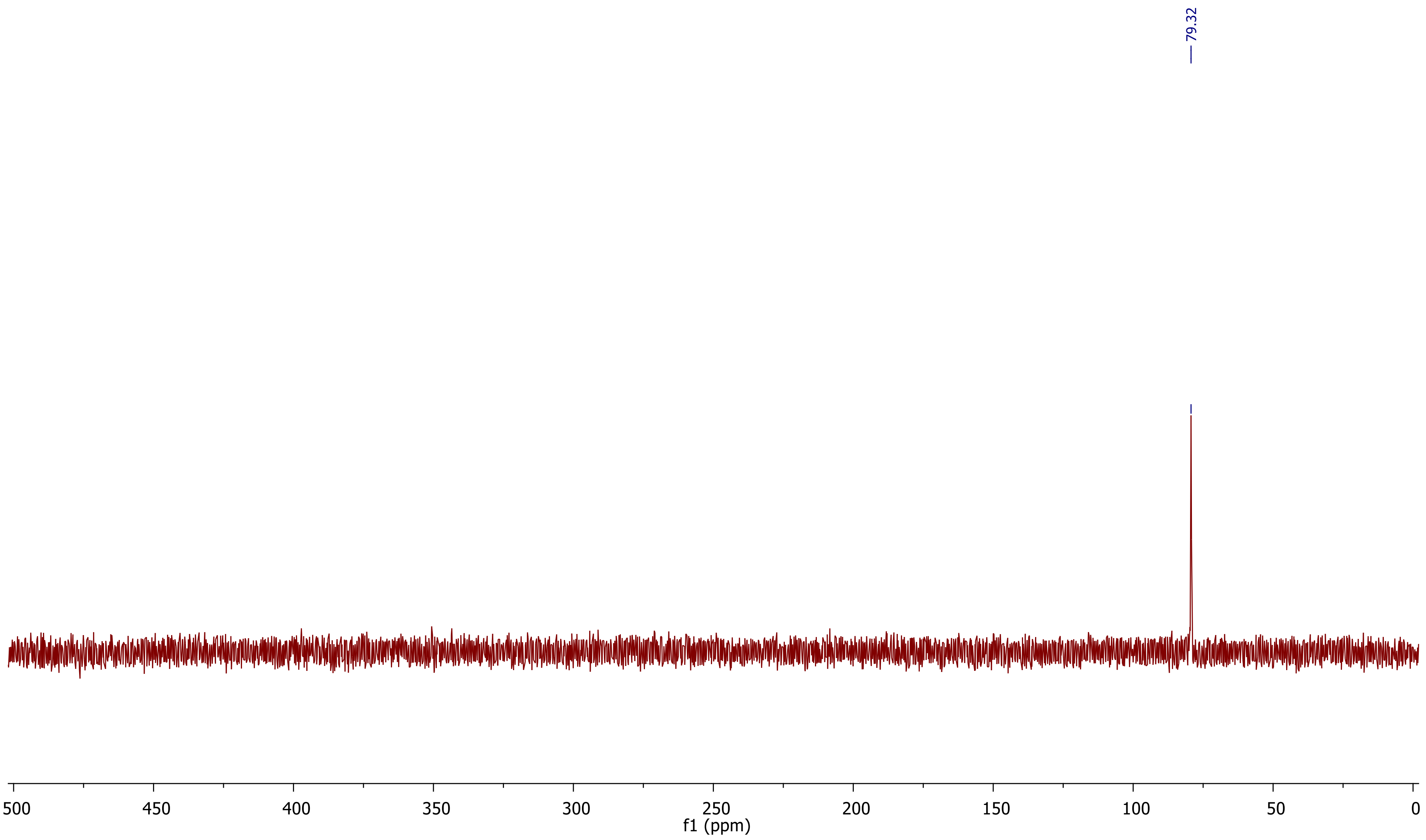

Figure S24. ^{77}Se NMR spectrum (95 MHz, C_6D_6, 298K) of **4**.

S3.3 (WCA-IDipp)Se$(CH_2)_4OSiMe_3$ (**4A**)

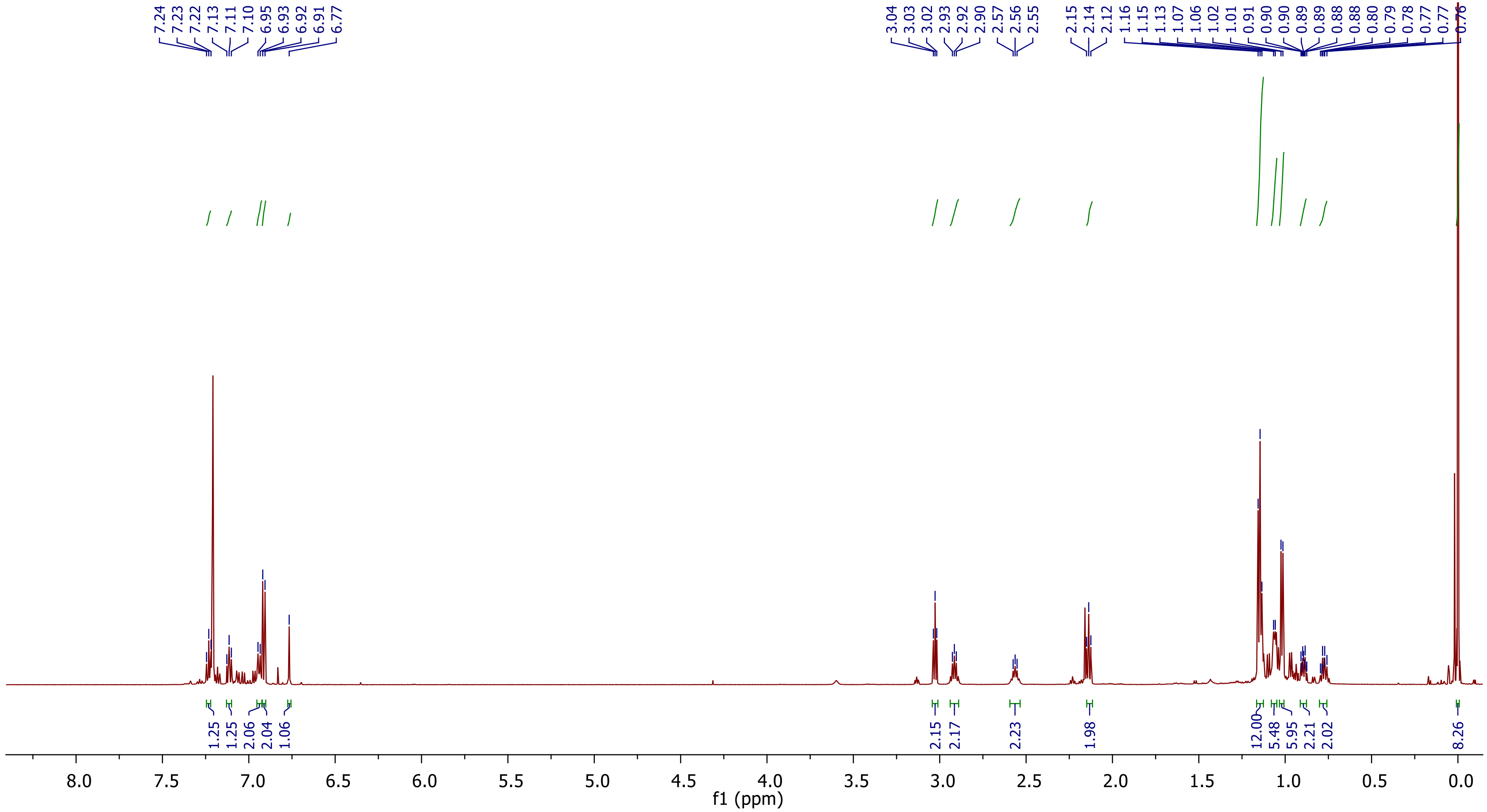

Figure S25. ^{1}H NMR spectrum (600 MHz, THF-d_8, 298K) of **4A**.

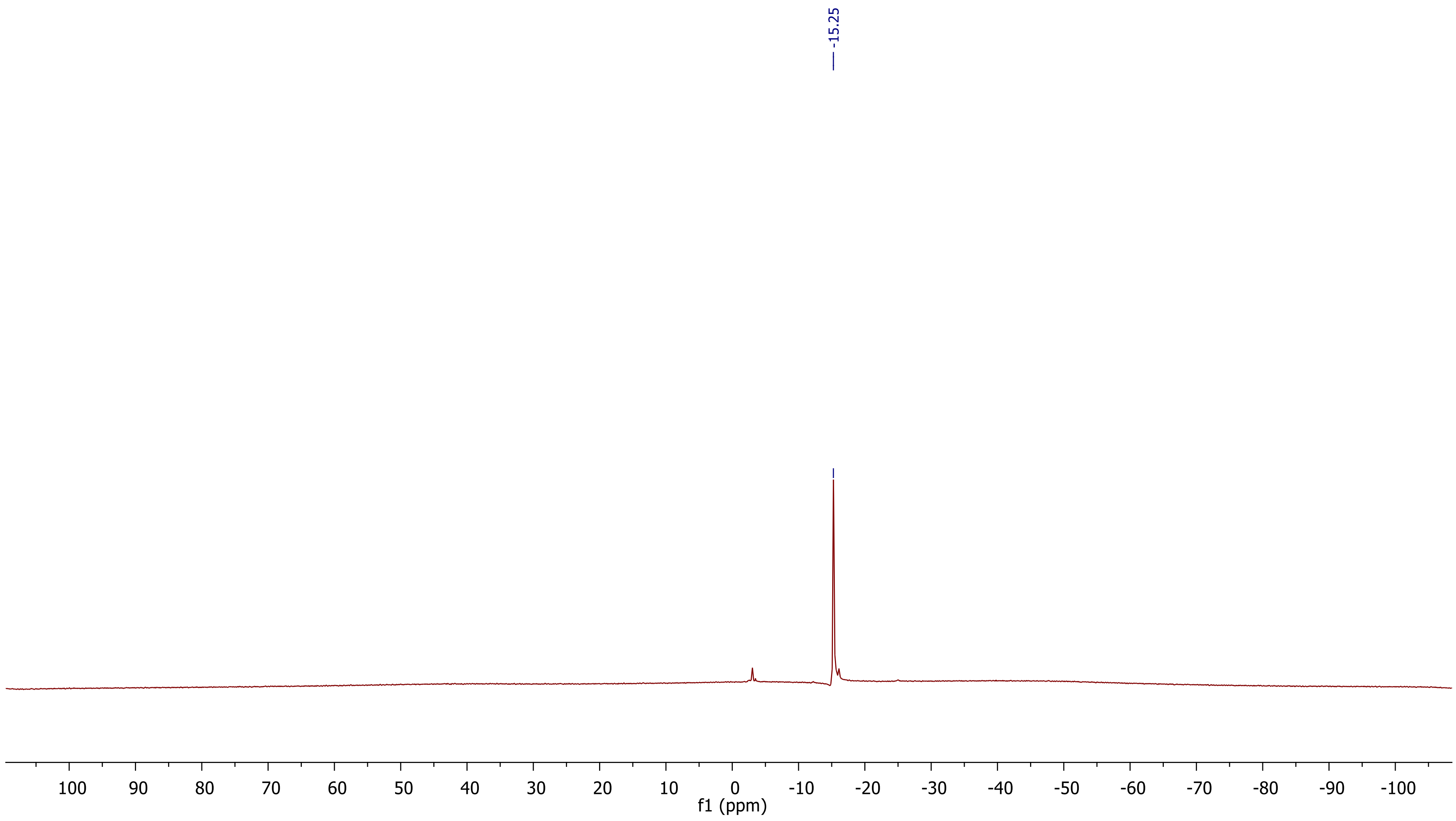

Figure S26. ^{11}B NMR spectrum (96 MHz, THF-d_8, 298K) of **4A**.

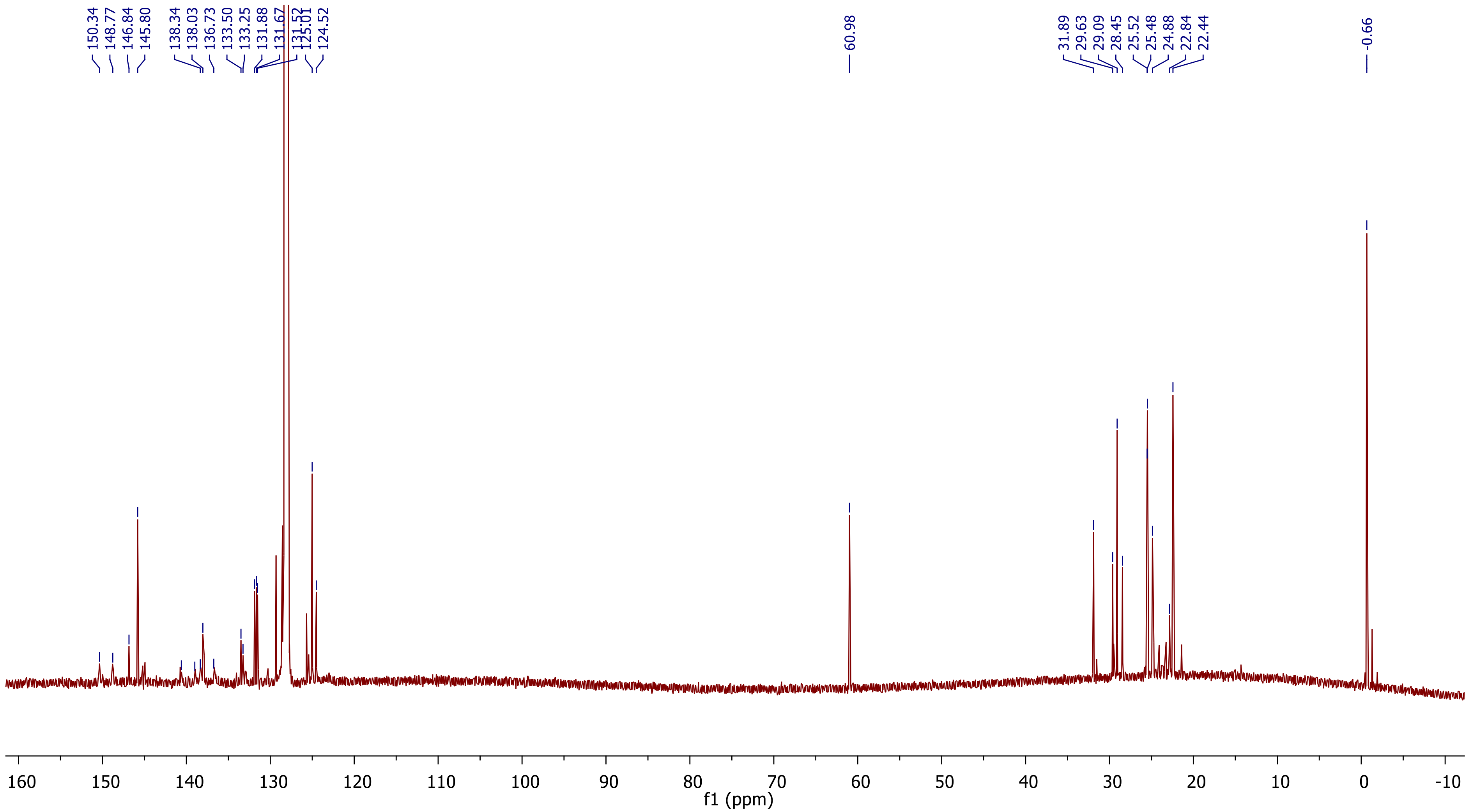

Figure S27. ^{13}C NMR spectrum (151 MHz, C_6D_6, 298K) of **4A**.

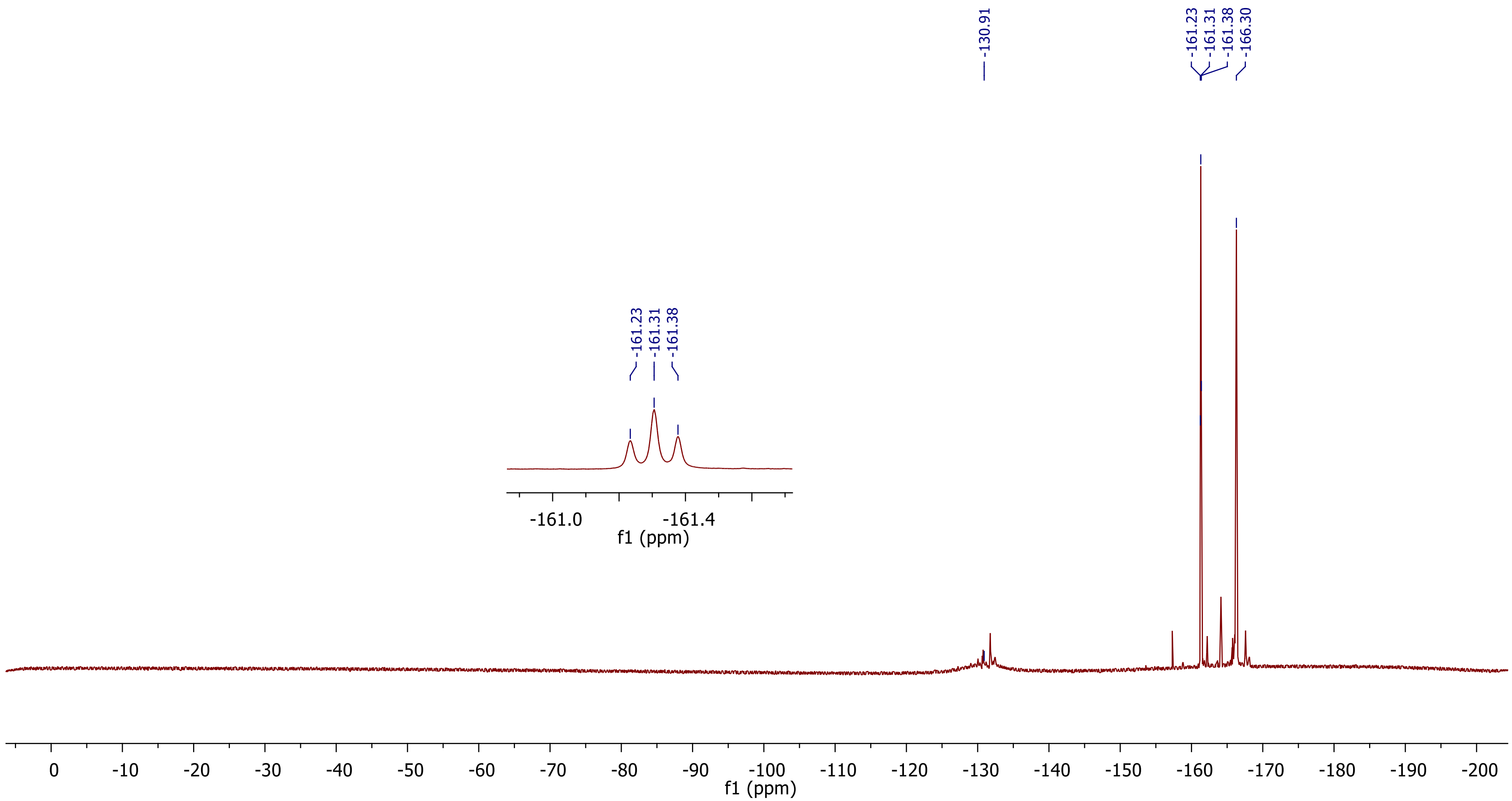

Figure S28. ^{19}F NMR spectrum (377 MHz, THF-d_8, 298K) of **4A**.

S3.4 [{(WCA-IDipp)S}RhCl(η^5-C_5Me_5)] (**5**)

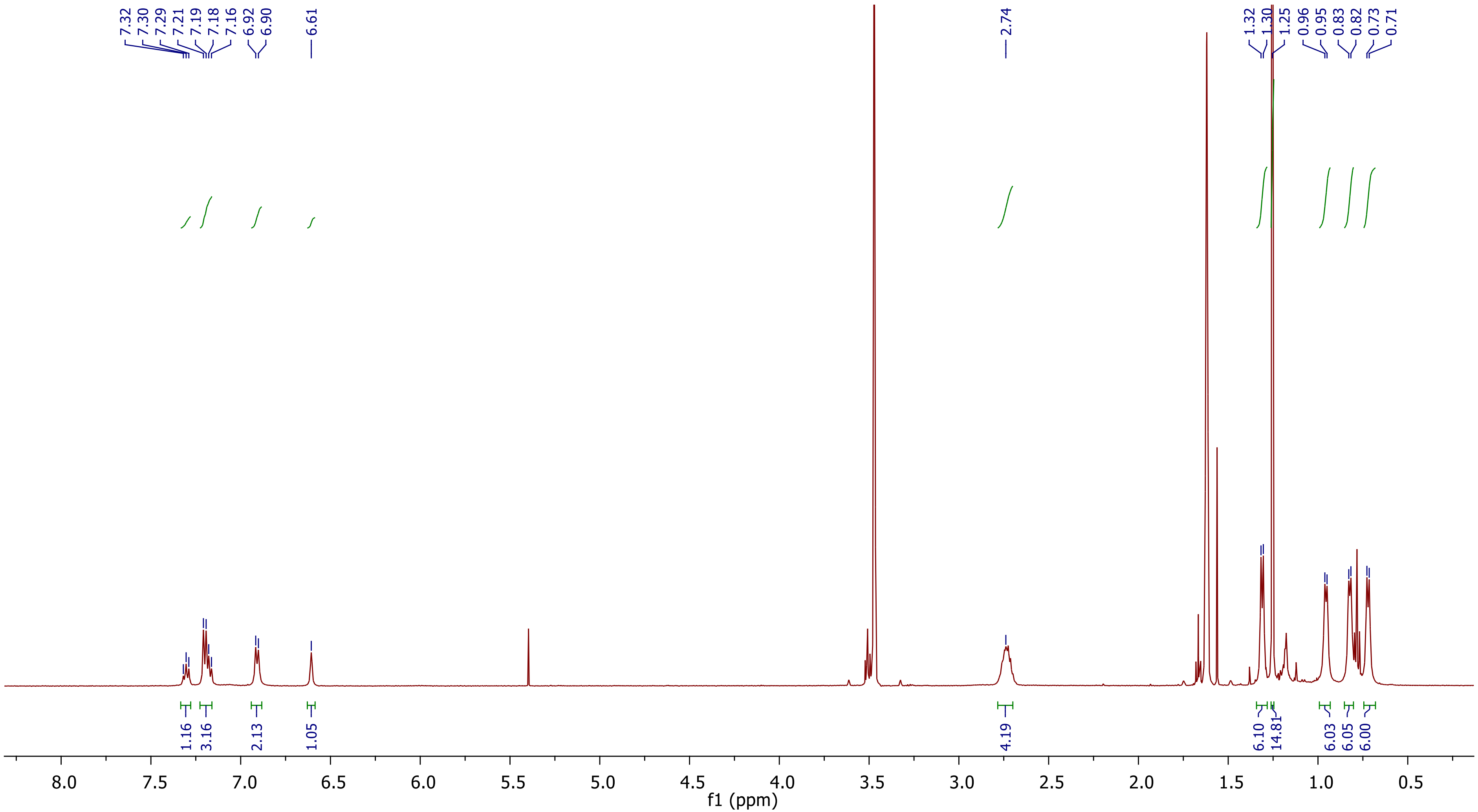

Figure S29. ^{1}H NMR spectrum (500 MHz, THF-d_8, 298K) of **5**.

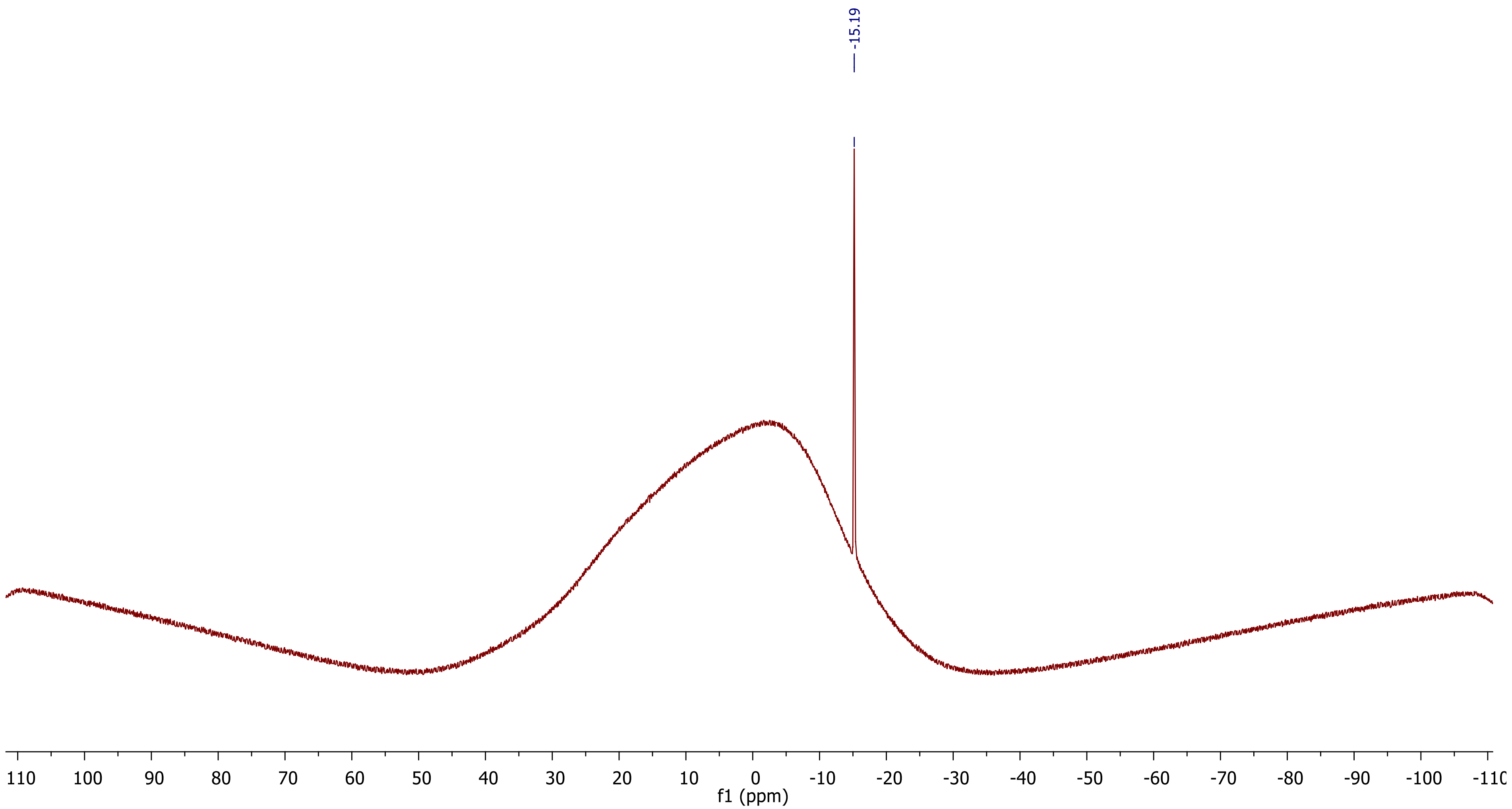

Figure S30. ^{11}B NMR spectrum (161 MHz, THF-d_8, 298K) of **5**.

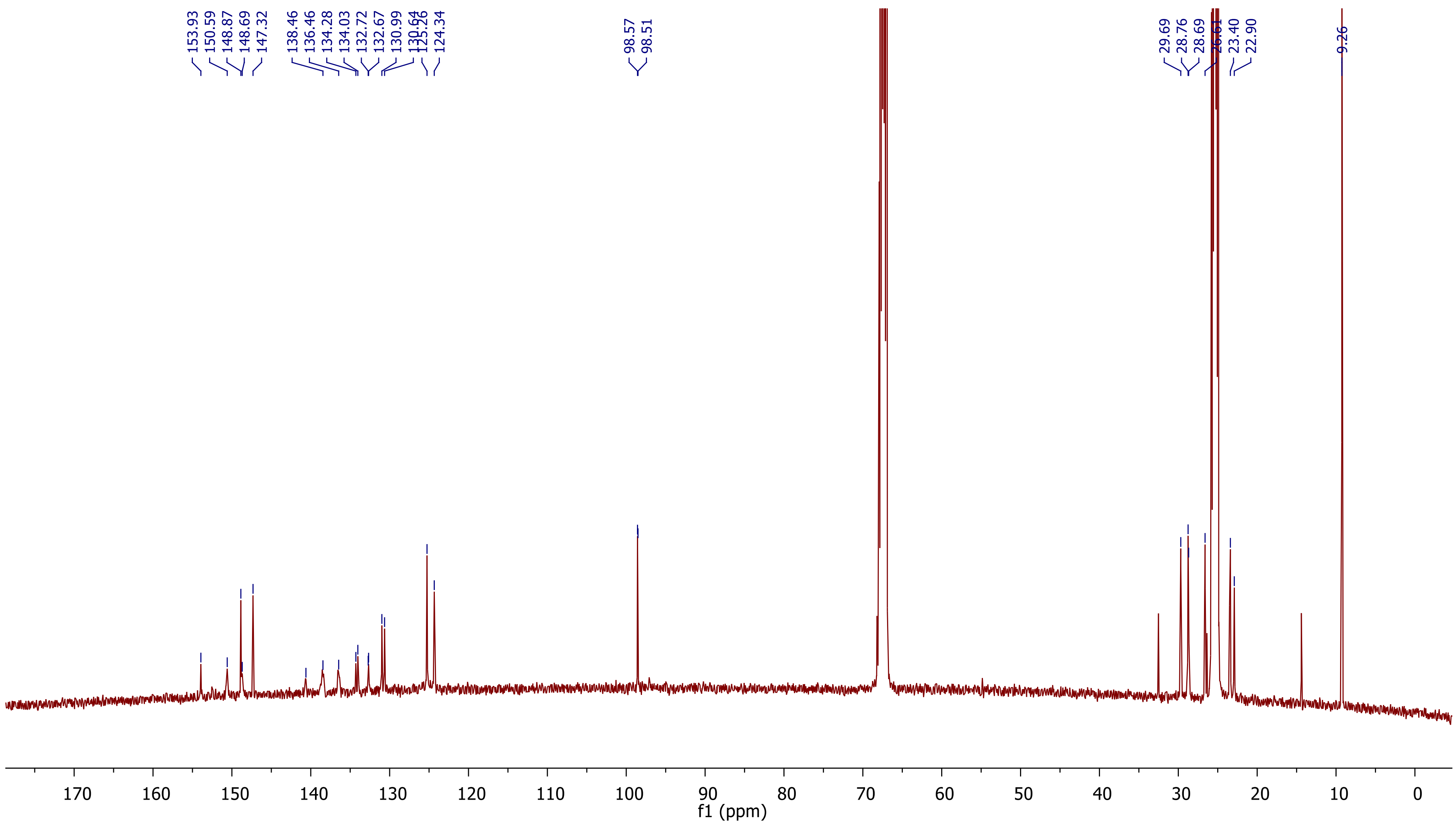

Figure S31. ^{13}C NMR spectrum (126 MHz, THF-d_8, 298K) of **5**.

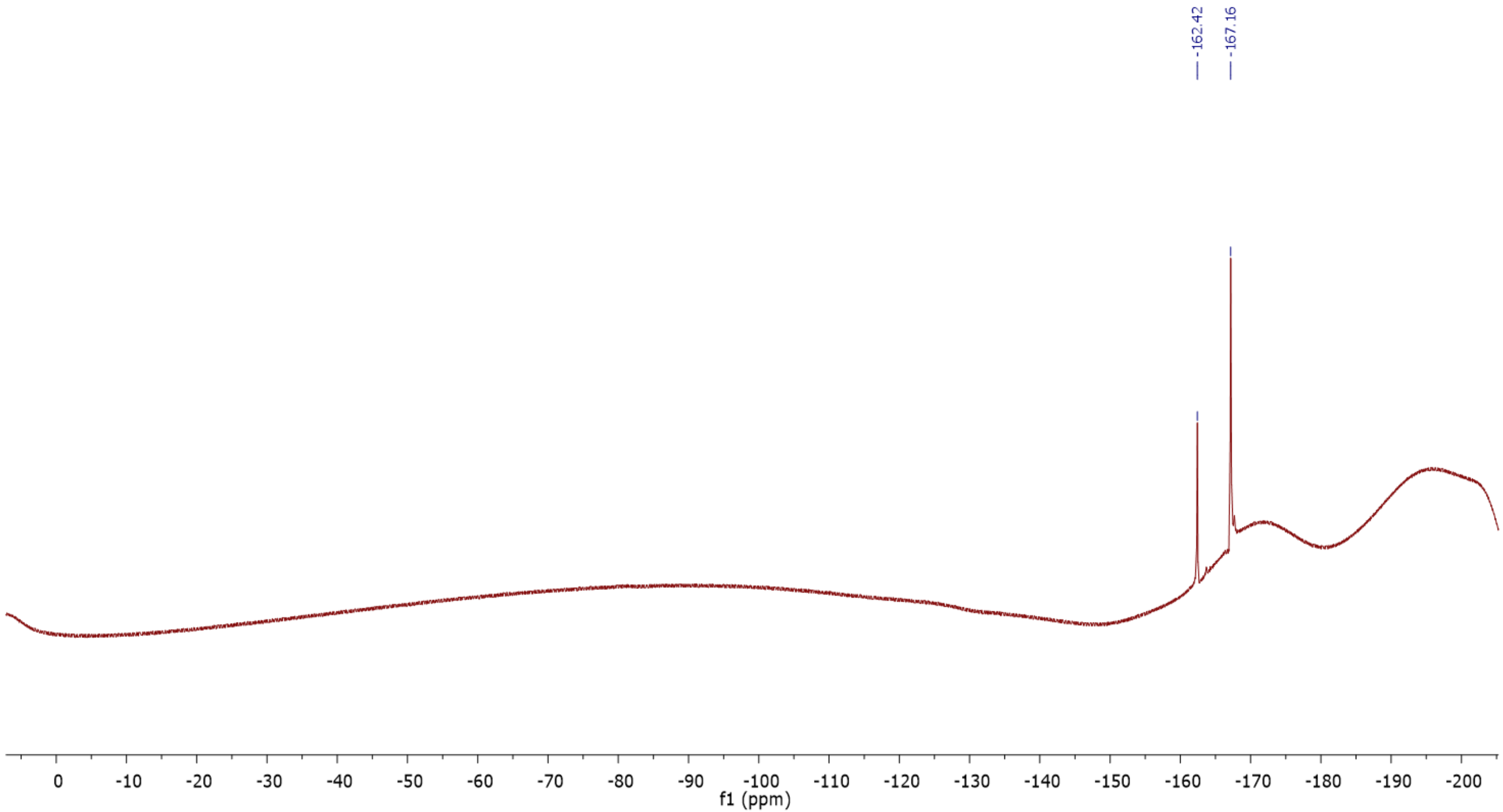

Figure S32. ^{19}F NMR spectrum (471 MHz, THF-d_8, 298K) of **5**.

S3.5 [{(WCA-IDipp)S}IrCl(η^5-C_5Me_5)] (**6**)

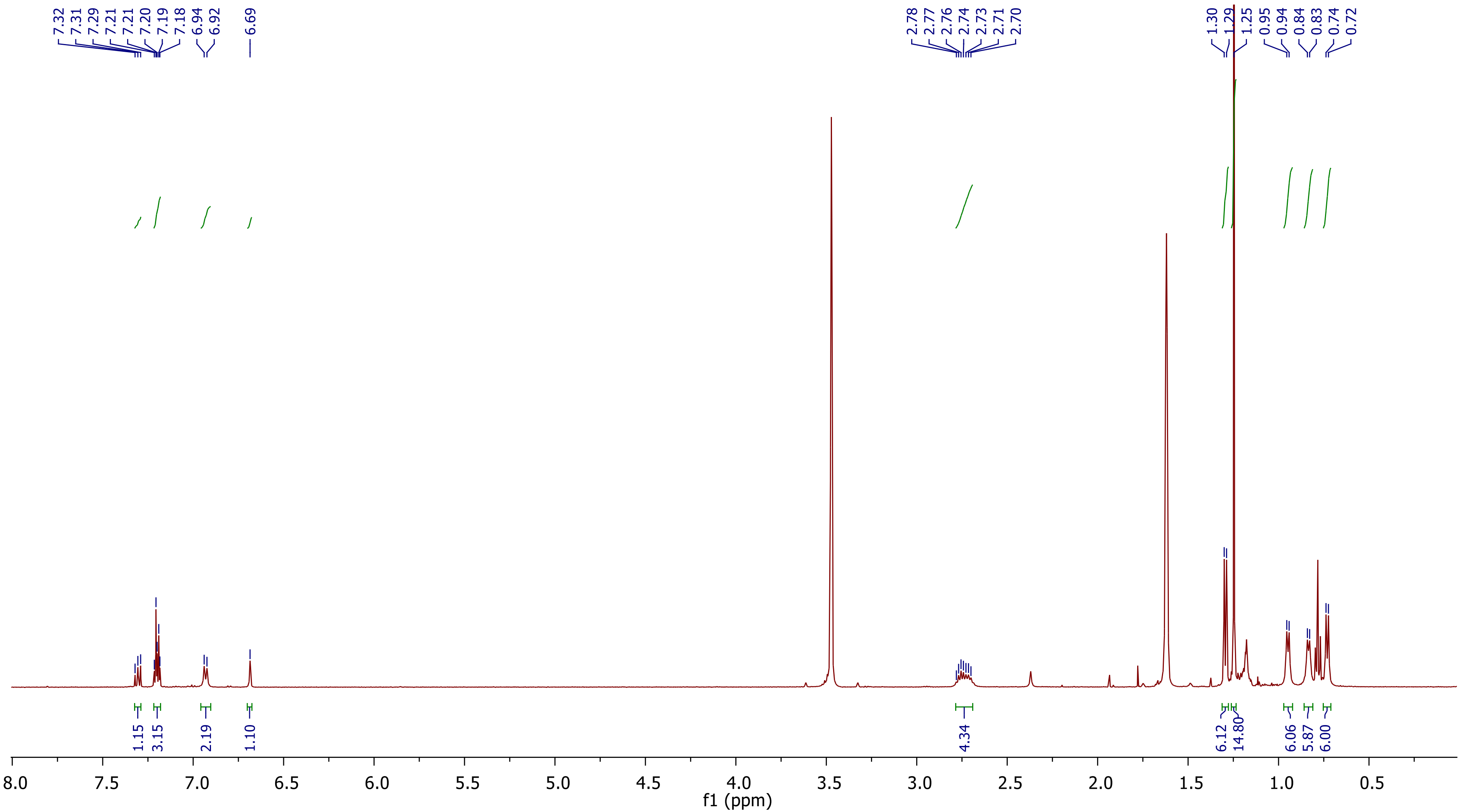

Figure S33. ^1H NMR spectrum (500 MHz, THF-d_8, 298K) of **6**.

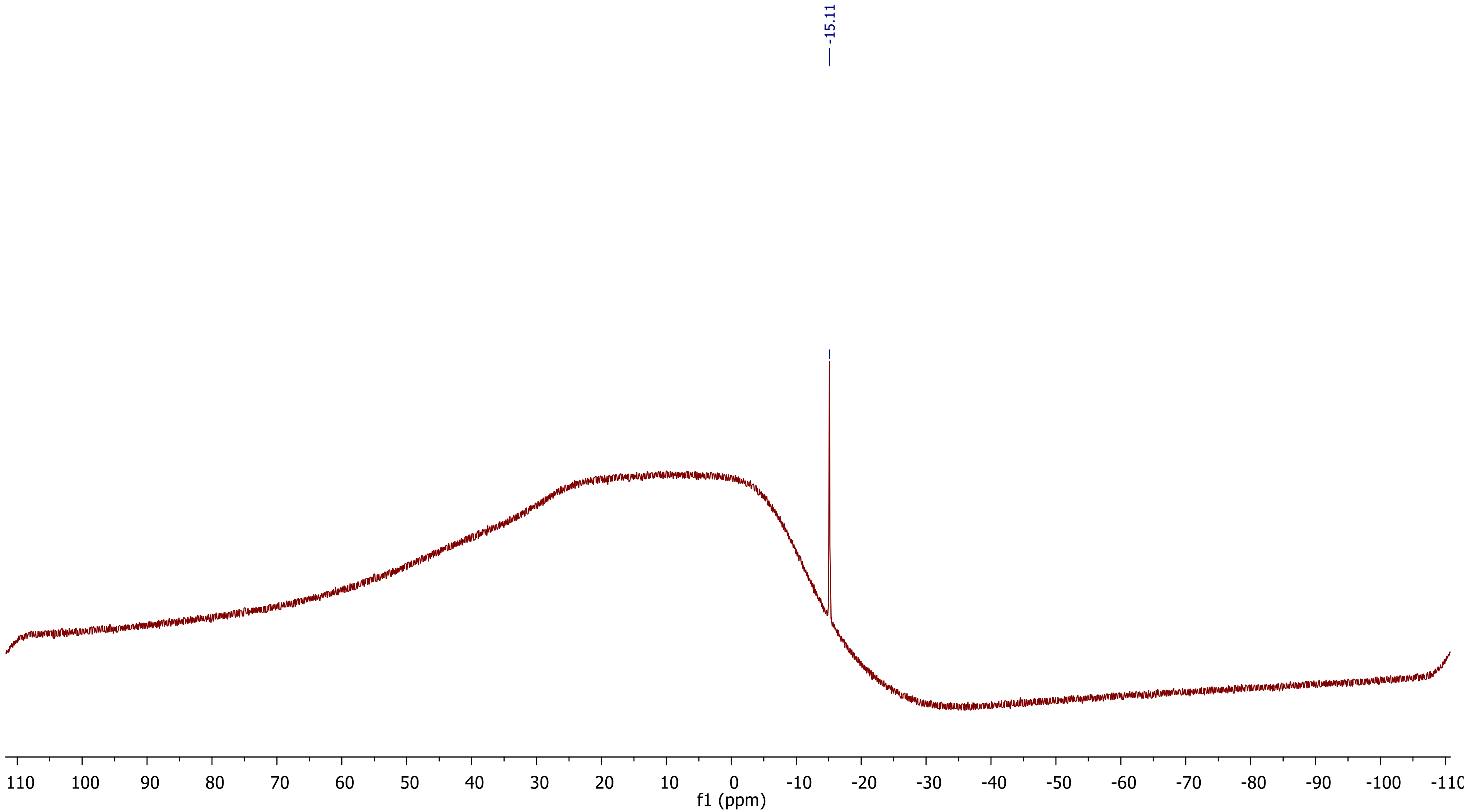

Figure S34. ^{11}B NMR spectrum (161 MHz, THF-d_8, 298K) of **6**.

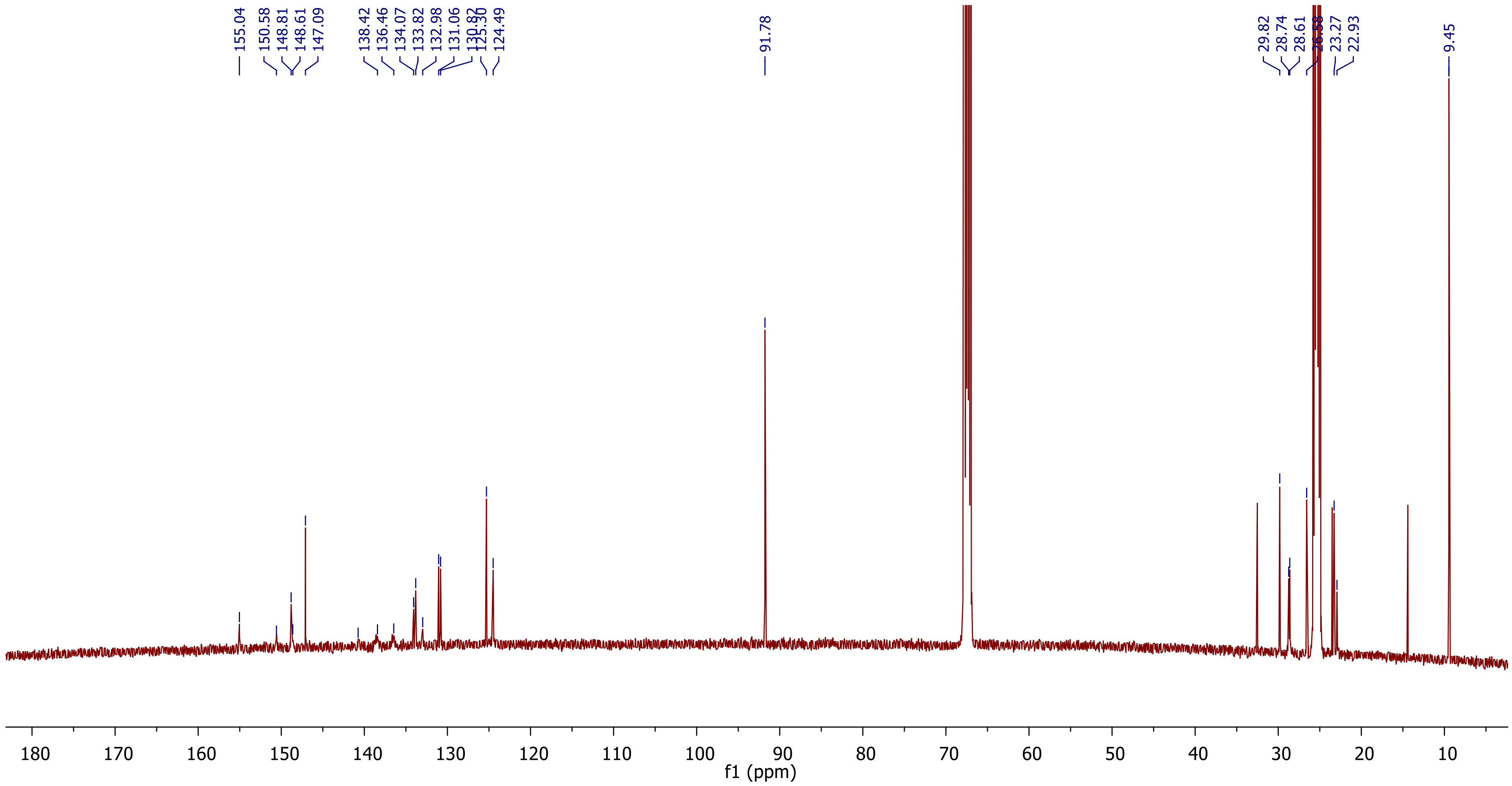

Figure S35. ^{13}C NMR spectrum (126 MHz, THF-d_8, 298K) of **6**.

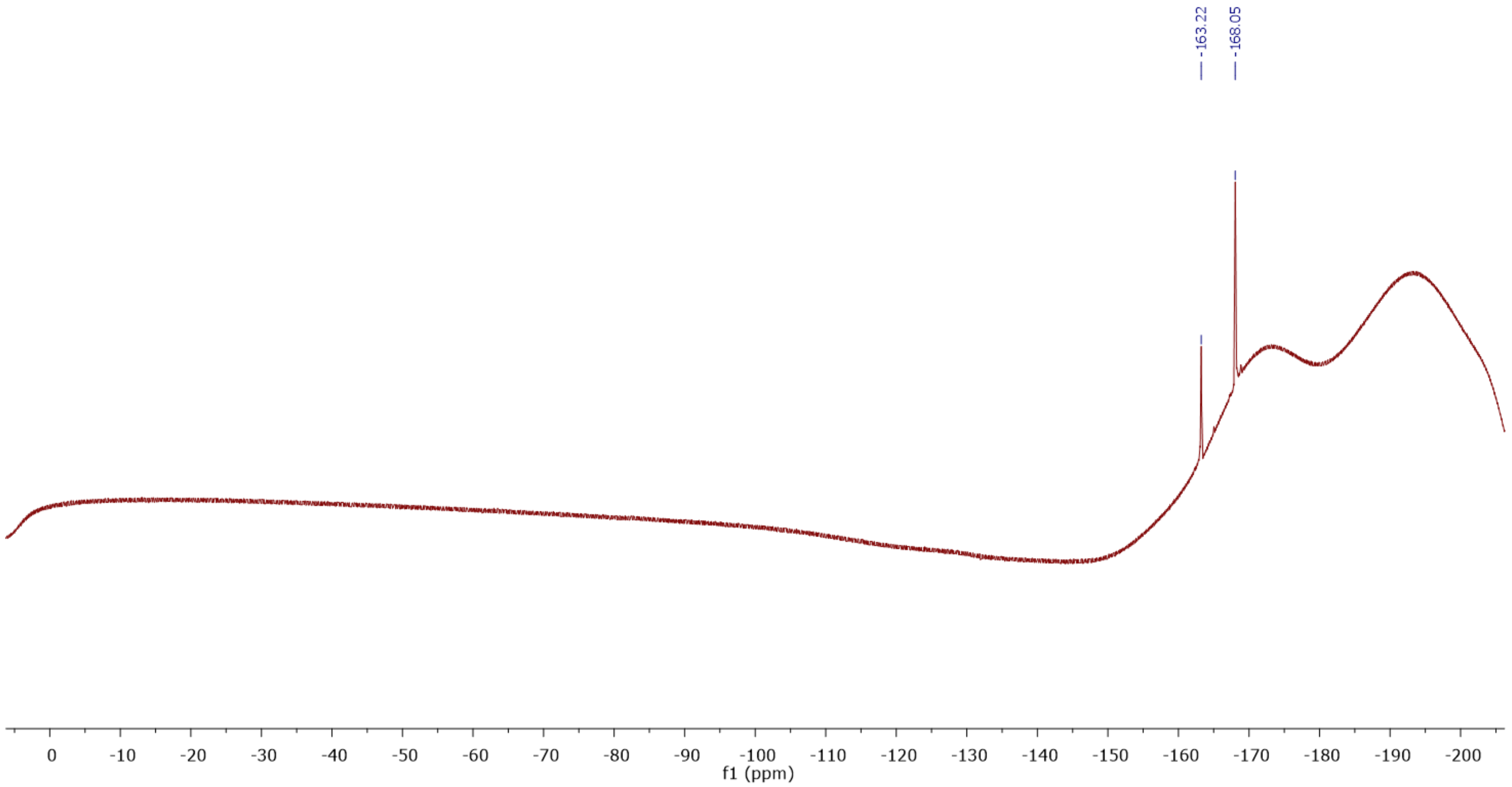

Figure S36. ^{19}F NMR spectrum (377 MHz, THF-d_8, 298K) of **6**.

S3.6 [{(WCA-IDipp)Se}RhCl(η^5-C_5Me_5)] (**7**)

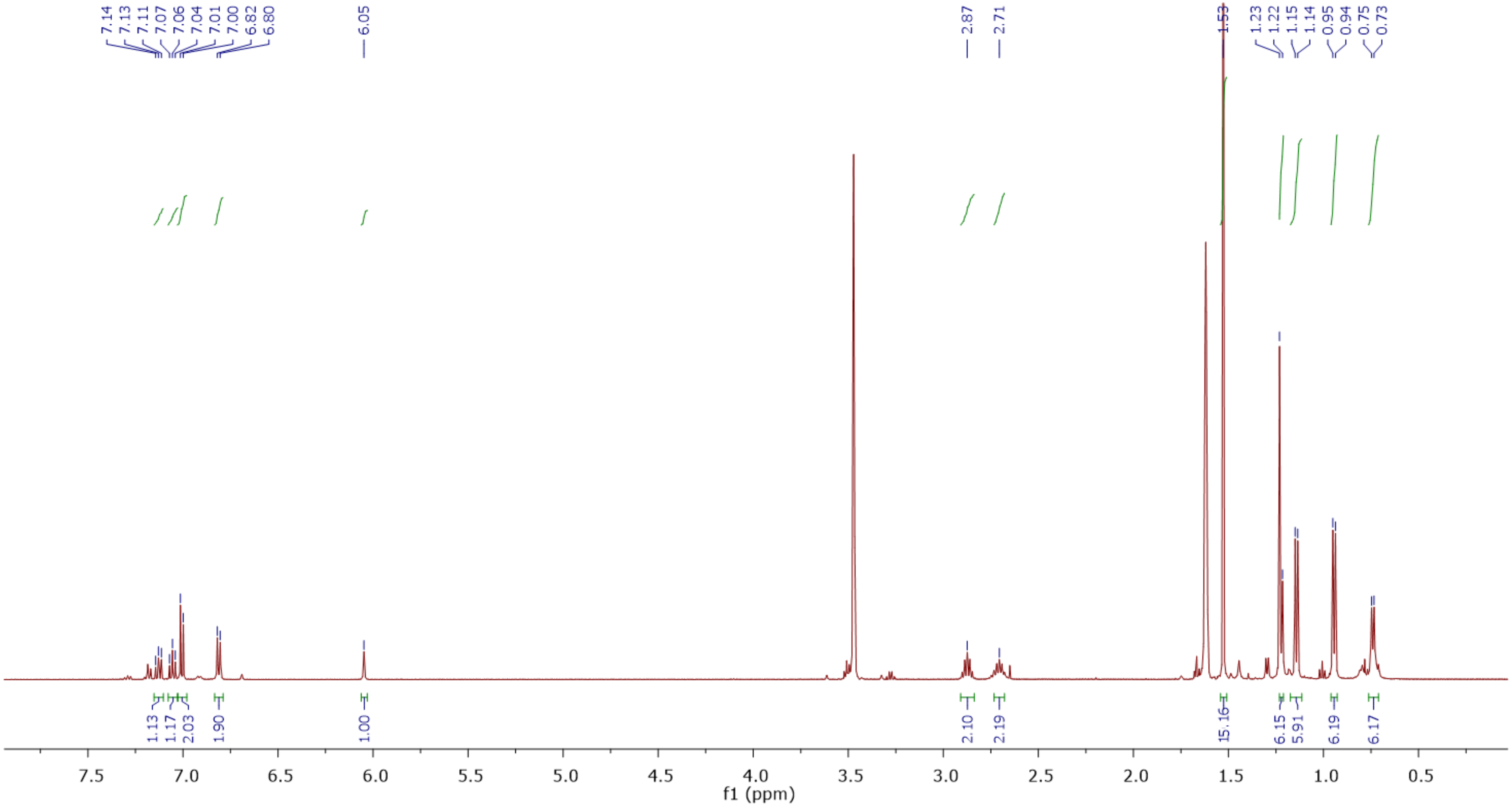

Figure S37. ^1H NMR spectrum (500 MHz, THF-d_8, 298K) of **7**.

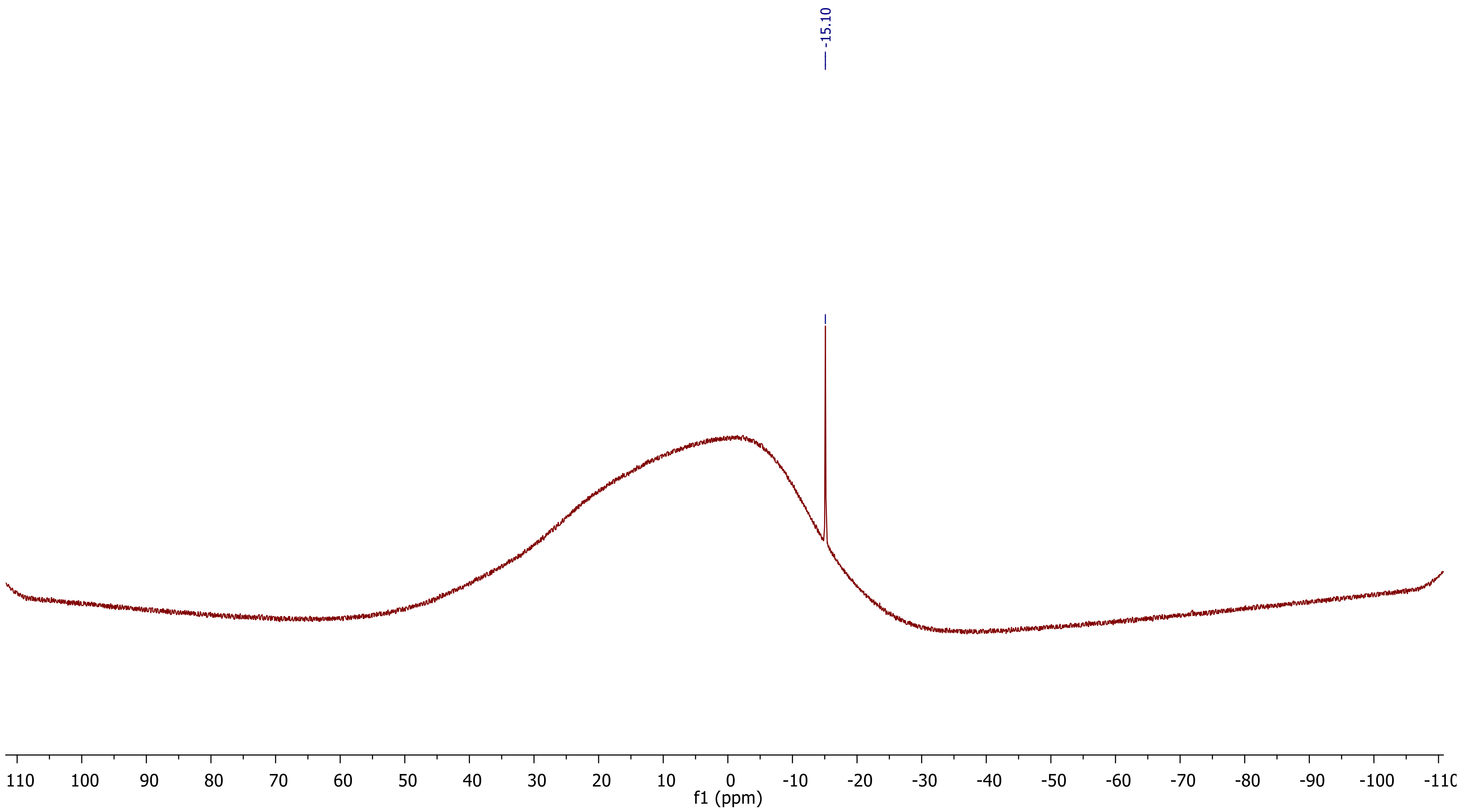

Figure S38. ^{11}B NMR spectrum (161 MHz, THF-d_8, 298K) of **7**.

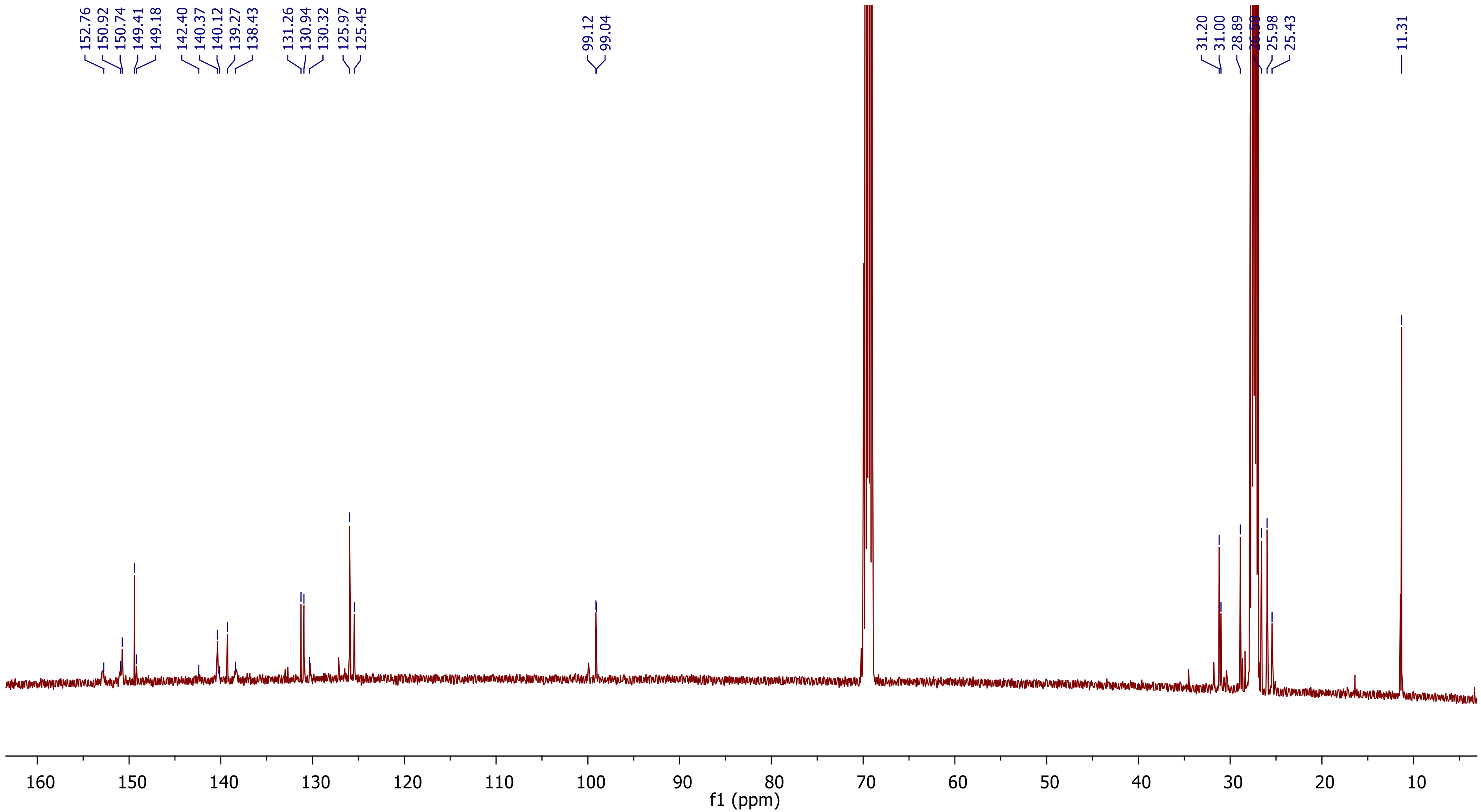

Figure S39. ^{13}C NMR spectrum (126 MHz, THF-d_8, 298K) of **7**.

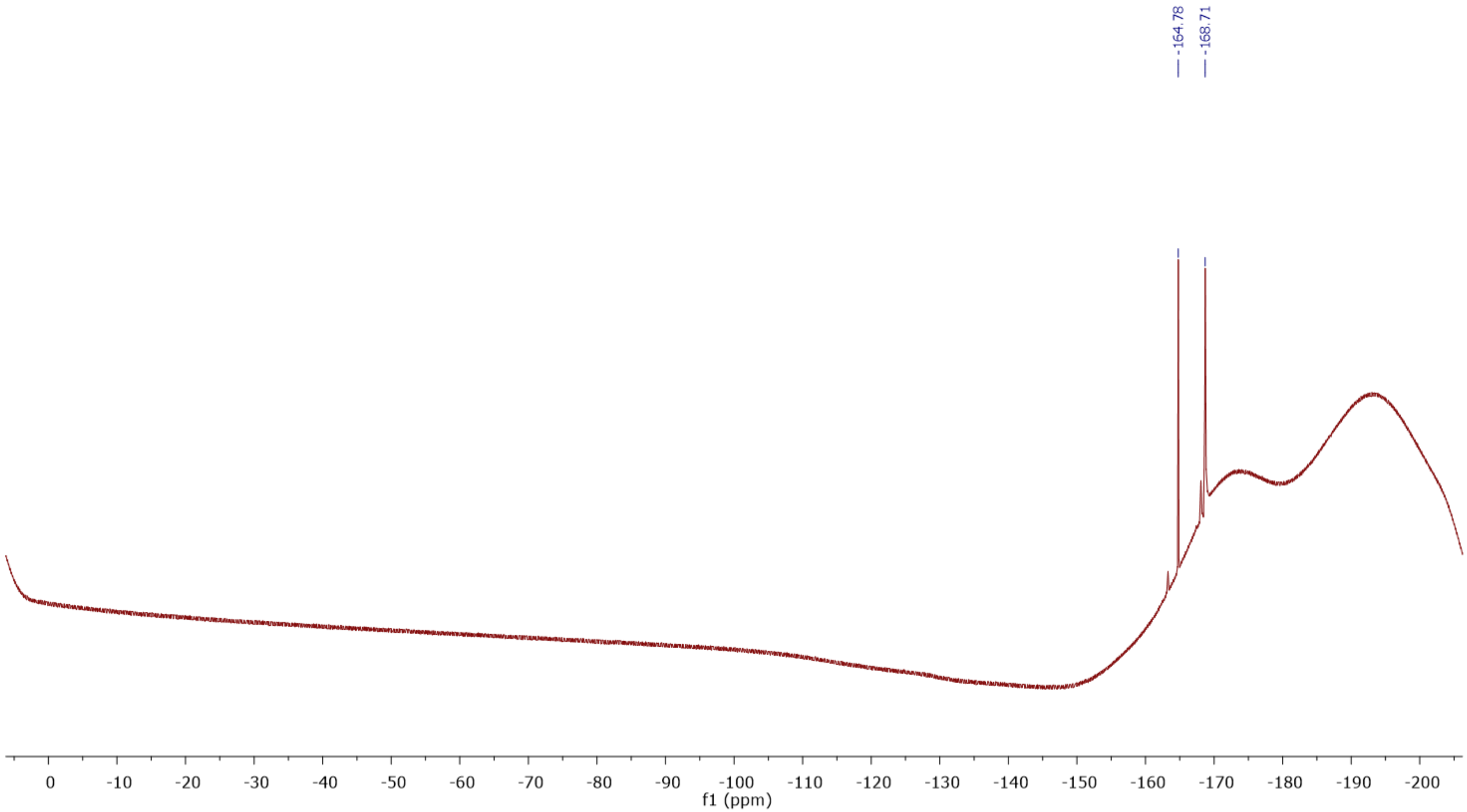

Figure S40. ^{19}F NMR spectrum (377 MHz, THF-d_8, 298K) of **7**.

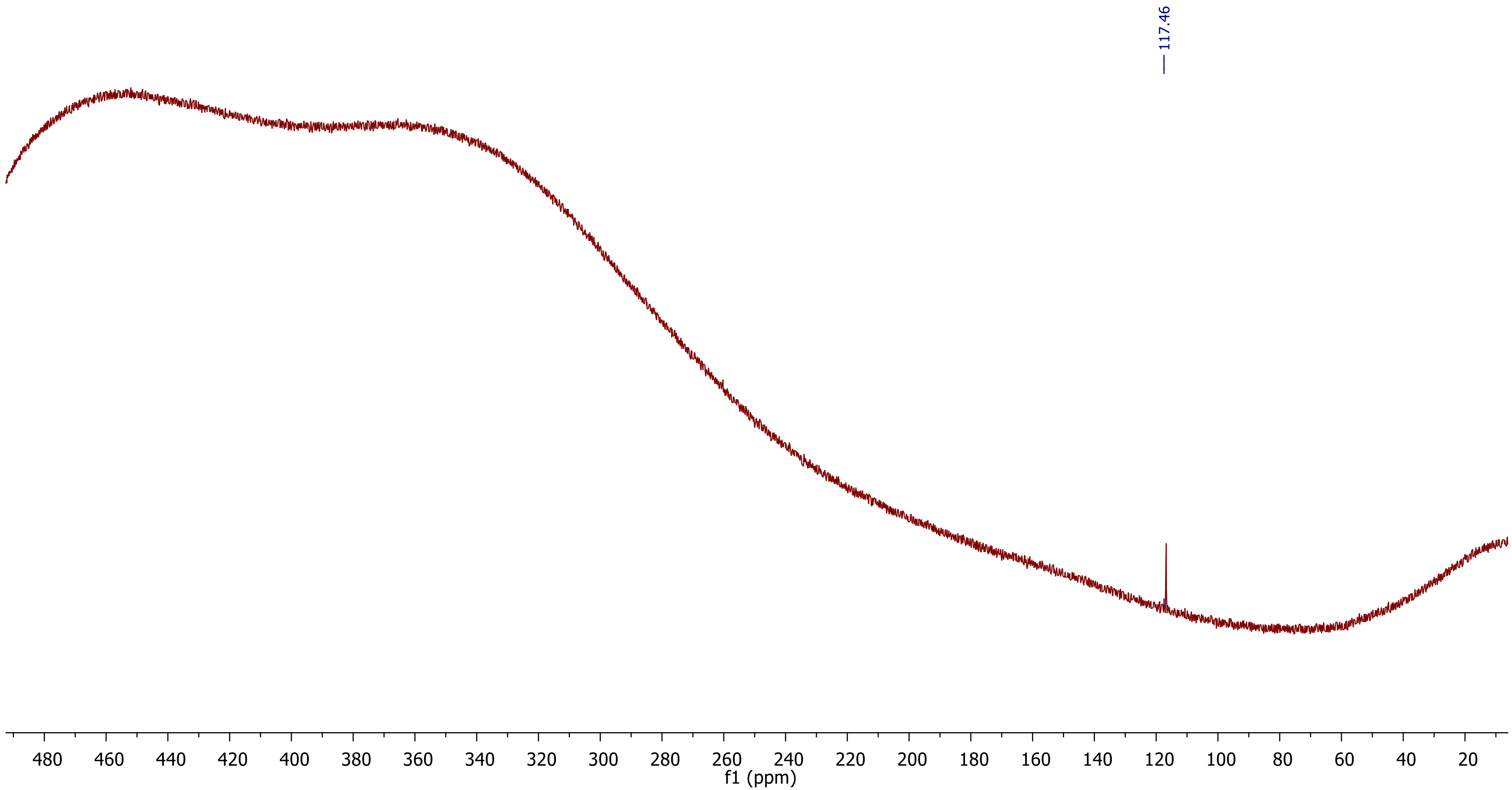

Figure S41. ^{77}Se NMR spectrum (95 MHz, THF-d_8, 298K) of **7**.

S3.7 [{(WCA-IDipp)Se}IrCl(η^5-C_5Me_5)] (**8**)

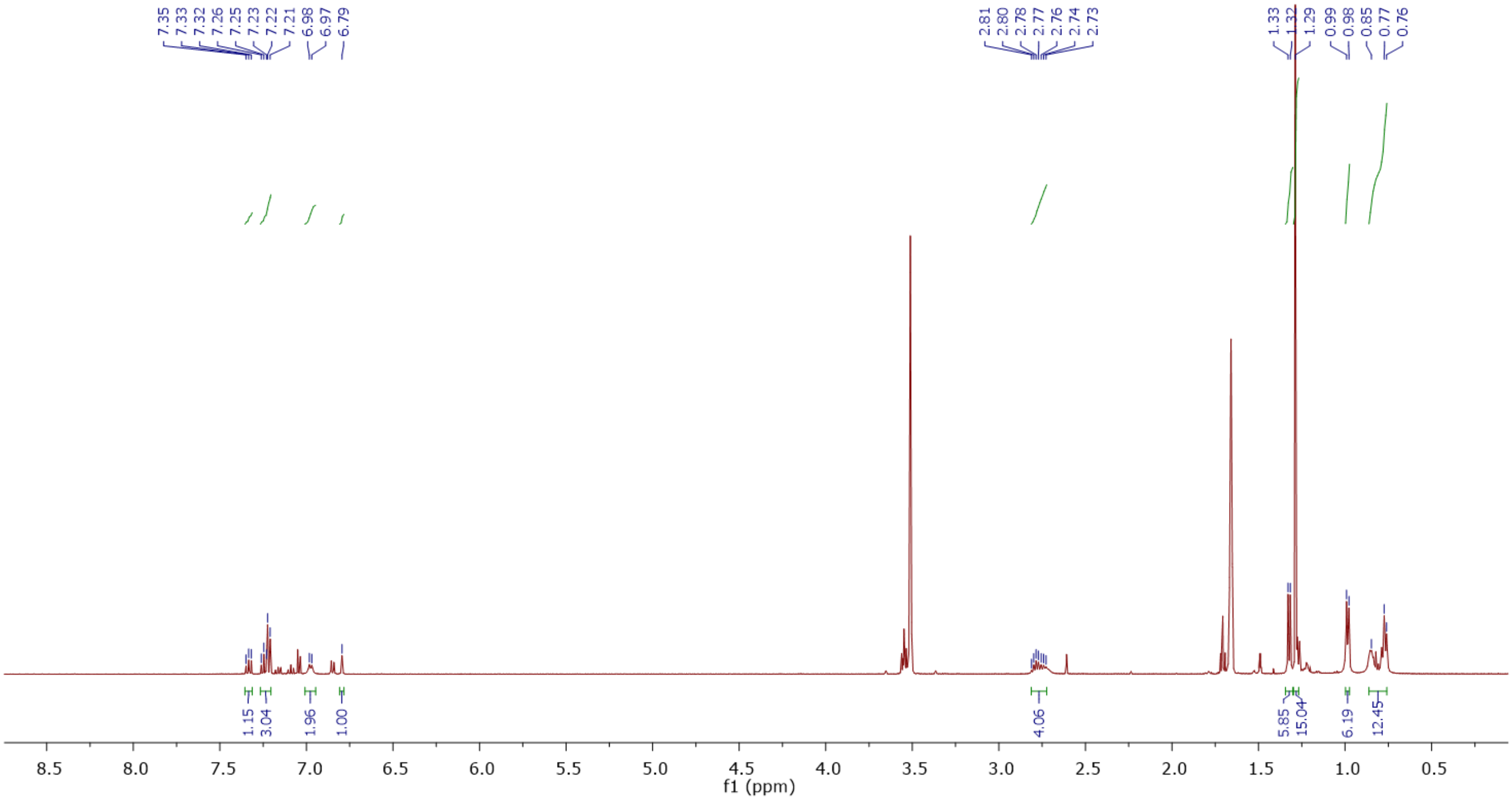

Figure S42. ^1H NMR spectrum (500 MHz, THF-d_8, 298K) of **8**.

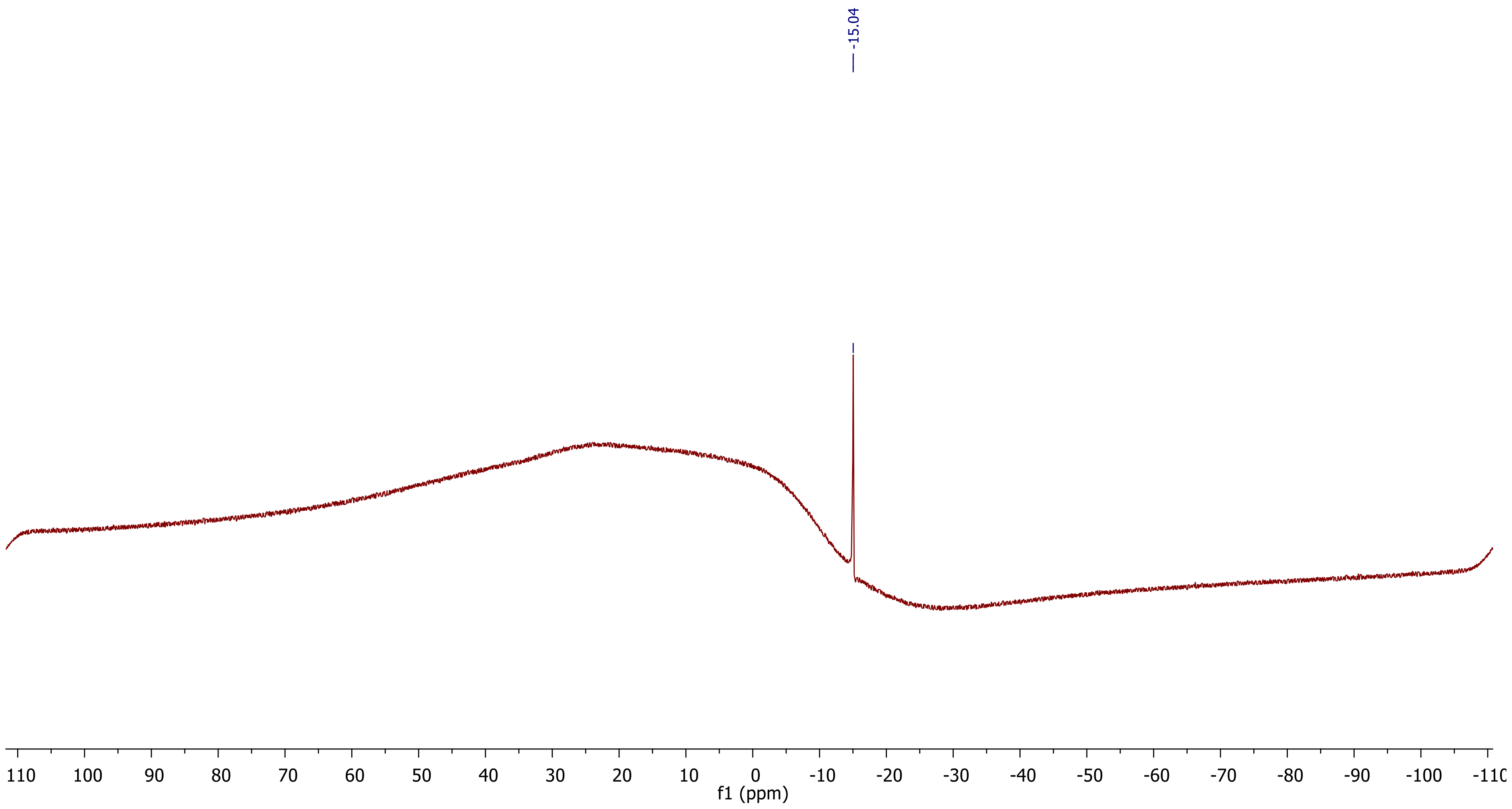

Figure S43. ^{11}B NMR spectrum (161 MHz, THF-d_8, 298K) of **8**.

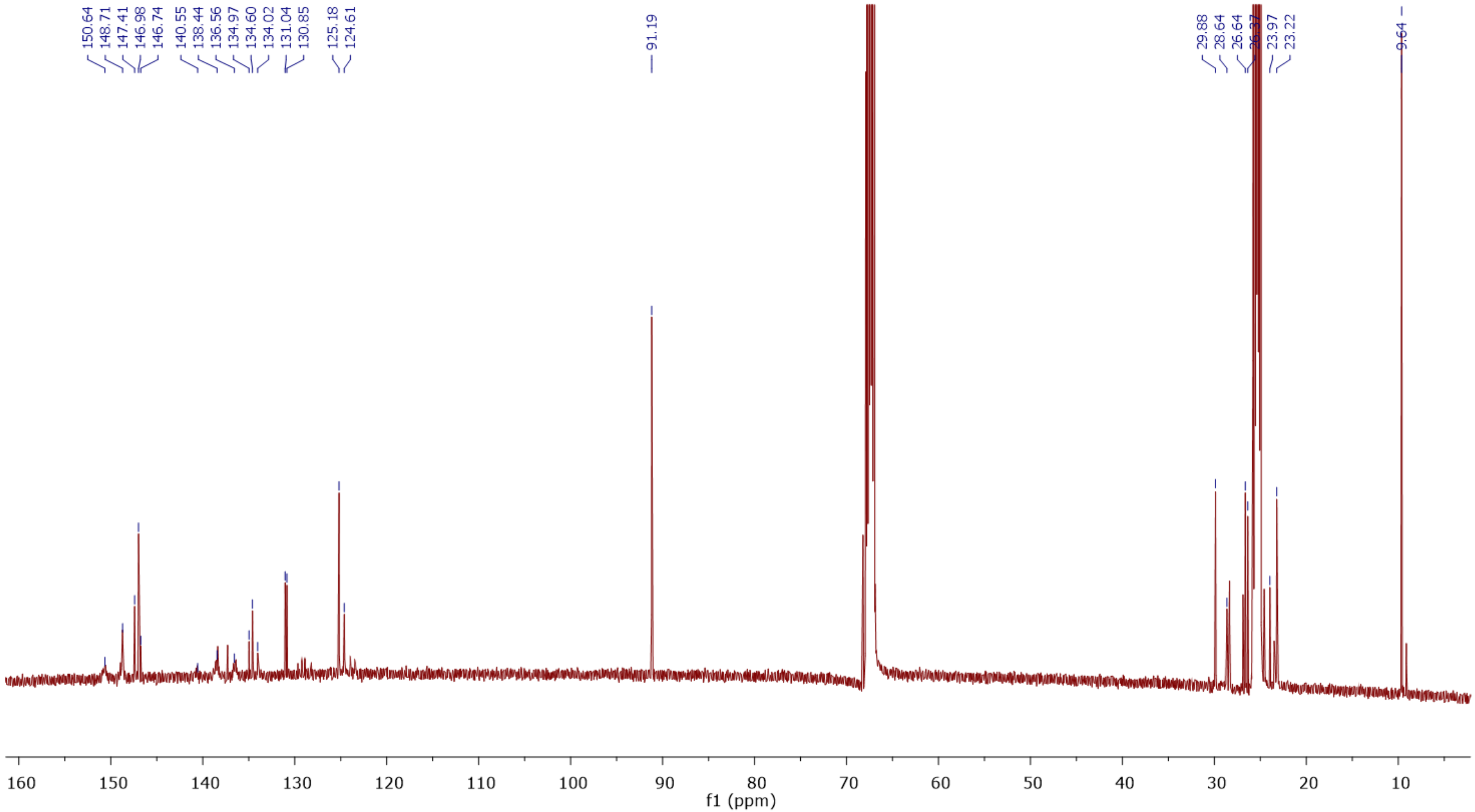

Figure S44. ^{13}C NMR spectrum (126 MHz, THF-d_8, 298K) of **8**.

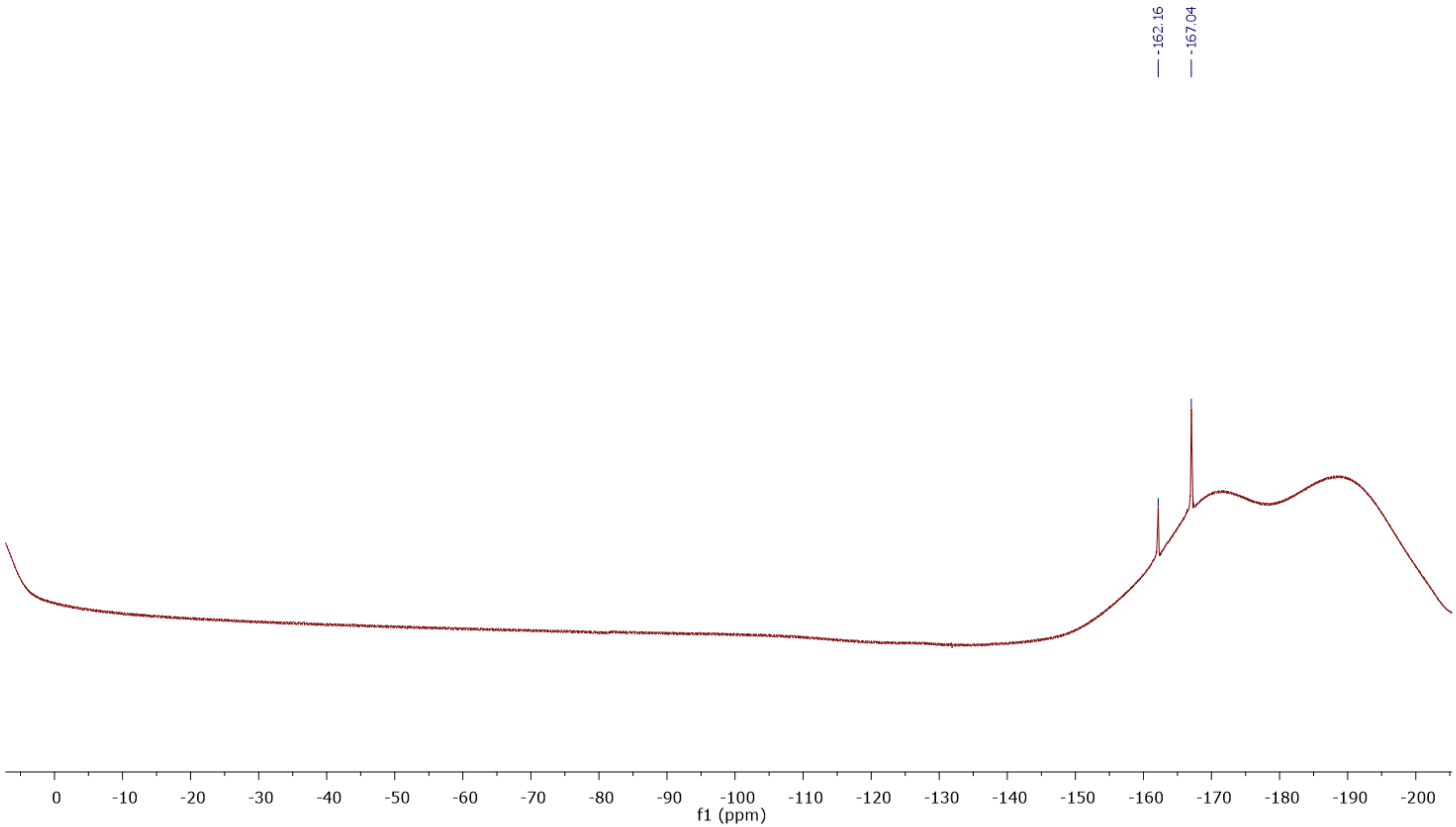

Figure S45. ^{19}F NMR spectrum (471 MHz, THF-d_8, 298K) of **8**.

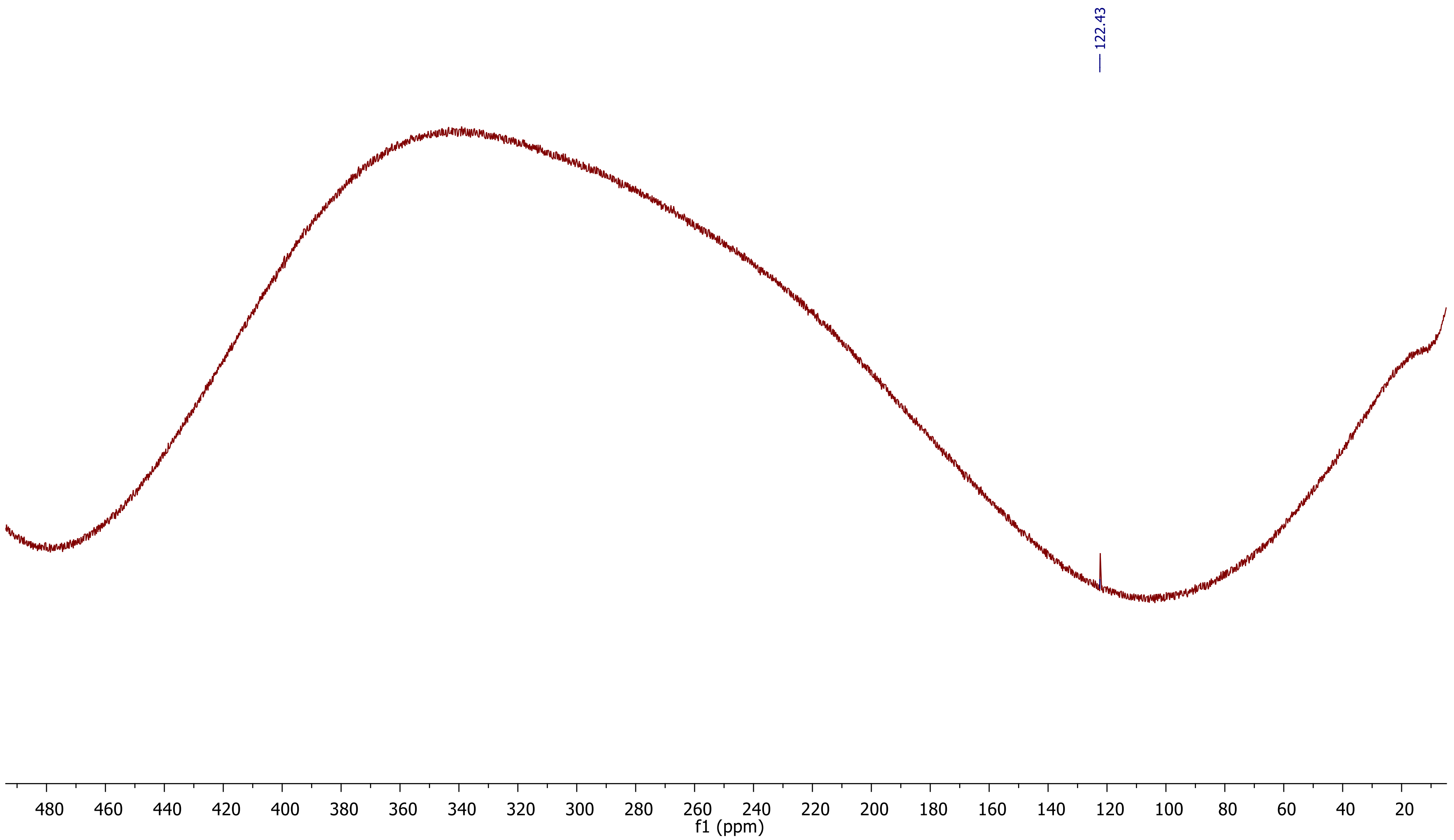

Figure S46. ^{77}Se NMR spectrum (95 MHz, THF-d_8, 298K) of **8**.

S3.8 [{(WCA-IDipp)S}Rh(COD)] (**9**)

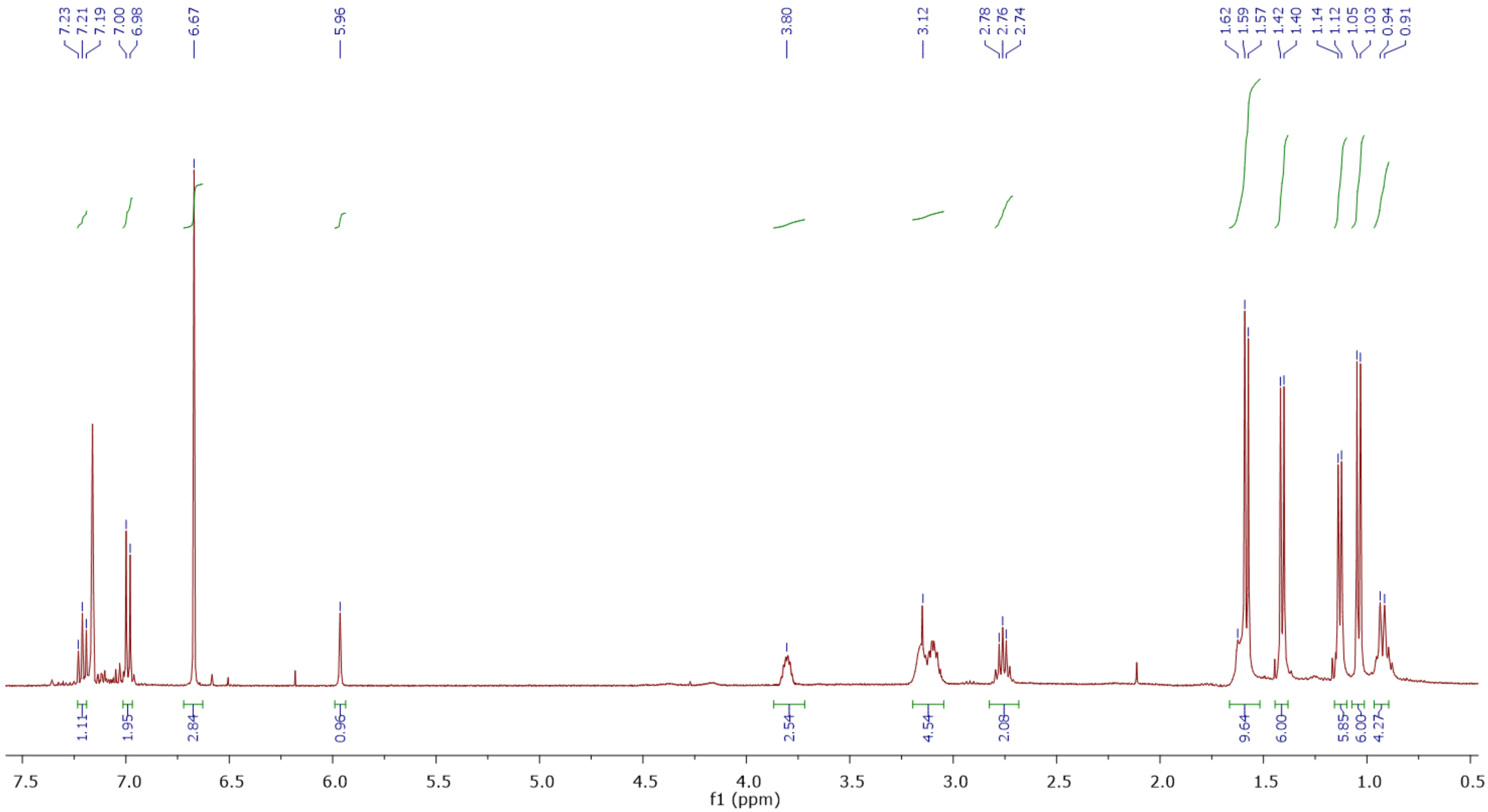

Figure S47. ^{1}H NMR spectrum (400 MHz, C_6D_6, 298K) of **9**.

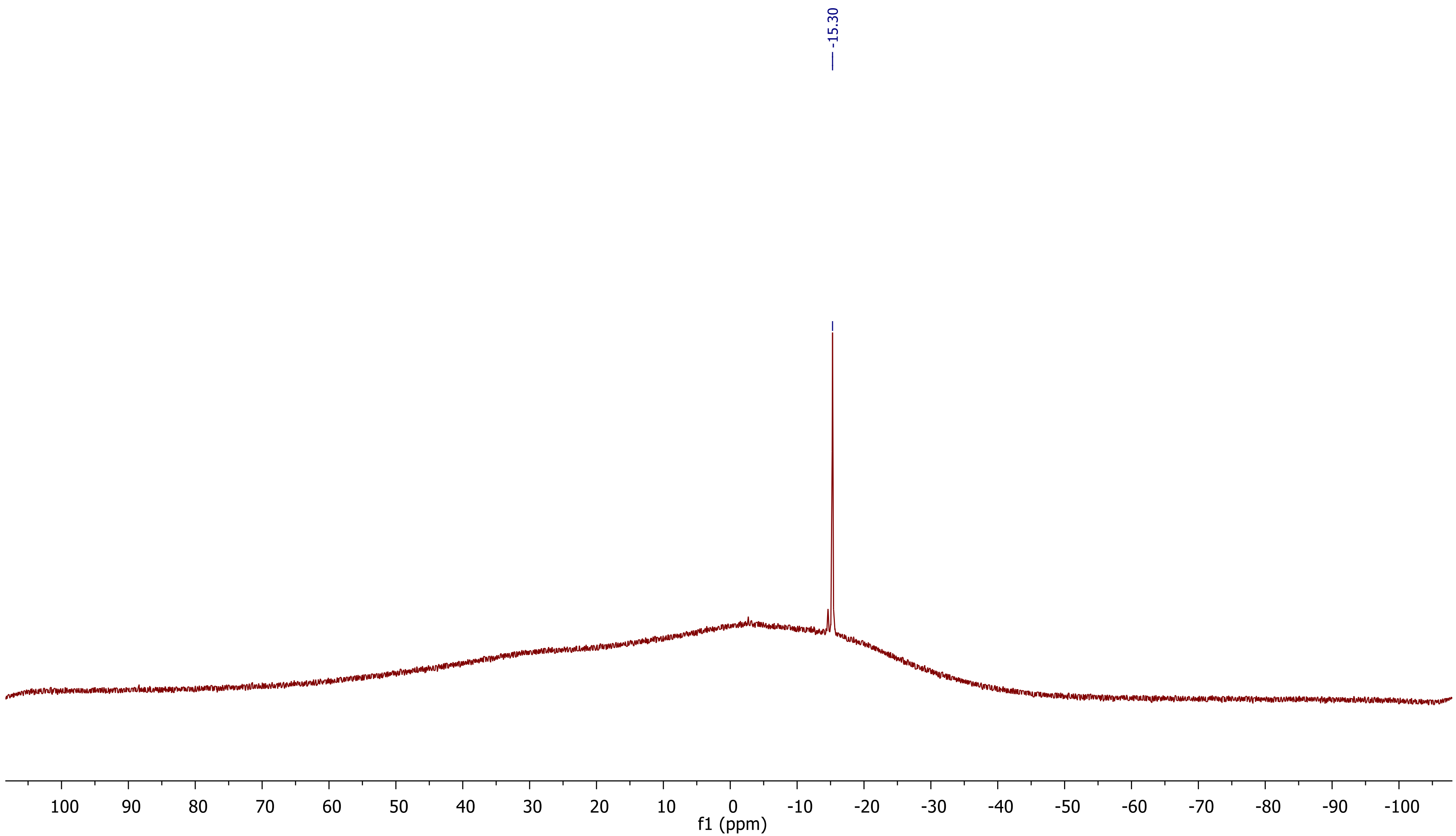

Figure S48. ^{11}B NMR spectrum (128 MHz, C_6D_6, 298K) of **9**.

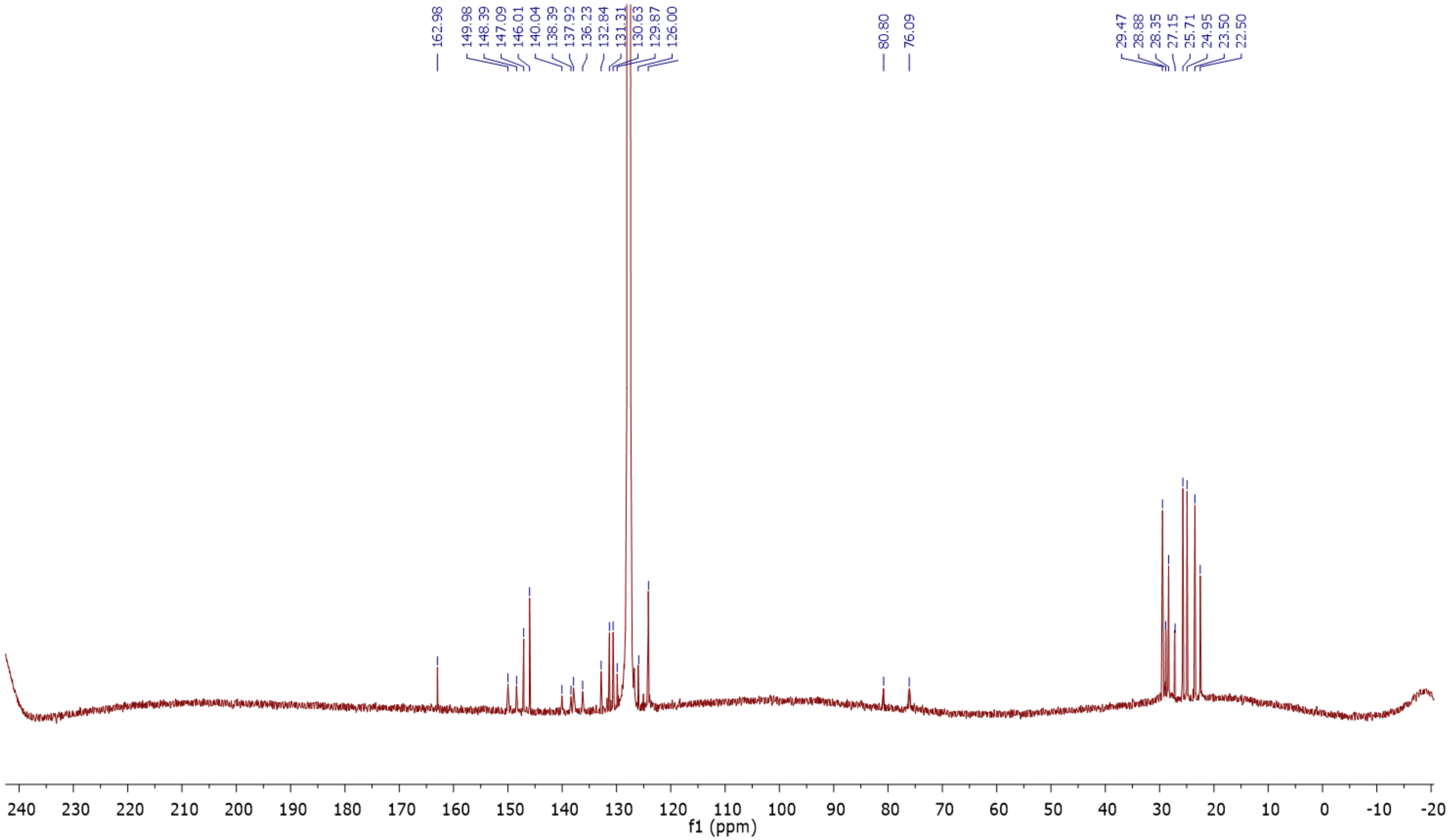

Figure S49. ^{13}C NMR spectrum (151 MHz, C_6D_6, 298K) of **9**.

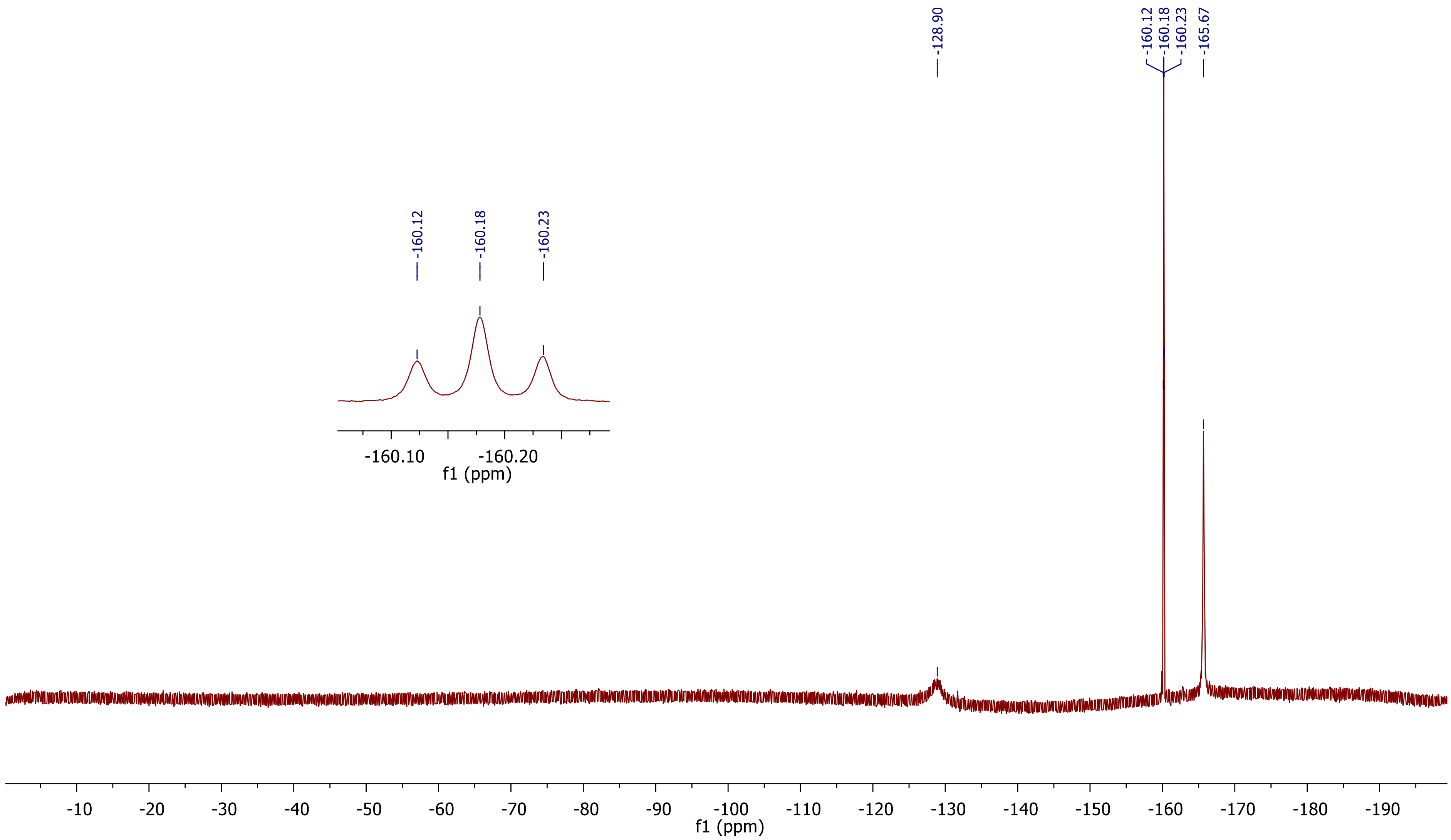

Figure S50. ^{19}F NMR spectrum (377 MHz, C_6D_6, 298K) of **9**.

S3.9 [{(WCA-IDipp)S}Ir(COD)] (**10**)

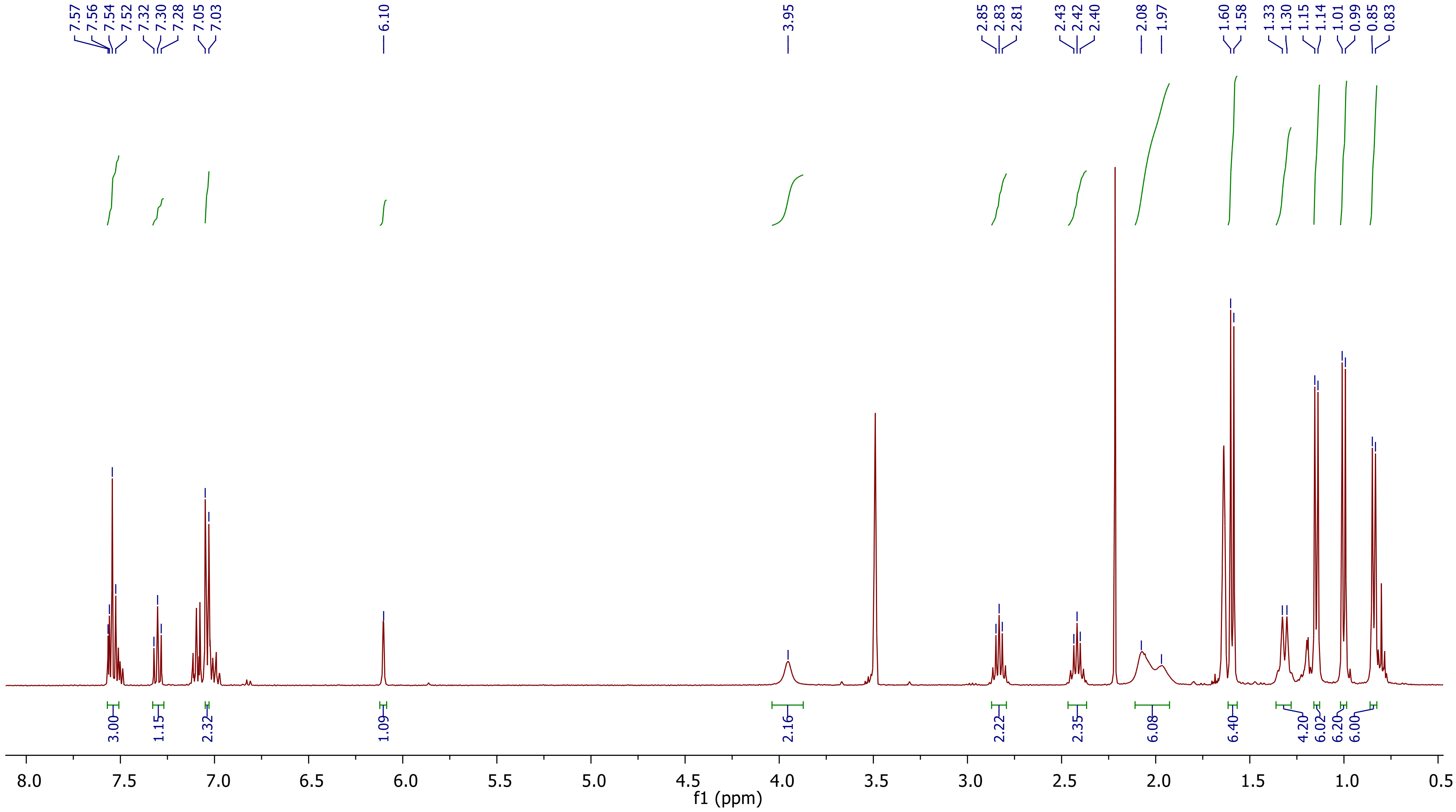

Figure S51. ^{1}H NMR spectrum (400 MHz, THF-d_8, 298K) of **10**.

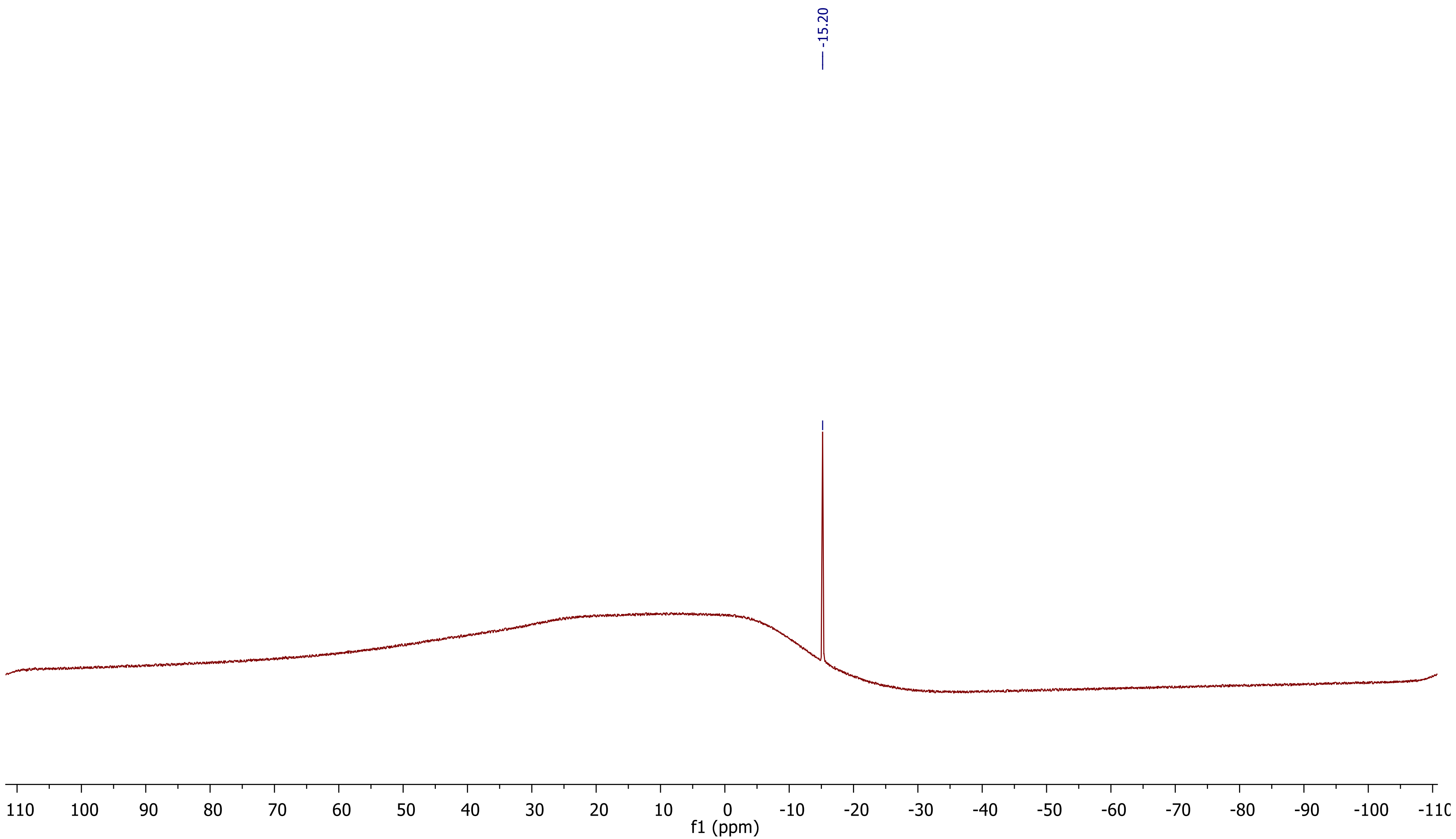

Figure S52. ^{11}B NMR spectrum (161 MHz, THF-d_8, 298K) of **10**.

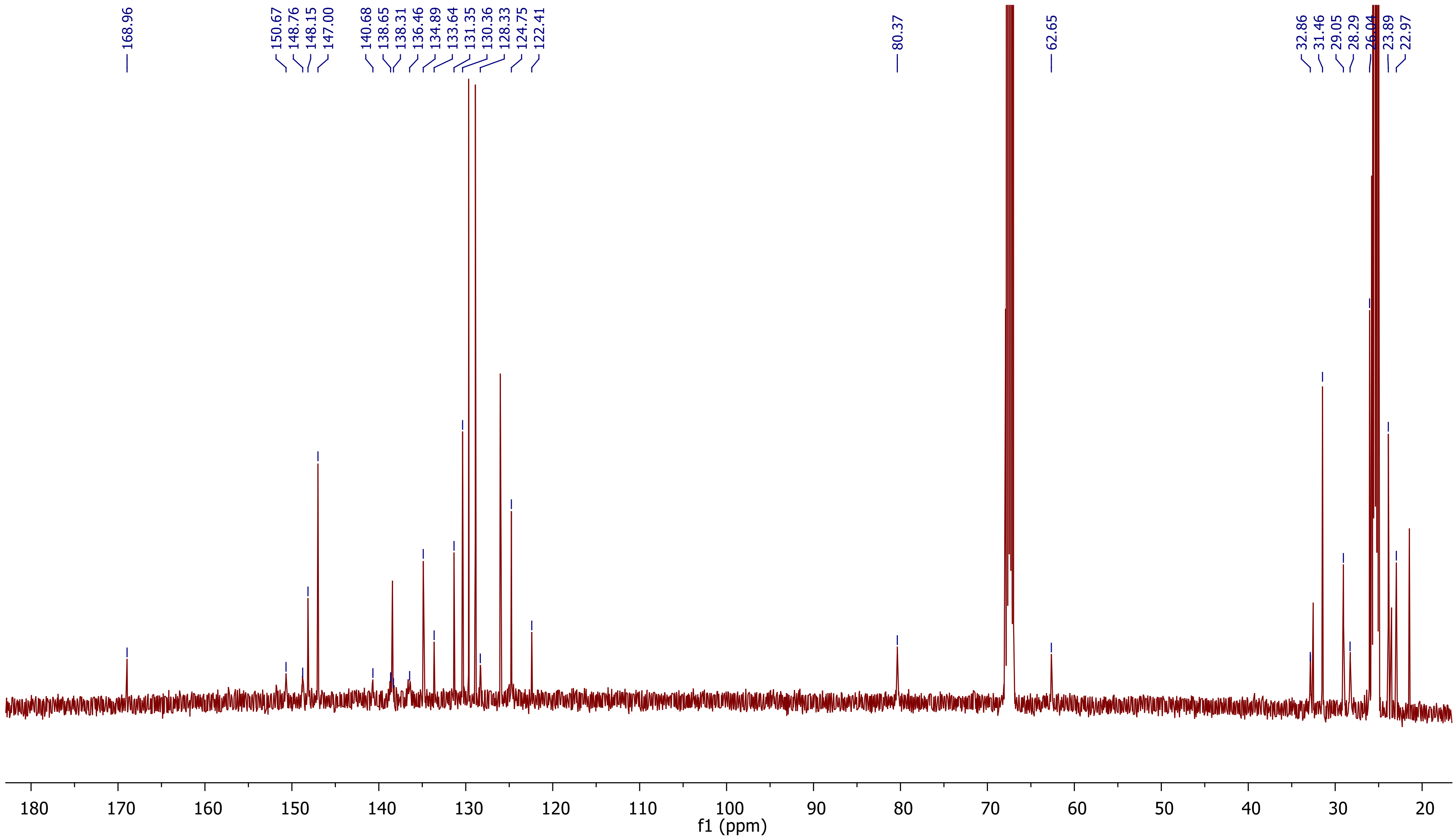

Figure S53. ^{13}C NMR spectrum (126 MHz, THF-d_8, 298K) of **10**.

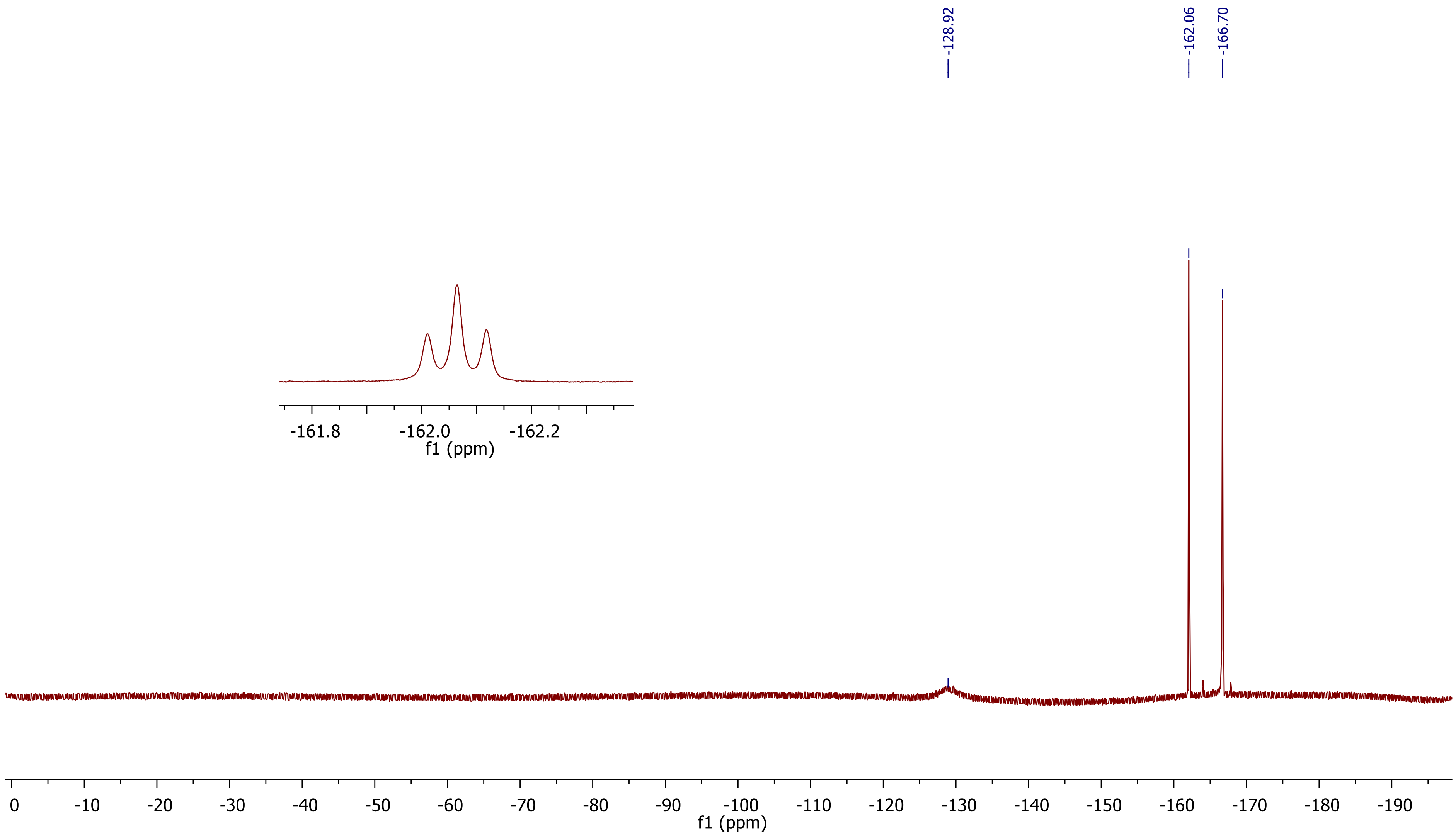

Figure S54. ^{19}F NMR spectrum (471 MHz, THF-d_8, 298K) of **10**.

S3.9 [{(WCA-IDipp)Se}Rh(COD)] (**11**)

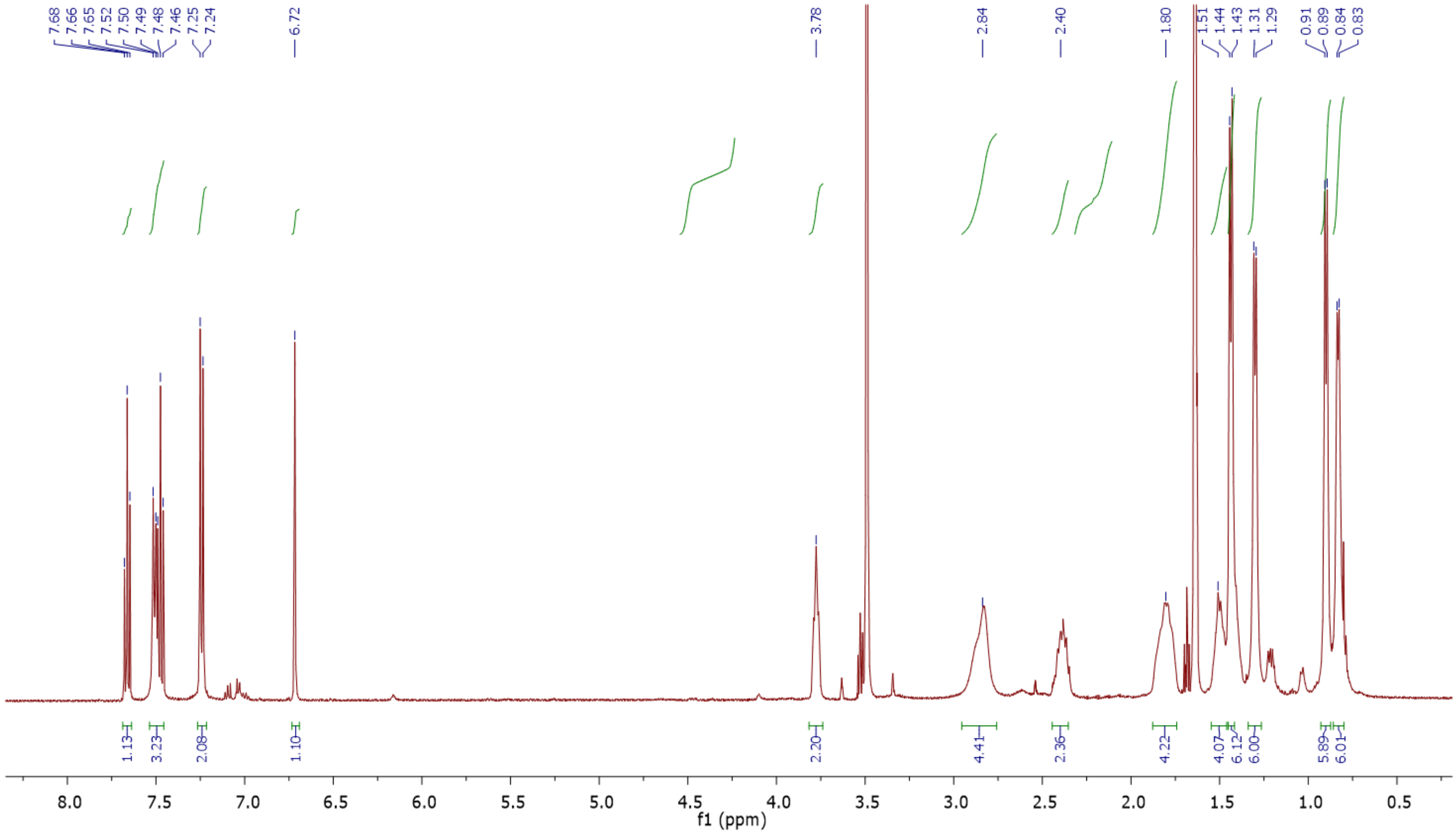

Figure S55. ^{1}H NMR spectrum (500 MHz, THF-d_8, 298K) of **11**.

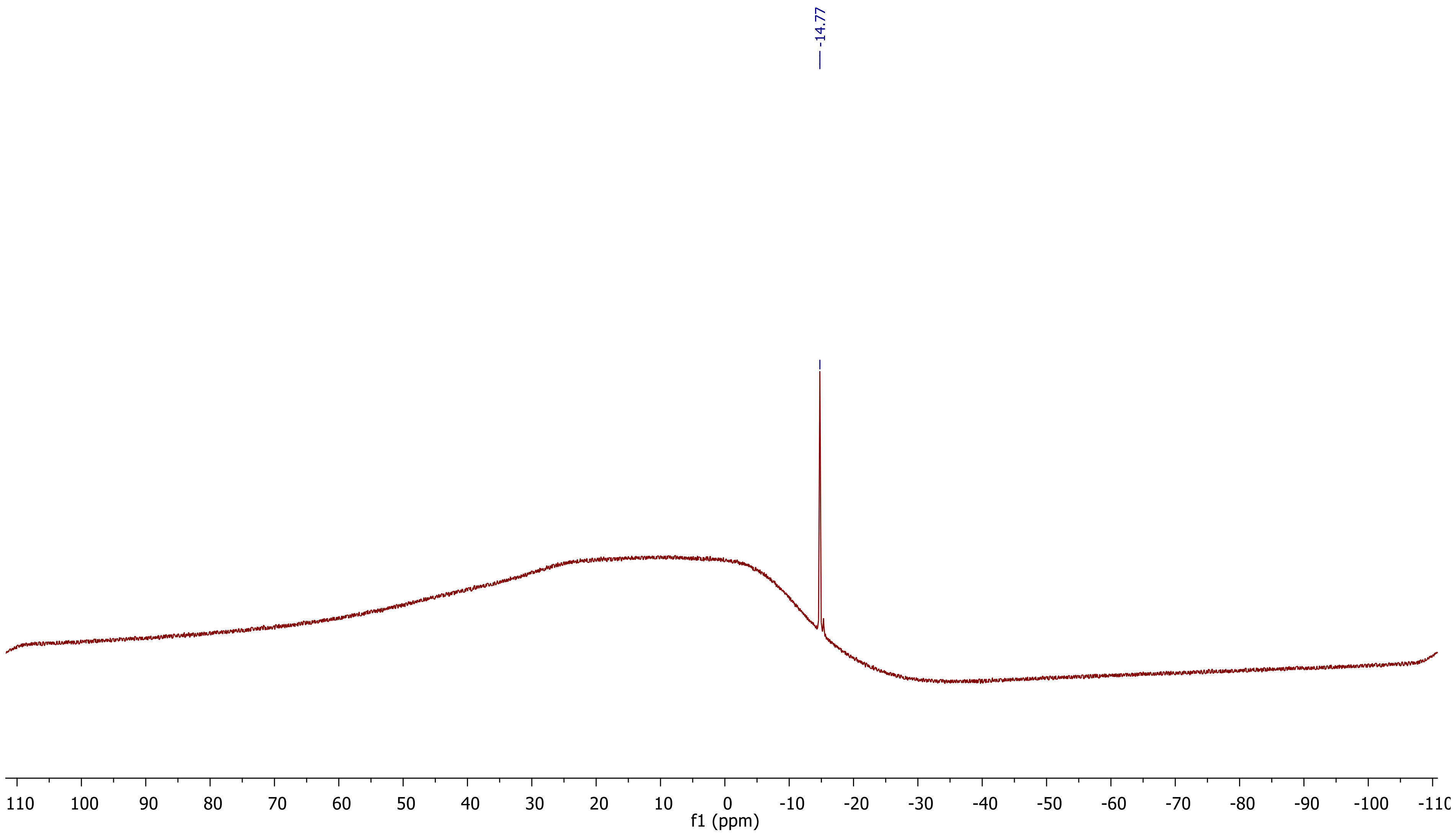

Figure S56. ^{11}B NMR spectrum (161 MHz, THF-d_8, 298K) of **11**.

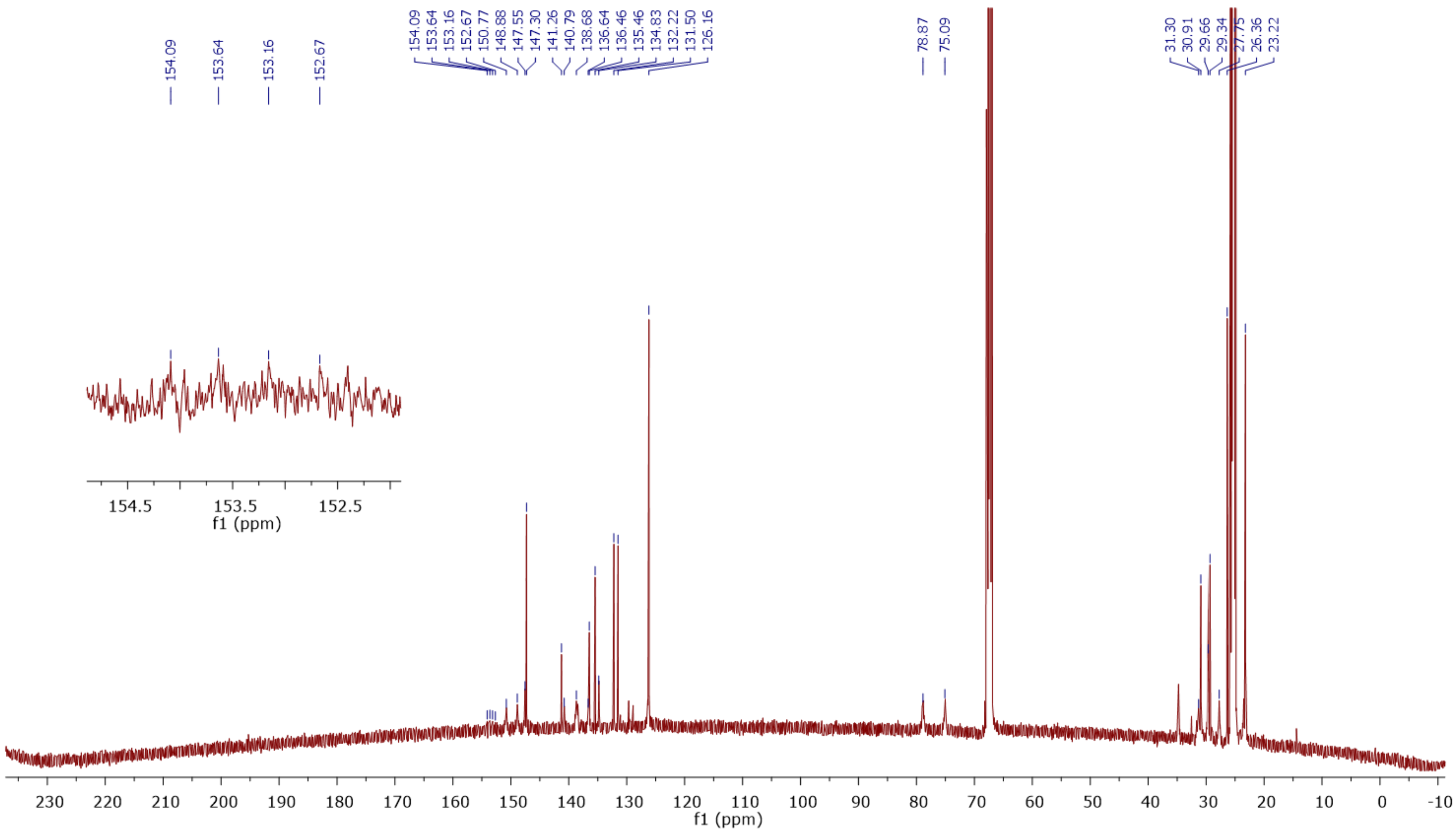

Figure S57. ^{13}C NMR spectrum (126 MHz, THF-d_8, 298K) of **11**.

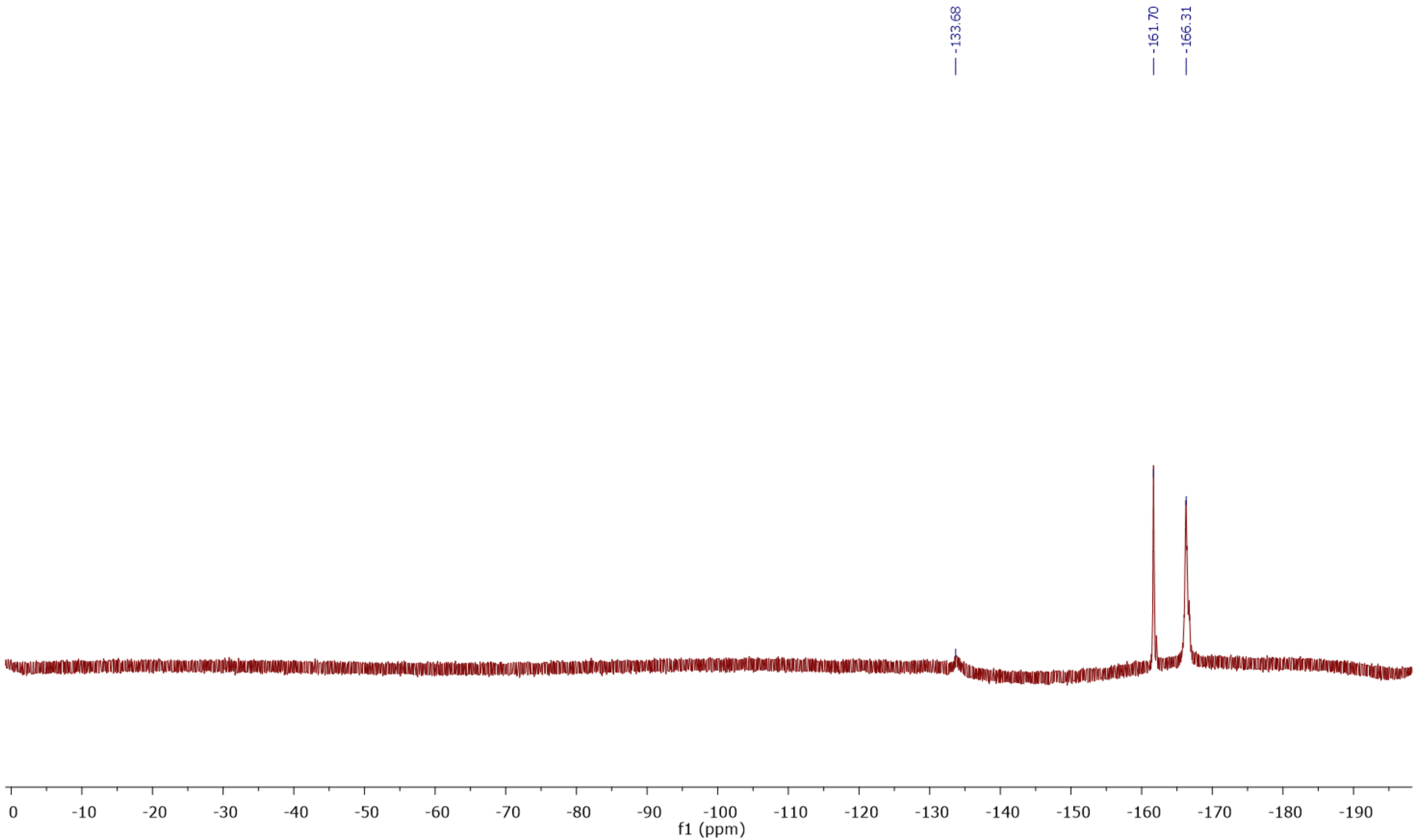

Figure S58. ^{19}F NMR spectrum (377 MHz, THF-d_8, 298K) of **11**.

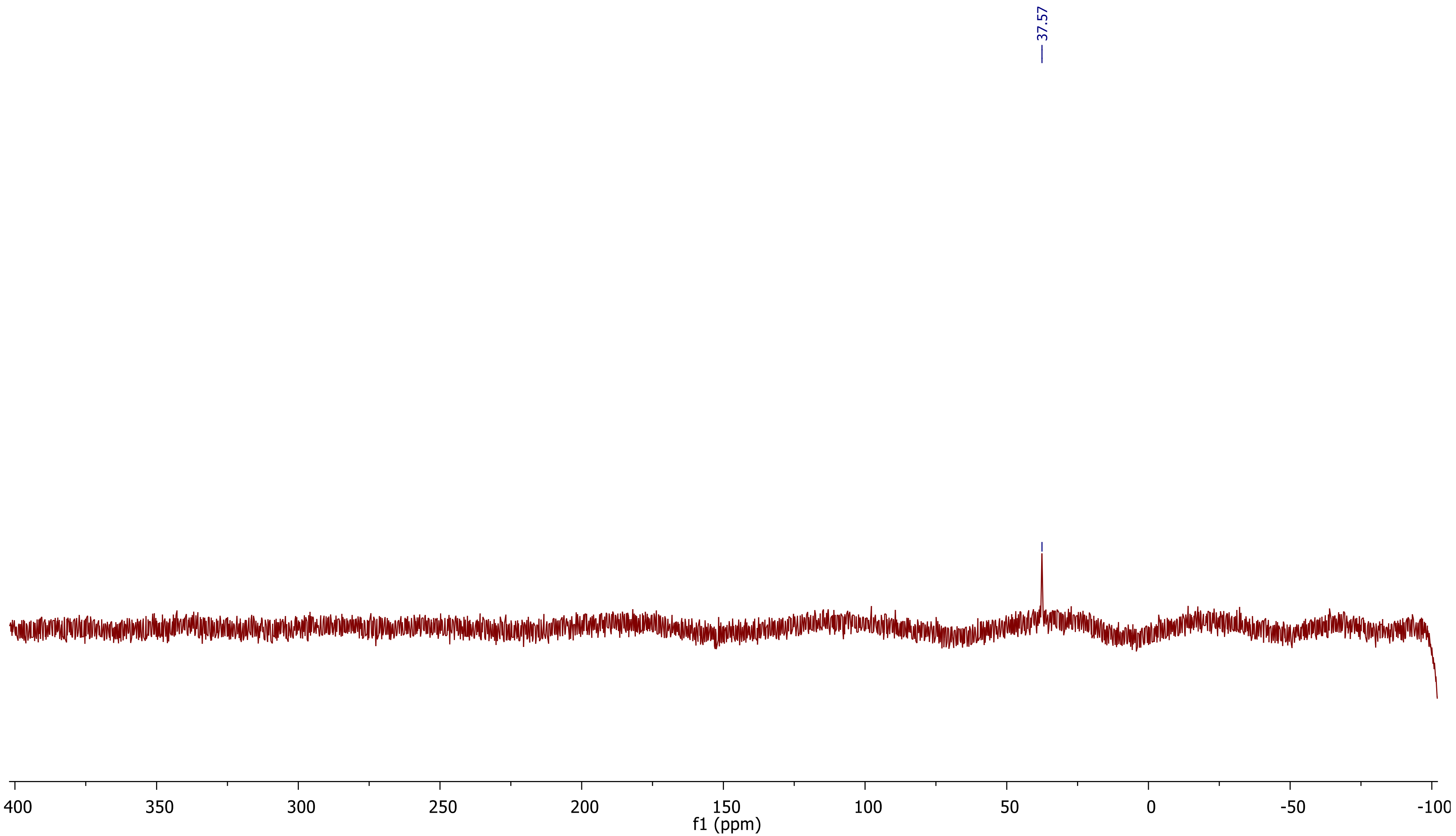

Figure S59. ^{77}Se NMR spectrum (95 MHz, THF-d_8, 298K) of **11**.

S3.10 [{(WCA-IDipp)Se}Ir(COD)] (**12**)

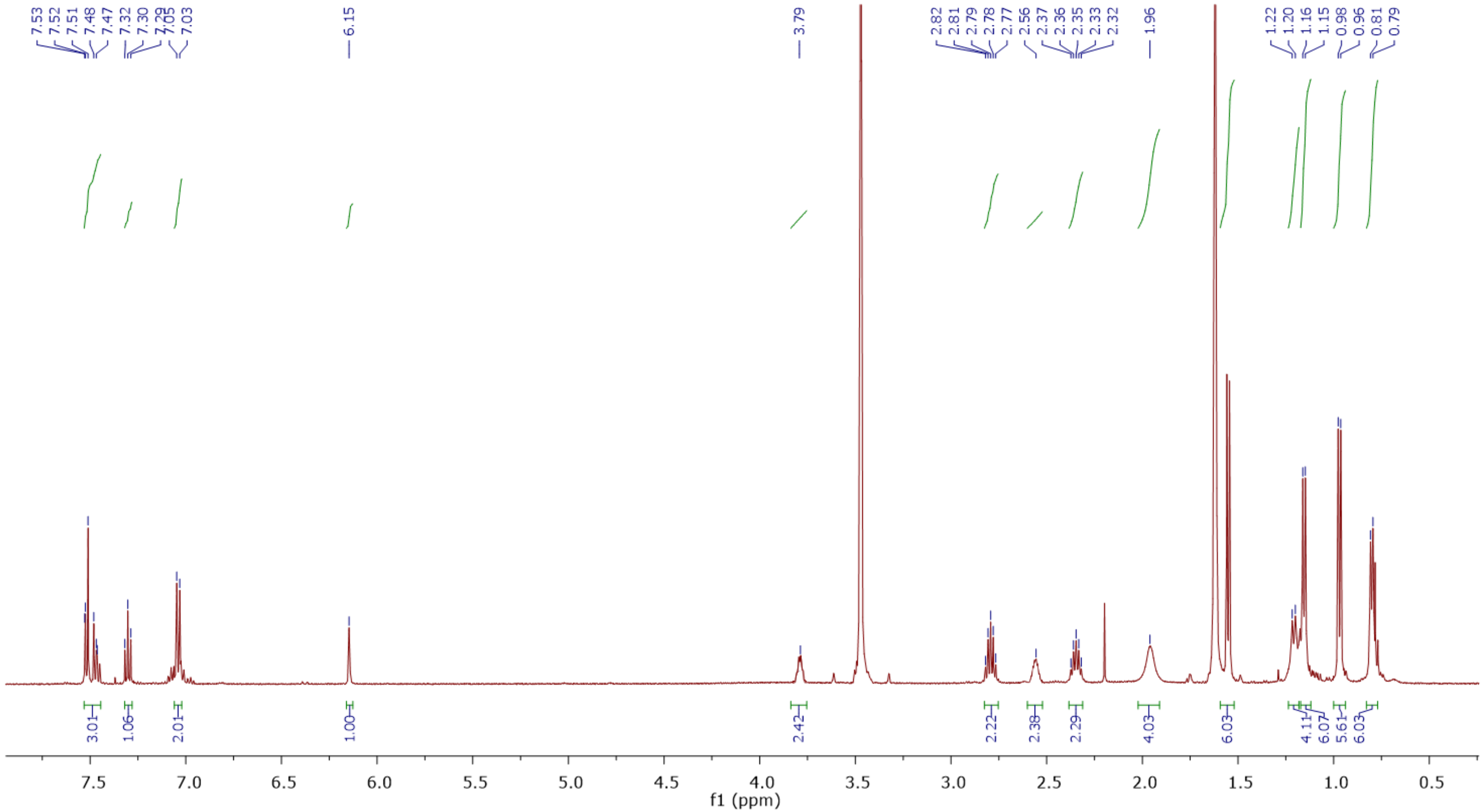

Figure S60. ^{1}H NMR spectrum (500 MHz, THF-d_8, 298K) of **12**.

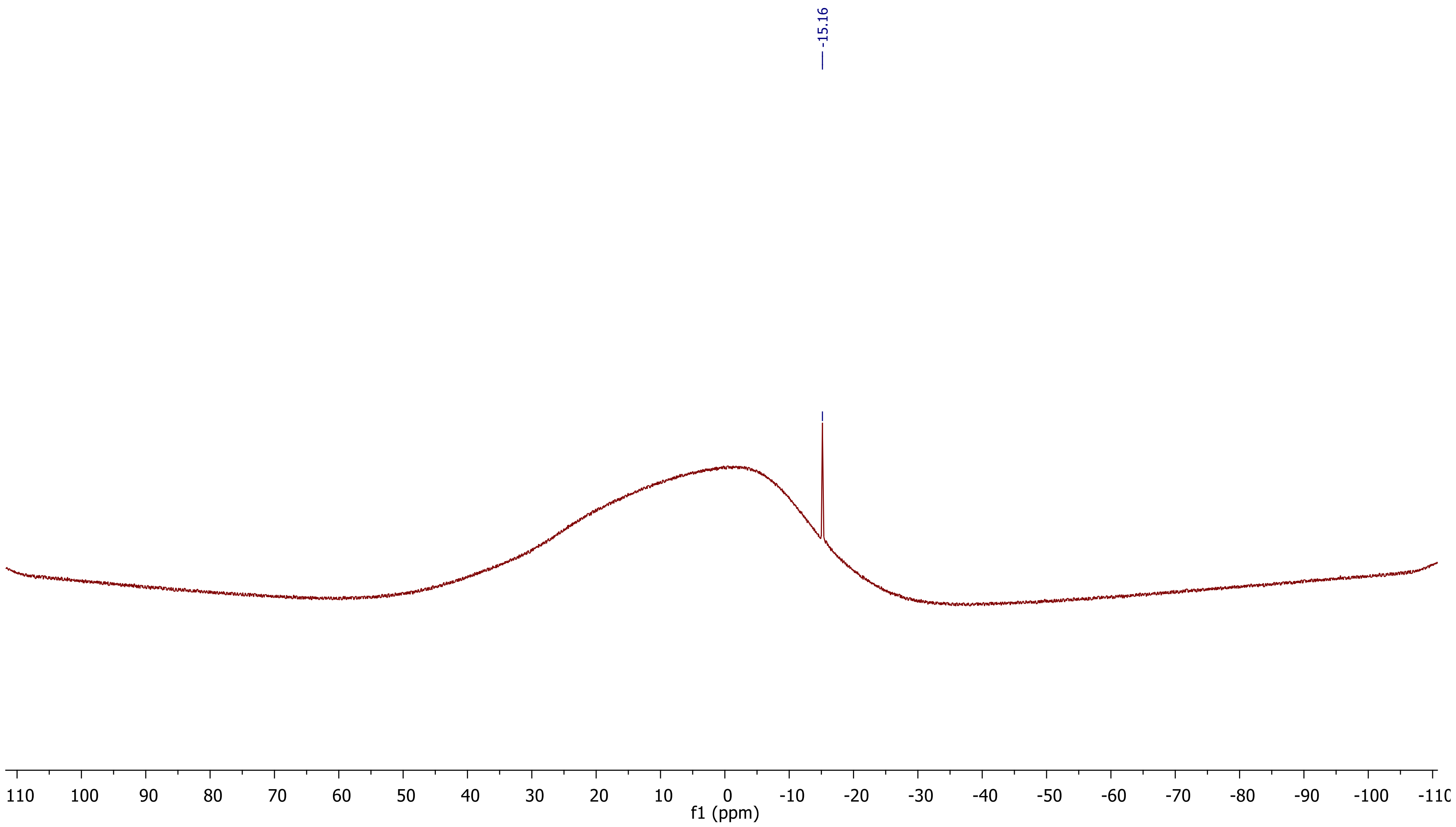

Figure S61. ^{11}B NMR spectrum (161 MHz, THF-d_8, 298K) of **12**.

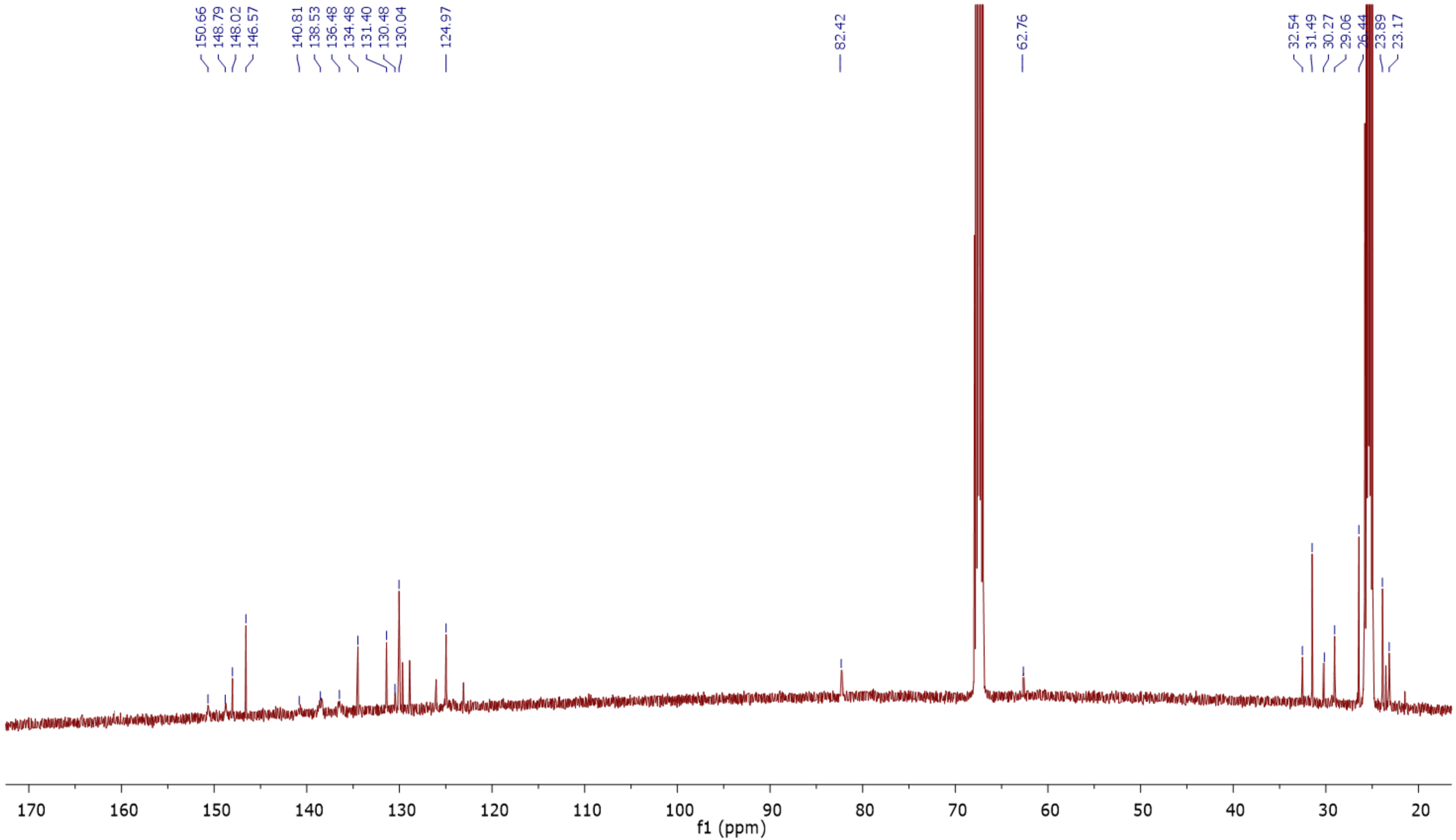

Figure S62. ^{13}C NMR spectrum (126 MHz, THF-d_8, 298K) of **12**.

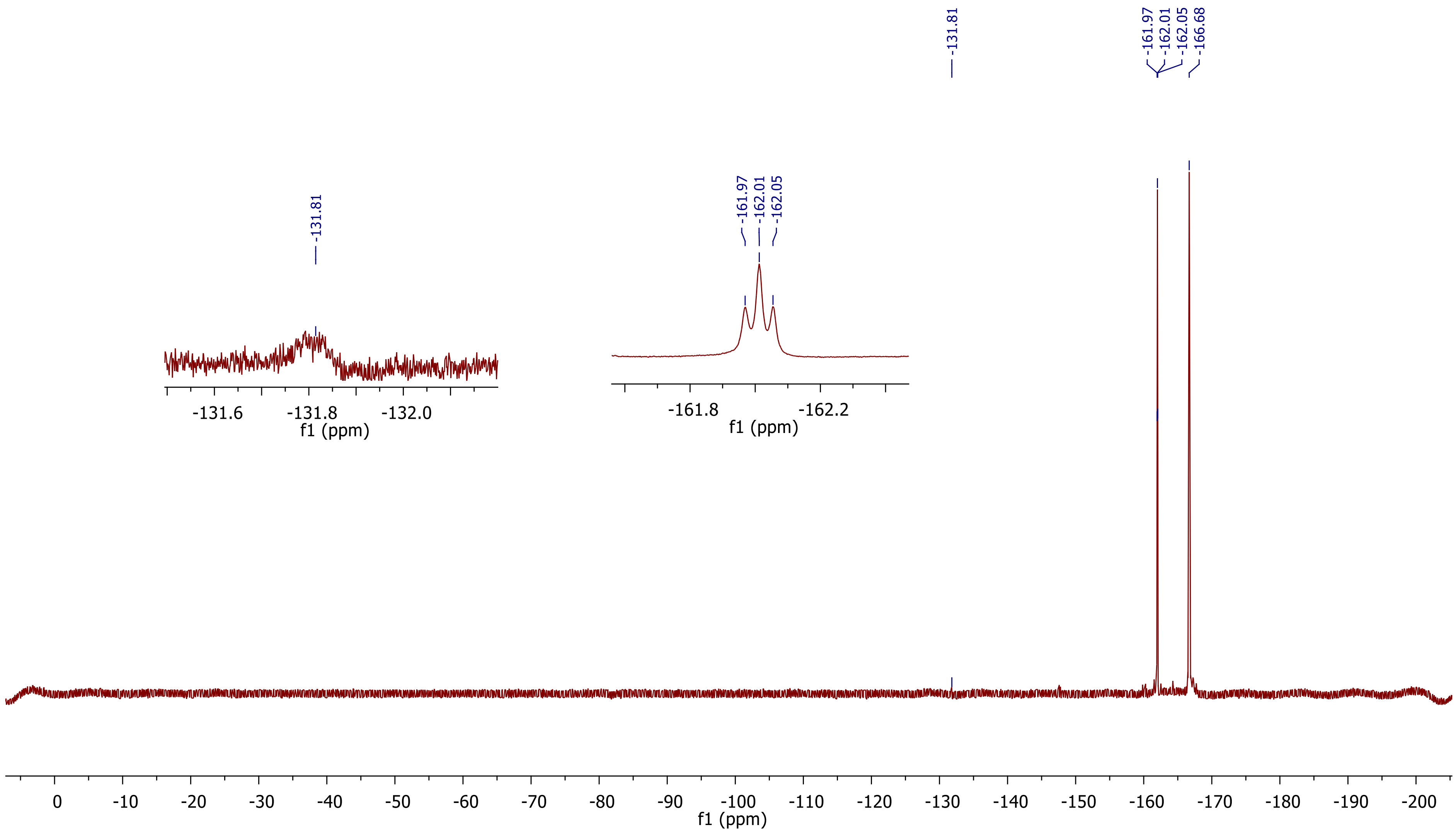

Figure S63. ^{19}F NMR spectrum (471 MHz, THF-d_8, 298K) of **12**.

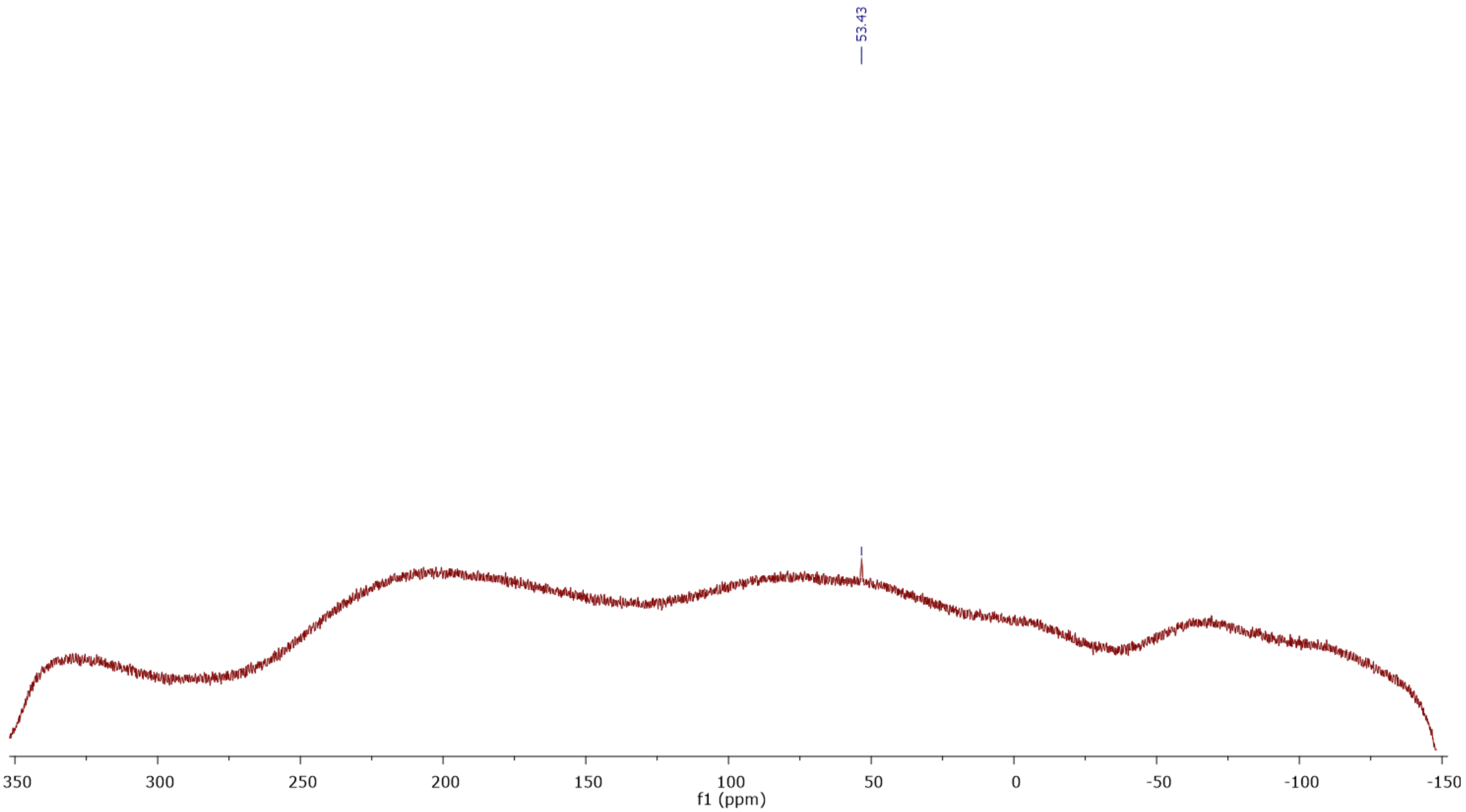

Figure S64. ^{77}Se NMR spectrum (95 MHz, THF-d_8, 298K) of **12**.

S3.11 [{(WCA-IDipp)S}$Rh_2(COD)_2Cl$] (**13**)

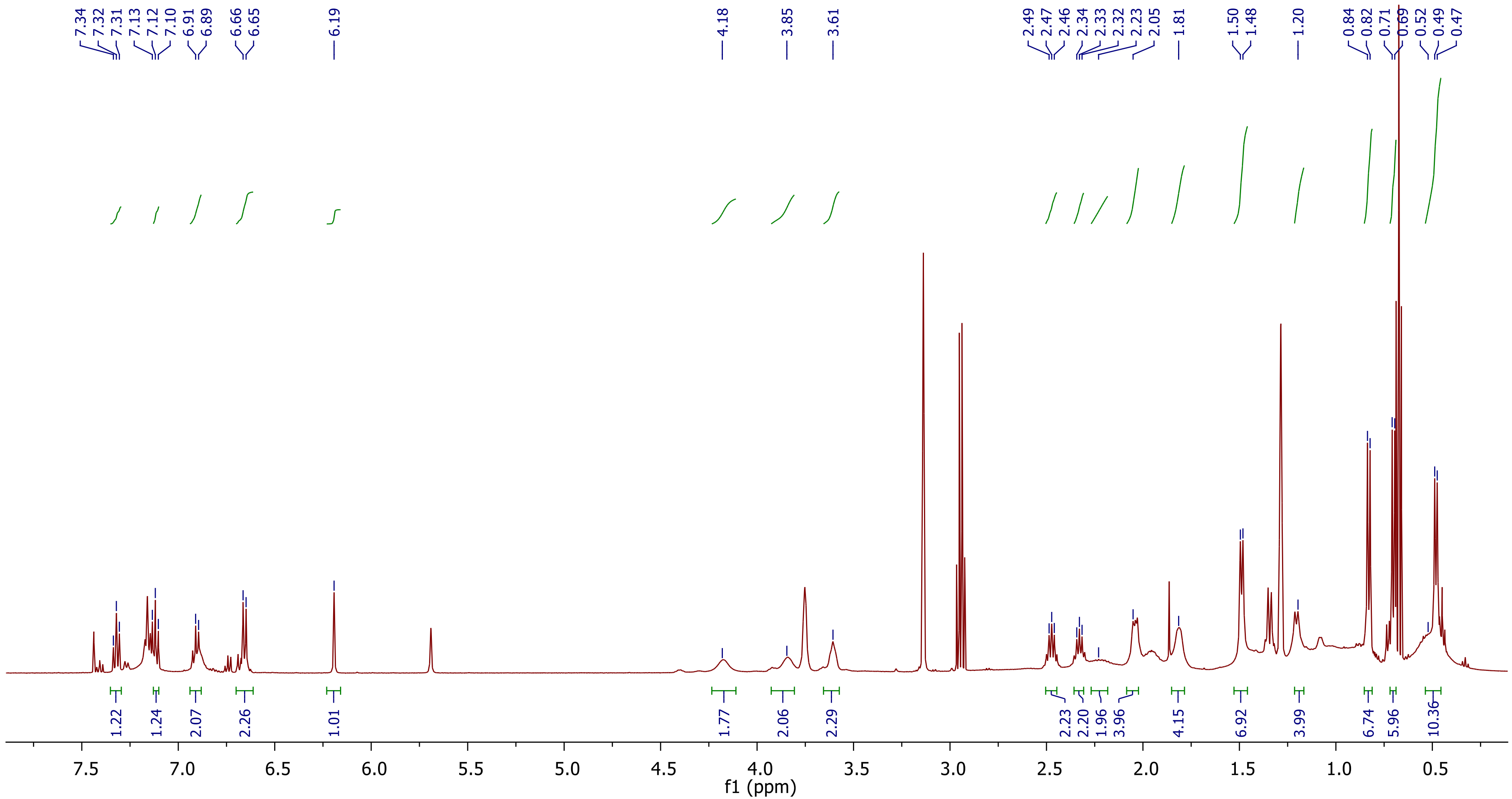

Figure S65. ^{1}H NMR spectrum (500 MHz, THF-d_8, 298K) of **13**.

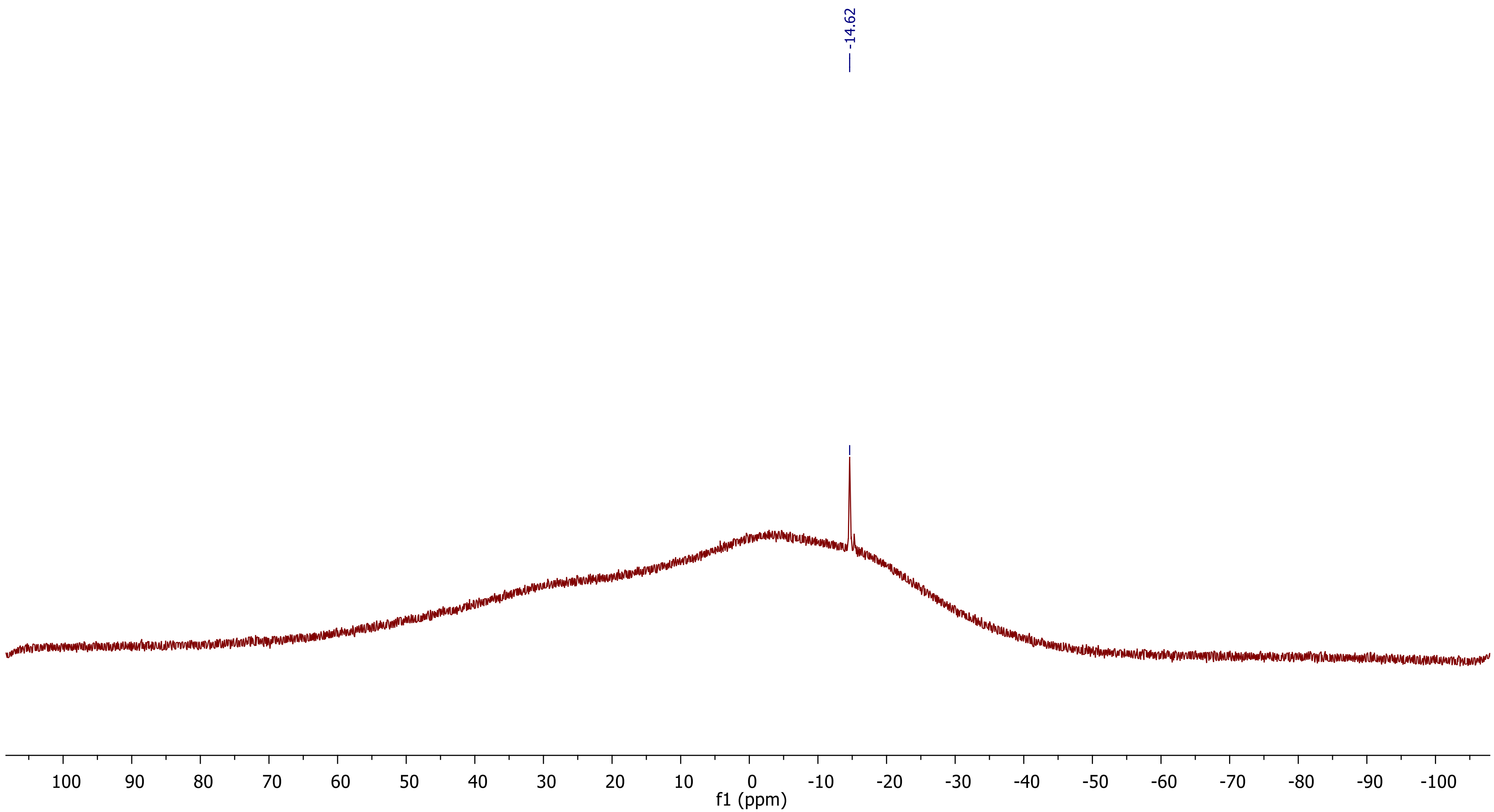

Figure S66. ^{11}B NMR spectrum (161 MHz, THF-d_8, 298K) of **13**.

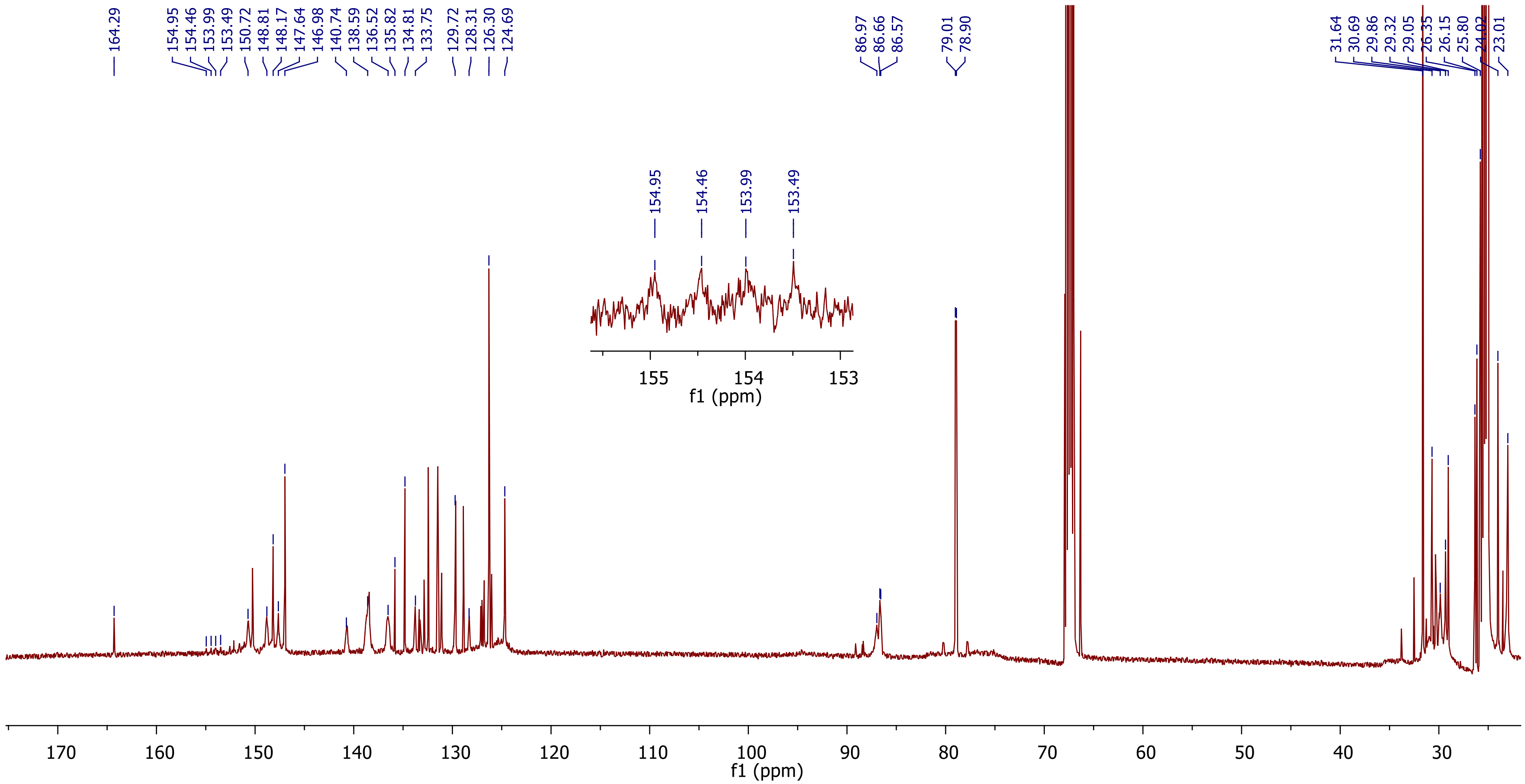

Figure S67. ^{13}C NMR spectrum (126 MHz, THF-d_8, 298K) of **13**.

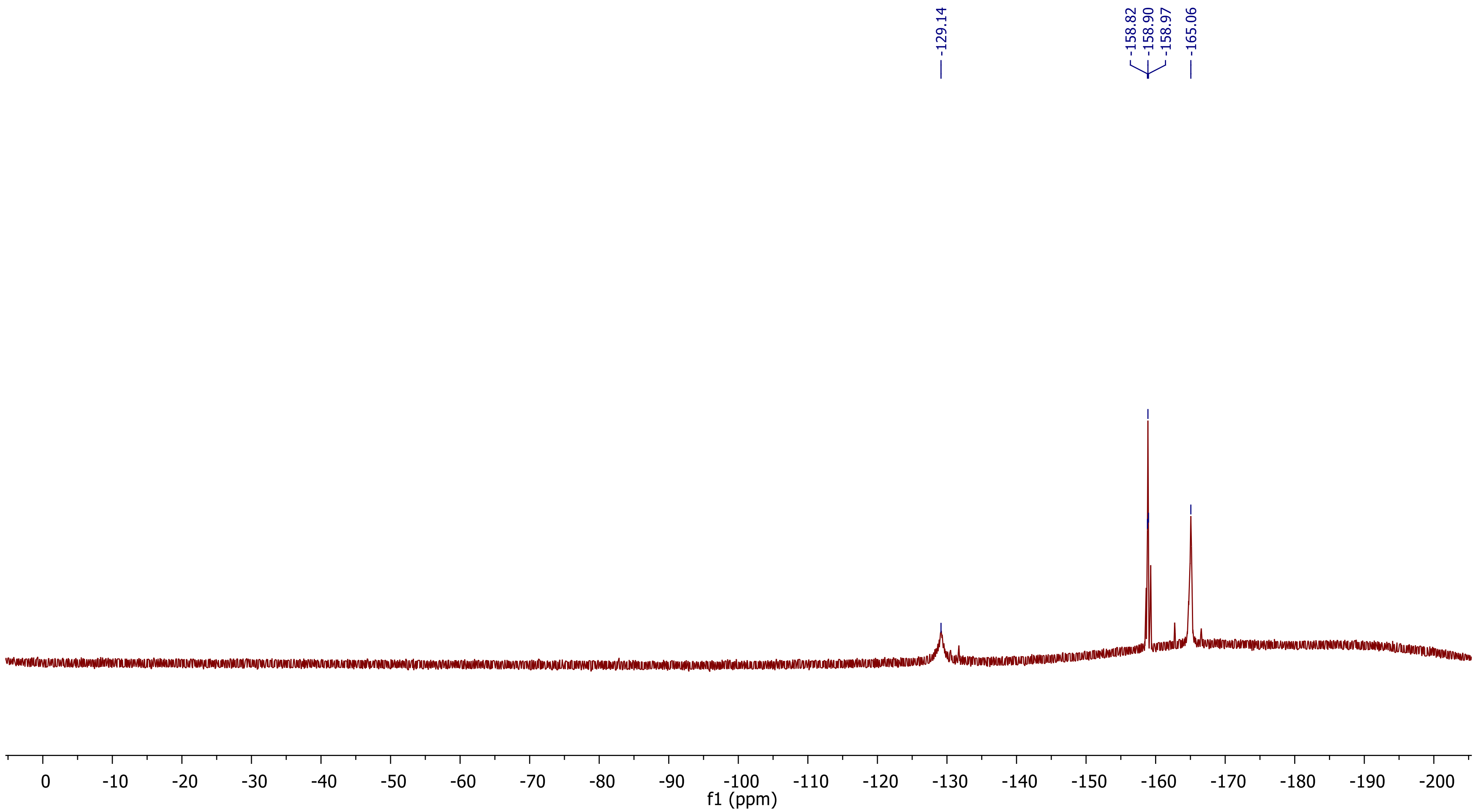

Figure S68. ^{19}F NMR spectrum (283 MHz, THF-d_8, 298K) of **13**.

S3.13 [{(WCA-IDipp)S}$Ir_2(COD)_2Cl$] (**14**)

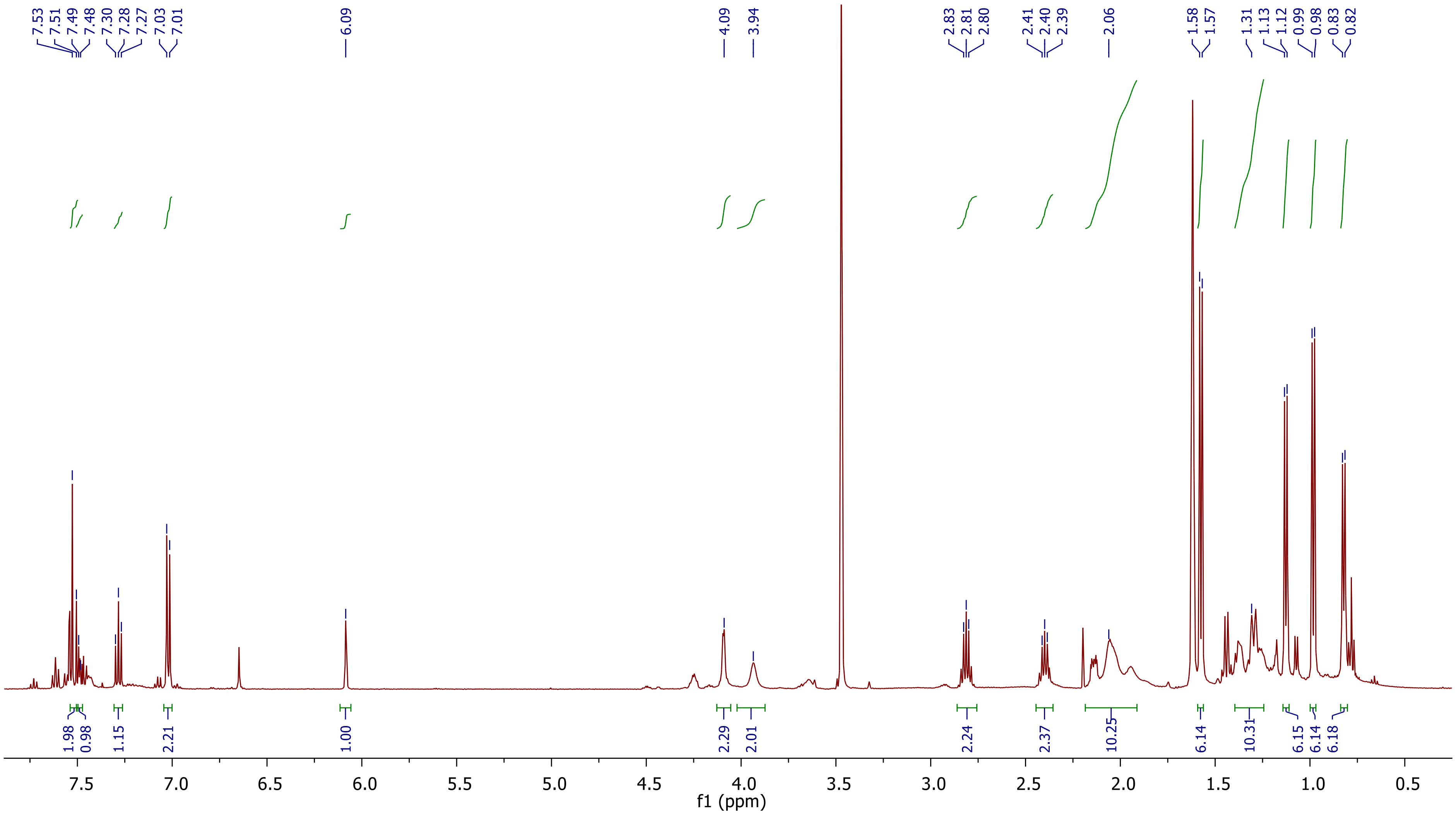

Figure S69. ^{1}H NMR spectrum (500 MHz, THF-d_8, 298K) of **14**.

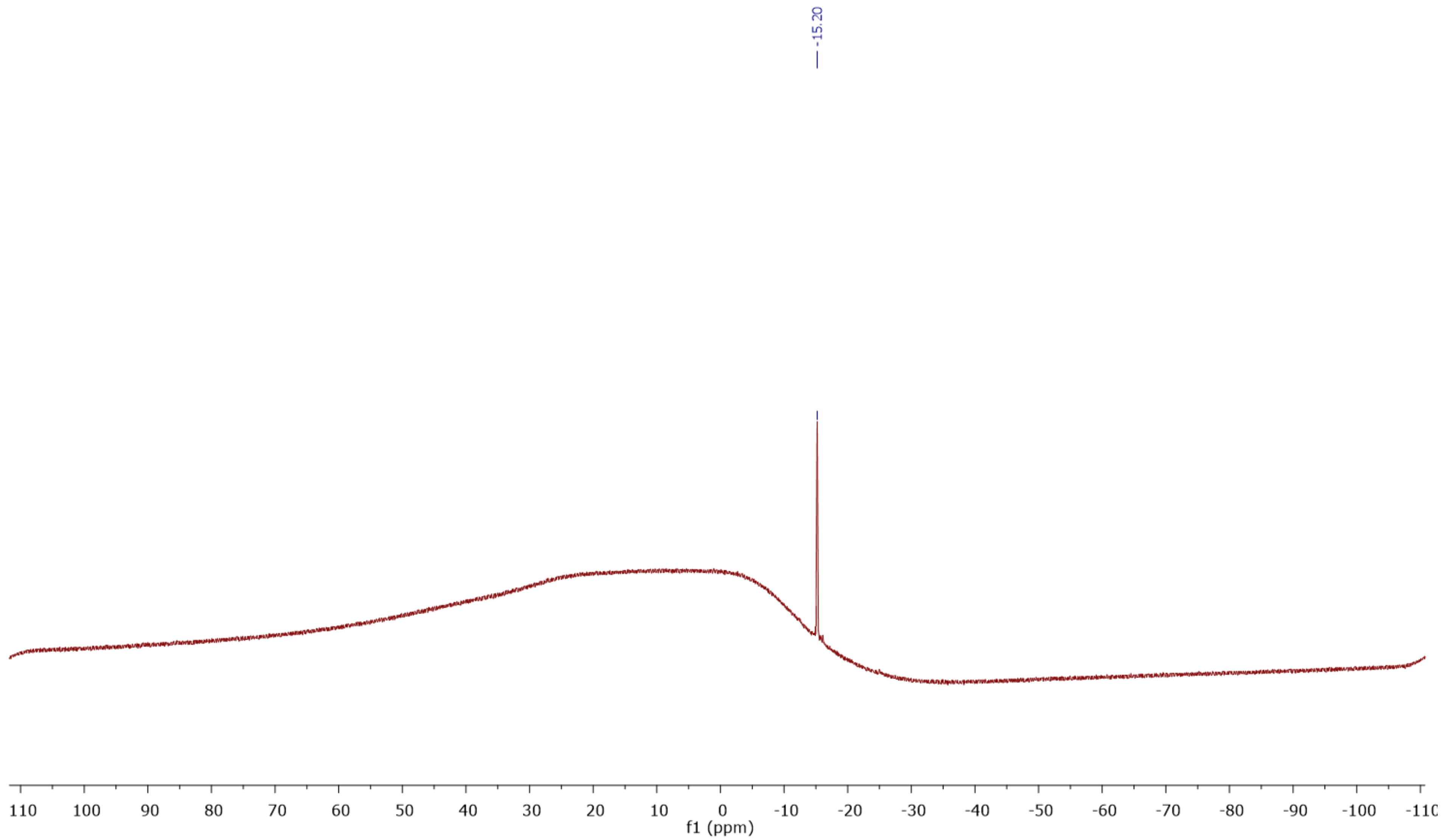

Figure S70. ^{11}B NMR spectrum (161 MHz, THF-d_8, 298K) of **14**.

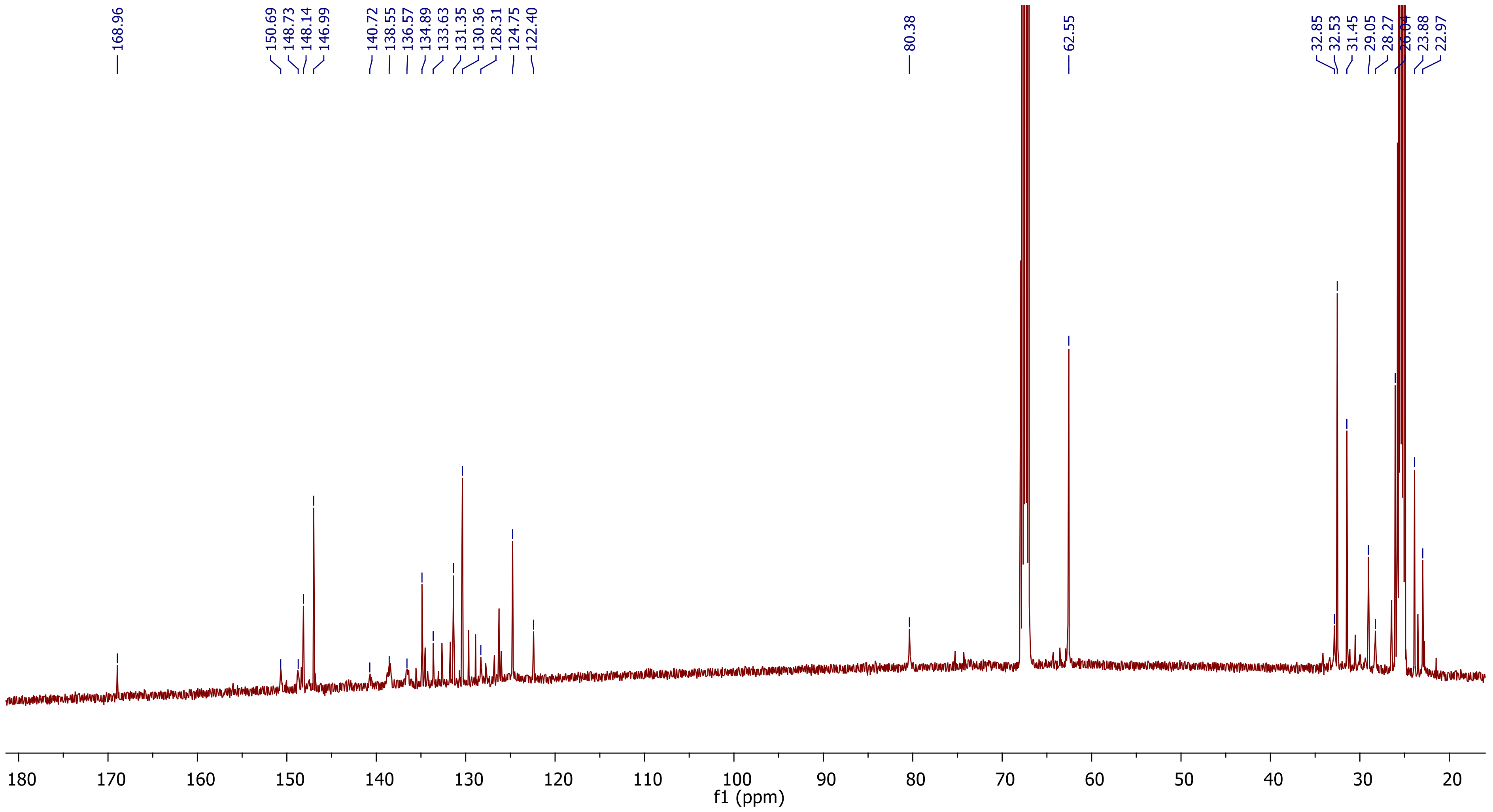

Figure S71. ^{13}C NMR spectrum (126 MHz, THF-d_8, 298K) of **14**.

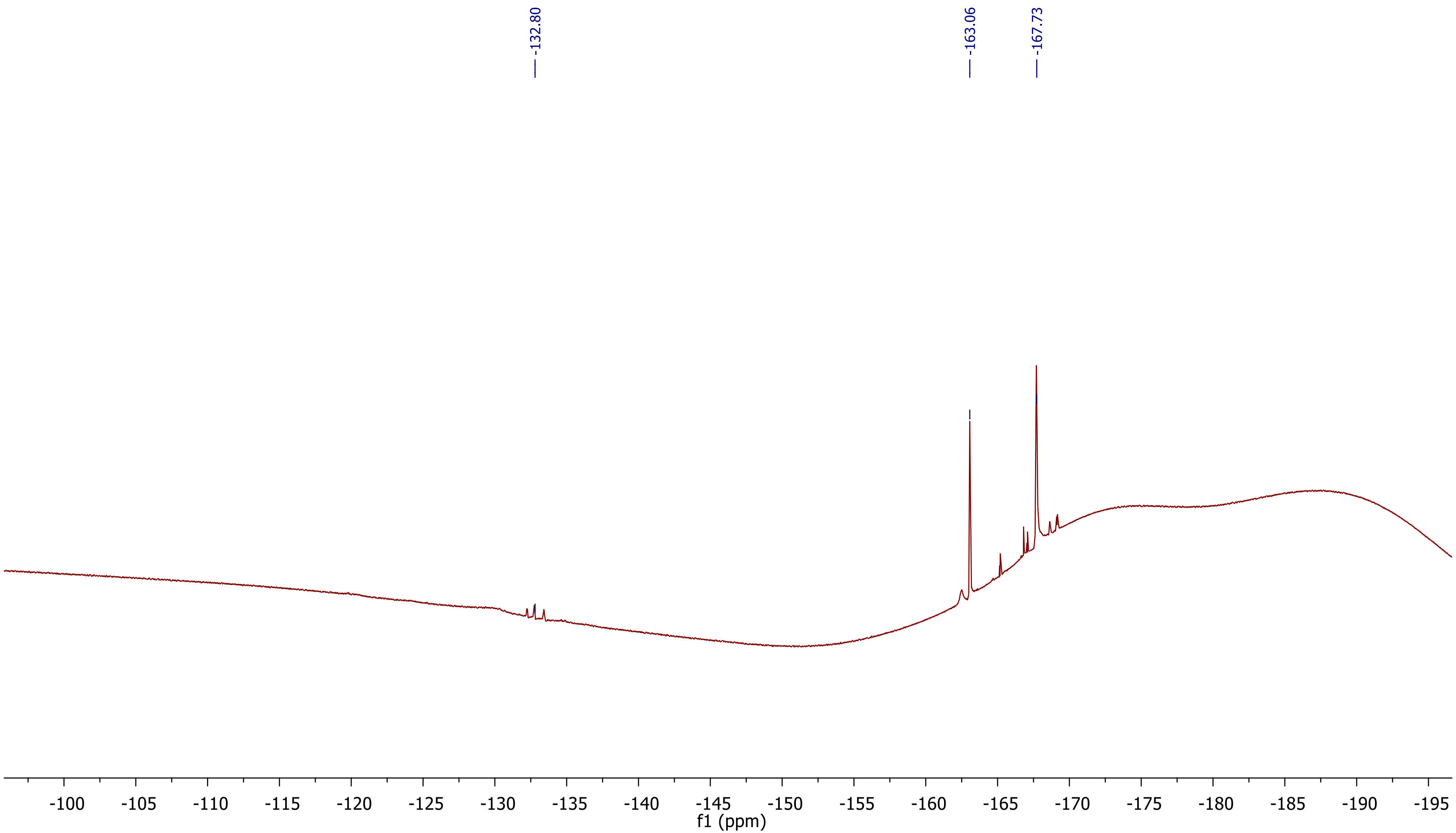

Figure S72. ^{19}F NMR spectrum (471 MHz, THF-d_8, 298K) of **14**.

S3.14 [{(WCA-IDipp)Se}Rh$_2$(COD)$_2$Cl] (**15**)

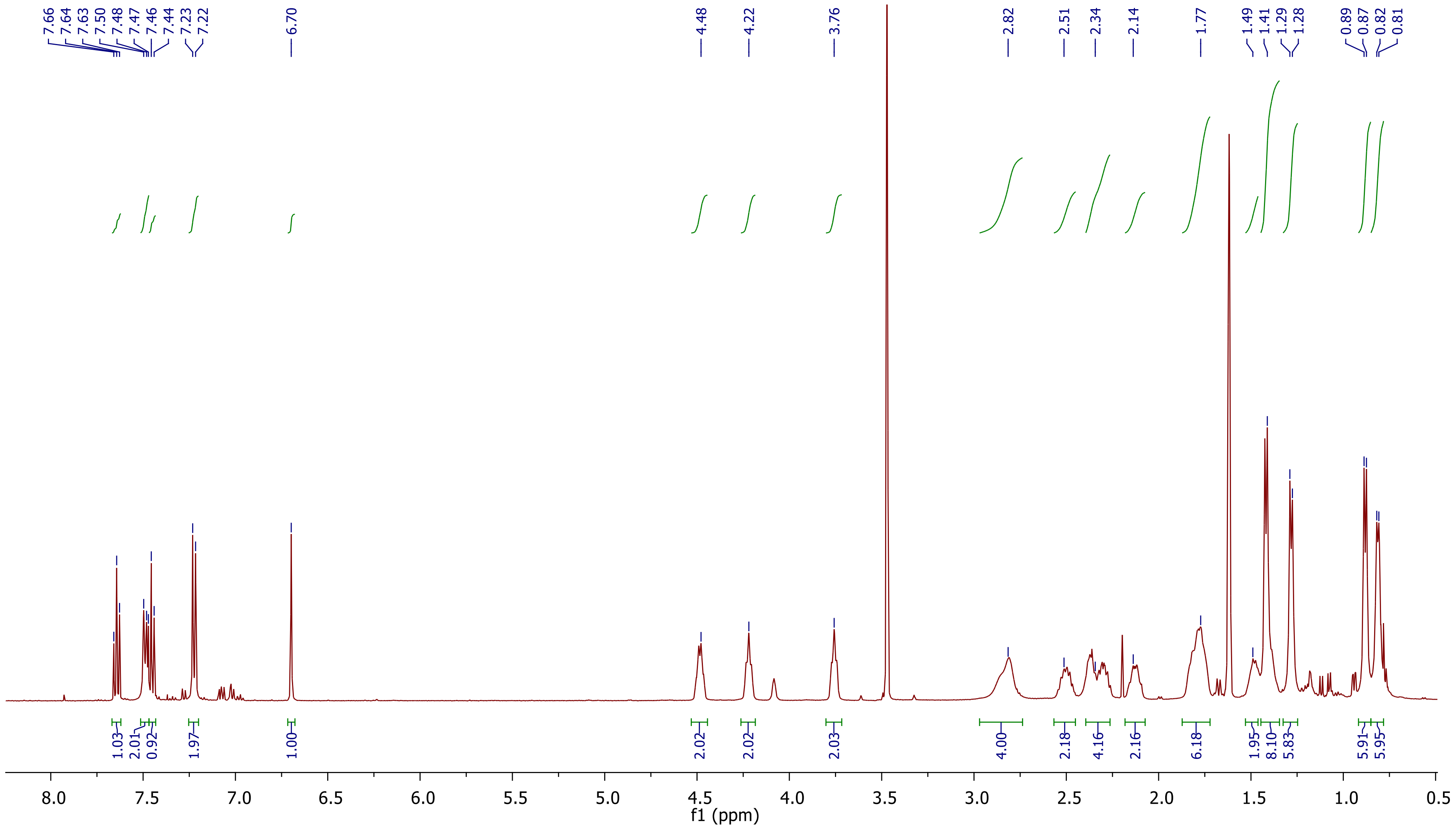

Figure S73. ^{1}H NMR spectrum (500 MHz, THF-d_8, 298K) of **15**.

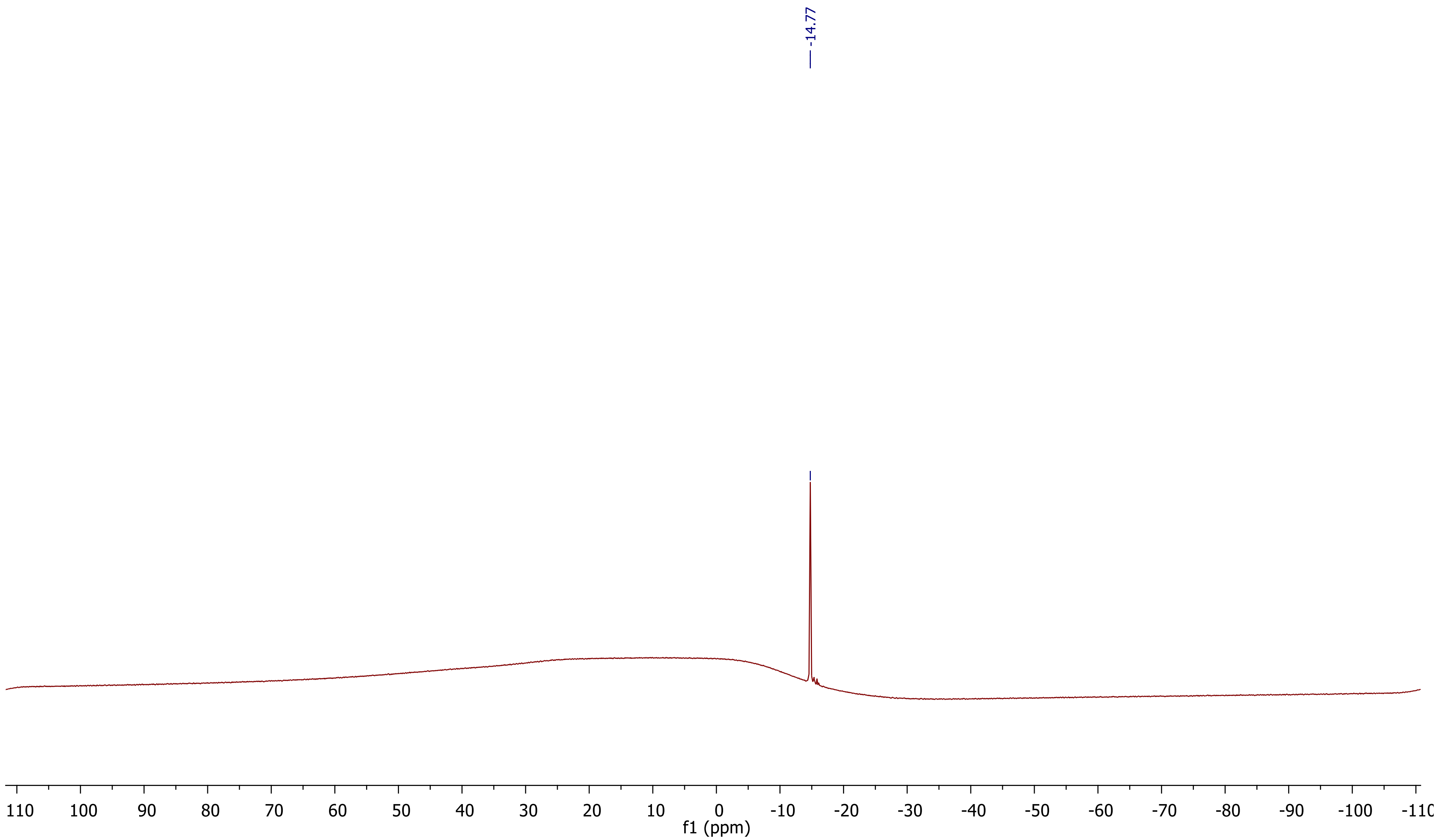

Figure S74. ^{11}B NMR spectrum (161 MHz, THF-d_8, 298K) of **15**.

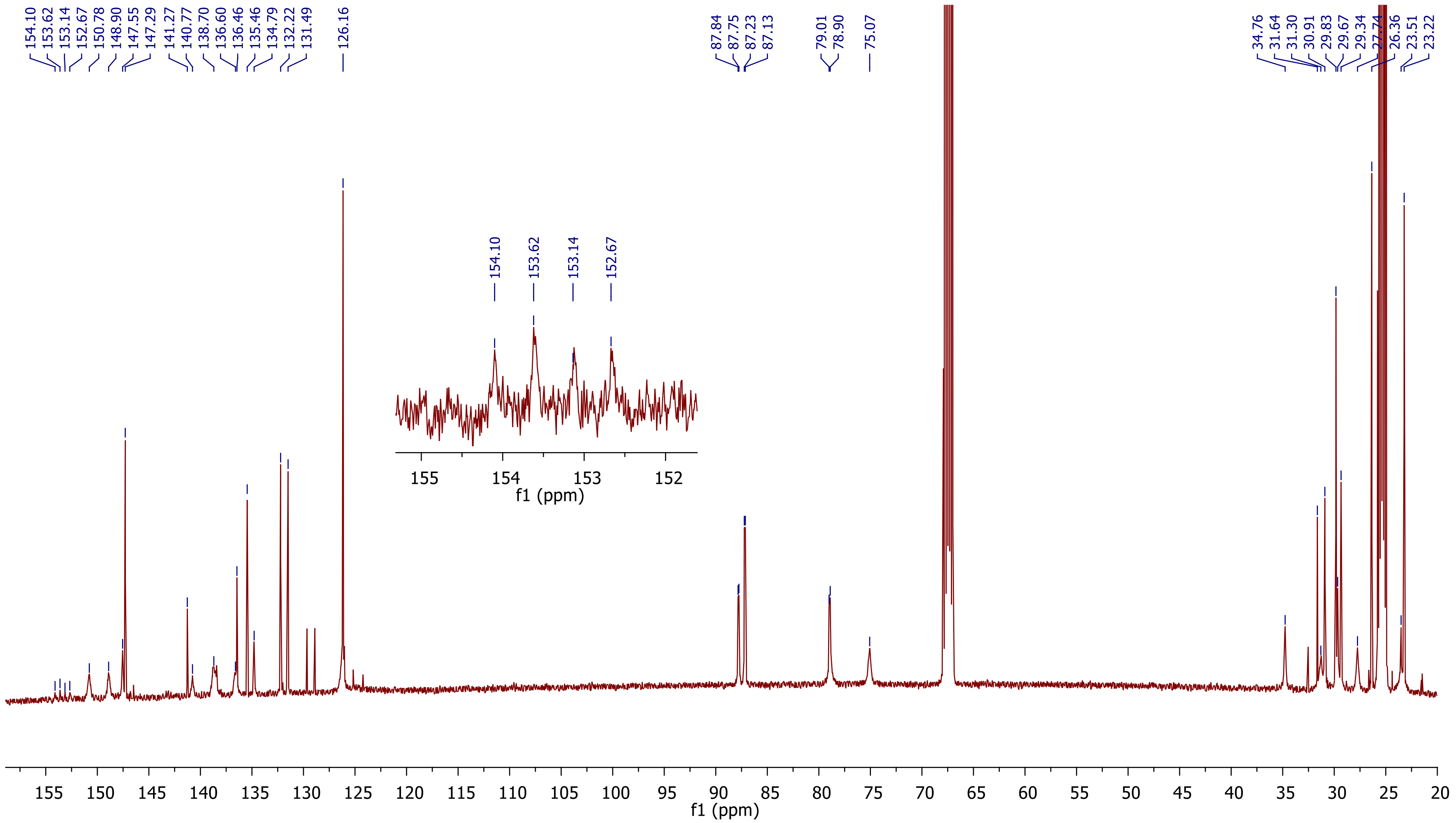

Figure S75. ^{13}C NMR spectrum (126 MHz, THF-d_8, 298K) of **15**.

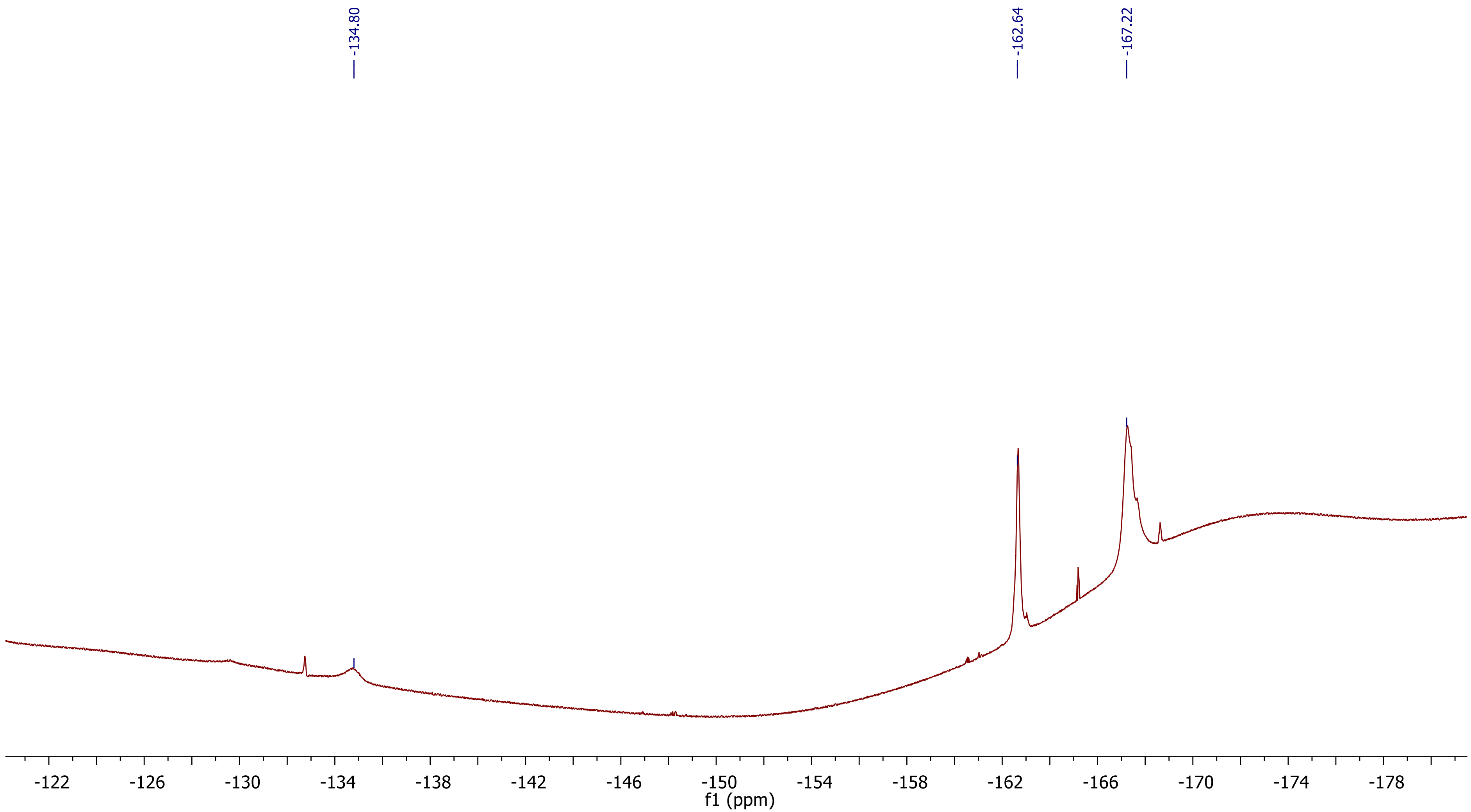

Figure S76. ^{19}F NMR spectrum (471 MHz, THF-d_8, 298K) of **15**.

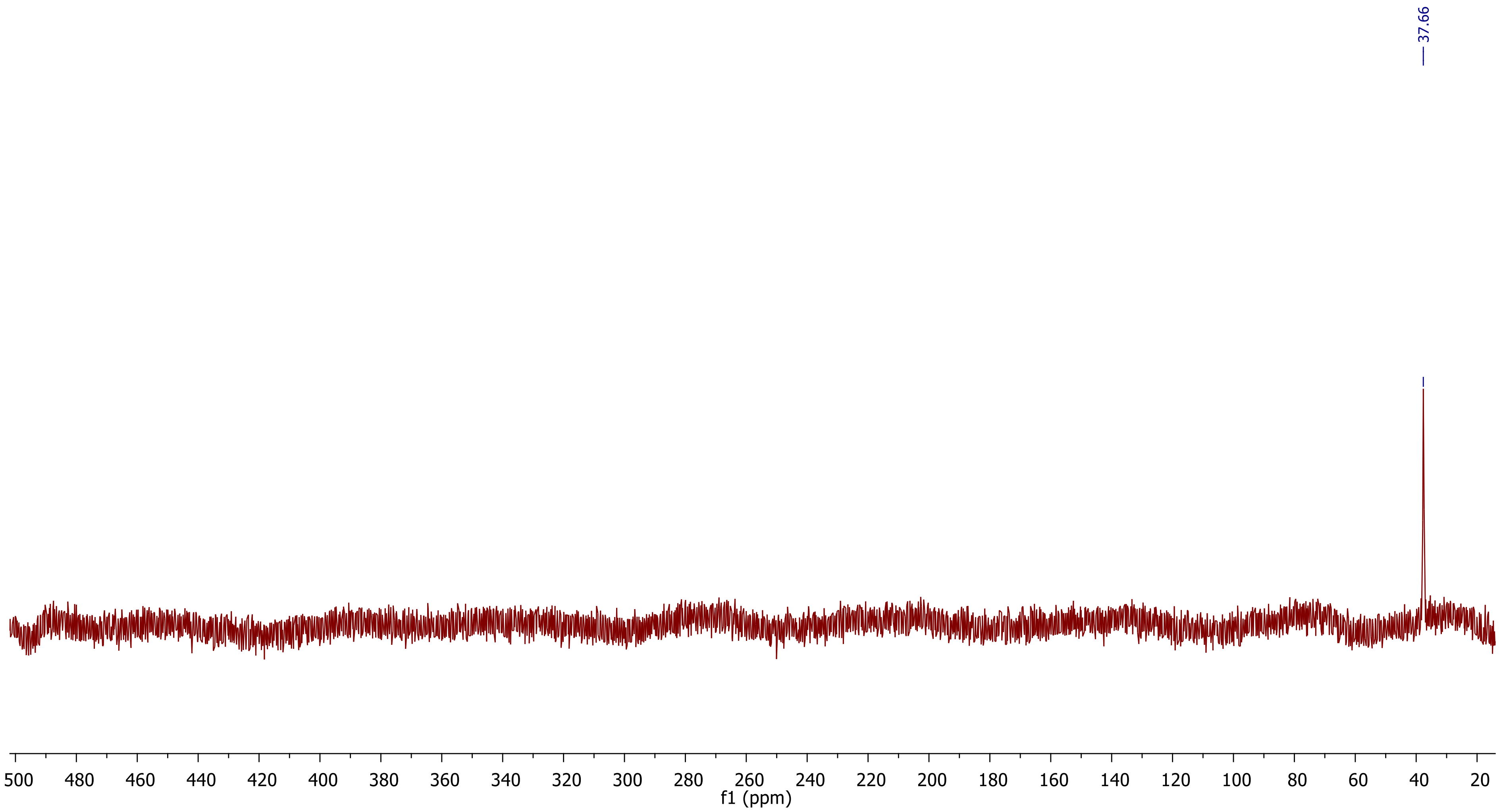

Figure S77. ^{77}Se NMR spectrum (95 MHz, THF-d_8, 298K) of **15**.

S3.15 [{(WCA-IDipp)Se}$Ir_2(COD)_2Cl$] (**16**)

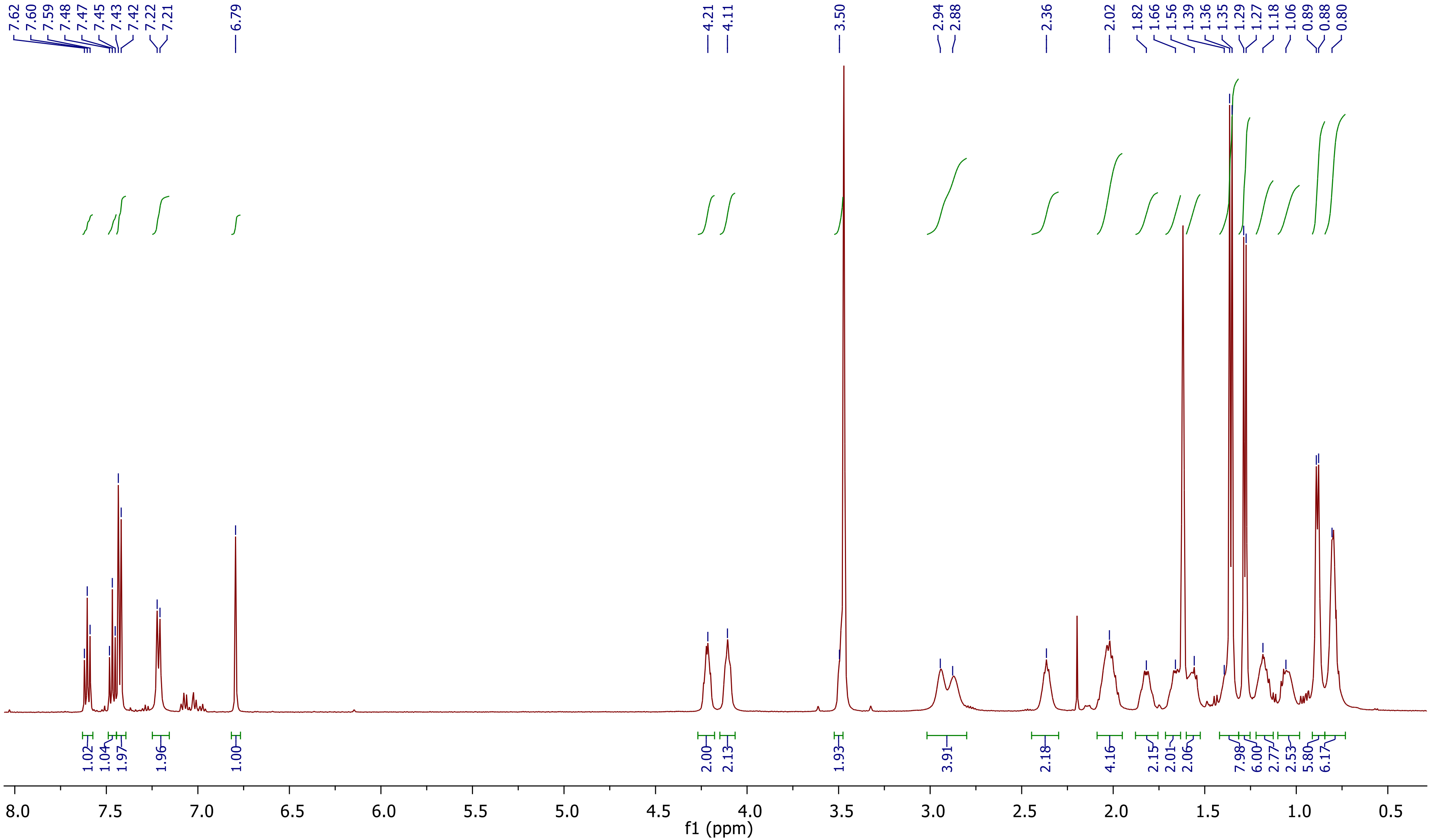

Figure S78. ^{1}H NMR spectrum (500 MHz, THF-d_8, 298K) of **16**.

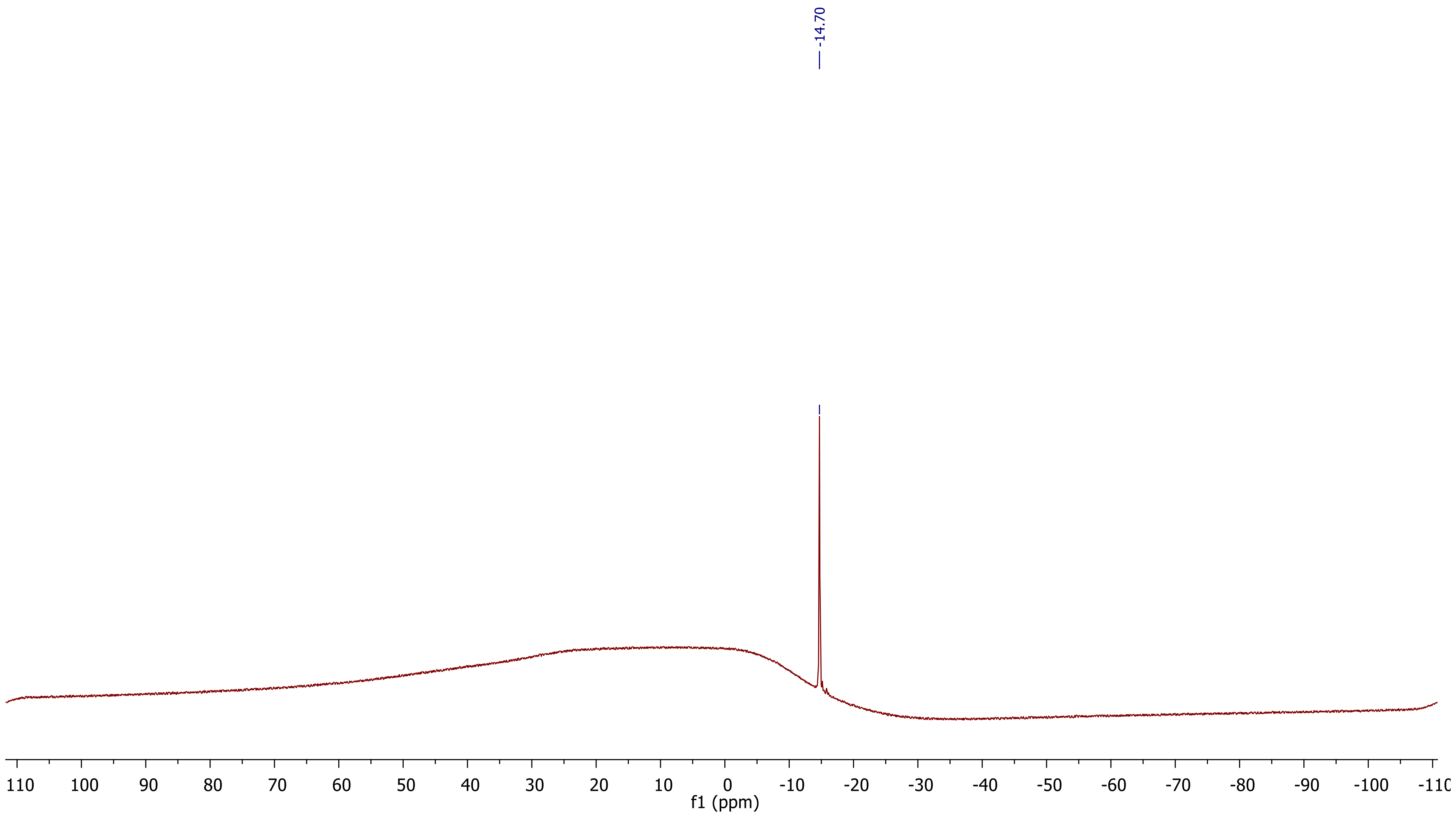

Figure S79. ^{11}B NMR spectrum (161 MHz, THF-d_8, 298K) of **16**.

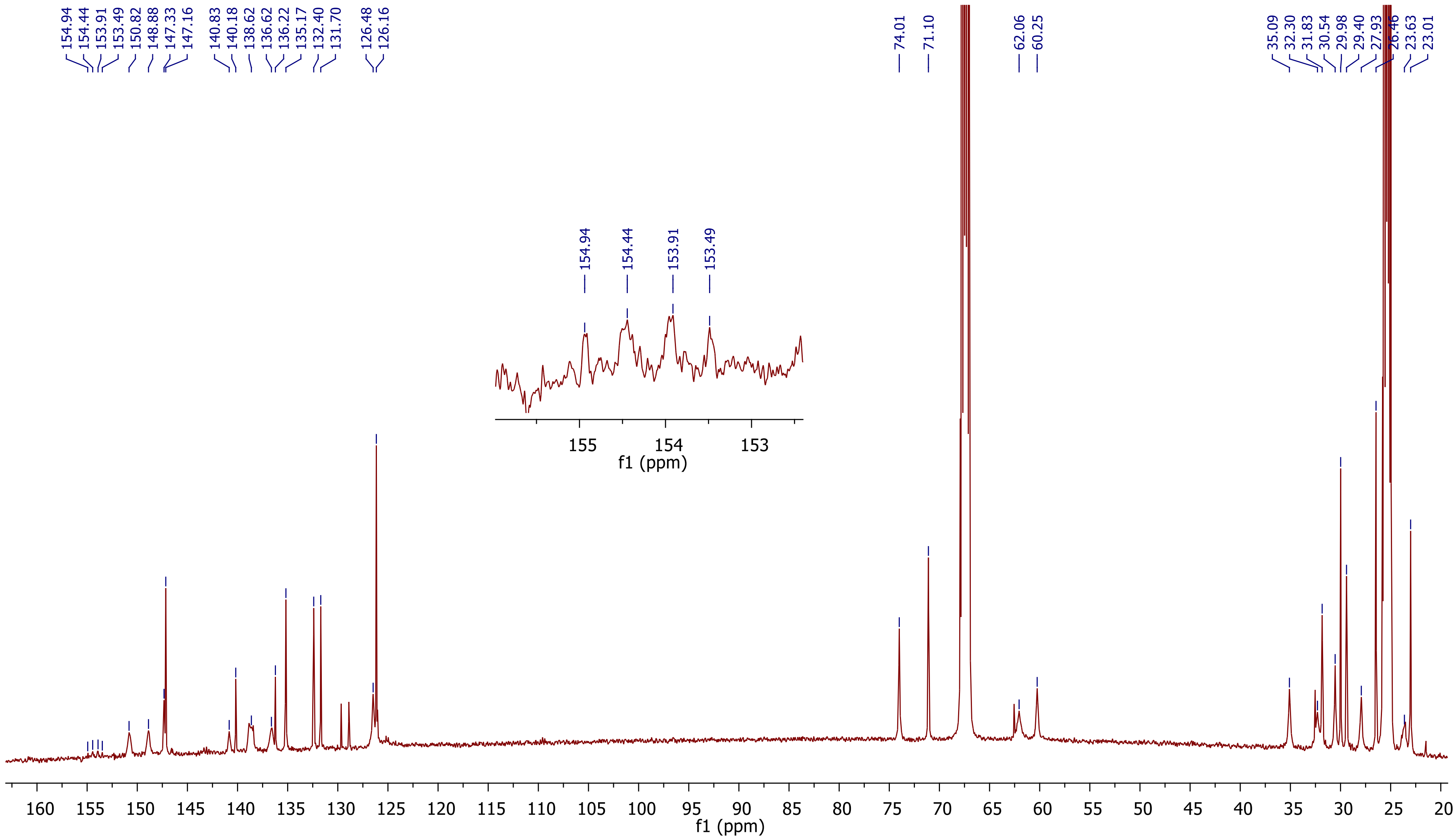

Figure S80. ^{13}C NMR spectrum (126 MHz, THF-d_8, 298K) of **16**.

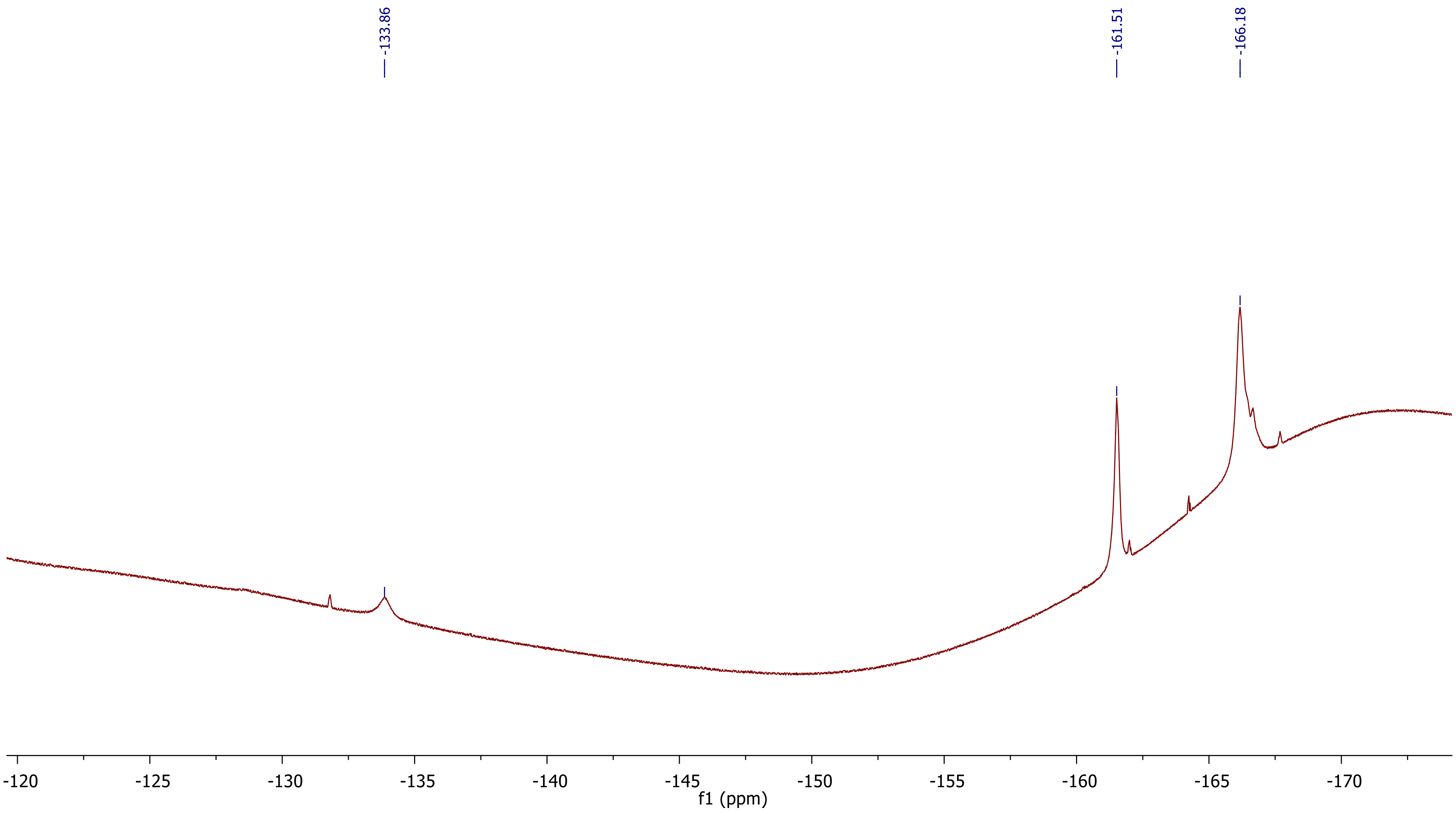

Figure S81. ^{19}F NMR spectrum (471 MHz, THF-d_8, 298K) of **16**.

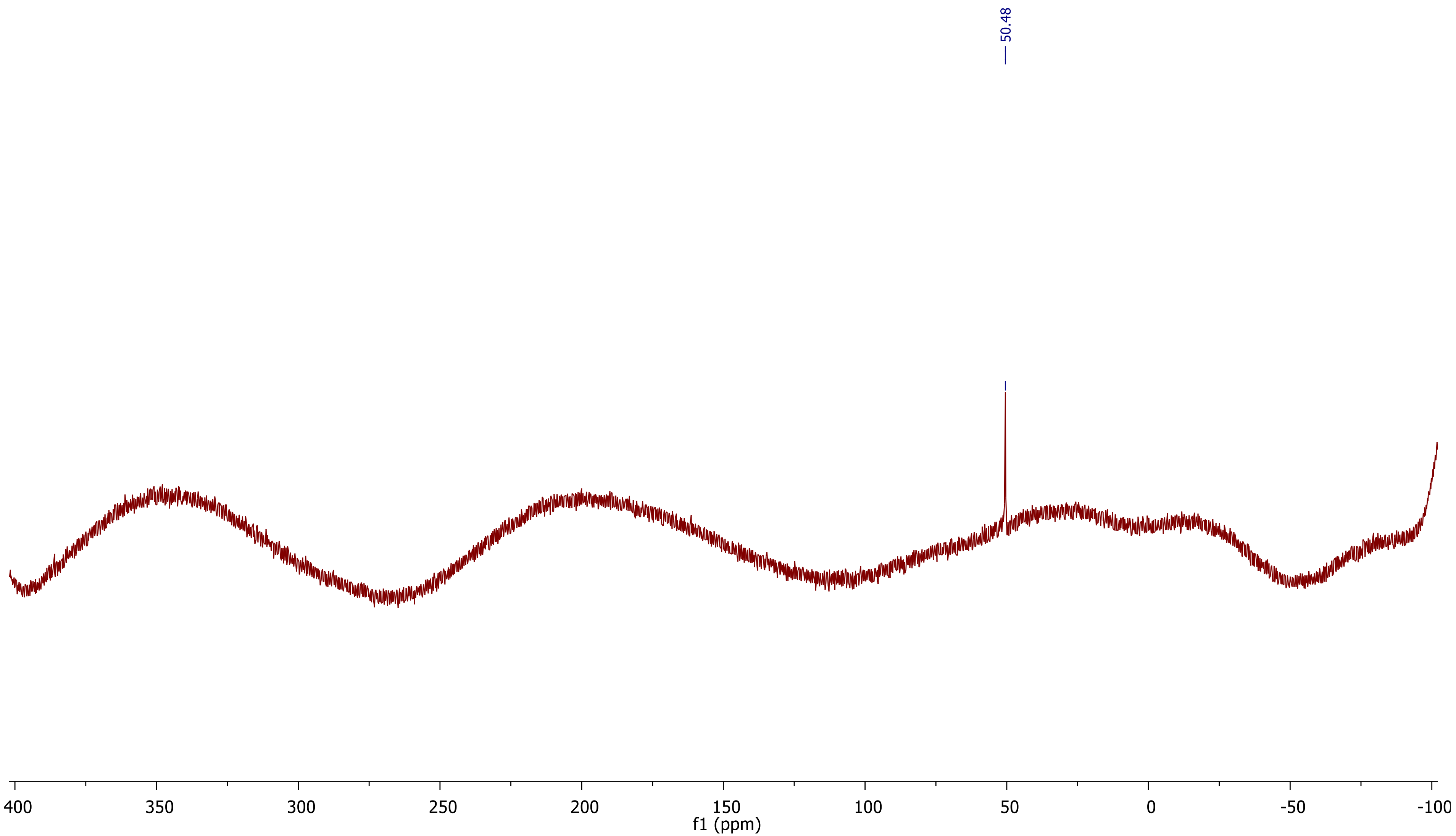

Figure S82. ^{77}Se NMR spectrum (95 MHz, THF-d_8, 298K) of **16**.

S4 Computational Details

All computations were performed using the density functional method B97-D (S. Grimme) as implemented in the Gaussian16 program.[3] For all main group elements (C, H, N, F, S, B and Cl) the all-electron triple-ζ basis set 6-311G(d,p)[4] was used, along with a quasi relativistic effective core potential – *Stuttgart RLC ECP* – for the 6d transition metal iridium (ECP60MWB) and for the heavier chalcogen element selenium (ECP28MWB).[5,6] Natural Bond Orbital (NBO) analysis (NBO charges, WBI) was carried out using NBO version 3,[7] which is part of the Gaussian16 program package. Harmonic vibrational frequencies are calculated to characterize respective minima structures (with no imaginary frequency).

Table S16. Energies for all optimized structures

	Compound		E_{0K}[a] / [Ha]	E_{298K}[b] / [Ha]	H_{298K}[b] / [Ha]	G_{298K}[b] / [Ha]
1	[(WCA-IDipp)**S**]⁻ [c]		–3764.227034	–3764.165669	–3764.164725	–3764.321295
2	[(WCA-IDipp)**Se**]⁻		–3375.389261	–3375.327548	–3375.326604	–3375.484564
3	[{(WCA-IDipp)**S**}IrCl(η^5-C_5Me_5)] (**6**)		–4718.805307	–4718.727127	–4718.726182	–4718.919276
4	[{(WCA-IDipp)**Se**}IrCl(η^5-C_5Me_5)] (**8**)		–4329.967038	–4329.888404	–4329.887460	–4330.081757
5	[{(WCA-IDipp)**S**}Ir(COD)] (**10**)	*anti*	–4180.564810	–4180.493769	–4180.492824	–4180.670107
		syn	–4180.562373	–4180.491345	–4180.490401	–4180.670013
		**Δ*E*(*anti-syn*)** / [kcal mol^{-1}]	–1.5	–1.5	–1.5	–0.1
6	[{(WCA-IDipp)**Se**}Ir(COD)] (**12**)	*anti*	–3791.726126	–3791.654824	–3791.653880	–3791.831632
		syn	–3791.722169	–3791.650827	–3791.649883	–3791.830138
		**Δ*E*(*anti-syn*)** / [kcal mol^{-1}]	–2.5	–2.5	–2.5	–0.9

[a] DFT energy incl. ZPE.
[b] standard conditions T = 298.15 K and p = 1 atm.
[c] WCA = weakly coordinating anion: $[B(C_6F_5)_3]^-$.

Table S17. NBO analysis of anionic chalcogen [(WCA-IDipp)E]⁻ ligands **III** (E = S, Se)

	NBO no.	type	element	NBO charge	WBI	coefficient	localization [%]	AO contribution [%] s	p	d
[(WCA-IDipp)**S**]⁻	238	LP(S)	S	–0.31		1	100	0	99.9	0.1
	237	LP(S)	S			1	100	0	99.9	0.1
	136	LP(S)	S			1	100	80.4	19.6	0
	126	s(S–C_{NHC})	S		1.44	0.65	41.6	20.0	79.3	0.7
			C	0.25		0.76	58.4	39.3	60.6	0.1
[(WCA-IDipp)**Se**]⁻	233	LP(Se)	Se	–0.29		1	100	0	100	0
	232	LP(Se)	Se			1	100	0	100	0
	148	LP(Se)	Se			1	100	87.8	12.2	0
	143	s(Se–C_{NHC})	Se		1.30	0.60	36.5	12.4	87.6	0
			C	0.20		0.80	63.5	38.9	61.1	0

[(WCA-IDipp)**S**]$^{-}$ (**III**: E = S)

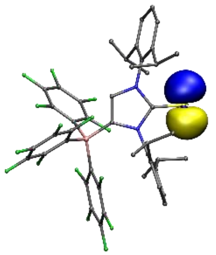

NBO #238

LP(S)

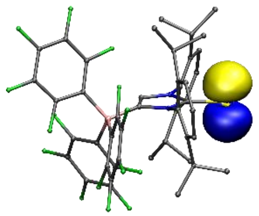

NBO #237

LP(S)

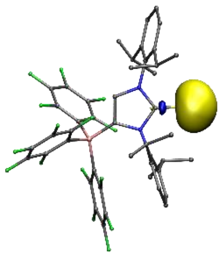

NBO #136

LP(S)

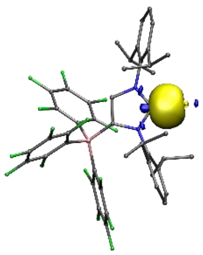

NBO #126

s(S–C_{NHC})

[(WCA-IDipp)**Se**]$^{-}$ (**III**: E = Se)

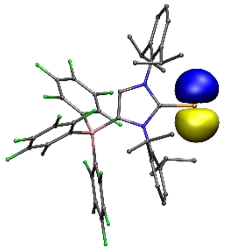

NBO #233

LP(Se)

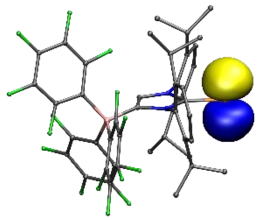

NBO #232

LP(Se)

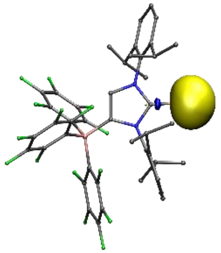

NBO #148

LP(Se)

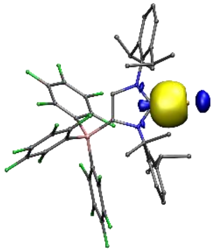

NBO #143

s(Se–C_{NHC})

Table S18. NBO analysis of pentamethylcyclopentadienyl (Cp*) iridium chalcogen complexes **6** and **8** (S and Se)

	NBO no.	type	element	NBO charge	WBI	coefficient	localization [%]	AO contribution [%] s	p	d
[{(WCA-IDipp)**S**}IrCl(η^5-C_5Me_5)] **(6)**	287	LP(S)	S	0.09		1	100	0.3	99.7	0.1
	200	s(Ir–S)	Ir	–0.06	0.88	0.55	30.2	26.6	34.6	38.8
			S			0.84	69.8	16.8	83.0	0.2
	167	LP(S)	S			1	100	64.9	35.1	0
	136	s(S–C_{NHC})	S		1.13	0.66	43.8	18.2	81.2	0.6
			C	0.29		0.75	56.2	36.1	63.8	0.1
[{(WCA-IDipp)**Se**}IrCl(η^5-C_5Me_5)] **(8)**	285	LP(Se)	Se	0.20		1	100	0.2	99.8	0
	230	s(Ir–Se)	Ir	–0.10	0.90	0.61	37.2	37.9	11.7	50.4
			Se			0.79	62.8	14.0	86.0	0
	194	LP(Se)	Se			1	100	74.3	25.7	0
	172	s(Se–C_{NHC})	Se		1.04	0.63	39.3	11.7	88.3	0
			C	0.25		0.78	60.7	35.2	64.8	0

[{(WCA-IDipp)**S**}IrCl(η^5-C_5Me_5)] (**6**)

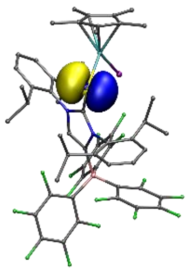

NBO #287

LP(S)

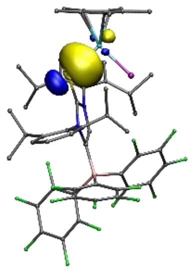

NBO #200

s(Ir–S)

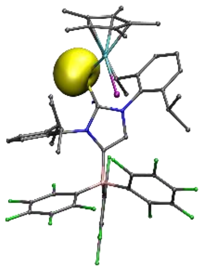

NBO #167

LP(S)

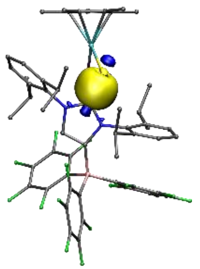

NBO #136

s(S–C_{NHC})

[{(WCA-IDipp)**Se**}IrCl(η^5-C_5Me_5)] (**8**)

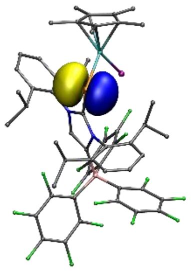

NBO #285

LP(Se)

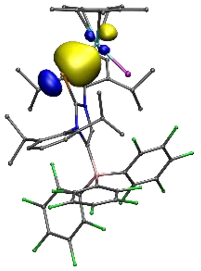

NBO #230

s(Ir–Se)

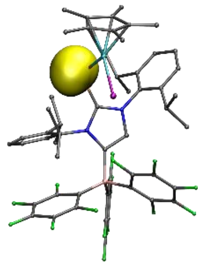

NBO #194

LP(Se)

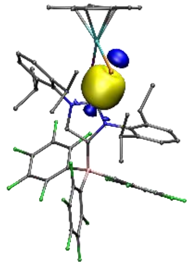

NBO #172

s(Se–C_{NHC})

Table S19. NBO analysis of cyclooctadiene (COD) iridium chalcogen complexes **10** and **12** with the iridium bound N-Dipp group coordinated opposite to the WCA moiety of the imidazole backbone (as it is found in the X-ray structure)

	NBO no.	type	element	NBO charge	WBI	coefficient	localization [%]	AO contribution [%] s	p	d
[{(WCA-IDipp)S}Ir(COD)] **(10)**	271	LP(S)	S	0.08		1	100	0.1	99.8	0.1
	220	s(Ir–S)	Ir	–0.08	0.75	0.49	23.7	25.6	48.6	25.9
			S			0.87	76.3	19.1	80.7	0.2
	154	LP(S)	S			1	100	62.5	37.5	0
	125	s(S–C_{NHC})	S		1.19	0.66	43.3	18.6	80.9	0.6
			C	0.29		0.75	56.7	36.0	63.9	0.1
[{(WCA-IDipp)Se}Ir(COD)] **(12)**	267	LP(Se)	Se	0.19		1	100	0.1	99.9	0
	246	s(Ir–Se)	Ir	–0.13	0.79	0.51	26.0	25.8	49.8	24.4
			Se			0.86	74.0	15.7	84.3	0
	199	LP(Se)	Se			1	100	72.2	27.8	0
	159	s(Se–C_{NHC})	Se		1.10	0.62	38.7	12.2	87.8	0
			C	0.24		0.78	61.3	35.4	64.5	0.1

[{(WCA-IDipp)**S**}Ir(COD] (**10**)

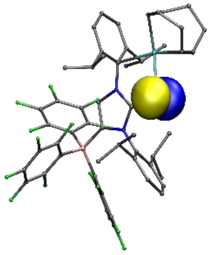

NBO #271

LP(S)

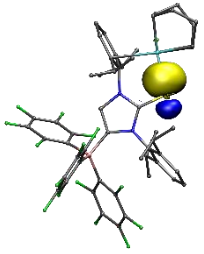

NBO #220

s(Ir–S)

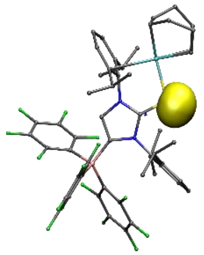

NBO #154

LP(S)

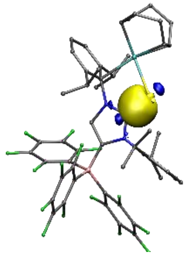

NBO #125

s(S–C_{NHC})

[{(WCA-IDipp)**Se**}Ir(COD] (**12**)

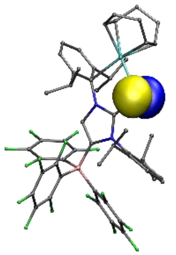

NBO #267

LP(Se)

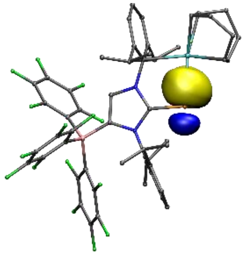

NBO #246

s(Ir–Se)

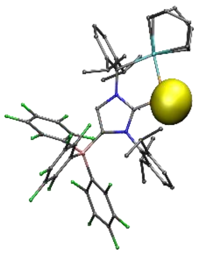

NBO #199

LP(Se)

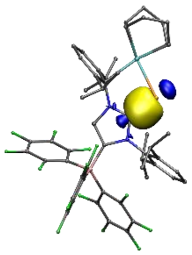

NBO #159

s(Se–C_{NHC})

S4 References

[1] CrysAlisPRO, Oxford Diffraction/Agilent Technologies UK Ltd, Yarnton, England.

[2] Sheldrick, G. M. *Acta Crystallogr., Sect. A: Found. Crystallogr*. **2008**, *64*, 112.

[3] Gaussian 16, Revision C.01, M. J. Frisch, G. W. Trucks, H. B. Schlegel, G. E. Scuseria, M. A. Robb, J. R. Cheeseman, G. Scalmani, V. Barone, G. A. Petersson, H. Nakatsuji, X. Li, M. Caricato, A. V. Marenich, J. Bloino, B. G. Janesko, R. Gomperts, B. Mennucci, H. P. Hratchian, J. V. Ortiz, A. F. Izmaylov, J. L. Sonnenberg, D. Williams-Young, F. Ding, F. Lipparini, F. Egidi, J. Goings, B. Peng, A. Petrone, T. Henderson, D. Ranasinghe, V. G. Zakrzewski, J. Gao, N. Rega, G. Zheng, W. Liang, M. Hada, M. Ehara, K. Toyota, R. Fukuda, J. Hasegawa, M. Ishida, T. Nakajima, Y. Honda, O. Kitao, H. Nakai, T. Vreven, K. Throssell, J. A. Montgomery, Jr., J. E. Peralta, F. Ogliaro, M. J. Bearpark, J. J. Heyd, E. N. Brothers, K. N. Kudin, V. N. Staroverov, T. A. Keith, R. Kobayashi, J. Normand, K. Raghavachari, A. P. Rendell, J. C. Burant, S. S. Iyengar, J. Tomasi, M. Cossi, J. M. Millam, M. Klene, C. Adamo, R. Cammi, J. W. Ochterski, R. L. Martin, K. Morokuma, O. Farkas, J. B. Foresman, and D. J. Fox, Gaussian, Inc., Wallingford CT, **2016**.

[4] X. Cao, M. Dolg, *J. Chem. Phys.,* **2001**, *115*, 7348.

[5] all Stuttgart RSC ECP basis sets (for Ir and Se) were obtained from the "Basis Set Exchange" website [https://www.basissetexchange.org/]. B. P. Pritchard, D. Altarawy, B. Didier, T. D. Gibson, T. L. Windus, *J. Chem. Inf. Model.* **2019**, *59*, 4814.

[6] a) for Ir: D. Andrae, U. Haeussermann, M. Dolg, H. Stoll, H. Preuss, *Theor. Chim. Acta* **1990**, *77*, 123; b) for Se: A. Bergner, M. Dolg, W. Kuechle, H. Stoll, H. Preuss, *Mol. Phys.* **1993**, *80*, 1431.

[7] a) J. P. Foster, F. Weinhold, *J. Am. Chem. Soc.,* **1980**, *102*, 7211; b) A. E. Reed, F. Weinhold, *J. Chem. Phys.,* **1983**, *78*, 4066; c) A. E. Reed, R. B. Weinstock, F. Weinhold, *J. Chem. Phys.,* **1985**, *83*, 735; d) A. E. Reed, F. Weinhold, *J. Chem. Phys.,* **1985**, *83*, 1736.
